# Total Synthesis of (+)-Euphorikanin A via an Atropospecific Cascade

**DOI:** 10.1021/jacs.3c11000

**Published:** 2023-12-05

**Authors:** Moritz J. Classen, Bilal Kicin, Vincent A. P. Ruf, Alexander Hamminger, Loélie Ribadeau-Dumas, Willi M. Amberg, Erick M. Carreira

**Affiliations:** Department of Chemistry and Applied Biosciences, Laboratory of Organic Chemistry, ETH Zürich, 8093 Zürich, Switzerland

## Abstract

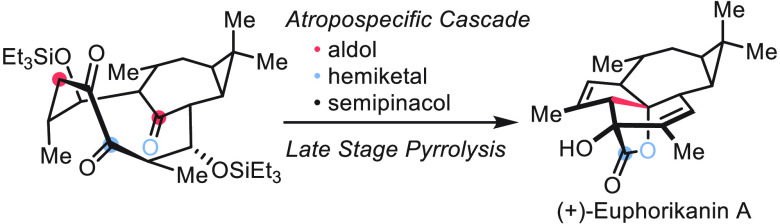

A total synthesis of the ingenane-derived diterpenoid (+)-euphorikanin A is described. Key to the strategy is a stereocontrolled one-pot sequence consisting of transannular aldol addition reaction, hemiketal formation, and subsequent semipinacol rearrangement that efficiently leads to the complete euphorikanin skeleton. Atroposelective ring-closing olefin metathesis proved critical for the stereospecific cascade, leading to formation of a (*Z*)-bicyclo[7.4.1]tetradecenone core. An additional salient feature of the route is pyrolysis of a bis-methylxanthate to cleanly furnish the natural product.

The Euphorbiaceae plant family produces a range of bioactive natural products, most notably the ingenane diterpenoids,^1−4^ which have been the subject of extensive synthetic studies for decades.^5^ Isolated in 2016 by Zhang and co-workers from *Euphorbia kansui*, (+)-euphorikanin A (**1**) (Scheme 1) has been proposed to be a rearranged ingenane.^6^ The intriguing 5/6/7/3-fused tetracyclic skeleton in combination with the [3.2.1]-bridging γ-lactone renders (+)-euphorikanin A (**1**) a significant synthetic challenge.^7^ To date, two total syntheses of **1** have been reported. The first proceeded in 19 steps from (+)-carene and featured a reductive annulation reaction that installed ring-A and γ-lactone in one step.^8^ A second approach showcased a benzilic acid type rearrangement in the final step to access the γ-lactone in overall 30 steps from (+)-carene.^9^ Common to both approaches is the assembly of the A and B rings in a sequential manner. We envisioned an alternative strategy in which the A and B rings along with the γ-lactone would be constructed from a functionalized 10-membered ring in a single operation. Herein we report a novel synthesis of (+)-euphorikanin A (**1**). At the heart of the approach is a sequence of transformations that includes the formation of bicyclo[7.4.1]tetradecene ***I*** via ring-closing metathesis (RCM) and a cascade from trione ***II*** to the euphorikanin skeleton ***IV***. We showcase that the formation of atropisomeric bicyclic rings is influenced by the configuration of the acyclic chains in the RCM precursor. Conversion of ***II*** to ***IV*** proceeds in one pot through a sequence of reactions consisting of transannular aldol addition, formation of hemiketal ***III***, and semipinacol rearrangement to afford the complete skeleton of euphorikanin A. The stereochemical challenge presented by target **1** is thus addressed by a series of atropospecific transformations.

**Scheme 1:**
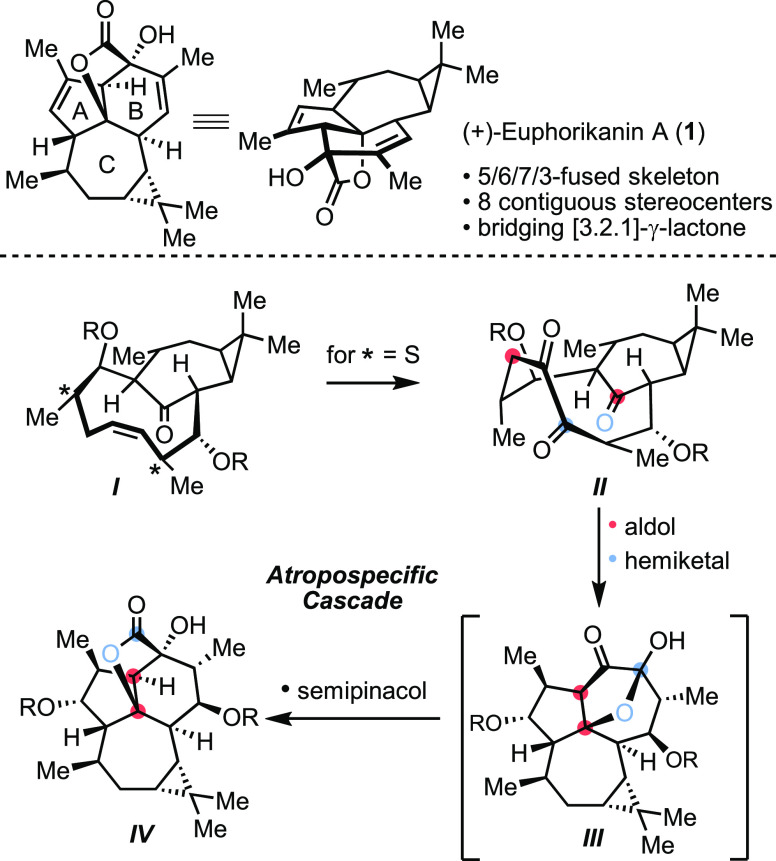
(+)-Euphorikanin A and Key Synthetic Steps

In our retrosynthetic analysis, we considered the proposed biosynthetic pathway by Zhang and co-workers in which dideoxyingenane-derived ***V*** is a precursor to the euphorikanin skeleton (Scheme 2). We envisioned ***II*** as a keystone intermediate that would enable the reaction cascade, providing rapid and efficient access to (+)-euphorikanin A (**1**). The 1,2-diketone in ***II*** could be derived from an olefin, which might be assembled by ring-closing metathesis of ***VI***. In turn, this intermediate would be accessed by two sequential aldol addition reactions of the corresponding cycloheptenone derived from (+)-carene (see **2**, Scheme 3).^8,10^ The formation of 10-membered rings via RCM is known to be difficult.^11−13^ More specifically, to the best of our knowledge there is no example of bicyclo[7.4.1.]tetradecene assembly by RCM. In this respect, the stereochemical features that arise from aldol addition reactions provide opportunities for examining a wide range of stereochemical permutations that could preorganize the side chains to favor cyclodecene formation.

**Scheme 2:**
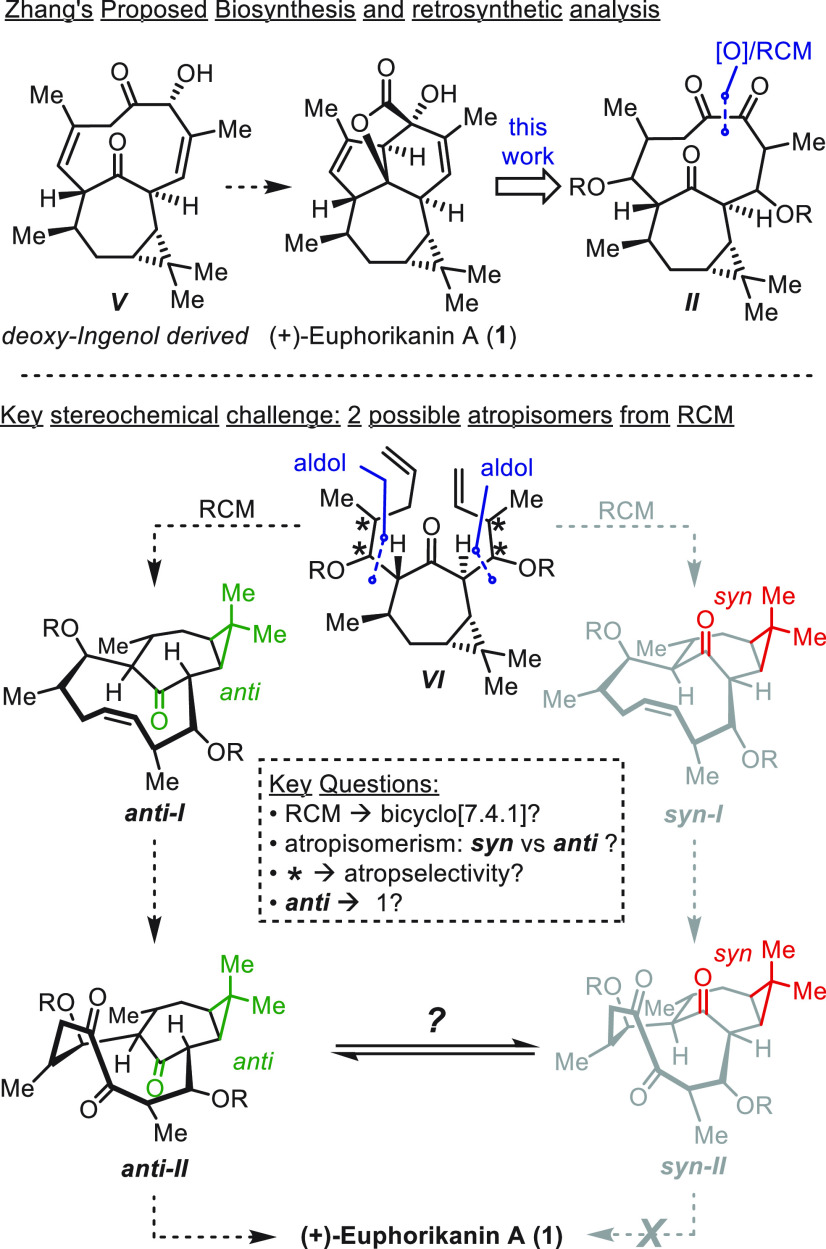
Retrosynthetic Analysis

**Scheme 3:**
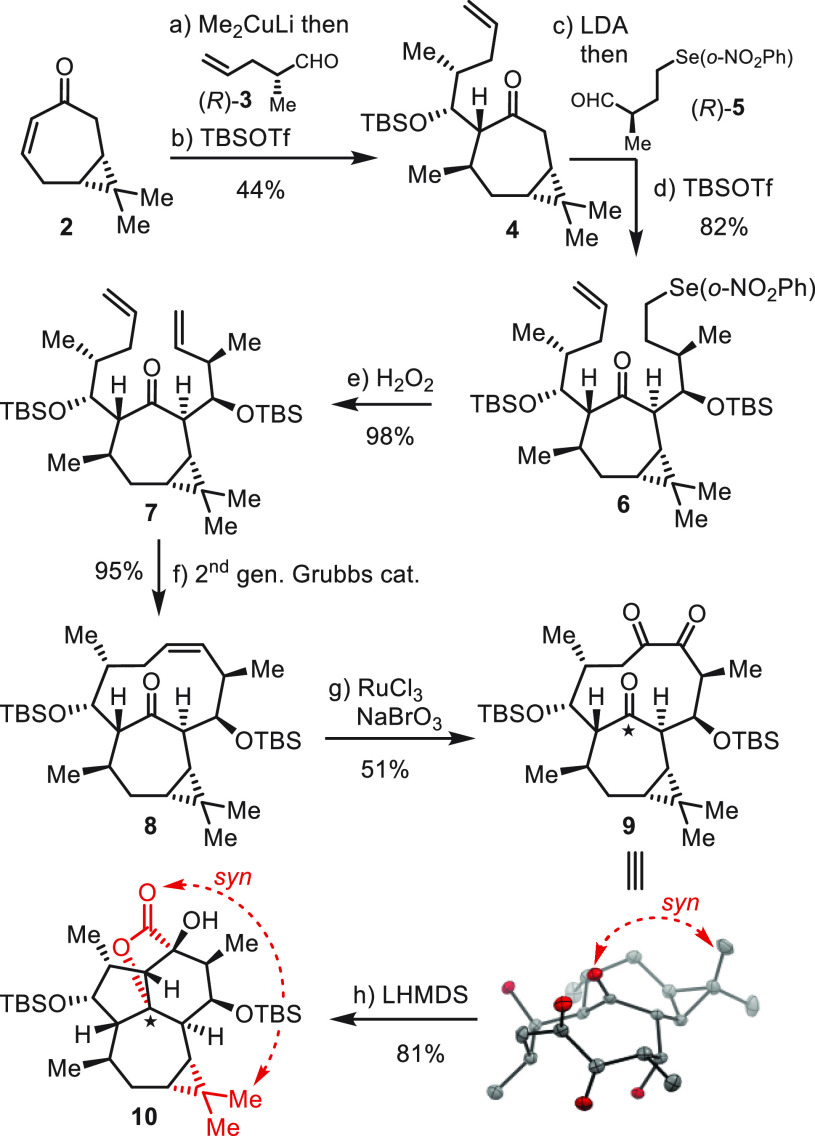
Initial Synthesis of Key Intermediate **13** Reagents and conditions: (a) Me_2_CuLi, Et_2_O, −78 °C, then (*R*)-**3**, THF, −78 °C to rt; (b) 2,6-lutidine, *t*-BuMe_2_SiOTf (TBSOTf), CH_2_Cl_2_, −78 °C to rt, (44% over two steps); (c) LiN(*i*-Pr)_2_ (LDA), −78 °C, THF, then (*R*)-**5**, (93%); (d) 2,6-lutidine, *t*-BuMe_2_SiOTf (TBSOTf), CH_2_Cl_2_, −78 °C, (88%); (e) H_2_O_2_, THF–pH 7 buffer (10:1), 0 °C to rt, (98%); (f) second-generation Grubbs catalyst (20 mol %), PhMe, 100 °C, (95%); (g) RuCl_3_·H_2_O (60 mol %), NaBrO_3_, NaHCO_3_, EtOAc–MeCN–H_2_O (6:6:1), 51%; (h) LiN(SiMe_3_)_2_ (LHMDS), 0 °C to rt, (81%). TBS = *t*-BuMe_2_Si–. In ORTEP drawings: H atoms and protecting groups (SiMe_2_*t*-Bu) were omitted for clarity.

It is important to note that the bicyclo[7.4.1] system exists as two possible atropisomers, as shown for ***anti*****-*****I*** and ***syn-I*** (Scheme 2).^14,15^ For a specific bridgehead configuration (*R*,*R*) as shown for ***anti*****-*****I*** and ***syn-I***, the *trans* intrabridgehead stereochemical relationships in each atropisomer can be described as two conformers differing in the position of the ketone above or below the plane defined by the bridging cyclodecene. In our analysis, ***anti-II*** and (+)-euphorikanin A (**1**) share the *anti*-relationship between cyclopropane and intrabridged ketone or γ-lactone, respectively. This leads to intriguing questions: (1) Can RCM be relied upon to furnish the bicyclo[7.4.1]tetradecenone? (2) Is there a preference for one atropisomer, and if so, are they interconvertible?^16^ (3) Can atroposelectivity in cyclodecene formation be influenced by the configuration of the side chains? (4) Will triketone ***anti*****-*****II*** engage in cascades that lead to (+)-euphorikanin A (**1**)?

The synthesis commenced with conjugate addition of Me_2_CuLi to enone **2** followed by treatment of the resulting enolate with aldehyde (*R*)-**3** (Scheme 3).^17^ The adduct alcohol was then treated with *t*-BuMe_2_SiOTf (TBSOTf), giving silyl ether **4** as a single diastereomer in 44% yield. The second side chain was introduced by site-selective enolization of **4** with LiN(*i*-Pr)_2_ and aldol addition with (*R*)-**5**.^18−20^ Subsequent silylation of the secondary alcohol with TBSOTf gave **6** in 82% yield.^21^

Treatment of **6** with H_2_O_2_ led to elimination, resulting in the formation of diolefin **7** in 98% yield. We then proceeded to investigate conditions for the RCM reaction. In the initial attempt, treatment of **7** with second-generation Grubbs catalyst^22^ in dichloromethane at 40 °C failed to give cyclodecene **8**, leading to reisolation of starting material. When the reaction was conducted in toluene at 100 °C, **8** was isolated in 95% yield. Inspection of the ^1^H and ^13^C NMR spectra for **8** indicated a single isomer, leading to the conclusion that the reaction was atroposelective. It was not possible to unambiguously determine the configuration at this point. Our next objective was to oxidize the alkene in **8**. Attempts involving a variety of conditions from the olefin that would provide diols, epoxides, and hydroxyketones were unproductive. Eventually, treatment of **8** with RuCl_3_ and NaBrO_3_^23^ resulted in the formation of triketone **9** in 51% yield.

At this stage of the synthesis, **9** was obtained as a crystalline solid from dichloromethane. Examination of the crystal structure provided insight into the relative configuration of the atropisomeric 10-membered ring, exhibiting the *syn*-configuration. A consequence of this arrangement (*syn*-relationship between the cyclopropane and intrabridging carbonyl) is that the intrabridgehead ketone (★) is poised to undergo transannular aldol addition at the *re*-diastereoface to afford a configuration that differs from that of the natural product.

Along the line of this analysis, treatment of triketone **9** with LiN(SiMe_3_)_2_ in THF at 0 °C for 30 min and 2 h at rt led to the isolation of a single product in 81% yield. We were able to obtain single crystals suitable for X-ray analysis. Crystallographic analysis confirmed the connectivity determined by NMR. Inspection of the X-ray structure revealed that **10** is unsuitable for the synthesis of (+)-euphorikanin A (**1**) because it bears the *syn*-relationship between the cyclopropane and the lactone subunit. At this stage, experiments were conducted to assess the ability of **8** or **9** to undergo atropisomerization. Heating a solution of either in *d*_8_-toluene at 20–100 °C and monitoring by ^1^H NMR spectroscopy returned the starting material unchanged. Since the interconversion of atropisomers was unsuccessful, we took advantage of the modular nature of the route described above to examine whether another diastereomer of RCM precursor ***VI*** would lead to ***anti***-***I****.*

We embarked on a second-generation synthesis that commences with enantiomeric aldehydes (*S*)-**3** and (*S*)-**5** and proceeds through otherwise identical steps (Scheme 4).

**Scheme 4:**
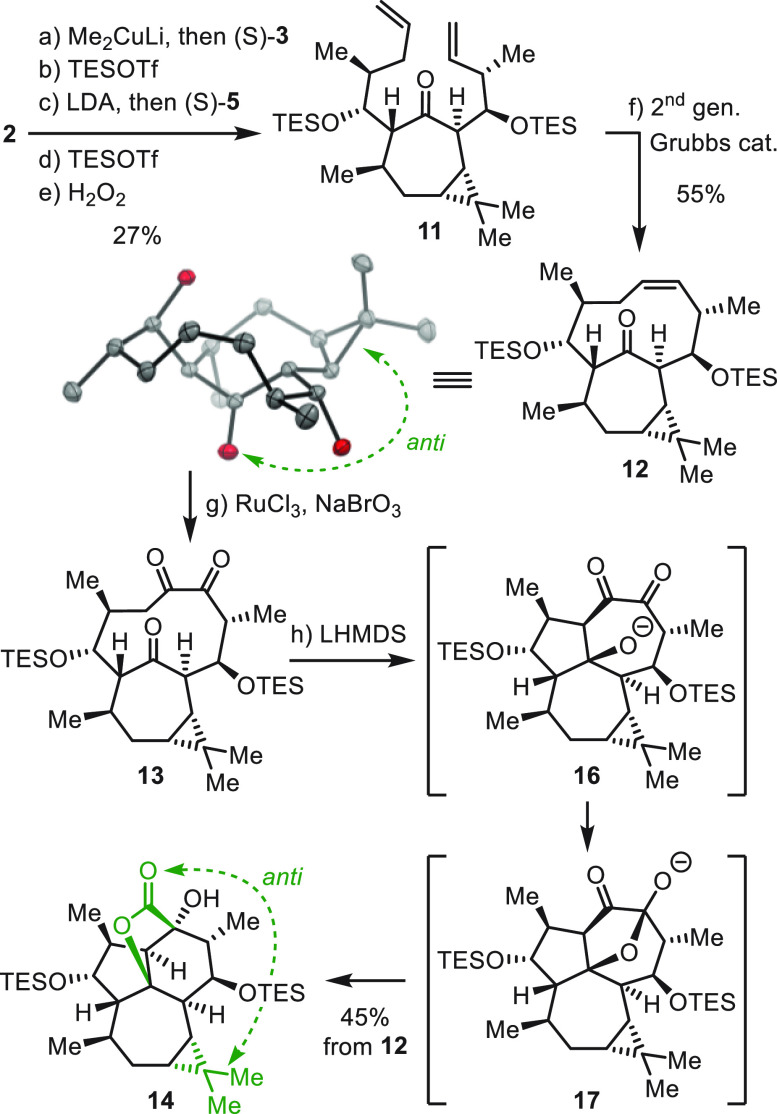
Second Synthesis of Key Intermediate **14** Reagents and conditions: (a) Me_2_CuLi, Et_2_O, −78 °C, then (*S*)-**3**, THF, −78 °C to rt, (45%); (b) 2,6-lutidine, Et_3_SiOTf (TESOTf), CH_2_Cl_2_, −78 °C to rt, (94%); (c) LiN(*i*-Pr)_2_ (LDA), −78 °C, THF, then (*S*)-**5**, (94%); (d) 2,6-lutidine, Et_3_SiOTf (TESOTf), CH_2_Cl_2_, −78 °C, (78%); (e) H_2_O_2_, THF–pH 7 buffer (10:1), 0 °C to rt, (90%); (f) second-generation Grubbs catalyst (20 mol %), PhMe, 100 °C, (55%); (g) RuCl_3_·H_2_O (60 mol %), NaBrO_3_, NaHCO_3_, EtOAc–MeCN–H_2_O (6:6:1); (h) LiN(SiMe_3_)_2_ (LHMDS), THF, 0 °C to rt, (45% over 2 steps). TES = Et_3_Si–. In ORTEP drawings: H atoms and protecting groups (SiEt_3_) were omitted for clarity.

As such, we prepared RCM precursor **11** from enone **2** in 27% yield over five steps.^24^ Initially, when **11** was subjected to RCM, bicyclo[7.4.1]tetradecenone **12** was isolated in 55% yield as a crystalline solid. Analysis of the X-ray structure reveals that it exhibits an *anti*-relationship between the cyclopropane and the bridging ketone. Thus, by permutation of the side chain configuration, we were able to influence the atropselectivity in the desired sense.

We next oxidized **12** to corresponding triketone **13** (RuCl_3_–NaBrO_3_), which without purification was treated with LHMDS and gave lactone **14** (single isomer) as a crystalline solid in 45% yield from **12**. Analysis of the X-ray structure revealed the configuration of lactone **14** as shown in Scheme 4, corresponding to that found in (+)-euphorikanin A (**1**).

To understand the stereochemical course of the cascade leading to **14**, we propose the pathway shown in Scheme 5. Atropisomer **13** is disposed to form enolate **15**, whose *si*-face then attacks the *si*-face of the intrabridging ketone, producing product **16** with the configuration at C-3 and C-4 found in (+)-euphorikanin A (**1**). The configuration at C-4 then dictates hemiketal formation. From **17**, the C-13–C-15 bond is stereoelectronically aligned to undergo semipinacol rearrangement and deliver the (+)-euphorikanin A scaffold (**14**). In a single operation, thereby, the 5/6/7 fused ring system and the bridging γ-lactone are established along with three contiguous stereocenters.

**Scheme 5:**
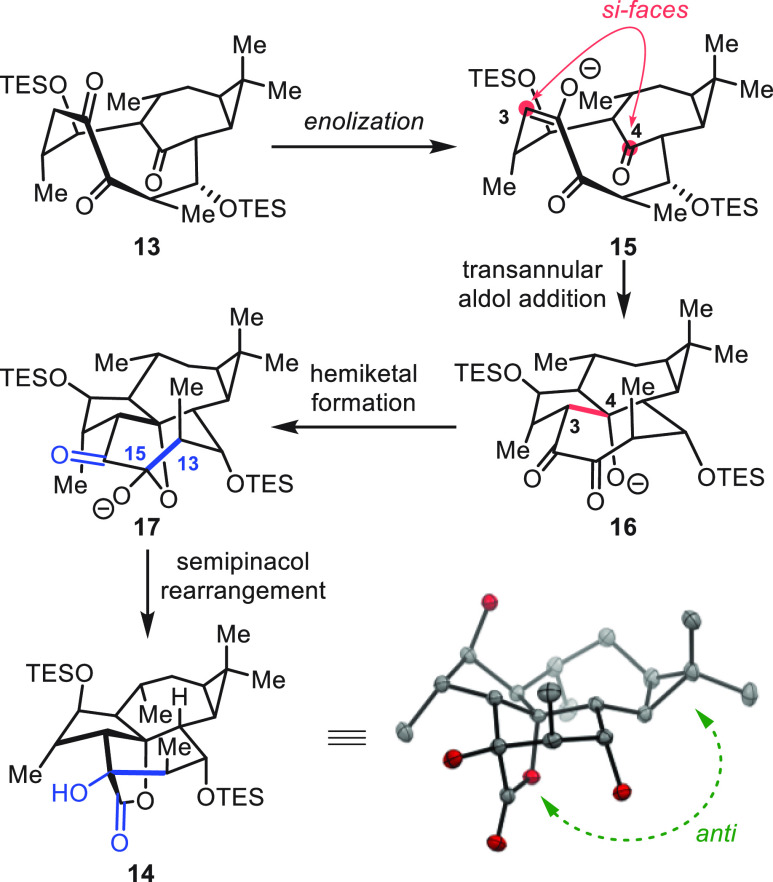
Stereochemical Analysis of the Atropospecific Cascade In ORTEP drawings: H atoms and protecting groups (SiEt_3_) were omitted for clarity.

To complete the synthesis, **14** was treated with *n*-Bu_4_NF to furnish triol **18** (99%) (Scheme 6). Subjection of the triol to Burgess’ reagent, Martin sulfurane, SOCl_2_/py, or Tf_2_O/K_2_CO_3_ failed to give **1**. Instead, selective formation of the methylxanthates afforded **19** in 91% yield.^25^ Subjecting **19** to standard Chugaev elimination conditions (mesitylene 160 °C, 1,2-dichlorobenzene 180 °C, microwave irradiation), merely led to complex mixtures.^26^

**Scheme 6:**
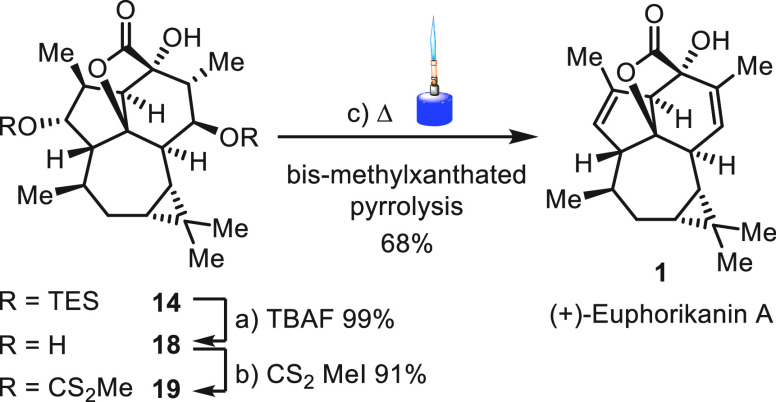
Completion of the Synthesis Reagents and conditions: (a) *n*-Bu_4_NF (TBAF), THF, 0 °C to rt, (99%); (b) DBU, CS_2_, DMF, then MeI, (91%); (c) Δ (Bunsen burner), 0.36 mbar, (68%).

Borrowing from techniques of flash vacuum pyrolysis,^27,28^ we devised a procedure in which **19** was heated under reduced pressure with an open flame and the product condensed at −78 °C.^29^ After purification, (+)-euphorikanin A (**1**) was isolated in 68% yield, successfully concluding the synthesis. All analytical data (^1^H NMR, ^13^C NMR, IR, HRMS, [α]_D_) of synthetic **1** were in agreement with those of natural and previously synthesized material.

In conclusion, we report a novel total synthesis of (+)-euphorikanin A (**1**) in 15 steps from (+)-3-carene. Ring-closing olefin metathesis leads to a (*Z*)-bicyclo[7.4.1]tetradecenone core, which exists as two possible atropisomers. A key observation from the study is that the configuration of the side chains influences atropisomer formation, which proved critical for the subsequent atropospecific cascade. The sequence consisted of transannular aldol addition reaction, hemiketal formation, and subsequent semipinacol rearrangement to efficiently furnish the complete skeleton of (+)-euphorikanin A (**1**). Strategically, the work more broadly demonstrates that fused, polycyclic frameworks can be prepared efficiently from medium-sized bicyclic systems. Importantly, the generation of atropisomerism may increase the complexity of an intermediate in the route, however, with the payoff that it can serve as an element of stereocontrol and facilitate transannular transformations.

## Supporting Information Available

Experimental procedures and characterization data for all new compounds and X-ray crystallographic data (PDF)
