# Supplementary material for: Total Synthesis of (+)-Euphorikanin A via an Atropospecific Cascade
Source: J Am Chem Soc. 2023 Dec 5;145(50):27225–9. doi: 10.1021/jacs.3c11000 (PMC10739989; doi:10.1021/jacs.3c11000)
Supplement: Supplementary file 1 — ja3c11000_si_001.pdf [file ja3c11000_si_001.pdf]

ACCOMPANYING SUPPORTING INFORMATION

Total Synthesis of (+)-Euphorikanin A via an Atropospecific Cascade

Moritz J. Classen,[‡] Bilal Kicin,[‡] Vincent A. P. Ruf,[‡] Alexander Hamminger, Loélie Ribadeau-Dumas, Willi M. Amberg, Erick M. Carreira*

<erickm.carreira@org.chem.ethz.ch>

Eidgenössische Technische Hochschule Zürich, Vladimir-Prelog-Weg 3,

HCI, 8093 Zürich, Switzerland

Table of Contents

General Methods S2

Experimental Procedures and Spectral Data S3

NMR Sprectra S37

Comparison of Natural and Synthetic (+)-Euphorikanin A S56

Cristallographic Data S59

Sources S107

General Methods

Unless stated otherwise, reactions were performed in dry solvents and under an atmosphere of nitrogen. Dry solvents were either purchased from commercial sources, or for larger quantities obtained from a purification column composed of activated alumina.

Generally, chemicals were purchased from commercial sources (Acros, Alfa Aesar, Fluka, Sigma-Aldrich, TCI) and used without further purification.

Enone 2 was prepared in 4 steps according to literature procedure.[1, 2]

(*R*)-2-methylpent-4-en-1-ol was prepared in two steps according to literature procedure.[3]

(*S*)-2-methylpent-4-en-1-ol was prepared in two steps according to modified literature procedure (other enantiomer of Evans auxiliary).[3]

(*R*)-*tert*-butyl((2-methylbut-3-en-1-yl)oxy)diphenylsilane and **(*S*)-*tert*-butyl((2-methylbut-3-en-1-yl)oxy)diphenylsilane** were prepared in three steps from either (*R*)-Roche ester or (*S*)-Roche ester according to literature procedures.[4, 5]

NMR spectra were recorded on Bruker DRX and Avance (400 MHz) or DRXII (500MHz) spectrometers at room temperature. Signals are reported relative to the residual signal of the undeuterated solvent. Data are reported as follows: chemical shift, multiplicity (s = singlet, br s = broad singlet, d = doublet, t = triplet, q = quartet, quin = quintet, dd = doublet of doublets, etc.) and integration.

Flash column chromatography was performed manually using glass columns with Sigma-Aldrich, 60 Å, 230–400 mesh silica gel using laboratory grade solvents. For small scale column chromatography, Et_2O was distilled prior to use to remove BHT, and stored under Argon in a brown glass bottle, while regularly being checked for peroxide formation.

Thin layer chromatography was performed on glass plates pre-coated with silica gel (Merck, Silica gel 60 F_{254}). Compounds were visualized using UV light and/or standard staining techniques involving $KMnO_4$ or phosphomolybdic acid.

Infrared spectra (IR) were recorded on a Perkin Elmer Spectrum Two FT-IR (UATR) instrument as thin films and are reported as wavenumbers (cm^{-1}).

Optical rotation: Optical rotations were measured on a Jasco P-2000 Polarimeter, 10 cm, 1.5 mL cell.

High resolution mass spectra (HRMS) were recorded on a Bruker Daltonics maXis ESI-Q-TOF by the ETH Zurich MS service.

X-Ray diffraction was measured on a Bruker Kappa Apex II DUO system equipped with a graphite monochromator and analyzed by Dr. Michael Wörle, Dr. Nils Trapp and Mr. Michael Solar.

Experimental Procedures and Spectral Data

Route to wrong diasterioisomer 10

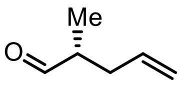

(*R*)-3

(*R*)-2-methylpent-4-enal ((*R*)-3):

A flame-dried and nitrogen-flushed round-bottomed flask was charged with (*R*)-2-methylpent-4-en-1-ol (2.38 g, 20.0 mmol, 2.00 eq), dry THF (45.0 mL) and IBX (11.2 g, 39.9 mmol, 4.00 eq). A reflux-condenser was mounted onto the reaction vessel and the suspension was heated to 80 °C under vigorous stirring for 4 h. After cooling to room temperature, the mixture was filtered, and the filter cake was washed with dry THF (5 mL). The filtrate was dried under nitrogen atmosphere for 30 min over Na_2SO_4 prior to use. An NMR of unpurified product confirmed it to be aldehyde **(*R*)-3** (80% purity), which was used in the next step without further purification as a 0.4 M stock solution in THF.

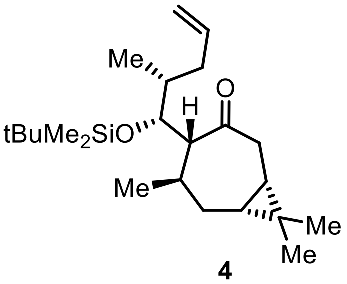

4

(1*S*,4*R*,5*R*,7*R*)-4-((1*R*,2*R*)-1-((*tert*-butyldimethylsilyl)oxy)-2-methylpent-4-en-1-yl)-5,8,8-trimethylbicyclo[5.1.0]octan-3-one (4): A flame-dried, nitrogen-flushed, 250-mL round-bottomed flask was charged with CuI (2.28 g, 12.0 mmol, 1.20 eq) and dry Et_2O (40.5 mL) and subsequently cooled to 0°C. After adding MeLi (15.0 mL, 1.60 M in Et_2O, 208 mmol, 2.40 eq) dropwise to the suspension the reaction mixture turned first yellow and then colorless. After stirring for 5 min the reaction was cooled to – 78 °C. Enone **2** (1.50 g, 10.0 mmol, 1.00 eq) dissolved in Et_2O (30.0 mL) was added dropwise and the reaction mixture was stirred for 30 min. After TLC analysis indicated consumption of the starting material (UV and phosphomolybdic acid stain), aldehyde **(*R*)-3** (50 mL of the freshly prepared 0.4 M in THF, assumed to be 20.0 mmol, 2.00 eq) was added dropwise. The reaction mixture was stirred for 2 h at – 78 °C and quenched by the addition of sat. aq. $NaHCO_3$ solution (60 mL). After extraction using Et_2O (3 x 250 mL) the combined organic layers were dried over Na_2SO_4, filtered, and concentrated under reduced pressure. Purification by chromatography on silica gel (hexanes/THF 95:5 to 80:20) afforded the aldol adduct as an inseparable mixture with (1*S*,5*R*,7*R*)-5,8,8-trimethylbicyclo[5.1.0]octan-3-one. The mixture was used without further purification in the next step.

A flame-dried, nitrogen-flushed 50-mL round-bottom flask was charged with dry CH_2Cl_2 (44.0 mL), the previously obtained mixture, consisting of the aldol adduct and (1*S*,5*R*,7*R*)-5,8,8-trimethylbicyclo[5.1.0]octan-3-one. The reaction was cooled to – 78 °C. Freshly distilled 2,6-lutidine (723 mg, 0.780 mL, 6.73 mmol, 0.673 eq) was added to the mixture followed by dropwise addition of *t*-$BuMe_2SiOTf$ (479 mg, 0.410 mL, 1.81 mmol, 0.181 eq). The reaction mixture was stirred for 1 h at – 78 °C and was then gradually warmed to room temperature over 4 h. After the reaction was quenched with sat. aq. NH_4Cl solution (26 mL) the layers were separated, and the aqueous layer was extracted 2 times with CH_2Cl_2 (2 x 80 mL). The combined organic layers were dried over Na_2SO_4, filtered, and concentrated under reduced pressure. Purification by chromatography on silica gel (hexanes/EtOAc 95:5 to 90:10) yielded the title compound **4** (1.60 g, 4.20 mmol, 42% over two steps) as a colorless oil.

^{1}H NMR (400 MHz, $CDCl_3$) δ = 5.84 – 5.66 (m, 1H), 5.11 – 4.91 (m, 2H), 4.00 (dd, *J* = 9.0, 2.0 Hz, 1H), 2.64 – 2.57 (m, 1H), 2.48 (ddd, *J* = 12.6, 7.6, 1.4 Hz, 1H), 2.33 (dd, *J* = 12.6, 9.2 Hz, 1H), 2.19 (dddt, *J* = 13.2, 6.6, 5.2, 1.5 Hz, 1H), 1.98 – 1.86 (m, 2H), 1.78 – 1.65 (m, 2H), 1.43 – 1.33 (m, 1H), 1.07 (s, 3H), 1.05 (d, *J* = 7.3 Hz, 6H), 0.94 (t, *J* = 7.9 Hz, 9H), 0.85 (d, *J* = 6.9 Hz, 3H), 0.74 – 0.68 (m, 1H), 0.65 – 0.56 (m, 7H).

^{13}C NMR (101 MHz, $CDCl_3$) δ = 210.8, 137.7, 116.1, 76.8, 63.7, 38.8, 38.4, 37.0, 31.7, 28.8, 26.1, 23.3, 21.5, 20.1, 19.4, 15.4, 13.4, 7.3, 5.8. **IR** (thin film, cm^{-1}): 3077, 2955, 2877, 1706, 1641, 1458, 1416, 1380, 1288, 1240, 1196, 1178, 1119, 1067, 1010, 967, 911, 861, 777, 737, 677, 642, 589, 532.

HRMS (ESI): *m/z*: exact mass calculated for $C_{23}H_{42}O_2Si$ $[M+Na]^+$, 401.2846; found 401.2841.

IR (thin film, cm^{-1}): 3480, 2957, 1686, 1639, 1459, 1412, 1378, 1259, 1134, 986, 907, 749.

$[a]_D^{25°}$ = +49.79 (c = 1.0, $CHCl_3$)

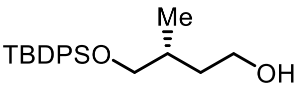

(*R*)-SI-1

(*R*)-4-((*tert*-butyldiphenylsilyl)oxy)-3-methylbutan-1-ol (*R*)-SI-1: An oven-dried 1 L round-bottomed flask was charged with olefin (*R*)-*tert*-butyl((2-methylbut-3-en-1-yl)oxy)diphenylsilane (10.4 g, 33.7 mmol, 1.00 equiv) and dry THF (300 mL) And subsequently cooled to 0 °C. To this mixture $BH_3 \bullet SMe_2$ complex (3.59 g, 4.70 mL, 10.0 M, 1.40 equiv) was added dropwise. The cooling bath was removed and the reaction mixture was stirred at room temperature for 3 h until complete consumption of the starting material was indicated by TLC analysis. The reaction mixture was poured onto ice cold 1 M aq. NaOH solution (750 mL) and aq. H_2O_2 solution (30 w%, 250 mL) was added. The mixture was allowed to warm to room temperature and was stirred for 2 h before the reaction was quenched with sat. aq. $NaHCO_3$ (500 mL) and sat. aq. $Na_2S_2O_3$ (200 mL) solution. The mixture was extracted with EtOAc (3 x 1 L), the combined organic layers were dried over Na_2SO_4, filtered, and concentrated under reduced pressure. Purification by column chromatography on silica gel (hexanes/Et_2O 90:10 to 80:20) yielded the title compound **(*R*)-SI-1** (8.00 g, 24.5 mmol, 73%) as a colorless oil.

^{1}H NMR (400 MHz, $CDCl_3$): δ = 7.69 – 7.65 (m, 4H), 7.46 – 7.37 (m, 6H), 3.69 (ddq, J = 17.5, 10.9, 6.7, 5.8 Hz, 2H), 3.56 – 3.47 (m, 2H), 2.13 (s, 1H), 1.83 (pd, J = 6.7, 5.2 Hz, 1H), 1.75 – 1.64 (m, 1H), 1.53 (dq, J = 13.9, 6.2 Hz, 1H), 1.06 (s, 9H), 0.90 (d, J = 6.8 Hz, 3H) ppm.

The analytical data was in accordance with that reported.[6]

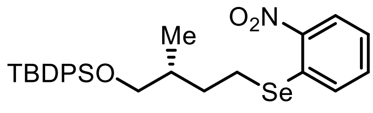

(*R*)-SI-2

(*R*)-*tert*-butyl(2-methyl-4-((2-nitrophenyl)selanyl)butoxy)diphenylsilane (R)-SI-2: A flame-dried and nitrogen-flushed 50-mL round-bottomed flask was charged with dry THF (18.0 mL) and 2-nitrophenyl selenocyanate (782 mg, 3.44 mmol, 1.20 eq) and subsequently cooled to 0 °C. After alcohol **(*R*)-SI-1** (0.933 mg, 2.86 mmol, 1.00 eq) dissolved in dry THF (7.00 mL) was added, tributyl phosphine (836 mg, 1.00 mL, 4.12 mmol, 1.44 eq) was added dropwise to the reaction mixture. The reaction was stirred for 20 min at 0 °C and subsequently quenched with sat. aq. $NaHCO_3$ solution (15 mL). The mixture was transferred to a separation funnel and diluted with Et_2O (20 mL). The layers were separated, and the aqueous layer was washed with Et_2O (3 x 20 mL). The combined organic layers were dried over Na_2SO_4, filtered, and concentrated under reduced pressure. Purification by column chromatography on silica gel (hexanes to hexanes/EtOAc 97:3) yielded the title compund **(*R*)-SI-2** (1.37 g,2.68 mmol, 78%) as a yellow oil.

^{1}H-NMR (400 MHz, $CDCl_3$): δ = 8.29 (ddd, *J* = 8.3, 1.2, 0.6 Hz, 1H), 7.65 (ddd, *J* = 8.1, 1.6, 0.7 Hz, 4H), 7.48 – 7.35 (m, 8H), 7.29 (ddd, *J* = 8.4, 5.9, 2.6 Hz, 1H), 3.59 – 3.49 (m, 2H), 2.96 – 2.82 (m, 2H), 1.98 (ddt, *J* = 13.6, 9.8, 6.1 Hz, 1H), 1.85 (dq, *J* = 12.7, 6.3 Hz, 1H), 1.65 – 1.56 (m, 2H), 1.04 (s, 9H), 0.98 (d, *J* = 6.7 Hz, 3H) ppm;

^{13}C-NMR (101 MHz, $CDCl_3$): δ = 135.8, 134.1, 133.9, 133.9, 133.6, 129.8, 129.2, 127.8, 126.6, 125.3, 68.4, 36.5, 32.0, 27.1, 24.1, 19.5, 16.7 ppm;

HRMS (ESI): *m/z*: exact mass calculated for $[C_{27}H_{33}NNaO_3SeSi]^+$ ($M+Na^+$): 550.1287, found: 550.1283;

IR (thin film, cm^{-1}): 2958, 2930, 2857, 1590, 1566, 1514, 1472, 1428, 1331, 1304, 1249, 1112, 1038, 852, 824, 784, 730, 703, 614, 505.

$[a]_D^{25^\circ}$= +3.2 (c = 1.0, $CHCl_3$).

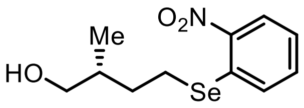

(*R*)-SI-3

(*R*)-2-methyl-4-((2-nitrophenyl)selanyl)butan-1-ol (*R*)-SI-3: A flame-dried and nitrogen-flushed 50 mL round-bottomed flask was charged with dry THF (25.0 mL) and alcohol **(*R*)-SI-2** (1.37 g, 2.68 mmol, 1.00 eq) and subsequently cooled to 0 °C. After the addition of tetrabutylammonium fluoride solution (1.05 mg, 4.03 mL, 1.00 M, 4.03 mmol, 1.50 eq) in THF, the cooling bath was removed, and the reaction mixture was stirred for 2 h at room temperature. The reaction was quenched by the addition of sat. aq. $NaHCO_3$ solution (15 mL). The mixture was transferred to a separation funnel, Et_2O (15 mL) was added, and the layers were separated. The aqueous layer was extracted with Et_2O (2 x 15 mL). The combined organic layers were washed with sat. aq. NaCl solution (20 mL) and dried over Na_2SO_4. After filtration and concentration under reduced pressure, the crude product was purified by column chromatography on silica gel (hexanes/EtOAc 85:15) yielding the title compound **(*R*)-SI-3** (745 mg, 2.58 mmol, 96%) as a yellow oil.

^{1}H-NMR (400 MHz, $CDCl_3$): δ = 8.29 (ddd, *J* = 8.3, 1.3, 0.5 Hz, 1H), 7.57 – 7.49 (m, 2H), 7.31 (ddd, *J* = 8.4, 6.4, 2.1 Hz, 1H), 3.56 (d, *J* = 5.9 Hz, 2H), 3.06 – 2.87 (m, 2H), 2.00 – 1.77 (m, 2H), 1.70 – 1.61 (m, 1H), 1.02 (d, *J* = 6.7 Hz, 3H) ppm;

^{13}C-NMR (101 MHz, $CDCl_3$): δ = 133.8, 133.7, 129.2, 126.7, 125.5, 67.8, 36.4, 32.0, 24.0, 16.5 ppm;

HRMS (ESI): *m/z*: exact mass calculated for $[C_{11}H_{15}NNaO_3Se]^+$ (M+Na$^+$): 312.0110; found: 312.0112.

IR (thin film, cm^{-1}): 3376, 2926, 1590, 1565, 1510, 1451, 1330, 1303, 1250, 1168, 1096, 1037, 981, 852, 783, 729, 702, 646.

[a]$_D$$^{25°}$ = +4.45 (c = 1.0, $CHCl_3$)

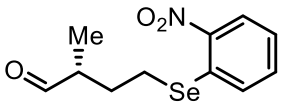

(*R*)-5

(*R*)-2-methyl-4-((2-nitrophenyl)selanyl)butanal (*R*)-5: A flame-dried and nitrogen-flushed 50 mL round-bottomed flask was charged with dry CH_2Cl_2 (16 mL), DMSO (1.45 g, 1.32 mL, 18.6 mmol, 10.0 eq) and alcohol **SI-3** (535 mg, 1.86 mmol, 1.00 eq). The solution was cooled to 0 °C and dry diisopropyl ethyl amine (1.44 mg, 1.90 mL, 11.1 mmol, 6.00 eq) was added, followed by the addition of SO_3 pyridine complex (1.33 g, 8.35 mmol, 4.50 eq) in one portion. After stirring for 30 min at 0 °C and 45 min at room temperature, the reaction mixture was quenched by the addition of sat. aq. NH_4Cl solution (10.0 mL). The mixture was transferred to a separation funnel, the layers were separated, and the aqueous layer was extracted with CH_2Cl_2 (2 x 15 mL). The combined organic layers were washed with sat. aq. NaCl solution (2 x 30 mL), dried over Na_2SO_4, filtered, and concentrated under reduced pressure. Purification by column chromatography on silica gel (hexanes/EtOAc 80:20) yielded the title compound **(*R*)-5** (453 mg, 1.58 mmol, 85%) as a yellow oil.

^{1}H-NMR (400 MHz, $CDCl_3$): δ = 9.69 (d, *J* = 1.3 Hz, 1H), 8.33 – 8.26 (m, 1H), 7.60 – 7.52 (m, 2H), 7.33 (ddd, *J* = 8.4, 6.4, 2.1 Hz, 1H), 2.95 (qdd, *J* = 12.0, 9.4, 6.1 Hz, 2H), 2.61 (tdd, *J* = 7.3, 6.0, 1.3 Hz, 1H), 2.20 (dddd, *J* = 14.2, 9.5, 7.2, 6.3 Hz, 1H), 1.83 (ddt, *J* = 14.2, 9.5, 6.1 Hz, 1H), 1.21 (d, *J* = 7.3 Hz, 3H) ppm;

^{13}C-NMR (101 MHz, $CDCl_3$): δ = 203.8, 133.9, 133.0, 129.1, 126.7, 125.7, 46.6, 29.3, 23.2, 13.6 ppm.

HRMS (ESI): *m/z*: exact mass calculated for [$C_{11}H_{13}NNaO_3Se$] $[M+Na]^+$, 309.9953, found 309.9951;

IR (thin film, cm^{-1}): 2930, 1721, 1590, 1566, 1512, 1453, 1331, 1304, 1250, 1097, 1037, 852, 784, 730.

$[a]_D^{25°}$ = +6.70 (c = 1.0, $CHCl_3$)

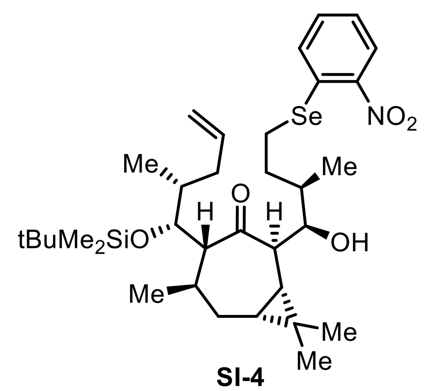

SI-4

(1*R*,2*R*,4*R*,5*R*,7*R*)-4-((1*R*,2*R*)-1-((*tert*-butyldimethylsilyl)oxy)-2-methylpent-4-en-1-yl)-2-((1*R*,2*R*)-1-hydroxy-2-methyl-4-((2-nitrophenyl)selanyl)butyl)-5,8,8-trimethylbicyclo[5.1.0]octan-3-one (SI-4): A flame-dried and nitrogen-flushed 25 mL round-bottomed flask was charged with ketone **4** (119 mg, 0.314 mmol, 1.00 eq) and dry THF (3.50 mL). The mixture was cooled to 0 °C and a freshly a prepared solution of LiN(*i*-Pr)$_2$ in THF (0.940 mL, 0.500 M, 471 mmol, 1.50 eq) was added dropwise. After the reaction mixture was stirred for 1 h, the mixture was cooled to –78 °C. Subsequently aldehyde **(*R*)-5** (0.270 g, 0.942 mmol, 3.00 eq) dissolved in THF (0.5 mL) was added dropwise. After 15 min the reaction was quenched by addition of sat. aq. NaHCO$_3$ solution (5 mL) and the mixture was diluted with Et$_2$O (5 mL). As soon as the aqueous layer completely melted, the layers were separated and the aqueous layer was extracted with EtOAc (2 x 10 mL). The combined organic layers were dried over Na$_2$SO$_4$, filtered, and concentrated under reduced pressure. Purification by column chromatography on silica gel (hexanes/EtOAc 95:5 to 80:20) yielded title compound **SI-4** (194 mg, 0.291 mmol, 93%) as a yellow oil.

^{1}H-NMR (400 MHz, CDCl$_3$): δ = 8.29 (dd, J = 8.3, 1.4 Hz, 1H), 7.61 – 7.48 (m, 2H), 7.30 (ddd, J = 8.4, 7.0, 1.4 Hz, 1H), 5.71 (ddt, J = 16.9, 10.4, 6.8 Hz, 1H), 5.04 – 4.96 (m, 2H), 3.82 – 3.70 (m, 2H), 3.12 (td, J = 10.8, 4.6 Hz, 1H), 2.81 (td, J = 10.7, 6.3 Hz, 1H), 2.69 (dd, J = 6.2, 3.1 Hz, 1H), 2.60 (t, J = 8.5 Hz, 1H), 2.39 (t, J = 9.3 Hz, 1H), 2.18 – 2.08 (m, 1H), 1.95 – 1.68 (m, 6H), 1.57 (ddd, J = 14.1, 9.6, 3.3 Hz, 2H), 1.14 (d, J = 6.5 Hz, 3H), 1.11 – 1.05 (m, 6H), 1.04 (s, 3H), 0.90 (m, 12H), 0.78 (td, J = 9.5, 5.8 Hz, 1H), 0.42 (t, J = 9.1 Hz, 1H), 0.09 (d, J = 5.9 Hz, 7H). ppm;

^{13}C-NMR (101 MHz, CDCl$_3$): δ = 215.9, 137.7, 133.7, 129.3, 126.6, 125.4, 116.1, 79.0, 78.1, 61.5, 51.1, 38.3, 38.2, 35.8, 33.1, 29.5, 29.1, 29.0, 26.6, 25.1, 24.4, 22.4, 20.5, 18.8, 17.6, 16.2, 15.9, –2.8, –3.9 ppm;

HRMS (ESI): calculated for [$C_{34}H_{56}NO_5SeSi$]+ (M+H$^+$): 666.3087, found: 666.3092.

IR (thin film, cm^{-1}): 3479, 3075, 2956, 2929, 2857, 1682, 1638, 1591, 1566, 1516, 1462, 1379, 1361, 1332, 1303, 1256, 1062, 1037, 994, 911, 835, 805, 774, 730, 703, 679, 646.

[a]$_D$$^{25°}$ = +58.8 (c = 1.0, CHCl$_3$)

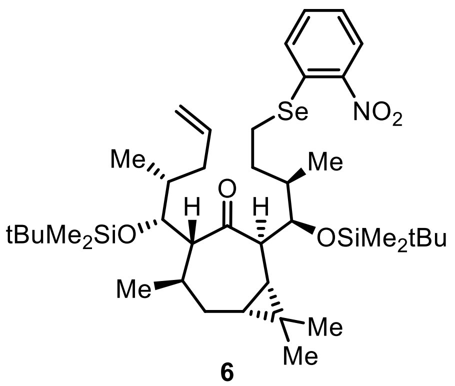

6

(1*R*,2*R*,4*R*,5*R*,7*R*)-2-((1*R*,2*R*)-1-((*tert*-butyldimethylsilyl)oxy)-2-methyl-4-((2-nitrophenyl)selanyl)butyl)-4-((1*R*,2*R*)-1-((*tert*-butyldimethylsilyl)oxy)-2-methylpent-4-en-1-yl)-5,8,8-trimethylbicyclo[5.1.0]octan-3-one (6): A flame-dried and nitrogen-flushed 10-mL round-bottomed flask was charged with dry CH_2Cl_2 (3.00 mL) and freshly distilled 2,6-lutidine (459 g, 0.500 mL, 4.26 mmol, 15.0 eq) and subsequently cooled to – 78 °C. After the addition of *t*-$BuMe_2SiOTf$ (751 mg, 0.653 mL, 2.84 mmol, 10.0 eq) the mixture was stirred for 1 min and alcohol **SI-4** (189 mg, 0.284 mmol, 1.00 eq) in dry CH_2Cl_2 (0.300 mL) was added slowly over the wall of the reaction flask. After the reaction mixture was stirred for 4 h at – 78 °C, it was quenched with sat. aq. NH_4Cl solution (2.5 mL). The mixture was diluted with CH_2Cl_2 (2.5 mL). As soon as the aqueous layer was melted completely, the layers were separated, and the aqueous layer was extracted with CH_2Cl_2 (2 x 20 mL). The combined organic layers were dried over Na_2SO_4, filtered, and concentrated under reduced pressure. The crude product was loaded onto silica gel. Purification by column chromatography on silica gel (hexanes/EtOAc 95:5 to 90:10) yielded title compound **6** (196 mg, 0.251 mmol, 88%) as a yellow oil.

^{1}H-NMR (400 MHz, $CDCl_3$): δ = 8.31 – 8.27 (m, 1H), 7.54 – 7.47 (m, 2H), 7.30 (ddd, *J* = 8.4, 5.9, 2.5 Hz, 1H), 5.75 (dddd, *J* = 16.5, 10.1, 7.8, 6.3 Hz, 1H), 5.03 – 4.93 (m, 2H), 4.05 (dd, *J* = 5.4, 4.1 Hz, 1H), 3.80 (dd, *J* = 4.1, 2.7 Hz, 1H), 3.06 (td, *J* = 11.0, 4.8 Hz, 1H), 2.84 (dd, *J* = 7.1, 2.7 Hz, 1H), 2.78 (td, *J* = 10.9, 6.1 Hz, 1H), 2.46 (dd, *J* = 9.6, 5.5 Hz, 1H), 2.40 (dd, *J* = 13.1, 5.5 Hz, 1H), 2.20 (tt, *J* = 7.2, 3.8 Hz, 1H), 2.11 – 2.00 (m, 1H), 1.82 – 1.74 (m, 1H), 1.63 (tt, *J* = 14.4, 4.5 Hz, 2H), 1.38 (ddd, *J* = 14.9, 11.3, 4.0 Hz, 1H), 1.18 (d, *J* = 6.7 Hz, 3H), 1.07 (s, 3H), 1.01 (d, *J* = 7.7 Hz, 6H), 0.94 – 0.90 (m, 12H), 0.88 – 0.82 (m, 10H), 0.49 (t, *J* = 9.4 Hz, 1H), 0.11 (s, 3H), 0.07 (s, 3H), 0.06 (s, 3H), 0.04 (s, 3H) ppm;

^{13}C-NMR (101 MHz, $CDCl_3$): δ = 212.9, 147.0, 138.3, 134.1, 133.7, 129.2, 126.6, 125.4, 115.7, 78.2, 78.1, 63.1, 54.3, 37.7, 37.4, 36.8, 32.3, 30.5, 29.8, 28.9, 26.6, 26.4, 24.8, 23.6, 21.7, 21.4, 20.4, 18.7, 18.6, 18.6, 17.6, 16.3, –3.0, –3.2, –4.3, –4.6 ppm;

HRMS (ESI): *m/z*: exact mass calculated for $[C_{40}H_{70}NO_5SeSi_2]^+$ (M+H$^+$): 780.3956, found: 780.3961.

IR (thin film, cm^{-1}): 2956, 2929, 2857, 1687, 1639, 1592, 1567, 1517, 1471, 1462, 1378, 1360, 1332, 1303, 1251, 1058, 1037, 1005, 938, 910, 835, 774, 729, 703, 677.

[a]$_D^{25°}$ = +43.6 (c = 1.0, $CHCl_3$)

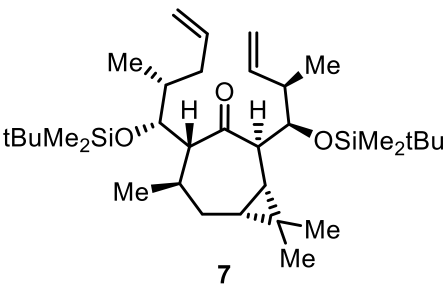

7

(1*R*,2*R*,4*R*,5*R*,7*R*)-2-((1*R*,2*R*)-1-((*tert*-butyldimethylsilyl)oxy)-2-methylbut-3-en-1-yl)-4-((1*R*,2*R*)-1-((*tert*-butyldimethylsilyl)oxy)-2-methylpent-4-en-1-yl)-5,8,8-trimethylbicyclo[5.1.0]octan-3-one (7): To a solution of seleno ether **6** (28.0 mg, 0.0353 mmol, 1.00 eq) in THF–Na_2HPO_4/KH_2PO_4 pH 7 buffer (10:1, 0.45 mL) at 0 °C was added aq. H_2O_2 solution (10.0 μL, 30 w%, 0.0980 mmol 2.80 eq). The cooling bath was removed, and the reaction mixture was stirred for 16 h at room temperature. Over the course of the reaction a discoloration of the yellow solution was observed. The reaction mixture was diluted with hexane (2 mL) and pH 7 buffer (5 mL) and transferred to a separation funnel. The layers were separated, and the aqueous layer was extracted with hexane (2 x 5 mL). The combined organic layers were dried over Na_2SO_4 and filtered. The hexane solution was loaded directly on a Column. Column chromatography on silica gel (hexanes/EtOAc 80:20) yielded titel compound **7** (20.0 mg, 0.0346 mmol, 98%) as a colorless oil.

^{1}H-NMR (400 MHz, $CDCl_3$): δ = 6.02 (ddd, J = 17.7, 10.5, 7.4 Hz, 1H), 5.77 (dddd, J = 16.3, 10.2, 7.9, 6.2 Hz, 1H), 5.05 – 4.94 (m, 4H), 4.06 (dd, J = 5.7, 3.4 Hz, 1H), 3.81 (t, J = 3.4 Hz, 1H), 2.90 (dd, J = 7.4, 3.1 Hz, 1H), 2.54 – 2.37 (m, 3H), 2.15 (tt, J = 7.2, 3.9 Hz, 1H), 2.01 – 1.91 (m, 1H), 1.88 – 1.76 (m, 1H), 1.61 (dt, J = 14.7, 4.3 Hz, 1H), 1.35 – 1.29 (m, 1H), 1.19 (d, J = 6.7 Hz, 3H), 1.07 (s, 3H), 1.03 (d, J = 7.1 Hz, 3H), 0.98 (s, 3H), 0.95 (d, J = 6.8 Hz, 3H), 0.91 (d, J = 5.8 Hz, 18H), 0.81 (ddd, J = 11.2, 9.2, 4.7 Hz, 1H), 0.70 (t, J = 9.1 Hz, 1H), 0.11 (s, 3H), 0.07 (s, 6H), 0.05 (s, 3H) ppm;

^{13}C-NMR (101 MHz, $CDCl_3$): δ = 213.5, 141.6, 138.4, 115.6, 114.2, 78.0, 77.7, 62.5, 55.4, 41.5, 37.1, 36.6, 32.4, 30.0, 28.8, 26.5, 26.4, 22.9, 21.6, 21.4, 20.2, 19.4, 18.7, 18.6, 18.6, 16.2, –3.1, –3.2, –4.4, –4.5 ppm;

HRMS (ESI): *m/z*: exact mass calculated for $[C_{34}H_{65}O_3Si_2]^+$ ($M+H^+$): 577.4467, found: 577.4467.

IR (thin film, cm^{-1}): 3076, 2956, 2929, 2857, 1689, 1640, 1472, 1462, 1417, 1378, 1361, 1253, 1054, 1005, 938, 909, 835, 774, 674.

[a]$_D^{27°}$ = +60.4 (c = 1.0, $CHCl_3$)

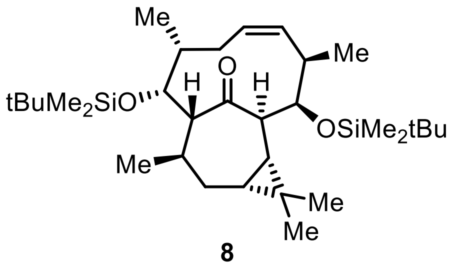

8

(1*R*,2*R*,3*R*,7*R*,8*R*,9*R*,10*R*,12*R*,14*R*,*Z*)-2,8-bis((*tert*-butyldimethylsilyl)oxy)-3,7,11,11,14-pentamethyltricyclo[7.5.1.010,12]pentadec-5-en-15-one (8): A flame-dried and argon-flushed 100-mL *Schlenk*-tube was charged with di-olefin **7** (20.0 mg, 0.0346 mmol,1.00 eq) and degassed-dry toluene (30.0 mL). Second generation Grubbs catalyst (5.90 mg, 690 µmol, 20.0 mol%) was added to the solution. The *Schlenk*-tube was capped, placed into a preheated oil bath and was stirred at 100 °C for 2 h. Consumption of the starting material was observed by TLC analysis on silvercoated TLC-plates. The heating bath was removed and after the reaction mixture cooled down to room temperature it was filtered through a plug of silica gel. The silica plug was washed with CH_2Cl_2 (3 x 15 mL). The filtrates were combined and concentrated under reduced pressure. Purification by column chromatography on silica gel (hexane to hexanes/EtOAc 100 to 95:05) yielded title compound **8** (18.0 mg, 0.0327 mmol, 95%) as a pale-yellow oil.

^{1}H-NMR (500 MHz, $CDCl_3$): δ = 5.83 (t, J = 10.3 Hz, 1H), 5.60 (td, J = 10.5, 6.9 Hz, 1H), 4.12 (dd, J = 10.5, 6.0 Hz, 1H), 4.00 (dd, J = 8.1, 1.8 Hz, 1H), 3.62 (d, J = 5.9 Hz, 1H), 3.04 – 2.97 (m, 1H), 2.76 (dt, J = 13.8, 10.5 Hz, 1H), 2.45 (ddd, J = 7.9, 6.6, 1.0 Hz, 1H), 1.69 (dt, J = 11.7, 6.3 Hz, 1H), 1.61 (dd, J = 10.2, 7.1 Hz, 1H), 1.50 – 1.39 (m, 2H), 1.16 (dd, J = 8.9, 6.7 Hz, 1H), 1.08 (s, 3H), 1.01 (d, J = 6.9 Hz, 3H), 0.95 – 0.93 (m, 16H), 0.91 (s, 9H), 0.72 (ddd, J = 13.0, 8.9, 4.2 Hz, 1H), 0.26 (d, J = 0.4 Hz, 3H), 0.06 – 0.05 (m, 3H), 0.04 (s, 6H) ppm;

^{13}C-NMR (126 MHz, $CDCl_3$): δ = 212.9, 133.4, 128.9, 77.7, 74.5, 59.3, 54.9, 40.3, 36.8, 29.9, 29.2, 28.1, 26.6, 263, 23.3, 21.6, 21.4, 20.5, 20.0, 19.8, 18.5, 18.3, 15.1, –2.4, –3.2, –3.5, –4.8 ppm;

HRMS (ESI): *m/z*: exact mass calculated for $[C_{32}H_{60}NaO_3Si_2]^+$ (M+Na$^+$): 571.3973, found: 571.3971.

IR (thin film, cm^{-1}): 2955, 2928, 2857, 1709, 1471, 1462, 1389, 1376, 1360, 1330, 1252, 1166, 1142, 1118, 1085, 1053, 1032, 1005, 993, 958, 937, 915, 886, 868, 833, 814, 796, 773, 742, 676.

[a]$_D$$^{25°}$ = +44.2 (c = 1.0, $CHCl_3$)

Various temperature experiments of 8:

8 was heated in d[8]-toluene from 20 °C to 100 °C. ^{1}H-NMR spectra were obtained at 10 °C intervals. After cooling down again to 20 °C an additional ^{1}H-NMR spectrum was obtained. All spectra were referenced to the methyl group of d[8]-toluene at 2.09 ppm (p, *J* = 2.2 Hz, 3H).

Spectra: 1 = 20 °C; 2 = 30 °C; 3 = 40 °C; 4 = 50 °C; 5 = 60 °C; 6 = 70 °C; 7 = 80 °C; 8 = 90 °C; 9 =100 °C; 10 = 20 °C.

No change for **8**, except the thermal induced ppm shift, was observed during the various temperature experiments.

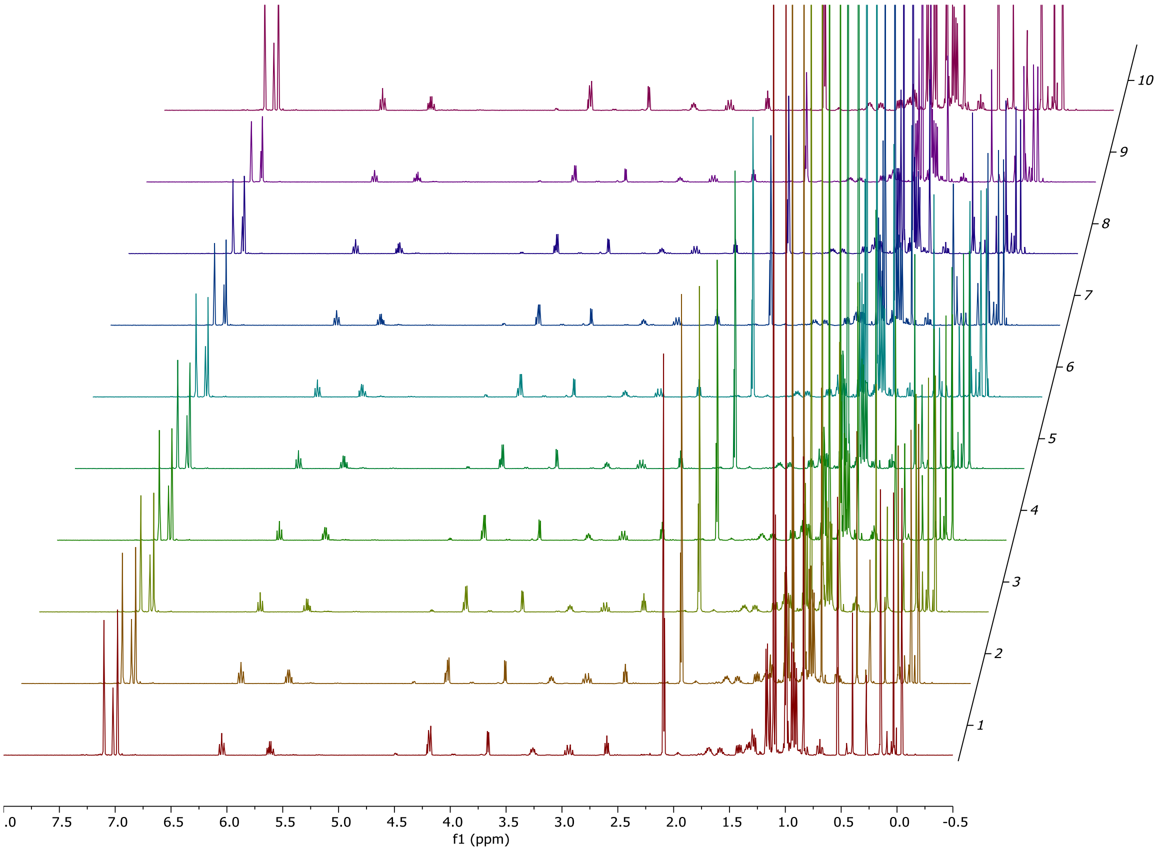

Compound **8** was heated in d[12]-mesitylene from 20 °C to 120 °C. ^{1}H-NMR spectra were obtained at 20 °C, 80 °C and 120 °C. After cooling down again to 20 °C an additional ^{1}H-NMR spectrum was obtained. All spectra were referenced to the methyl group of d[12]-mesitylene at 2.11 ppm (p, *J* = 2.2 Hz, 3H).

Spectra: 1 = 20 °C; 2 = 80 °C; 3 = 120 °C; 4 = 20 °C.

No change for **8**, except the thermal induced ppm shift, was observed during the various temperature experiments.

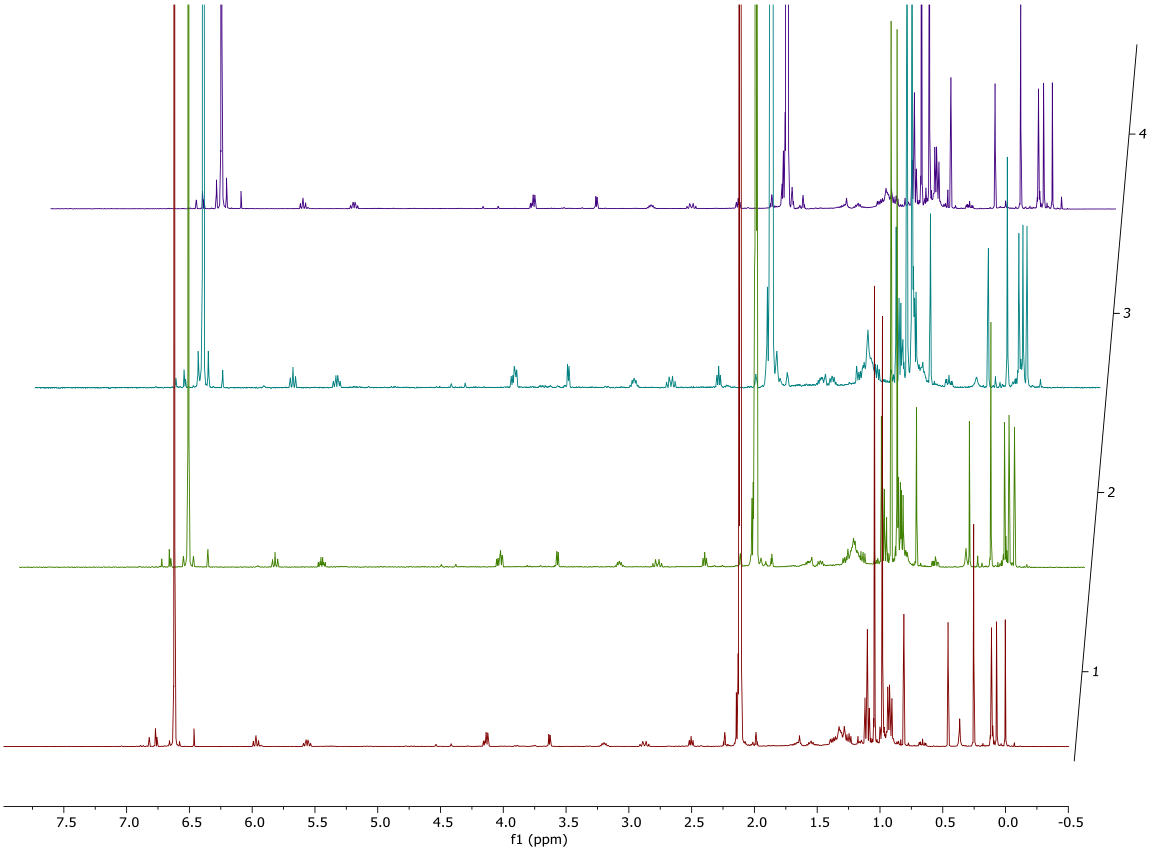

Compound **8** was heated in d[12]-mesitylene to 160 °C. ^{1}H-NMR spectra were obtained at before (Spectra 1) and after heating and cooling down to room temp (Spectra 2). The two spectra were

referenced to the methyl group of d[12]-mesitylene at 2.11 ppm (p, *J* = 2.2 Hz, 3H). No change for **8** was observed.

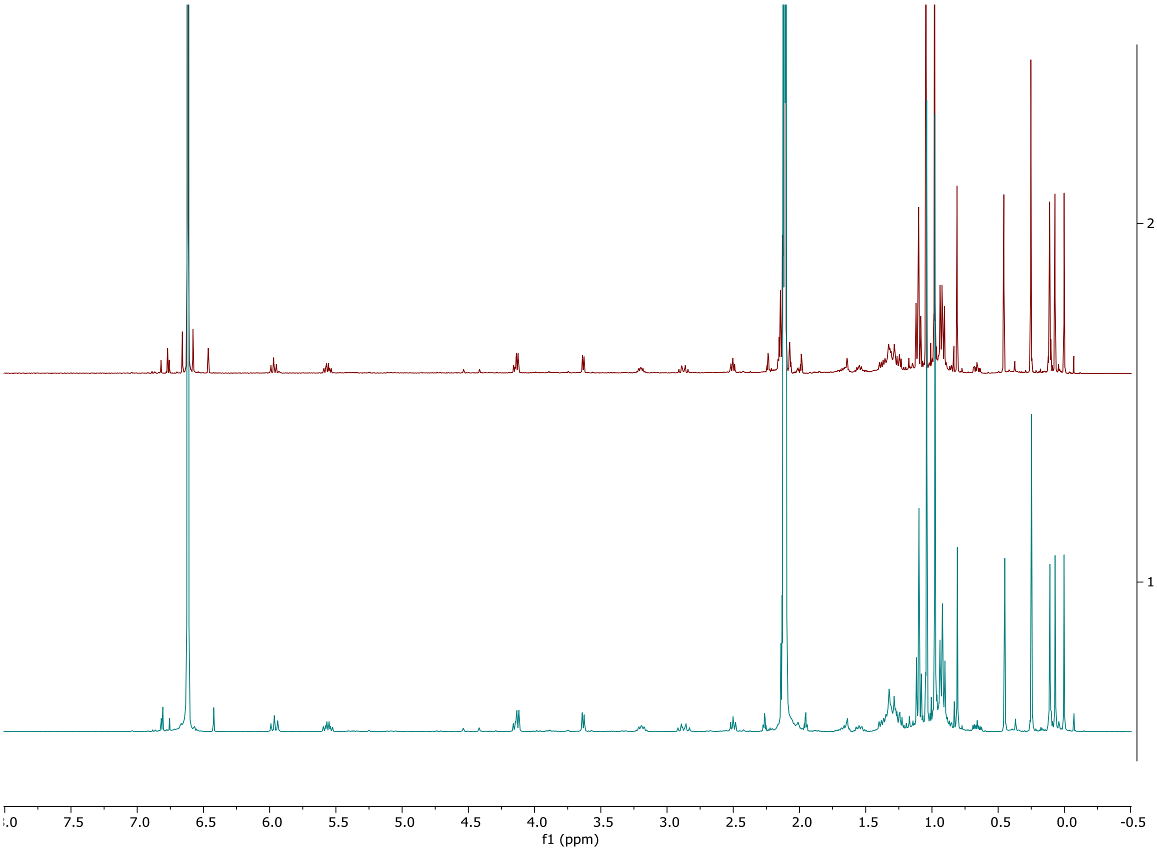

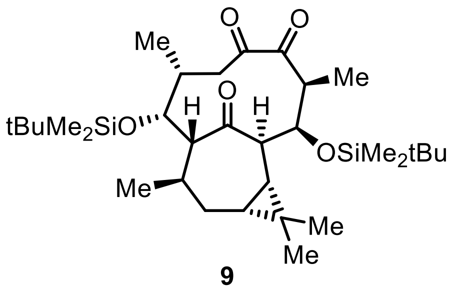

9

Reaction conditions were employed as described in the literature.[7]

(1*R*,2*R*,3*R*,7*S*,8*R*,9*R*,10*R*,12*R*,14*R*)-2,8-bis((*tert*-butyldimethylsilyl)oxy)-3,7,11,11,14-pentamethyltricyclo[7.5.1.010,12]pentadecane-5,6,15-trione (9): A 25 mL round-bottomed flask was charged with a 0.100 M solution of $NaHCO_3$ (0.820 mL, 0.0819 mmol, 3.00 eq) in water and a 0.100 M solution of $RuCl_3$•H_2O (0.160 mL, 0.0163 mmol, 0.60 eq) in water. To this solution $NaBrO_3$ (222 mg, 1.48 mmol, 54.0 eq) was added and the mixture was stirred for 5 min. Subsequently EtOAc (5.00 mL) and MeCN (5.70 mL) were added, and the mixture was stirred for 5 min. After this, a solution of **8** (15.0 mg, 0.0273 mmol, 1.0 eq) in EtOAc (0.700 mL) was added, and the reaction mixture was stirred for 2.5 h at room temperature. The reaction was quenched by the addition of a mixture of sat. aq. $NaHCO_3$ (6 mL) and sat. aq. $Na_2S_2O_3$ (2 mL) solution. After stirring the mixture for 15 min, it was transferred to a separation funnel, and it was diluted with CH_2Cl_2 (30 mL). The layers were separated, and the aqueous layer was extracted with CH_2Cl_2 (2 x 30 mL). The combined organic layers were dried over Na_2SO_4, filtered, and concentrated under reduced pressure. Purification by column chromatography on silica gel (hexanes/EtOAc 95:05 to 90:10) yielded title compound **9** (8.00 mg, 0.0138 mmol, 51%) as a crystalline solid. Single crystals suitable for X-Ray analysis were obtained by slow evaporation from CH_2Cl_2.

^{1}H-NMR (400 MHz, $CDCl_3$): δ = (d, J = 3.3 Hz, 1H), 3.87 – 3.79 (m, 1H), 3.66 (d, J = 2.8 Hz, 1H), 3.18 (dd, J = 16.9, 8.5 Hz, 1H), 2.99 (dd, J = 9.4, 2.8 Hz, 1H), 2.42 – 2.35 (m, 1H), 2.32 (dd, J = 5.9, 3.3 Hz, 1H), 2.24 – 2.15 (m, 2H), 1.62 – 1.57 (m, 1H), 1.27 (d, J = 7.2 Hz, 3H), 1.11 – 1.07 (m, 9H), 0.96 (d, J = 1.5 Hz, 20H), 0.88 (s, 3H), 0.81 (dd, J = 8.5, 5.8 Hz, 1H), 0.27 (s, 3H), 0.16 (s, 3H), 0.09 (s, 3H), 0.05 (s, 3H) ppm;

^{13}C-NMR (101 MHz, $CDCl_3$): δ = 216.2, 205.6, 205.2, 82.1, 75.0, 61.0, 60.4, 44.7, 40.3, 33.3, 33.1, 29.1, 28.3, 26.4, 26.3, 25.3, 23.7, 23.0, 20.3, 19.0, 18.9, 18.8, 17.7, 15.4, –2.9, –3.2, –4.5, –4.6 ppm.

HRMS (ESI): *m/z*: exact mass calculated for $[C_{32}H_{58}NaO_5Si_2]^+$ (M+Na$^+$): 601.3715, found: 601.3709.

IR (thin film, cm^{-1}): 2956, 2929, 2858, 1702, 1462, 1390, 1255, 1140, 1098, 1059, 1039, 1013, 979, 926, 898, 835, 804, 774.

[a]$_D$$^{30°}$ = +73.2 (c = 0.5, $CHCl_3$)

X-Ray crystallographic data: see page 59

Various temperature experiments of 9:

9 was heated in d[8]-toluene from 20 °C to 100 °C. ^{1}H-NMR spectra were obtained at 10 °C intervals. After cooling down again to 20 °C an additional ^{1}H-NMR spectrum was obtained. All spectra were referenced to the methyl group of d[8]-toluene at 2.09 ppm (p, *J* = 2.2 Hz, 1H).

Spectra: 1 = 20 °C; 2 = 30 °C; 3 = 40 °C; 4 = 50 °C; 5 = 60 °C; 6 = 70 °C; 7 = 80 °C; 8 = 90 °C; 9 =100 °C; 10 = 20 °C.

No change for **9**, except the thermal induced ppm shift was observed during the various temperature experiments.

When **9** was heated to higher temperatures, it started to undergo transannular aldol addition and slow decomposition.

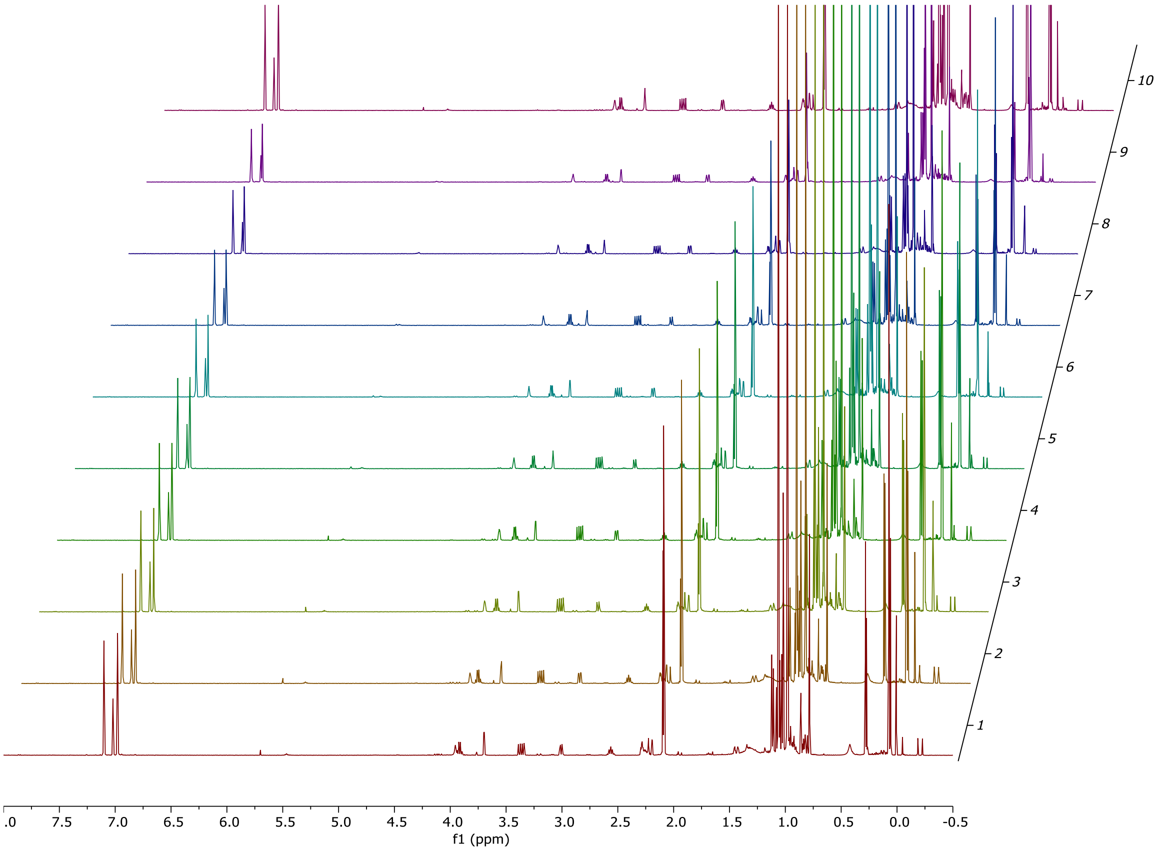

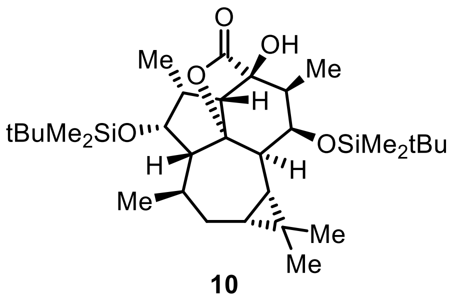

10

(1*R*,2*R*,2a*R*,2a[1]*S*,3*R*,4a*R*,5a*R*,5b*R*,6*R*,7*S*,8*S*,8a*R*)-2,6-bis((*tert*-butyldimethylsilyl)oxy)-8-hydroxy-1,3,5,5,7-pentamethyldodecahydro-1H-2a[1],8-(epoxymethano)benzo[*cd*]cyclopropa[*f*]azulen-9-one (10): In a 5 mL point-bottomed flask was added triketone **9** (7.50 mg, 0.0130 mmol, 1.00 eq) as a solution in THF (0.200 mL) and subsequently cooled to 0 °C. Next, a 1M solution of $LiN(SiMe_3)_2$ in THF (65.0 µL, 0.07 mmol, 5 eq) was added and the mixture was stirred at 0 °C for 1 h and then warmed to room temperature. After 3 h the reaction was quenched by the addition of sat. aq. NH_4Cl (3 mL). The mixture was transferred to a separation funnel diluted with CH_2Cl_2 (5 mL) and H_2O (2 mL). The layers were separated, and the aqueous layer was extracted with CH_2Cl_2 (2 x 5 mL). The combined organic layers were dried over Na_2SO_4, filtered, and concentrated under reduced pressure. Purification by column chromatography on silica gel (hexanes/Et_2O 90:10 to 80:20) yielded the title compound **10** (6.10 mg, 0.0110 mmol, 81%). Single crystals suitable for X-Ray analysis were obtained by slow evaporation from pentane.

^{1}H-NMR (400 MHz, $CDCl_3$): δ = 4.25 (dd, *J* = 5.8, 4.4 Hz, 1H), 3.90 (t, *J* = 4.0 Hz, 1H), 3.77 (d, *J* = 11.2 Hz, 1H), 2.47 – 2.38 (m, 2H), 2.32 (s, 1H), 2.14 (qd, *J* = 7.3, 3.7 Hz, 1H), 1.84 (ddd, *J* = 14.2, 10.3, 4.3 Hz, 2H), 1.58 – 1.54 (m, 1H), 1.45 – 1.39 (m, 1H), 1.13 – 1.07 (m, 9H), 1.06 (s, 3H), 1.00 (s, 3H), 0.92 (d, *J* = 1.9 Hz, 18H), 0.85 (d, *J* = 7.6 Hz, 1H), 0.81 – 0.77 (m, 1H), 0.09 (s, 3H), 0.05 (d, *J* = 2.1 Hz, 6H), 0.02 (s, 3H).

^{13}C-NMR (101 MHz, $CDCl_3$): δ = 77.36, 77.21, 75.63, 55.93, 46.45, 46.06, 45.54, 39.06, 29.86, 27.89, 27.03, 26.69, 26.24, 22.63, 21.01, 20.30, 18.72, 14.97, 14.14, 13.26, -2.39, -3.64, -3.70, -3.79.

HRMS (ESI): *m/z*: exact mass calculated for $[C_{32}H_{58}NaO_5Si_2]^+$ ($M+Na^+$): 601.3715, found: 601.3701.

IR (thin film, cm^{-1}): 2927, 2856, 1767, 1463, 1377, 1253, 1179, 1133, 1088, 1021, 954, 889, 868, 834, 801, 772.

$[a]_D^{28°}$ = +4.0 (c = 0.51, $CHCl_3$)

X-Ray crystallographic data: see page 68

Main Route to Euphorikanin A (1)

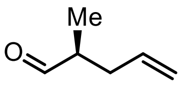

(*S*)-3

(S)-2-methylpent-4-enal (S)-3: A flame-dried and nitrogen-flushed round-bottomed flask was charged with (*S*)-2-methylpent-4-en-1-ol (2.38 g, 20.0 mmol, 2.00 eq), dry THF (45.0 mL) and IBX (11.2 g, 39.9 mmol, 4.00 eq). A reflux-condenser was mounted onto the reaction vessel and the suspension was heated to 80 °C with vigorous stirring for 4 h. After cooling to room temperature, the mixture was filtered, and the filter cake was washed with dry THF (5 mL). The filtrate was dried under nitrogen atmosphere for 30 min over Na_2SO_4 prior to use. An NMR of unpurified product confirmed it to be aldehyde **(*S*)-3** (80% purity), which was used in the next step without further purification as a 0.4 M stock solution in THF.

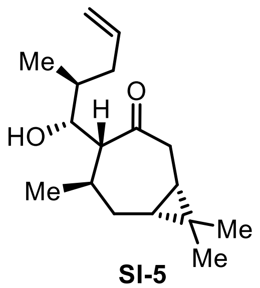

SI-5

(1*S*,4*R*,5*R*,7*R*)-4-((1*R*,2*S*)-1-hydroxy-2-methylpent-4-en-1-yl)-5,8,8-trimethylbicyclo[5.1.0]octan-3-one (SI-5): A flame-dried and nitrogen-flushed 250 mL round-bottomed flask was charged with CuI (2.28 g, 12.0 mmol, 1.20 eq) and dry Et_2O (40.5 mL) and subsequently cooled to 0 °C. After adding MeLi (15.0 mL, 1.60 M in Et_2O, 208 mmol, 2.40 eq) dropwise to the suspension, the reaction mixture turned first yellow and then colorless. After stirring for 5 min the reaction was cooled to – 78 °C. Enone **2** (1.50 g, 10.0 mmol, 1.00 eq) dissolved in Et_2O (30.0 mL) was added dropwise and the reaction mixture was stirred for 30 min. After TLC analysis indicated consumption of the starting material (UV and phosphomolybdic acid stain), aldehyde **(*S*)-3** (50 mL of the freshly prepared 0.4 M in THF, assumed to be 20.0 mmol, 2.00 eq) was added dropwise. The reaction mixture was stirred for 2 h at – 78 °C and quenched by the addition of sat. aq. $NaHCO_3$ solution (60 mL). After extraction using Et_2O (3 x 250 mL) the combined organic layers were dried over Na_2SO_4, filtered, and concentrated under reduced pressure. Purification by column chromatography on silica gel (hexanes/THF 95:5 to 80:20) yielded the title compound **SI-5** (1.19 g, 4.59 mmol, 45%) as a yellow oil.

^{1}H NMR (400 MHz, $CDCl_3$) δ = 5.79 (dddd, *J* = 17.1, 10.1, 8.3, 6.1 Hz, 1H), 5.10 – 4.97 (m, 2H), 3.65 (q, *J* = 5.6 Hz, 1H), 2.84 (t, *J* = 5.8 Hz, 1H), 2.61 (ddd, *J* = 15.1, 9.0, 1.0 Hz, 1H), 2.49 (d, *J* = 4.6 Hz, 1H), 2.45 – 2.37 (m, 2H), 2.20 – 2.11 (m, 1H), 1.95 – 1.84 (m, 1H), 1.74 (ddd, *J* = 15.1, 5.4, 3.8 Hz, 1H), 1.59 (ddt, *J*

= 9.3, 6.1, 3.2 Hz, 1H), 1.28 – 1.20 (m, 1H), 1.18 (dd, *J* = 7.0, 0.5 Hz, 3H), 1.09 (s, 3H), 0.98 (s, 3H), 0.93 (d, *J* = 6.8 Hz, 3H), 0.88 (ddd, *J* = 11.9, 8.9, 5.4 Hz, 1H), 0.71 (td, *J* = 8.9, 7.5 Hz, 1H).

^{13}C NMR (101 MHz, $CDCl_3$) δ = 214.6, 137.3, 116.5, 75.2, 59.7, 40.0, 36.6, 35.7, 29.9, 28.8, 27.6, 23.3, 21.0, 20.3, 19.1, 16.6, 15.3.

IR (thin film, cm^{-1}): 3467, 2957, 2927, 2873, 1687, 1640, 1459, 1415, 1378, 1290, 1224, 1196, 1177, 1140, 1064, 986, 955, 911, 642, 555.

$[a]_D^{25^\circ}$= 101.8 (c = 1.0, $CHCl_3$).

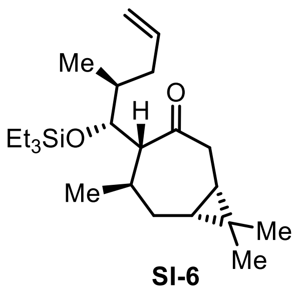

SI-6

(1*S*,4*R*,5*R*,7*R*)-5,8,8-trimethyl-4-((1*R*,2*S*)-2-methyl-1-((triethylsilyl)oxy)pent-4-en-1-yl)bicyclo[5.1.0]octan-3-one (SI-6): A flame-dried and nitrogen-flushed 50-mL round-bottomed flask was charged with dry CH_2Cl_2 (17.0 mL), and alcohol **SI-5** (456 mg, 1.73 mmol, 1.00 eq) and subsequently cooled to – 78 °C. Freshly distilled 2,6-lutidine (277 mg, 0.300 mL, 2.59 mmol, 1.50 eq) was added to the mixture followed by dropwise addition of Et_3SiOTf (479 mg, 0.410 mL, 1.81 mmol, 1.05 eq). The reaction mixture was stirred for 1 h at – 78 °C and was then gradually warmed to room temperature over 4 h. After the reaction was quenched with sat. aq. NH_4Cl solution (10 mL) the layers were separated, and the aqueous layer was extracted with CH_2Cl_2 (2 x 30 mL). The combined organic layers were dried over Na_2SO_3, filtered, and concentrated under reduced pressure. Purification by column chromatography on silica gel (hexanes/EtOAc 95:5 to 90:10) yielded the title compound **SI-6** (612 mg, 1.61 mmol, 94%) as a colorless oil.

^{1}H NMR (400 MHz, $CDCl_3$) δ = 5.84 – 5.66 (m, 1H), 5.11 – 4.91 (m, 2H), 4.00 (dd, *J* = 9.0, 2.0 Hz, 1H), 2.64 – 2.57 (m, 1H), 2.48 (ddd, *J* = 12.6, 7.6, 1.4 Hz, 1H), 2.33 (dd, *J* = 12.6, 9.2 Hz, 1H), 2.19 (dddt, *J* = 13.2, 6.6, 5.2, 1.5 Hz, 1H), 1.98 – 1.86 (m, 2H), 1.78 – 1.65 (m, 2H), 1.43 – 1.33 (m, 1H), 1.07 (s, 3H), 1.05 (d, *J* = 7.3 Hz, 6H), 0.94 (t, *J* = 7.9 Hz, 9H), 0.85 (d, *J* = 6.9 Hz, 3H), 0.74 – 0.68 (m, 1H), 0.65 – 0.56 (m, 7H).

^{13}C NMR (101 MHz, $CDCl_3$) δ = 210.8, 137.7, 116.1, 76.8, 63.7, 38.8, 38.4, 37.0, 31.7, 28.8, 26.1, 23.3, 21.5, 20.1, 19.4, 15.4, 13.4, 7.3, 5.8. **IR** (thin film, cm^{-1}): 3077, 2955, 2877, 1706, 1641, 1458, 1416, 1380, 1288, 1240, 1196, 1178, 1119, 1067, 1010, 967, 911, 861, 777, 737, 677, 642, 589, 532.

HRMS (ESI): *m/z*: exact mass calculated for $C_{23}H_{42}O_2Si$ $[M+Na]^+$, 401.2846; found 401.2841.

$[a]_D^{25°}$ = +129.5 (c = 1.0, $CHCl_3$)

Two step procedure to selenoether baring alcohol **SI-8**:

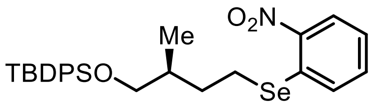

(*S*)-SI-7

(*S*)-*tert*-butyl(2-methyl-4-((2-nitrophenyl)selanyl)butoxy)diphenylsilane (*S*)-SI-7: A flame-dried and nitrogen-flushed 50-mL round-bottomed flask was charged with dry THF (18.0 mL) and 2-nitrophenyl selenocyanate (782 mg, 3.44 mmol, 1.20 eq) and subsequently cooled to 0 °C. After (*S*)-4-((*tert*-butyldiphenylsilyl)oxy)-3-methylbutan-1-ol (0.933 mg, 2.86 mmol, 1.00 eq) dissolved in dry THF (7.00 mL) was added, tributyl phosphine (836 mg, 1.00 mL, 4.12 mmol, 1.44 eq) was added dropwise to the reaction mixture. The reaction was stirred for 20 min at 0 °C and subsequently quenched with sat. aq. $NaHCO_3$ solution (15 mL). The mixture was transferred to a separation funnel, diluted with Et_2O (20 mL). The layers were separated, and the aqueous layer was extrected with Et_2O (3 x 20 mL). The combined organic layers were dried over Na_2SO_4, filtered and concentrated under reduced pressure. Purification by column chromatography on silica gel (hexanes to hexanes/EtOAc 97:3) yielded title compound **(*S*)-SI-7** (1.37 g, 2.68 mmol, 78%) as a yellow oil.

^{1}H NMR (400 MHz, $CDCl_3$) δ = 8.33 – 8.23 (m, 1H), 7.71 – 7.63 (m, 4H), 7.50 – 7.34 (m, 8H), 7.29 (ddd, *J* = 8.4, 6.2, 2.2 Hz, 1H), 3.55 (qd, *J* = 10.0, 5.7 Hz, 2H), 2.98 – 2.81 (m, 2H), 2.03 – 1.93 (m, 1H), 1.86 (dq, *J* = 12.6, 6.2 Hz, 1H), 1.63 (dddd, *J* = 13.3, 9.7, 7.3, 5.9 Hz, 1H), 1.05 (s, 9H), 0.98 (d, *J* = 6.7 Hz, 3H).

^{13}C NMR (101 MHz, $CDCl_3$) δ = 135.8, 135.7, 134.1, 133.9, 133.6, 129.8, 129.2, 127.8, 126.6, 125.3, 68.3, 36.4, 32.0, 27.0, 24.1, 19.4, 16.7.

IR (thin film, cm^{-1}): 2958, 2930, 2857, 1590, 1566, 1514, 1472, 1428, 1331, 1304, 1249, 1112, 1038, 852, 824, 784, 730, 703, 614, 505.

HRMS (ESI): *m/z*: exact mass calculated for $[C_{27}H_{33}NNaO_3SeSi]^+$ (M+Na^+): 550.1287, found: 550.1283;

$[a]_D^{25°}$= –3.2 (c = 1.0, $CHCl_3$).

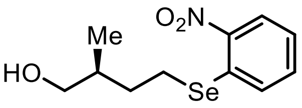

(*S*)-SI-8

(*S*)-2-methyl-4-((2-nitrophenyl)selanyl)butan-1-ol (*S*)-SI-8: A flame-dried and nitrogen-flushed 50-mL round-bottomed flask was charged with dry THF (25.0 mL) and TBDPS protected alcohol **(*S*)-SI-7** (1.37 g, 2.68 mmol, 1.00 eq) and subsequently cooled to 0 °C. After the addition of *n*-Bu_4NF solution (4.0 mL, 1.0 M, 4.0 mmol, 1.5 eq) in THF, the cooling bath was removed, and the reaction mixture was stirred for 2 h at room temperature. The reaction was quenched by the addition of sat. aq. $NaHCO_3$ solution (15 mL). The mixture was transferred to a separation funnel, Et_2O (15 mL) was added and the layers were separated. The aqueous layer was extracted with Et_2O (2 x 15 mL), the combined organic layers were washed with sat. aq. NaCl solution (20 mL) and dried over Na_2SO_4. After filtration and concentration under reduced pressure, the crude product was purified by column chromatography on silica gel (hexanes/EtOAc 85:15) yielding the title compound **(*S*)-SI-8** (745 mg, 2.58 mmol, 96%) as a yellow oil.

^{1}H NMR (400 MHz, $CDCl_3$) δ = 8.28 (ddd, *J* = 8.3, 1.3, 0.5 Hz, 1H), 7.60 – 7.47 (m, 2H), 7.31 (ddd, *J* = 8.4, 6.4, 2.1 Hz, 1H), 3.55 (dd, *J* = 5.9, 0.8 Hz, 2H), 3.06 – 2.89 (m, 2H), 2.00 – 1.89 (m, 1H), 1.89 – 1.78 (m, 1H), 1.64 (dddd, *J* = 13.2, 9.7, 7.5, 5.6 Hz, 1H), 1.41 (s, 1H), 1.01 (d, *J* = 6.7 Hz, 3H).

^{13}C NMR (101 MHz, $CDCl_3$) δ = 133.8, 133.7, 129.2, 126.6, 125.5, 67.7, 36.4, 31.9, 23.9, 16.5.

IR (thin film, cm^{-1}): 3368, 2 2957, 2926, 2874, 1590, 1565, 1510, 1452, 1432, 1330, 1304, 1251, 1169, 1150, 1097, 1037, 982, 852, 784, 729, 703, 680, 647.

HRMS (ESI): *m/z*: exact mass calculated for $[C11H15NNaO3Se]^+$ ($M+Na^+$): 312.0109, found: 312.0105;

$[a]_D^{25°}$= –5.0 (c = 1.0, $CHCl_3$).

One pot procedure to selenoether bearing alcohol **(*S*)-SI-8**:

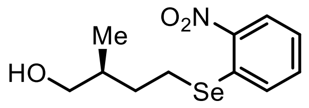

(*S*)-SI-8

(*S*)-2-methyl-4-((2-nitrophenyl)selanyl)butan-1-ol (*S*)-SI-8: A flame-dried and nitrogen-flushed 25-mL round-bottomed flask was charged with dry THF (1.60 mL) and 2-nitrophenyl selenocyanate (160 mg, 0.700 mmol, 1.15 eq) and subsequently cooled to 0 °C. After (*S*)-4-((*tert*-butyldiphenylsilyl)oxy)-3-methylbutan-1-ol (208 mg, 0.607 mmol, 1.00 eq) dissolved in dry THF (1.60 mL) was added, tributyl phosphine (181 mg, 0.220 mL, 0.840 mmol, 1.38 eq) was added dropwise to the reaction mixture. The reaction was stirred for 20 min at 0 °C and subsequently treated with *n*-Bu_4NF solution in THF (0.88 mL, 1.0 M, 0.88 mmol, 1.4 eq). The reaction mixture was allowed to warm to room temperature and stirred for an additional 45 min. The reaction was quenched with sat. aq. $NaHCO_3$ solution (5 mL). The mixture was transferred to a separation funnel, diluted with EtOAc (7 mL), the layers were separated, and the aqueous layer was washed with EtOAc (3 x 7 mL). The combined organic layers were dried over Na_2SO_4, filtered and concentrated under reduced pressure. Purification by column chromatography on silica gel (hexanes/EtOAc 80:20) yielded title compound **(*S*)-SI-8** (0.120 μg, 0.416 mmol, 69%) as a yellow oil. The analytical data was in accordance with **(*S*)-SI-8** prepared from the two-step procedure.

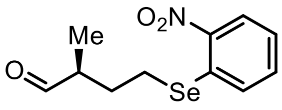

(*S*)-5

(*S*)-2-methyl-4-((2-nitrophenyl)selanyl)butanal (*S*)-5: A flame-dried and nitrogen-flushed 50-mL round-bottomed flask was charged with dry CH_2Cl_2 (16.0 mL), DMSO (1.45 g, 1.32 mL, 18.6 mmol, 10.0 eq) and alcohol **(*S*)-SI-8** (535 mg, 1.86 mmol, 1.00 eq). The solution was cooled to 0 °C and dry diisopropyl ethyl amine (1.44 mg, 1.90 mL, 11.1 mmol, 6.00 eq) was added, followed by the addition of SO_3 pyridine complex (1.33 g, 8.35 mmol, 4.50 eq) in one portion. After stirring for 30 min at 0 °C and 45 min at room temperature, the reaction mixture was quenched by the addition of sat. aq. NH_4Cl solution (10 mL). The mixture was transferred to a separatory funnel, the layers were separated, and the aqueous layer was extracted with CH_2Cl_2 (2 x 15 mL). The combined organic layers were washed with sat. aq. NaCl solution (2 x 30 mL), dried over Na_2SO_4, filtered, and concentrated under reduced pressure. Purification by column chromatography on silica gel (hexanes/EtOAc 80:20) yielded title compound **(*S*)-5** (453 mg, 1.58 mmol, 85%) as a yellow oil.

^{1}H NMR (400 MHz, $CDCl_3$) δ = 9.68 (d, *J* = 1.3 Hz, 1H), 8.33 – 8.25 (m, 1H), 7.61 – 7.49 (m, 2H), 7.33 (ddd, *J* = 8.4, 6.4, 2.1 Hz, 1H), 2.95 (qdd, *J* = 12.0, 9.4, 6.1 Hz, 2H), 2.60 (pdd, *J* = 7.3, 6.1, 1.3 Hz, 1H), 2.19 (dddd, *J* = 14.1, 9.5, 7.2, 6.4 Hz, 1H), 1.82 (ddt, *J* = 14.2, 9.5, 6.1 Hz, 1H), 1.21 (d, *J* = 7.2 Hz, 3H).

^{13}C NMR (101 MHz, $CDCl_3$) δ = 203.8, 133.9, 133.0, 129.1, 126.7, 125.7, 46.6, 29.2, 23.2, 13.6.

IR (thin film, cm^{-1}): 2969, 2932, 1721, 1590, 1566, 1511, 1453, 1397, 1331, 1304, 1250, 1169, 1097, 1038, 926, 852, 785, 730, 703, 647.

HRMS (ESI): *m/z*: exact mass calculated for [C11H13NNaO3Se]$^+$ (M+Na$^+$): 309.9953, found: 309.9951;

[a]$_D$$^{25°}$= –11.9 (c = 1.0, $CHCl_3$).

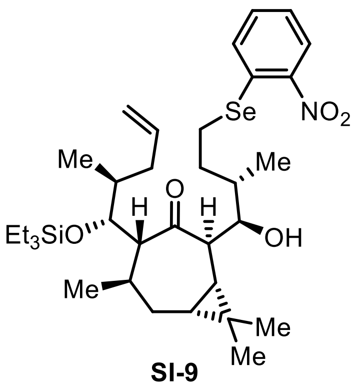

SI-9

(1*R*,2*R*,4*R*,5*R*,7*R*)-2-((1*R*,2*S*)-1-hydroxy-2-methyl-4-((2-nitrophenyl)selanyl)butyl)-5,8,8-trimethyl-4-((1*R*,2*S*)-2-methyl-1-((triethylsilyl)oxy)pent-4-en-1-yl)bicyclo[5.1.0]octan-3-one (SI-9): To a 0.24 M solution of LiN*i*-Pr_2 in THF (5.53 mL, 2.26 mmol, 1.5 eq) was added a solution of **SI-6** (572 mg, 1.51 mmol, 1.00 eq) in THF (6.00 mL) and stirred for 1 h at 0 °C. Aldehyde **(*S*)-5** (864 mg, 3.02 mmol, 2.00 eq) was dissolved in THF (4.00 mL) and added to the reaction mixture over the wall of the reaction flask. The reaction was stirred for 5 min and then quenched with Na_2HPO_4/KH_2PO_4 pH 7 buffer solution (5 mL). The mixture was diluted with EtOAc (15 mL). The phases were separated, and the aqueous phase was extracted with EtOAc (2 x 30 mL). The combined organic layers were dried over Na_2SO_4, filtered and concentrated under reduced pressure. Purification by column chromatography on silica gel (hexanes/EtOAc 90:10) afforded the title compound **SI-9** (946 mg, 1.42 mmol, 94%) as a yellow oil.

^{1}H NMR (400 MHz, $CDCl_3$) δ = 8.27 (dd, *J* = 8.3, 1.4 Hz, 1H), 7.58 – 7.49 (m, 2H), 7.30 (ddd, *J* = 8.4, 6.9, 1.5 Hz, 1H), 5.72 (dddd, *J* = 16.8, 10.2, 7.5, 6.2 Hz, 1H), 5.06 – 4.97 (m, 2H), 4.05 (ddd, *J* = 9.6, 3.9, 1.2 Hz, 1H), 3.89 (t, *J* = 5.0 Hz, 1H), 3.01 (td, *J* = 7.5, 2.0 Hz, 2H), 2.73 (t, *J* = 9.4 Hz, 1H), 2.58 (t, *J* = 5.3 Hz, 1H), 2.28 (dd, *J* = 3.9, 0.9 Hz, 1H), 2.09 (qd, *J* = 6.6, 2.6 Hz, 1H), 2.05 – 1.98 (m, 1H), 1.93 – 1.82 (m, 4H), 1.75 (dt, *J* = 14.8, 6.6 Hz, 1H), 1.62 (ddd, *J* = 15.0, 8.9, 2.8 Hz, 1H), 1.08 (s, 6H), 1.01 (d, *J* = 6.8 Hz, 3H), 0.98 – 0.94 (m, 12H), 0.92 – 0.90 (m, 3H), 0.70 – 0.62 (m, 7H), 0.15 (t, *J* = 9.2 Hz, 1H).

^{13}C NMR (101 MHz, $CDCl_3$) δ = 213.9, 147.0, 137.0, 134.0, 133.7, 129.3, 126.6, 125.4, 116.4, 78.3, 74.4, 63.0, 51.1, 37.6, 37.2, 33.7, 33.2, 32.6, 28.8, 28.8, 25.6, 24.5, 22.5, 20.9, 19.8, 16.3, 16.0, 13.3, 7.3, 5.7.

IR (thin film, cm^{-1}): 2957, 2935, 2876, 1685, 1591, 1567, 1516, 1455, 1379, 1332, 1304, 1248, 1120, 1096, 1068, 1038, 1002, 912, 783, 730.

HRMS (ESI): *m/z*: exact mass calculated for $[C_{34}H_{55}NNaO_5SeSi]^+$ (M+Na^+): 688.2907, found: 688.2898;

$[a]_D^{25°}$= 78.5 (c = 1.0, $CHCl_3$).

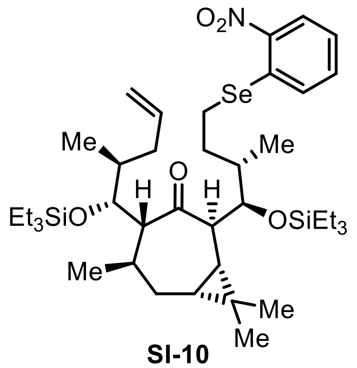

SI-10

(1*R*,2*R*,4*R*,5*R*,7*R*)-5,8,8-trimethyl-4-((1*R*,2*S*)-2-methyl-1-((triethylsilyl)oxy)pent-4-en-1-yl)-2-((1*R*,2*S*)-2-methyl-4-((2-nitrophenyl)selanyl)-1-((triethylsilyl)oxy)butyl)bicyclo[5.1.0]octan-3-one (SI-10): 2,6-lutidine (2.47 mL, 227 mg, 21.1 mmol, 15.0 eq) was dissolved in CH_2Cl_2 (10.0 mL) and the solution was cooled to –78 °C. Et_3SiOTf (3.20 mL, 374 mg, 14.1 mmol, 10.0 eq) was added. A solution of aldol addition product **SI-9** (942 mg, 1.41 mmol, 1.00 eq) in CH_2Cl_2 (5.00 mL) was slowly added along the wall of the flask. The reaction mixture was stirred for 4 h. The reaction was quenched with Na_2HPO_4/KH_2PO_4 pH 7 buffer solution (15 mL), diluted with CH_2Cl_2 (20 mL) and the phases were separated. The aqueous phase was extracted with CH_2Cl_2 (2 x 30 mL). The combined organic layers were dried over Na_2SO_4, filtered and concentrated under reduced pressure. Purification by column chromatography on silica gel (hexanes/EtOAc 95:5) afforded the title compound **SI-10** (860 mg, 1.10 mmol, 78%) as a yellow oil.

^{1}H NMR (400 MHz, $CDCl_3$) δ = 8.37 – 8.22 (m, 1H), 7.57 – 7.48 (m, 2H), 7.31 (ddd, *J* = 8.4, 6.2, 2.3 Hz, 1H), 5.75 (dddd, *J* = 16.8, 10.2, 7.9, 6.4 Hz, 1H), 5.05 – 4.95 (m, 2H), 4.06 (dd, *J* = 8.5, 1.2 Hz, 1H), 3.74 (dd, *J* = 6.5, 3.5 Hz, 1H), 3.00 (dt, *J* = 11.2, 4.0 Hz, 1H), 2.97 – 2.88 (m, 1H), 2.82 (dd, *J* = 9.7, 8.5 Hz, 1H), 2.54 (t, *J* = 3.7 Hz, 1H), 2.24 – 2.14 (m, 1H), 2.13 – 2.06 (m, 1H), 2.03 – 1.96 (m, 1H), 1.93 – 1.85 (m, 2H), 1.82 – 1.63 (m, 3H), 1.08 (s, 3H), 1.06 (s, 3H), 1.02 (d, *J* = 7.0 Hz, 3H), 0.97 (td, *J* = 8.0, 4.7 Hz, 21H), 0.91 (d, *J* = 6.8 Hz, 3H), 0.70 – 0.58 (m, 13H), 0.17 (t, *J* = 9.5 Hz, 1H).

^{13}C NMR (101 MHz, $CDCl_3$) δ = 210.8, 147.0, 136.5, 134.0, 133.7, 129.2, 126.6, 125.4, 116.5, 78.8, 77.6, 63.9, 52.2, 38.7, 36.8, 35.9, 34.1, 33.8, 29.0, 28.7, 26.9, 24.9, 22.2, 21.6, 20.3, 16.6, 16.3, 14.1, 7.4, 7.4, 5.7, 5.7.

IR (thin film, cm^{-1}): 2955, 2912, 2876, 1689, 1592, 1517, 1457, 1416, 1378, 1332, 1304, 1240, 1095, 1069, 1038, 1005, 911, 852, 784, 729.

HRMS (ESI): *m/z*: exact mass calculated for $[C_{40}H_{69}NNaO_5SeSi_2]^+$ (M+Na$^+$): 802.3772, found: 802.3754;

$[a]_D^{25°}$= 66.9 (c = 1.0, $CHCl_3$).

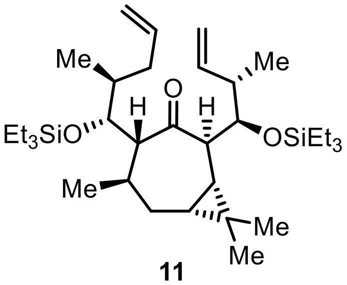

11

(1*R*,2*R*,4*R*,5*R*,7*R*)-5,8,8-trimethyl-2-((1*R*,2*S*)-2-methyl-1-((triethylsilyl)oxy)but-3-en-1-yl)-4-((1*R*,2*S*)-2-methyl-1-((triethylsilyl)oxy)pent-4-en-1-yl)bicyclo[5.1.0]octan-3-one (11): To a solution of seleno ether **SI-10** (0.700 g, 0.899 mmol, 1.00 eq) in THF–Na_2HPO_4/KH_2PO_4 pH 7 buffer (10:1, 10.0 mL) at 0 °C was added aq. H_2O_2 solution (0.200 mL, 30 w%, 1.96 mmol 2.20 eq). The cooling bath was removed, and the reaction mixture was stirred for 16 h at room temperature. Over the course of the reaction a discoloration of the yellow solution was observed. The reaction mixture was diluted with hexane (10 mL) and Na_2HPO_4/KH_2PO_4 pH 7 buffer (10 mL) and transferred to a separation funnel. The layers were separated, and the aqueous layer was extracted with hexane (2 x 15 mL). The combined organic layers were dried over Na_2SO_4 and filtered. The hexane solution was loaded directly on a column. Column chromatography on silica gel (hexanes/EtOAc 80:20) yielded the title compound **11** (467 mg, 0.809 mmol, 90%) as a colorless oil.

^{1}H NMR (400 MHz, C_6D_6) δ = 6.19 (ddd, *J* = 17.2, 10.3, 6.9 Hz, 1H), 6.00 (dddd, *J* = 16.8, 10.1, 7.7, 6.4 Hz, 1H), 5.29 – 5.17 (m, 4H), 4.44 (dd, *J* = 8.0, 2.5 Hz, 1H), 3.97 (t, *J* = 2.9 Hz, 1H), 3.00 (dd, *J* = 9.6, 8.0 Hz, 1H), 2.89 (dd, *J* = 5.0, 3.3 Hz, 1H), 2.74 (tdd, *J* = 5.5, 2.5, 1.3 Hz, 1H), 2.55 – 2.46 (m, 1H), 2.38 – 2.27 (m, 2H), 2.20 (dtd, *J* = 8.0, 5.3, 3.0 Hz, 1H), 1.80 (ddd, *J* = 13.7, 10.3, 3.1 Hz, 1H), 1.70 – 1.61 (m, 1H), 1.29 – 1.23 (m, 12H), 1.19 – 1.12 (m, 18H), 1.05 (s, 3H), 0.97 – 0.92 (m, 6H), 0.82 (d, *J* = 8.0 Hz, 6H), 0.71 (dt, *J* = 10.1, 4.9 Hz, 1H), 0.46 – 0.36 (m, 1H).

^{13}C NMR (101 MHz, C_6D_6) δ = 209.33, 144.04, 137.03, 116.49, 114.35, 79.15, 78.91, 63.58, 52.76, 40.20, 39.49, 36.91, 33.86, 29.51, 28.82, 26.88, 22.39, 20.99, 20.70, 16.52, 16.46, 13.90, 7.64, 7.53, 6.08, 5.98.

IR (thin film, cm^{-1}): 3076, 2955, 2913, 2877, 1693, 1639, 1458, 1416, 1378, 1239, 1116, 1066, 1005, 972, 911, 838, 812, 790, 738, 677.

HRMS (ESI): *m/z*: exact mass calculated for $[C_{34}H_{64}NaO_3Si_2]^+$ (M+Na$^+$): 599.4286, found: 599.4271;

$[a]_D^{25°}$= 37.0 (c = 0.5, $CHCl_3$).

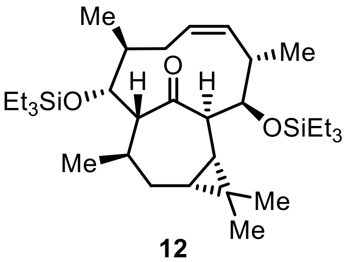

12

(1*R*,2*R*,3*S*,7*S*,8*R*,9*R*,10*R*,12*R*,14*R*,*Z*)-3,7,11,11,14-pentamethyl-2,8-bis((triethylsilyl)oxy)tricyclo[7.5.1.0^{10,12}]pentadec-5-en-15-one (12): A flame-dried and nitrogen-flushed round-bottomed flask was charged with di-olefin **11** (467 mg, 0.809 mmol,1.00 eq) in dry-degassed toluene (800 mL). Grubbs 2nd generation catalyst (137 mg, 0.161 mmol, 20.0 mol%) was added to the solution. The flask was put into a preheated oil bath and was stirred at 100 °C for 2 h. The heating bath was removed and after the reaction mixture cooled down to room temperature it was filtered through a plug of silica gel. The silica plug was washed with CH_2Cl_2 (3 x 50 mL). The filtrates were combined and concentrated under reduced pressure. Purification by column chromatography on silica gel (hexanes/EtOAc 90:10 to 80:20) yielded title compound **12** (246 mg, 0.448 mmol, 55%) as a crystalline solid. Single crystals suitable for X-Ray analysis were obtained by slow evaporation from CH_2Cl_2.

^{1}H NMR (400 MHz, $CDCl_3$) δ = 5.49 (td, *J* = 12.3, 11.7, 3.3 Hz, 1H), 4.84 (td, *J* = 10.8, 2.3 Hz, 1H), 3.82 (t, *J* = 2.9 Hz, 1H), 3.28 (dd, *J* = 10.1, 3.0 Hz, 1H), 2.90 (td, *J* = 10.5, 5.8 Hz, 1H), 2.80 (dd, *J* = 10.7, 3.0 Hz, 1H), 2.48 – 2.35 (m, 2H), 2.07 – 1.95 (m, 3H), 1.83 – 1.70 (m, 2H), 1.13 (s, 3H), 1.02 (d, *J* = 3.7 Hz, 6H), 1.00 – 0.94 (m, 24H), 0.78 (dd, *J* = 10.7, 9.4 Hz, 1H), 0.70 – 0.64 (m, 13H).

^{13}C NMR (101 MHz, $CDCl_3$) δ = 212.0, 131.5, 129.0, 85.1, 79.8, 62.0, 51.4, 41.1, 38.6, 36.2, 32.5, 29.2, 26.8, 23.2, 22.9, 21.8, 20.0, 19.9, 19.9, 15.4, 7.3, 7.3, 6.0, 5.5.

IR (thin film, cm^{-1}): 2956, 2925, 2876.3, 1692, 1459, 1416, 1379, 1339, 1292, 1264, 1237, 1203, 1163, 1133, 1105, 1088, 1063, 1038, 1007, 982, 924, 888, 873, 841, 821, 802, 771, 735, 677, 643.

HRMS (ESI): *m/z*: exact mass calculated for $C_{32}H_{60}O_3NaSi_2$ [M+Na]$^+$, 571.3973; found 571.3967.

$[a]_D^{25°}$= 73.7 (c = 1.0, $CHCl_3$).

X-Ray crystallographic data: see page 83

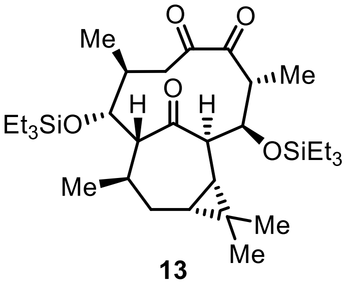

13

According to modified literature procedure.[7]

(1*R*,2*R*,3*S*,7*R*,8*R*,9*R*,10*R*,12*R*,14*R*)-3,7,11,11,14-pentamethyl-2,8-bis((triethylsilyl)oxy)tricyclo[7.5.1.010,12]pentadecane-5,6,15-trione (13): A 250-mL round-bottomed flask was charged with a 0.10 M solution of $NaHCO_3$ (6.2 mL, 0.62 mmol, 3.0 eq) in water and a 0.10 M solution of $RuCl_3$•H_2O (1.3 mL, 0.13 mmol, 0.60 eq) in water. To this solution $NaBrO_3$ (1.69g, 11.2 mmol, 54.0 eq) was added and the mixture was stirred for 5 min. Subsequently EtOAc (33.3 mL) and MeCN (43.3 mL) were added and the mixture was stirred for 5 min. After this ten-membered ring **12** (0.114 g, 0.208 mmol, 1.00 eq) in EtOAc (10 mL) was added and the reaction mixture was stirred for 3.5 h at room temperature. The reaction was quenched by the addition of sat. aq. $NaHCO_3$ (45 mL) and sat. aq. $Na_2S_2O_3$ (15 mL) solution. The mixture was transferred to a separation funnel diluted with CH_2Cl_2 (150 mL) and H_2O (100 mL). The layers were separated, and the aqueous layer was extracted with CH_2Cl_2 (2 x 100 mL). The combined organic layers were dried over Na_2SO_4, filtered, and concentrated under reduced pressure. Triketone **13** rapidly underwent aldol reaction to **SI-11** when purification on silica gel was attempted. Therefore, it was used directly in the next step without further purification.

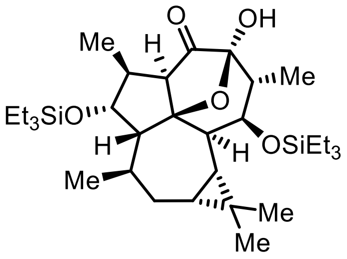

SI-11

Analytical data of hemiketal

(1*S*,2*R*,2a*R*,2a[1]*R*,3*R*,4a*R*,5a*R*,5b*R*,6*S*,7R,8*S*,9a*R*)-8-hydroxy-1,3,5,5,7-pentamethyl-2,6-bis((triethylsilyl)oxy)dodecahydro-2a[1],8-epoxycyclopenta[*ef*]cyclopropa[*a*]heptalen-9(*2H*)-one (SI-11).

^{1}H NMR (600 MHz, $CDCl_3$) δ = 4.09 (dt, *J* = 3.4, 0.9 Hz, 1H), 3.69 (dd, *J* = 10.4, 5.0 Hz, 1H), 3.26 (s, 1H), 3.02 – 2.97 (m, 1H), 2.33 (dqd, *J* = 8.5, 7.5, 1.0 Hz, 1H), 2.22 (dq, *J* = 10.4, 6.8 Hz, 1H), 1.98 (dd, *J* = 10.1, 3.4 Hz, 1H), 1.95 – 1.89 (m, 1H), 1.89 – 1.83 (m, 1H), 1.82 – 1.74 (m, 1H), 1.69 (dtd, *J* = 12.7, 6.3, 3.1 Hz, 1H), 1.04 (d, *J* = 4.9 Hz, 6H), 0.95 (dt, *J* = 11.3, 7.9 Hz, 21H), 0.89 (d, *J* = 6.2 Hz, 3H), 0.85 (d, *J* = 7.5 Hz, 3H), 0.83 – 0.80 (m, 1H), 0.72 (t, *J* = 2.2 Hz, 1H), 0.66 – 0.55 (m, 12H).

^{13}C NMR (151 MHz, $CDCl_3$) δ = 212.9, 104.4, 89.3, 83.4, 74.3, 59.2, 56.1, 43.4, 41.7, 41.1, 33.5, 30.3, 28.6, 26.0, 22.6, 22.0, 17.5, 15.8, 14.2, 11.2, 7.2, 7.1, 5.4, 5.3.

IR (thin film, cm^{-1}): 3403, 2954, 2921, 2876, 1759, 1457, 1414, 1375, 1277, 1238, 1208, 1171, 1090, 1059, 1004, 973, 938, 867, 842, 788, 726, 636.

HRMS (ESI): *m/z*: exact mass calculated for $C_{32}H_{58}O_5NaSi_2$ $[M+Na]^+$, 601.3715; found 601.3706.

$[a]_D^{25°}$= 44.3 (c = 1.0, $CHCl_3$).

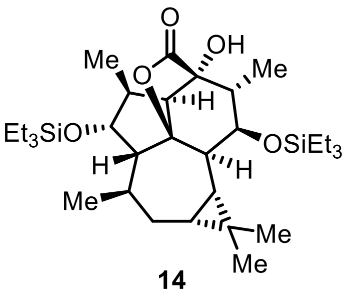

14

(1*S*,2*R*,2a*R*,2a¹*R*,3*R*,4a*R*,5a*R*,5b*R*,6*R*,7*R*,8*R*,8a*S*)-8-hydroxy-1,3,5,5,7-pentamethyl-2,6-bis((triethylsilyl)oxy)dodecahydro-1H-2a¹,8-(epoxymethano)benzo[*cd*]cyclopropa[*f*]azulen-9-one (14): In a 10-mL point-bottomed flask was added unpurified triketone **13** from the previous step as a solution in THF (4.00 mL) and subsequently cooled to 0 °C. Next, a 1M solution of $LiN(SiMe_3)_2$ in THF (1.1 mL, 1.1 mmol, 5.1 eq) was added and the mixture was stirred at 0 °C for 1 h and then warmed to room temperature. After 3 h the reaction was quenched by the addition of sat. aq. NH_4Cl (3 mL). The mixture was transferred to a separation funnel diluted with EtOAc (5 mL) and H_2O (2 mL). The layers were separated, and the aqueous layer was extracted with EtOAc (2 x 5 mL). The combined organic layers were dried over Na_2SO_4, filtered, and concentrated under reduced pressure. Purification by column chromatography on silica gel (hexanes/EtOAc 80:20 to 70:30) yielded the title compound **14** (55.0 mg, 0.0950 mmol, 46% over two steps) as a crystalline solid. Single crystals suitable for X-Ray analysis were obtained by slow evaporation from CH_2Cl_2.

^{1}H NMR (600 MHz, $CDCl_3$) δ = 3.99 (dd, *J* = 4.9, 3.4 Hz, 1H), 3.67 (dd, *J* = 4.6, 1.1 Hz, 1H), 2.77 (d, *J* = 8.5 Hz, 1H), 2.39 (d, *J* = 0.9 Hz, 1H), 2.33 (t, *J* = 4.6 Hz, 1H), 2.19 (ddd, *J* = 8.5, 7.5, 3.6 Hz, 1H), 2.07 (tt, *J* = 8.0, 3.6 Hz, 1H), 1.99 (qd, *J* = 7.2, 1.1 Hz, 1H), 1.70 (dd, *J* = 11.0, 4.6 Hz, 1H), 1.63 (dt, *J* = 14.5, 4.8 Hz, 1H), 1.49 – 1.43 (m, 1H), 1.20 (d, *J* = 7.1 Hz, 4H), 1.15 – 1.09 (m, 4H), 1.07 (s, 3H), 1.01 – 0.98 (m, 6H), 0.95 (q, *J* = 8.1 Hz, 18H), 0.79 (ddd, *J* = 11.9, 9.2, 5.1 Hz, 1H), 0.62 – 0.55 (m, 12H).

^{13}C NMR (151 MHz, $CDCl_3$) δ = 179.70, 99.83, 85.17, 78.20, 75.63, 55.46, 52.18, 49.39, 41.64, 41.48, 29.86, 29.00, 28.68, 23.31, 21.66, 21.37, 18.20, 14.72, 13.68, 13.02, 7.09, 7.07, 5.16, 5.03.

IR (thin film, cm^{-1}): 2955, 2877, 1771, 1458, 1414, 1377, 1238, 1152, 1097, 1076, 1057, 1005, 975, 958, 867, 849, 802, 776, 725, 530.

HRMS (ESI): *m/z*: exact mass calculated for $C_{32}H_{58}O_5NaSi_2$ $[M+Na]^+$, 601.3715; found 601.3704.

$[a]_D^{25°}$= –2.5 (c = 1.0, $CHCl_3$).

X-Ray crystallographic data: see page 92

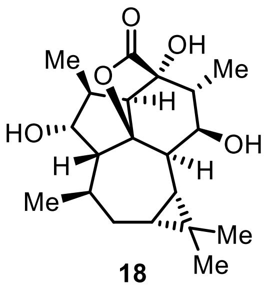

18

(1*S*,2*R*,2a*R*,2a^1*R*,3*R*,4a*R*,5a*R*,5b*R*,6*R*,7*R*,8*R*,8a*S*)-2,6,8-trihydroxy-1,3,5,5,7-pentamethyldodecahydro-1H-2a^1,8-(epoxymethano)benzo[*cd*]cyclopropa[*f*]azulen-9-one (18): A 5-mL point-bottomed flask was charged with lactone **14** (55.0 mg, 0.0950 mmol, 1.00 eq) and dry THF (1.00 mL). The mixture was cooled to 0 °C and a 1.0 M *n*-Bu_4NF solution (0.31 mL, 0.31 mmol, 3.2 eq) in THF was added dropwise. The cooling bath was removed, and the reaction mixture was stirred for 17 h at room temperature. The reaction was quenched by the addition of sat. aq. $NaHCO_3$ solution (1 mL). The mixture was transferred to a separation funnel diluted with EtOAc (5 mL) and H_2O (5 mL). The layers were separated, and the aqueous layer was extracted with EtOAc (2 x 5 mL). The combined organic layers were dried over Na_2SO_4, filtered, and concentrated under reduced pressure. Purification by column chromatography on silica gel (hexanes/EtOAc 40:60) yielded title compoundl **18** (33.0 mg, 0.0941 mmol, 99%) as a crystalline solid.

^{1}H NMR (500 MHz, $CDCl_3$) δ = 4.13 (t, *J* = 5.5 Hz, 1H), 3.74 (d, *J* = 5.5 Hz, 1H), 2.74 (d, *J* = 9.4 Hz, 2H), 2.37 (dd, *J* = 7.9, 5.8 Hz, 1H), 2.29 (dqd, *J* = 9.5, 7.5, 5.2 Hz, 1H), 2.12 (qd, *J* = 7.2, 2.1 Hz, 1H), 2.05 – 1.98 (m, 2H), 1.92 – 1.87 (m, 1H), 1.81 (s, 1H), 1.71 – 1.64 (m, 1H), 1.52 (ddd, *J* = 14.9, 9.7, 5.2 Hz, 1H), 1.21 (d, *J* = 6.7 Hz, 3H), 1.16 (dd, *J* = 7.2, 0.9 Hz, 3H), 1.09 (d, *J* = 7.6 Hz, 3H), 1.08 (s, 3H), 1.02 (d, *J* = 0.7 Hz, 3H), 0.93 – 0.86 (m, 2H).

^{13}C NMR (126 MHz, $CDCl_3$) = δ 179.9, 100.4, 83.0, 78.4, 74.7, 53.8, 50.2, 47.7, 41.6, 39.8, 29.9, 28.9, 28.8, 23.5, 21.4, 21.2, 18.7, 14.8, 14.3, 13.3.

IR (thin film, cm^{-1}): 3435, 2956, 2924, 1760, 1636, 1460, 1377, 1306, 1258, 1186, 1153, 1122, 1094, 1062, 1027, 947, 879, 840, 800, 759, 706, 668.

HRMS (ESI): *m/z*: exact mass calculated for $C_{20}H_{30}NaO_5^+$ $[M+Na]^+$, 373.1985; found 373.1984.

$[a]_D^{25°}$= -1.1 (c = 0.6, $CHCl_3$)

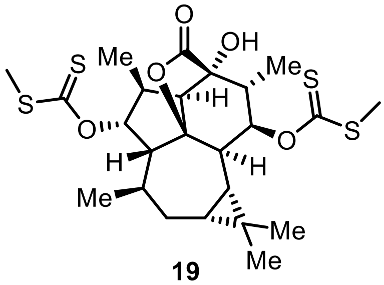

19

O,O'-((1*S*,2*R*,2a*R*,2a^1*R*,3*R*,4a*R*,5a*R*,5b*R*,6*R*,7*R*,8*R*,8a*S*)-8-hydroxy-1,3,5,5,7-pentamethyl-9-oxododecahydro-1H-2a^1,8-(epoxymethano)benzo[*cd*]cyclopropa[*f*]azulene-2,6-diyl) *S*,*S*'-dimethyl bis(carbonodithioate) (19): A 5-mL point-bottomed flask charged with triol **18** (8.00 mg, 22.8 μmol, 1.00 eq) and dry DMF (1.20 mL). DBU (80.0 μL, 0.536 mmol, 23.5 eq) was added and the mixture was stirred for 2 min at room temperature. Subsequently, CS_2 (80.0 μL, 1.32 mmol, 56.0 eq) was added, and the mixture was stirred for 3.5 h. Then MeI (80.0 μL, 1.85 mmol, 56.0 eq) was added and the mixture was stirred for an additional 30 min. The reaction mixture was diluted with CH_2Cl_2 (1 mL) and quenched by the addition of water (1 mL). The mixture was transferred to a separation funnel and the layers were separated. The organic layer was washed with sat. aq. NaCl solution (1 mL), dried over Na_2SO_4, filtrated, and concentrated under reduced pressure. Purification by column chromatography on silica gel (hexanes/EtOAc 90:10 to 80:20) yielded title compound **19** (11.0 mg, 0.0207 mmol, 91%) as a wight solid.

^{1}H NMR (500 MHz, $CDCl_3$) δ = 5.90 – 5.84 (m, 2H), 2.78 – 2.72 (m, 2H), 2.69 (dddd, *J* = 11.1, 7.4, 4.1, 1.0 Hz, 1H), 2.58 (s, 3H), 2.55 (s, 3H), 2.54 – 2.51 (m, 1H), 2.21 (qd, *J* = 7.3, 1.9 Hz, 1H), 2.16 (dd, *J* = 10.9, 5.8 Hz, 1H), 2.01 (tt, *J* = 6.8, 5.0 Hz, 1H), 1.73 (dt, *J* = 14.9, 5.5 Hz, 1H), 1.60 – 1.51 (m, 2H), 1.25 (d, *J* = 7.3 Hz, 3H), 1.12 (dd, *J* = 7.1, 4.3 Hz, 6H), 1.03 (s, 3H), 0.98 (s, 3H), 0.87 – 0.82 (m, 1H), 0.75 (dd, *J* = 10.9, 9.2 Hz, 1H).

^{13}C NMR (126 MHz, $CDCl_3$) = δ 215.5, 214.5, 178.7, 99.0, 94.1, 83.6, 78.1, 53.5, 51.2, 45.0, 40.1, 38.8, 29.6, 29.5, 28.6, 22.9, 21.5, 21.4, 19.4, 18.9, 18.4, 14.6, 13.3, 12.8.

IR (thin film, cm^{-1}): 3437, 2956, 2923, 2854, 1770, 1665, 1609, 1458, 1423, 1378, 1260, 1199, 1165, 1129, 1055, 961, 886, 801, 757, 709, 666, 636.

HRMS (ESI): *m/z*: exact mass calculated for $C_{24}H_{34}NaO_5S_4$ $[M+Na]^+$, 553.1181; found 553.1179

[a]$_D^{28°}$= +53.4 (c = 1.0, $CHCl_3$)

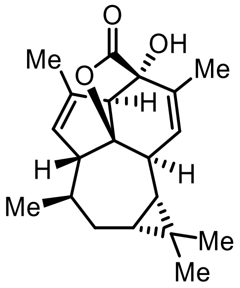

(+)-Euphorikanin A

(+)-Euphorikanin A (1):

Passivation of the glass surface: A flame-dried and nitrogen-flushed 10 mL J Young *Schlenk* tube was charged with a mixture of N,O-Bis(trimethylsilyl) acetamide (0.0100 mL) and *n*-hexane (0.400 mL) and heated to 60 °C. The flask was sealed and shaken, so the entire glass surface was in contact with the reagent. After cooling, the solution was discarded, the flask was rinsed with dry hexane (2 x 0.200 mL) and CH_2Cl_2 (0.200 mL) and dried under reduced pressure.

Chugaev elimination: A solution of bis-methylxanthate **19** (3.00 mg, 5.65 μmol, 1.00 eq) in CH_2Cl_2 (0.20 mL) was transferred into the dried and passivated (as described above) J Young *Schlenk* tube (15 cm length, 1 cm outer diameter, 1.3 mm glass thickness) and the solvent was evaporated under a constant flow of nitrogen, resulting in localization of the starting material at one end of the Schlenk tube. The residue was subjected to reduced pressure for 10 min, after which the flask was flushed with nitrogen again. This process was repeated 3 times. The *Schlenk* flask was tilted by 90 ° and at a distance 12 cm away along the Schlenk tube cooling was applied with dry ice (–78 °C). The Schlenk tube was evacuated to 0.36 mbar, and the end of the Schlenk tube, containing the bis-methylxanthate, was heated for 5 sec. with two Bunsen burners, as shown below. During the process the residue was observed to slowly disappear. After disappearance of the residue, the heating region was moved along the uncooled length of the Schlenk tube over 8 sec, whereupon heating was stopped. Condensation of a pale-yellow oil was observed in the cooled part of the *Schlenk* tube. After the *Schlenk* tube reached room temperature, the residue was transferred into a 5 mL flask using CH_2Cl_2 (3 x 0.5 mL) and concentrated under reduced pressure. Purification by column chromatography on silica gel (hexanes/acetone 90:10 to 80:20) yielded **(+)-Euphorikanin A (1)** (1.2 mg, 0.0038 mmol, 68%) as a white solid.

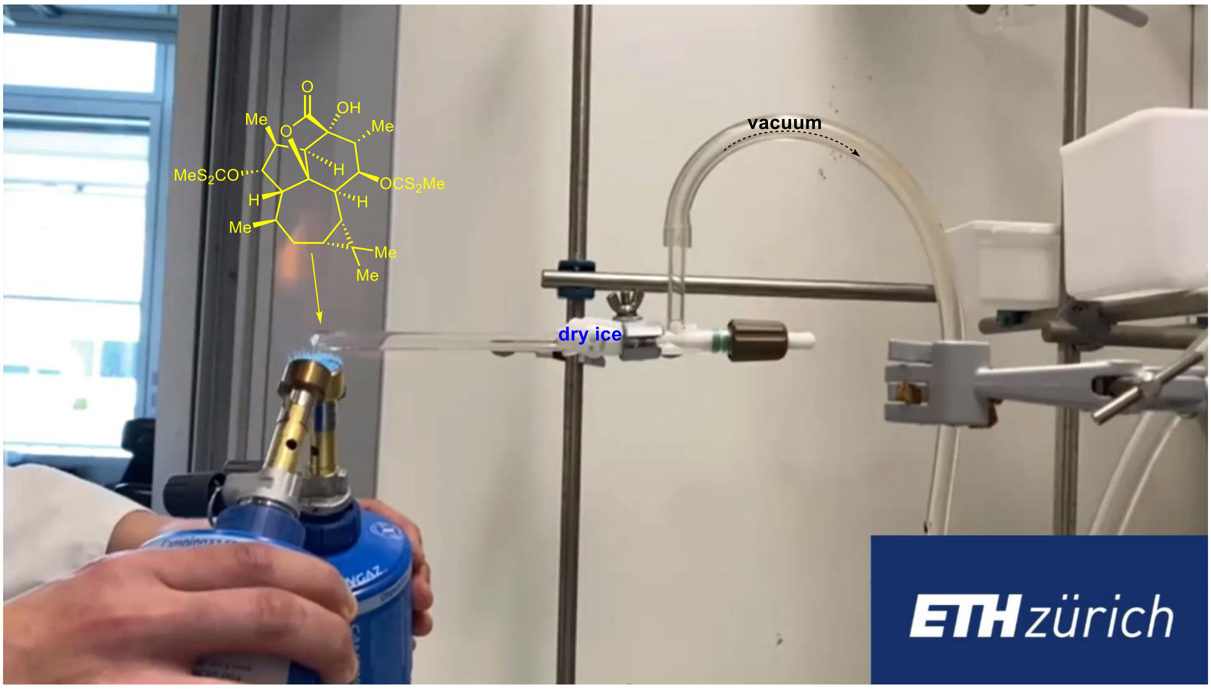

^{1}H NMR (600 MHz, $CDCl_3$) δ = 5.53 (td, *J* = 2.4, 1.5 Hz, 1H), 5.39 (dq, *J* = 3.2, 1.6 Hz, 1H), 3.09 (dq, *J* = 2.7, 1.3 Hz, 1H), 2.74 (s, 1H), 2.71 (ddd, *J* = 10.9, 4.4, 1.7 Hz, 1H), 2.34 (dp, *J* = 11.4, 2.4 Hz, 1H), 1.84 (q, *J* = 1.6 Hz, 3H), 1.81 (dd, *J* = 2.3, 1.6 Hz, 3H), 1.79 (dd, *J* = 6.2, 3.6 Hz, 1H), 1.69 (dt, *J* = 15.5, 10.1 Hz, 1H), 1.35 – 1.32 (m, 1H), 1.05 (s, 3H), 1.00 (s, 3H), 0.88 (d, *J* = 6.5 Hz, 3H), 0.81 (dd, *J* = 11.5, 9.2 Hz, 1H), 0.69 (td, *J* = 9.5, 3.7 Hz, 1H).

^{13}C NMR (151 MHz, $CDCl_3$) δ = 177.0, 139.5, 137.3, 130.6, 126.1, 97.3, 80.4, 59.7, 54.6, 39.1, 32.7, 31.3, 28.6, 26.6, 20.9, 18.8, 16.6, 16.3, 15.3.

IR (thin film, cm^{-1}): 3437, 2923, 2853, 1757, 1462, 1377, 1183, 1158, 959, 883, 839.

HRMS (ESI): *m/z*: exact mass calculated for $C_{20}H_{26}NaO_3$ $[M+Na]^+$, 337.1774; found 337.1777.

$[a]_D^{27°}$= +58.1 (c = 0.1, $CHCl_3$)

NMR Spectra

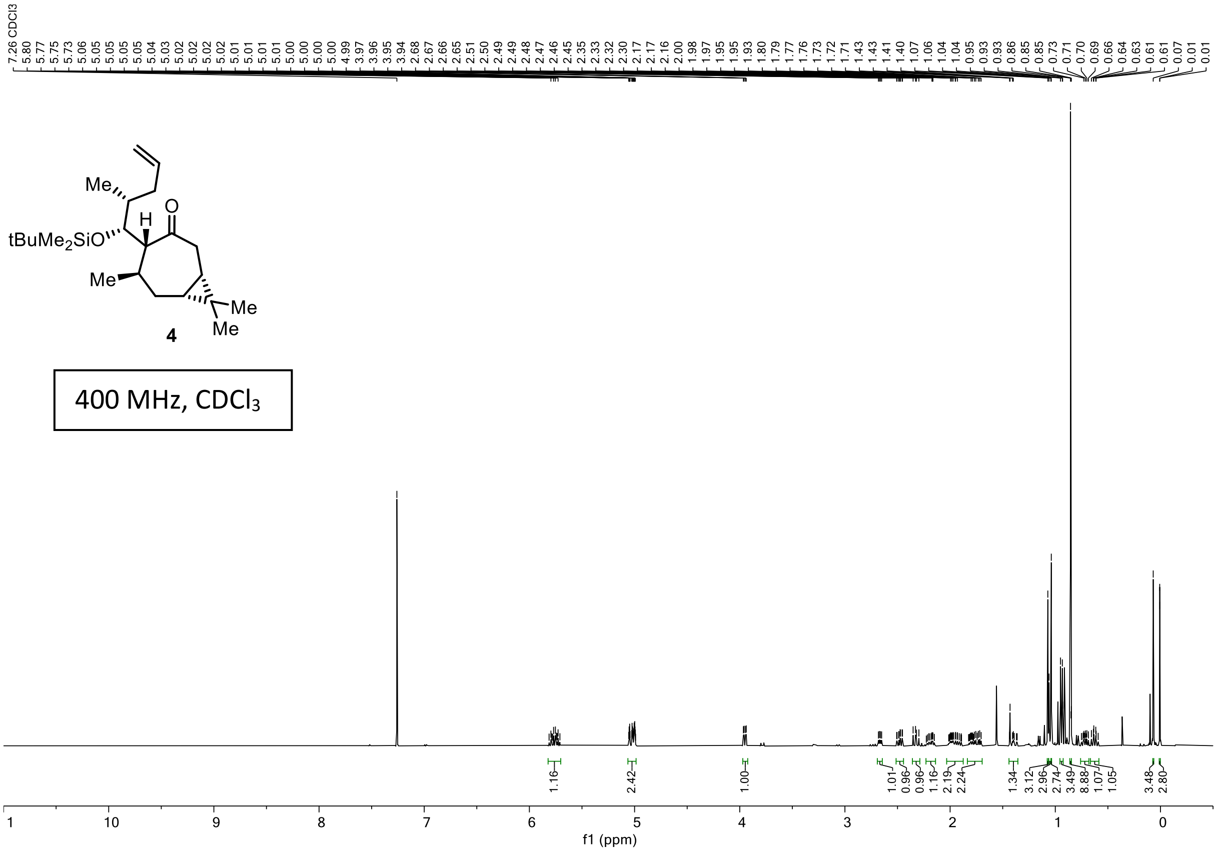
Me
H
O
tBuMe2SiO
Me
Me
Me
4
400 MHz, CDCl3
f1 (ppm)

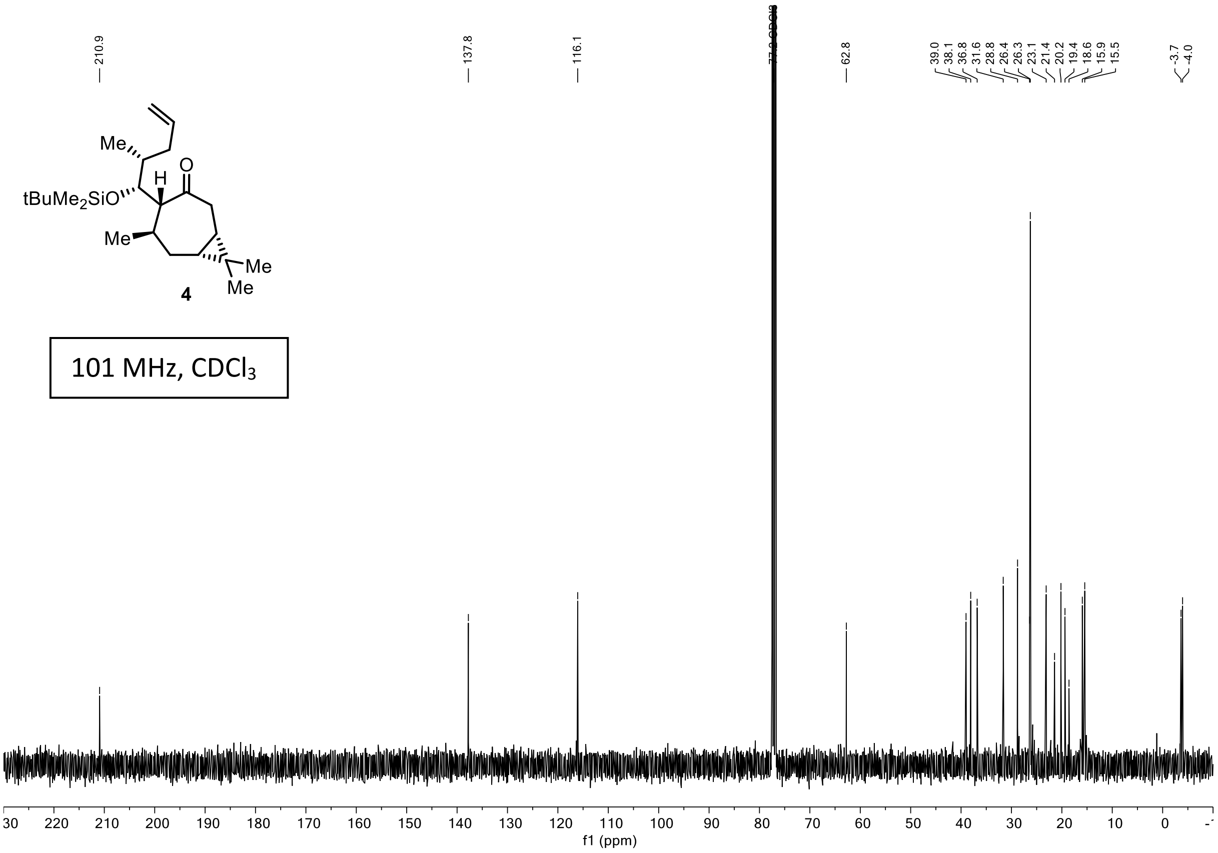
Me
H
O
tBuMe2SiO
Me
Me
Me
4
101 MHz, CDCl3
f1 (ppm)

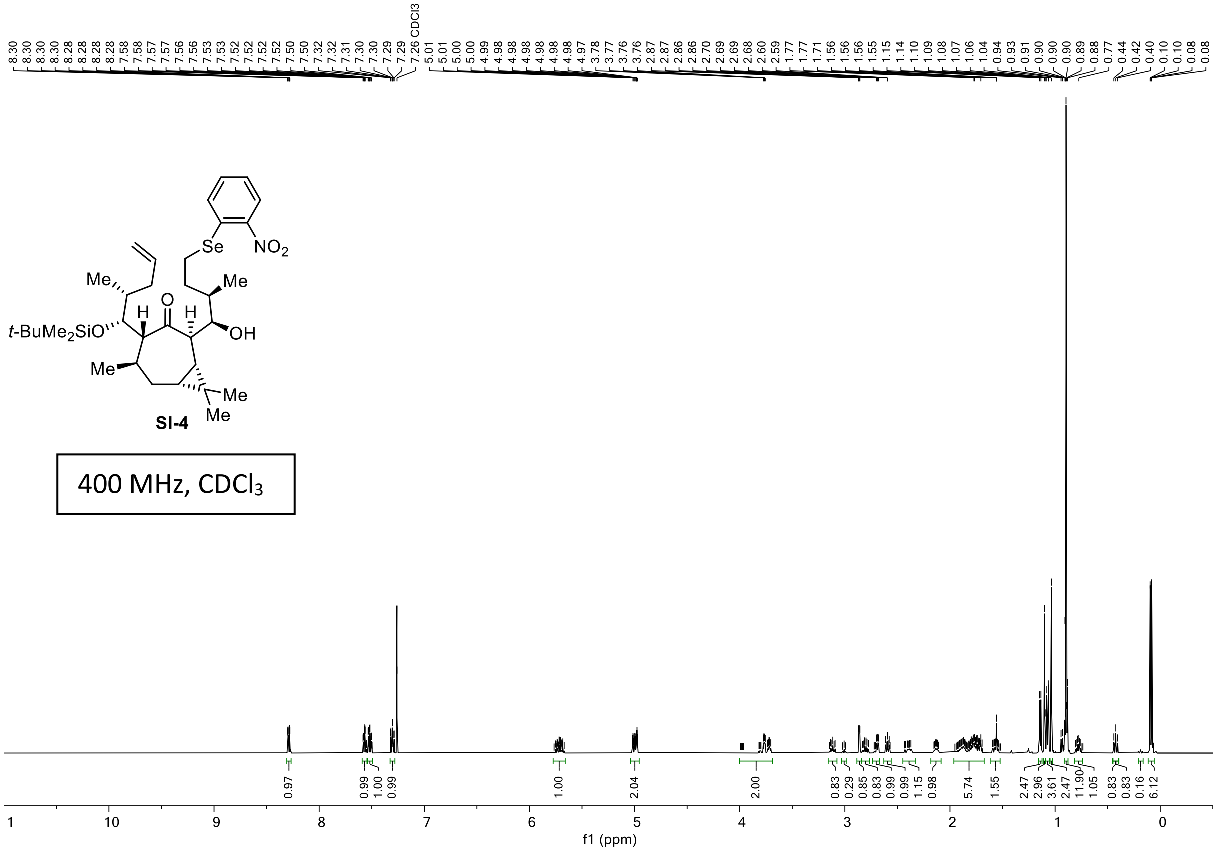
SI-4
400 MHz, $CDCl_3$
f1 (ppm)

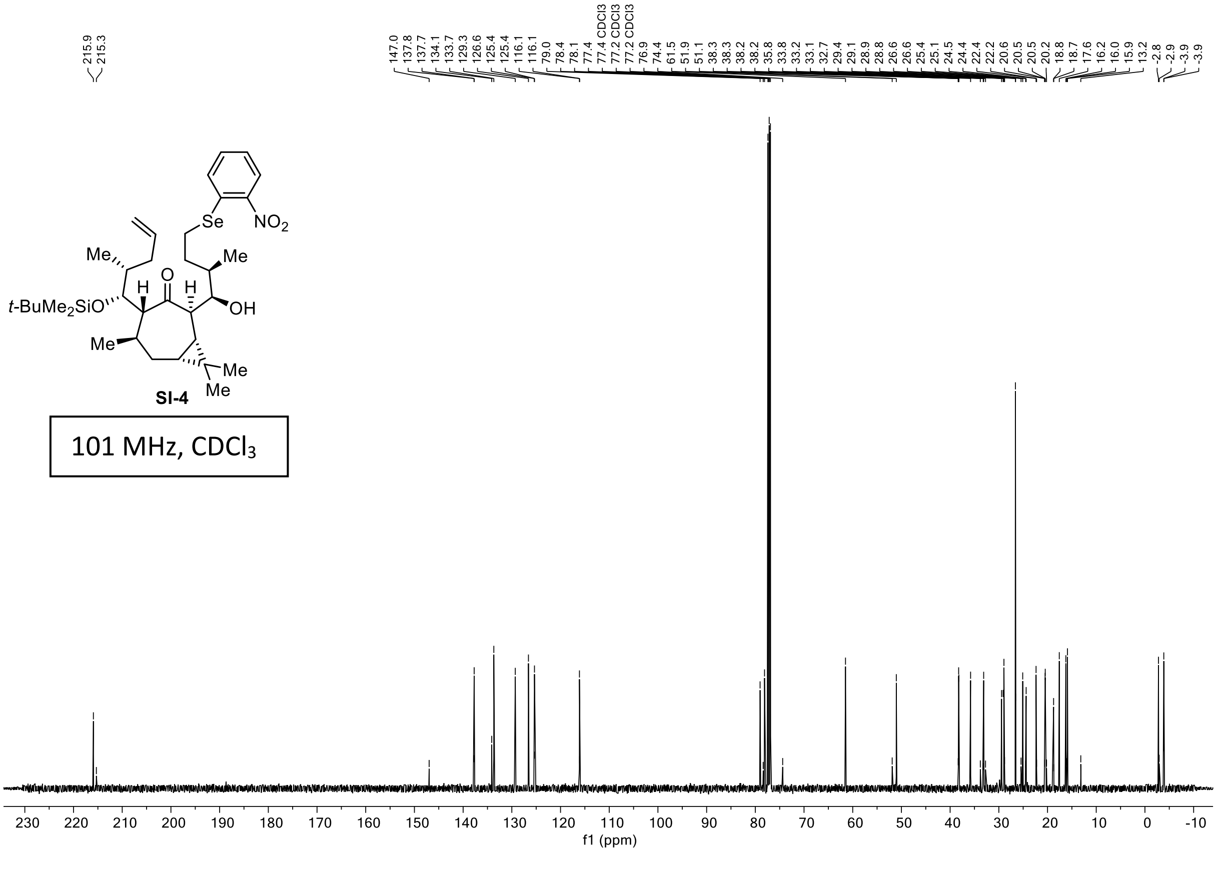
SI-4
101 MHz, $CDCl_3$
f1 (ppm)

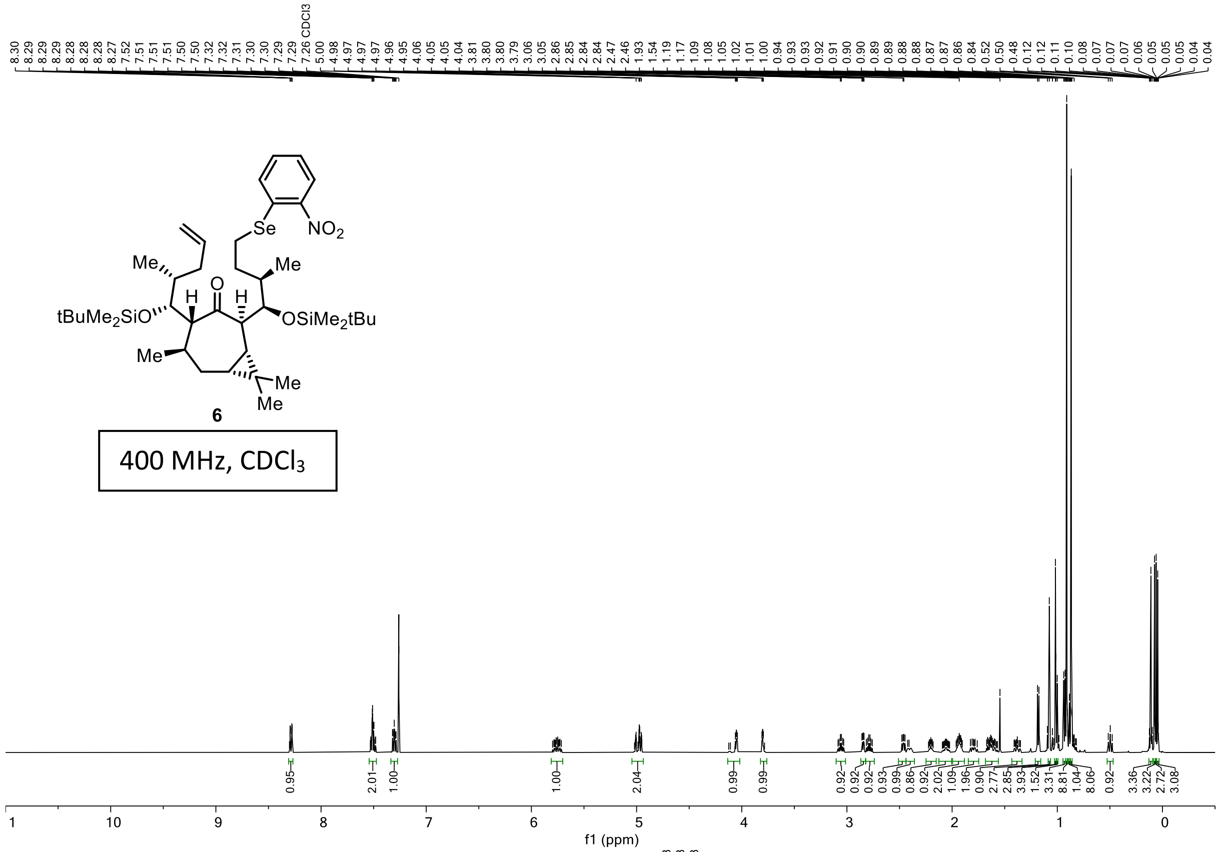
6
400 MHz, $CDCl_3$
f1 (ppm)

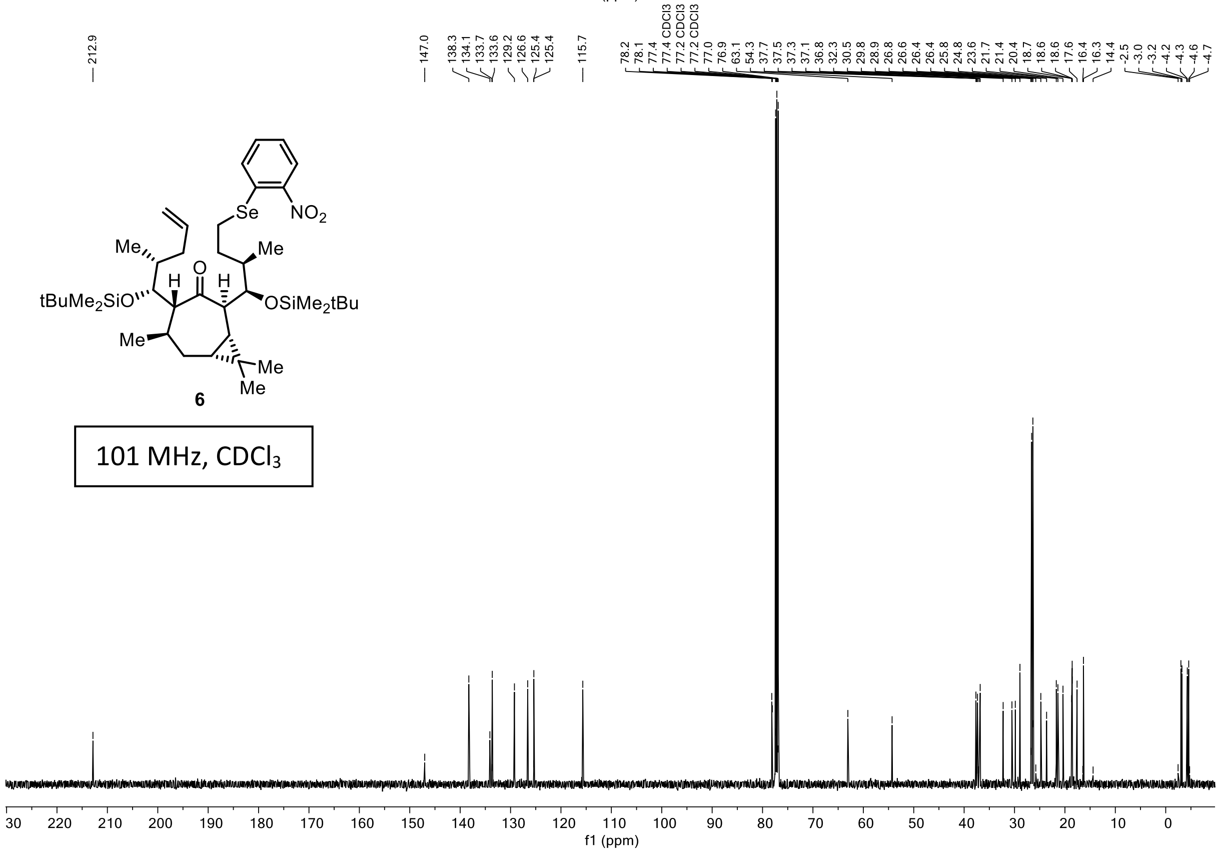
6
101 MHz, $CDCl_3$
f1 (ppm)

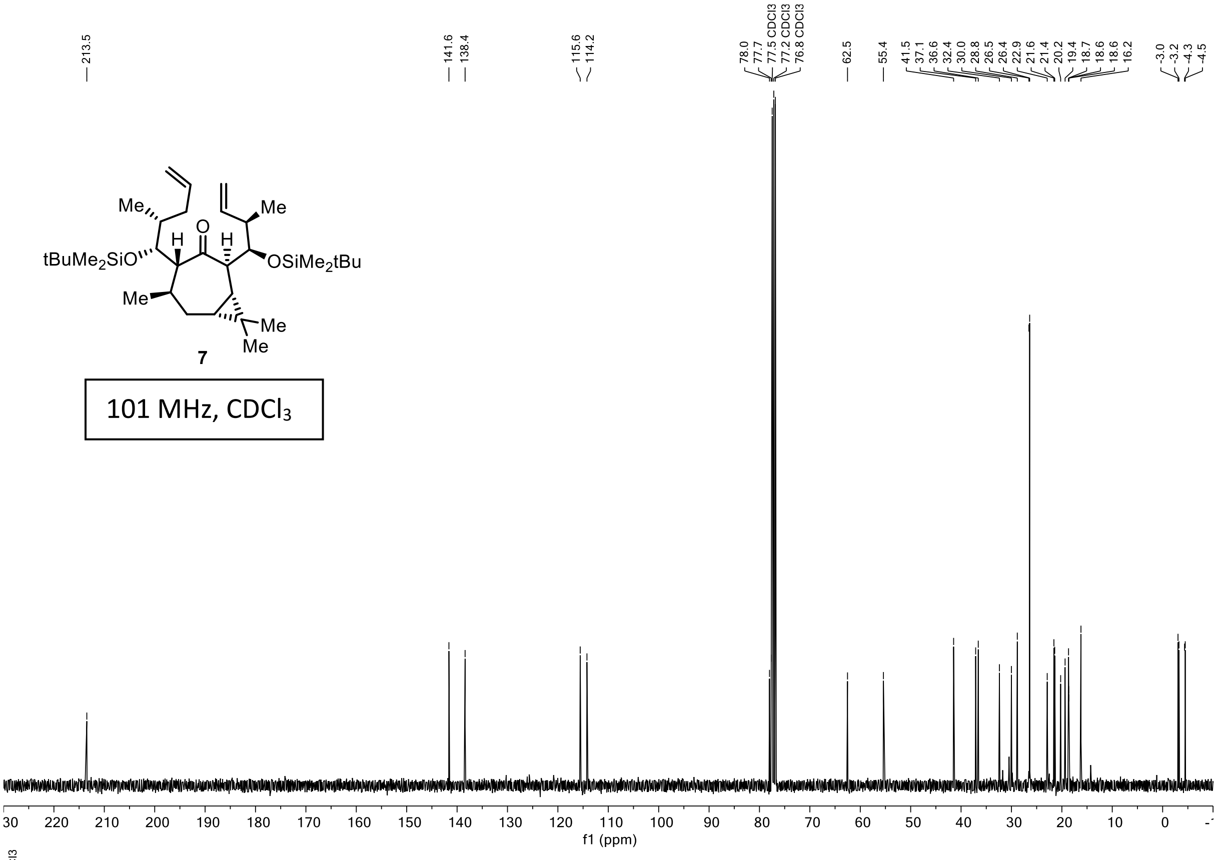
7
101 MHz, CDCl3
f1 (ppm)

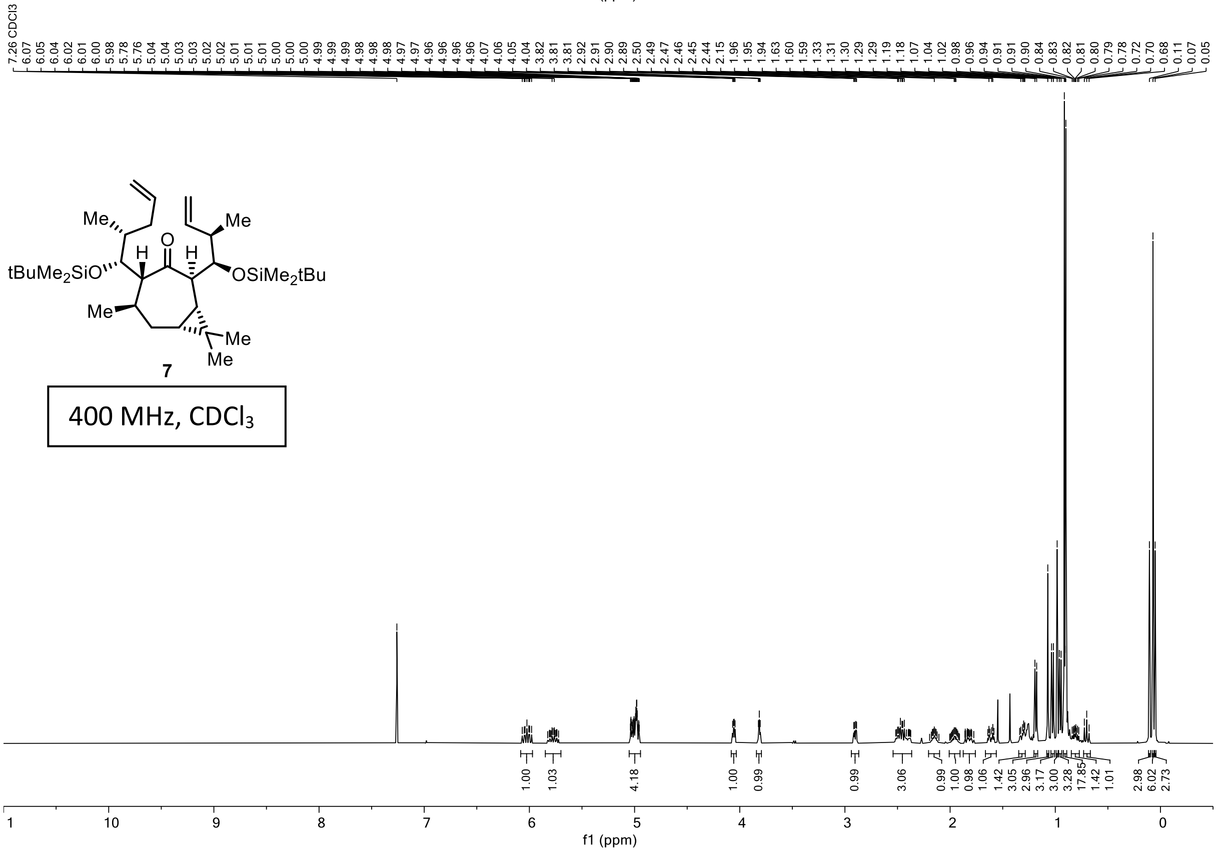
7
400 MHz, CDCl3
f1 (ppm)

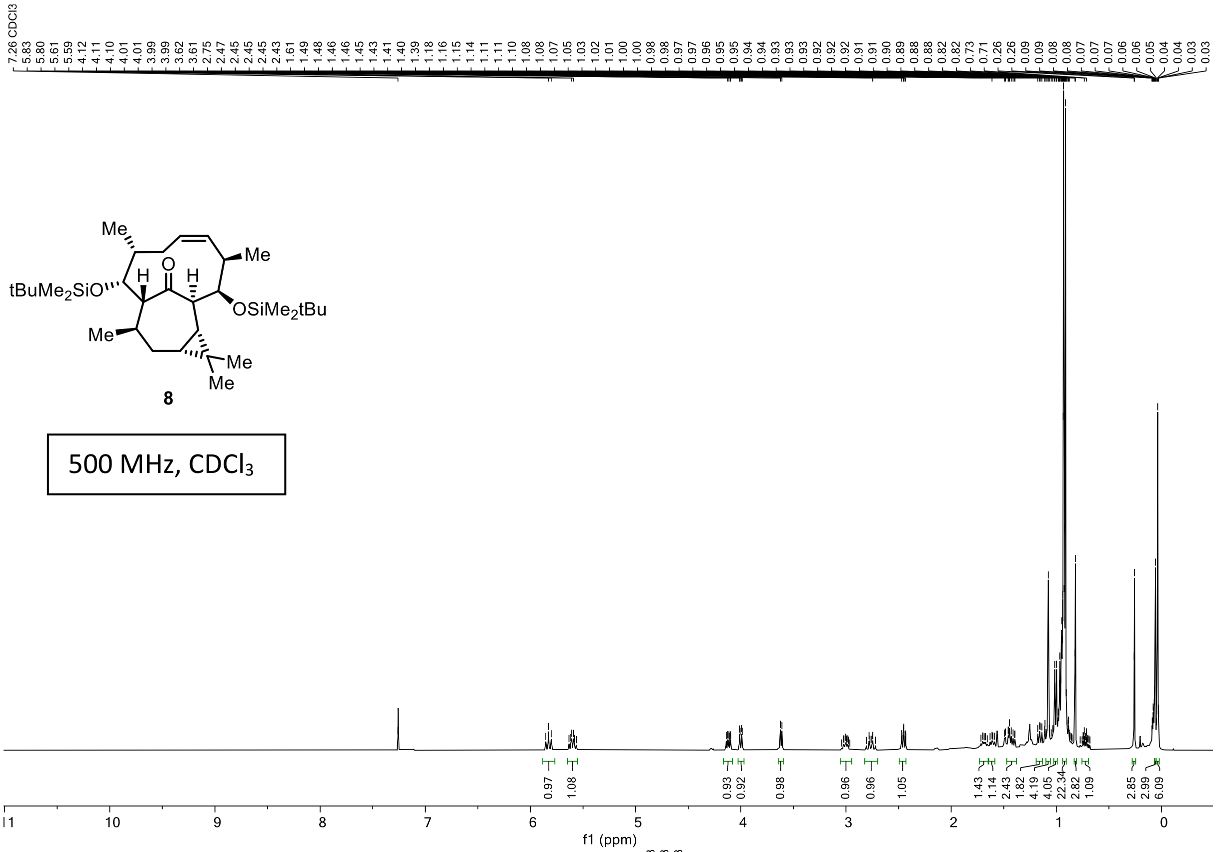
7.26 CDCl3
5.83
5.80
5.61
5.59
4.12
4.11
4.10
4.01
4.01
3.99
3.99
3.62
3.61
2.75
2.47
2.45
2.45
2.43
1.61
1.49
1.48
1.46
1.45
1.43
1.41
1.40
1.39
1.18
1.16
1.15
1.14
1.11
1.11
1.10
1.08
1.08
1.07
1.05
1.03
1.02
1.01
1.00
1.00
0.98
0.98
0.97
0.97
0.97
0.96
0.95
0.95
0.94
0.94
0.93
0.93
0.93
0.92
0.92
0.92
0.91
0.91
0.90
0.89
0.88
0.88
0.88
0.82
0.82
0.73
0.71
0.26
0.26
0.09
0.09
0.08
0.08
0.08
0.07
0.07
0.07
0.06
0.06
0.05
0.04
0.04
0.03
0.03
Me
Me
tBuMe2SiO
H
O
H
OSiMe2tBu
Me
Me
Me
8
500 MHz, CDCl3
0.97
1.08
0.93
0.92
0.98
0.96
0.96
1.05
1.43
1.14
2.43
1.82
4.19
4.05
22.34
2.82
1.09
2.85
2.99
6.09
11
10
9
8
7
6
5
4
3
2
1
0
f1 (ppm)

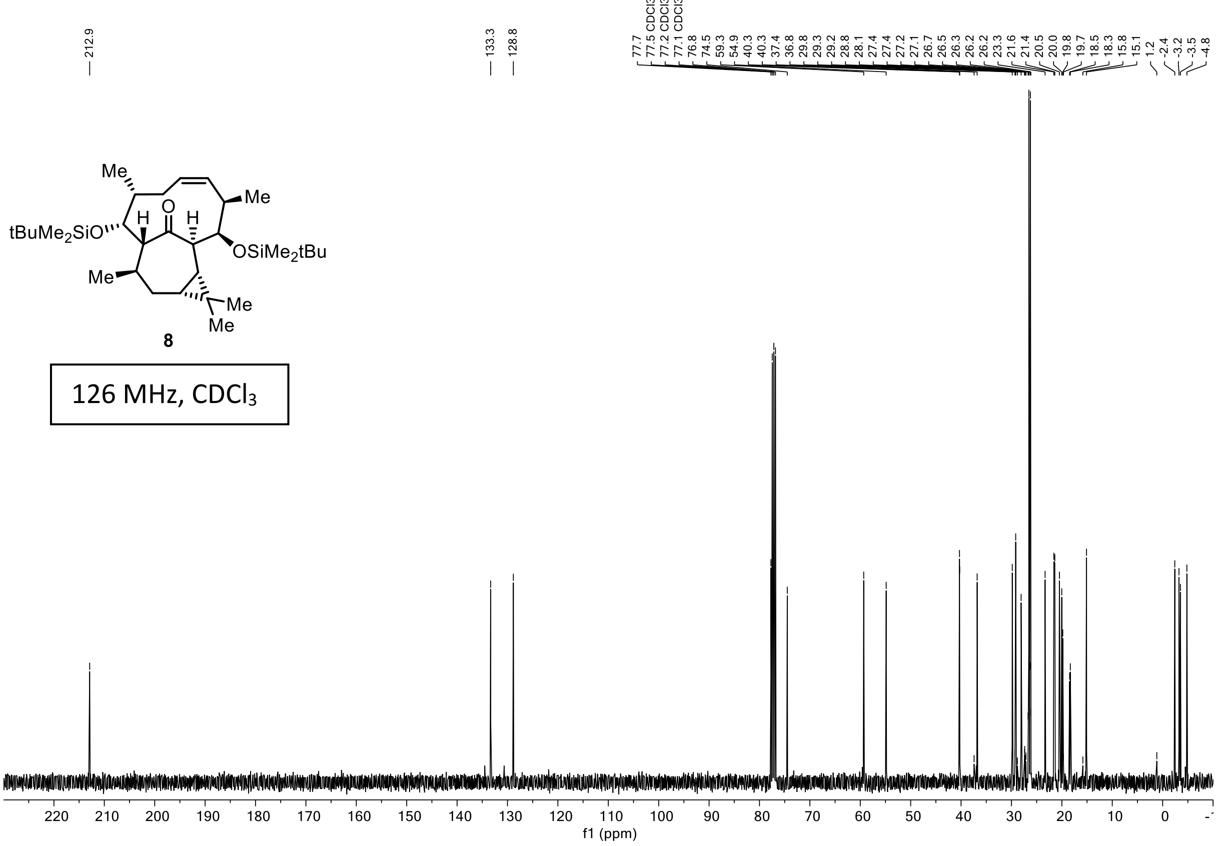
212.9
133.3
128.8
77.7
77.5 CDCl3
77.2 CDCl3
77.1 CDCl3
76.8
74.5
59.3
54.9
40.3
40.3
37.4
36.8
29.8
29.3
29.2
28.8
28.1
27.4
27.4
27.2
27.1
26.7
26.5
26.3
26.2
26.2
23.3
21.6
21.4
20.5
20.0
19.8
19.7
18.5
18.3
15.8
15.1
1.2
-2.4
-3.2
-3.5
-4.8
Me
Me
tBuMe2SiO
H
O
H
OSiMe2tBu
Me
Me
Me
8
126 MHz, CDCl3
220
210
200
190
180
170
160
150
140
130
120
110
100
90
80
70
60
50
40
30
20
10
0
f1 (ppm)

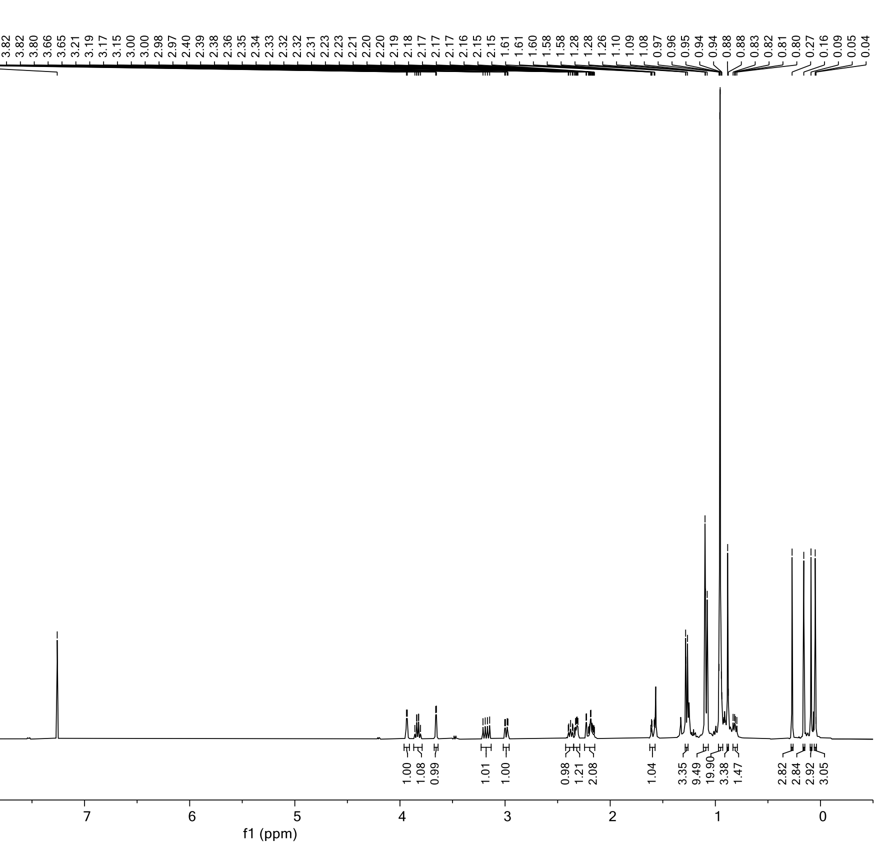

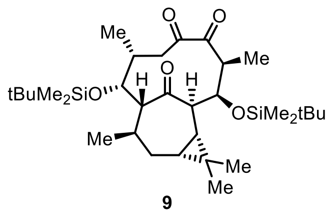

9

400 MHz, $CDCl_3$

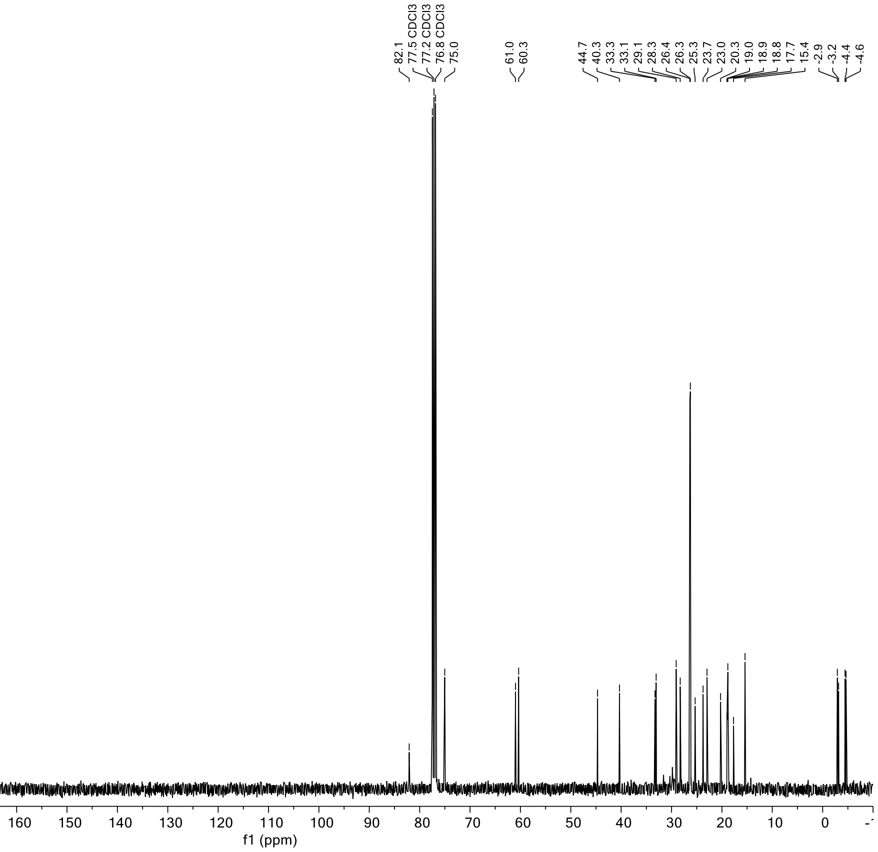

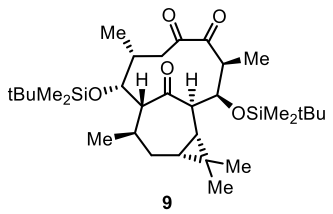

9

101 MHz, $CDCl_3$

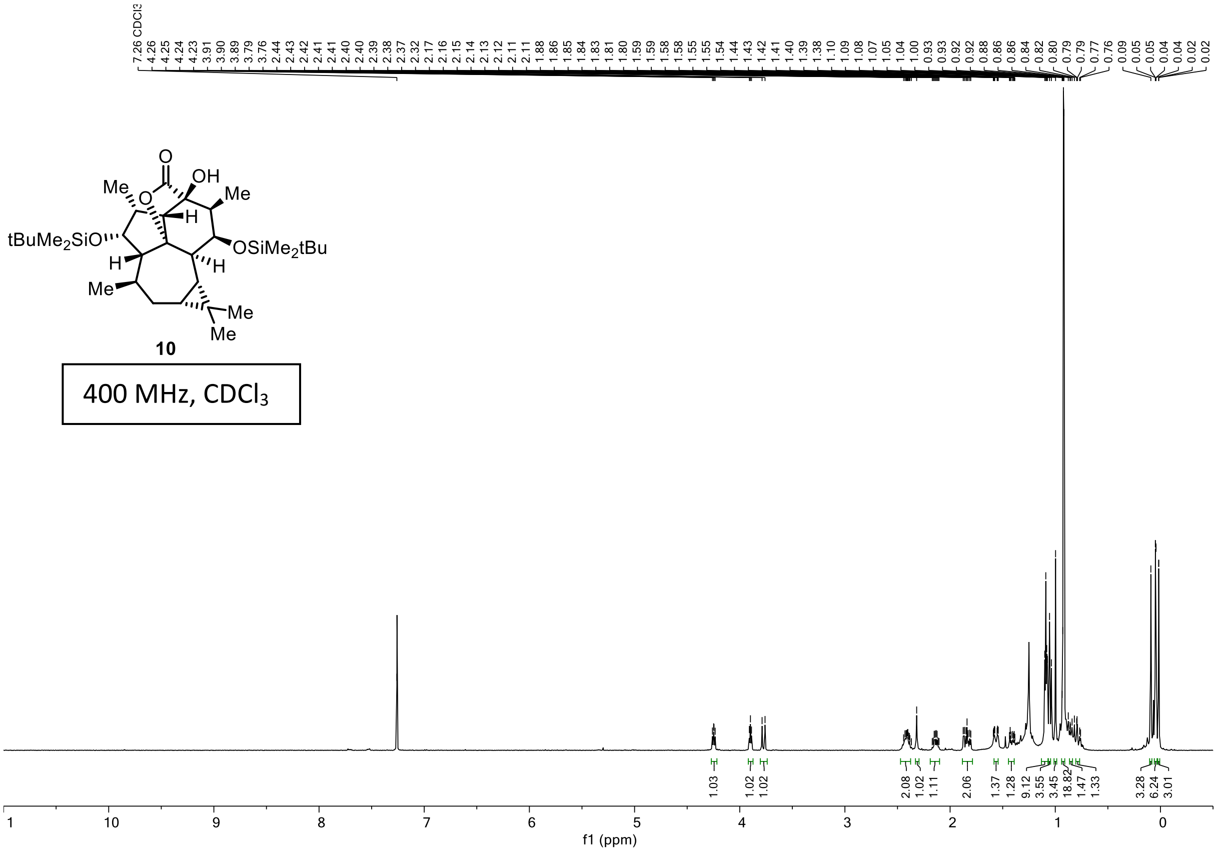
tBuMe2SiO
OSiMe2tBu
OH
Me
10
400 MHz, CDCl3
f1 (ppm)

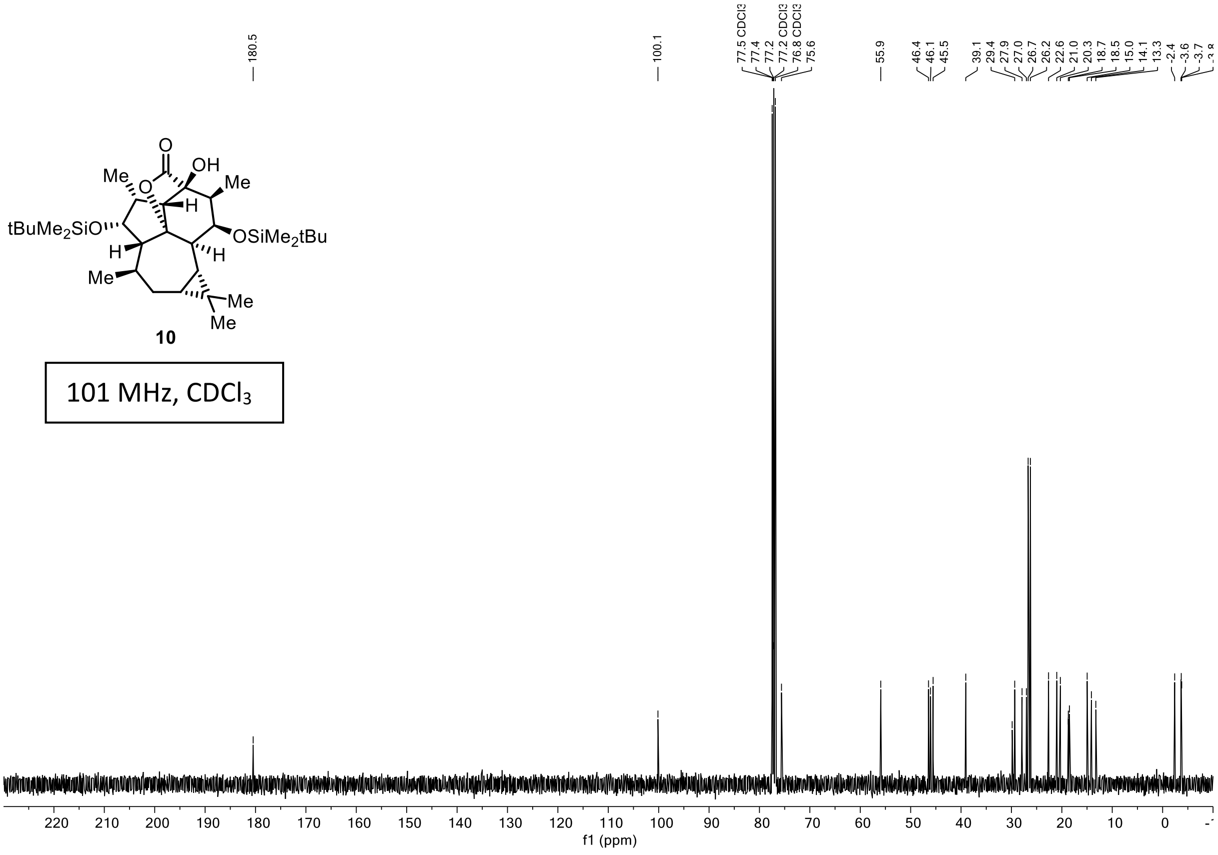
tBuMe2SiO
OSiMe2tBu
OH
Me
10
101 MHz, CDCl3
f1 (ppm)

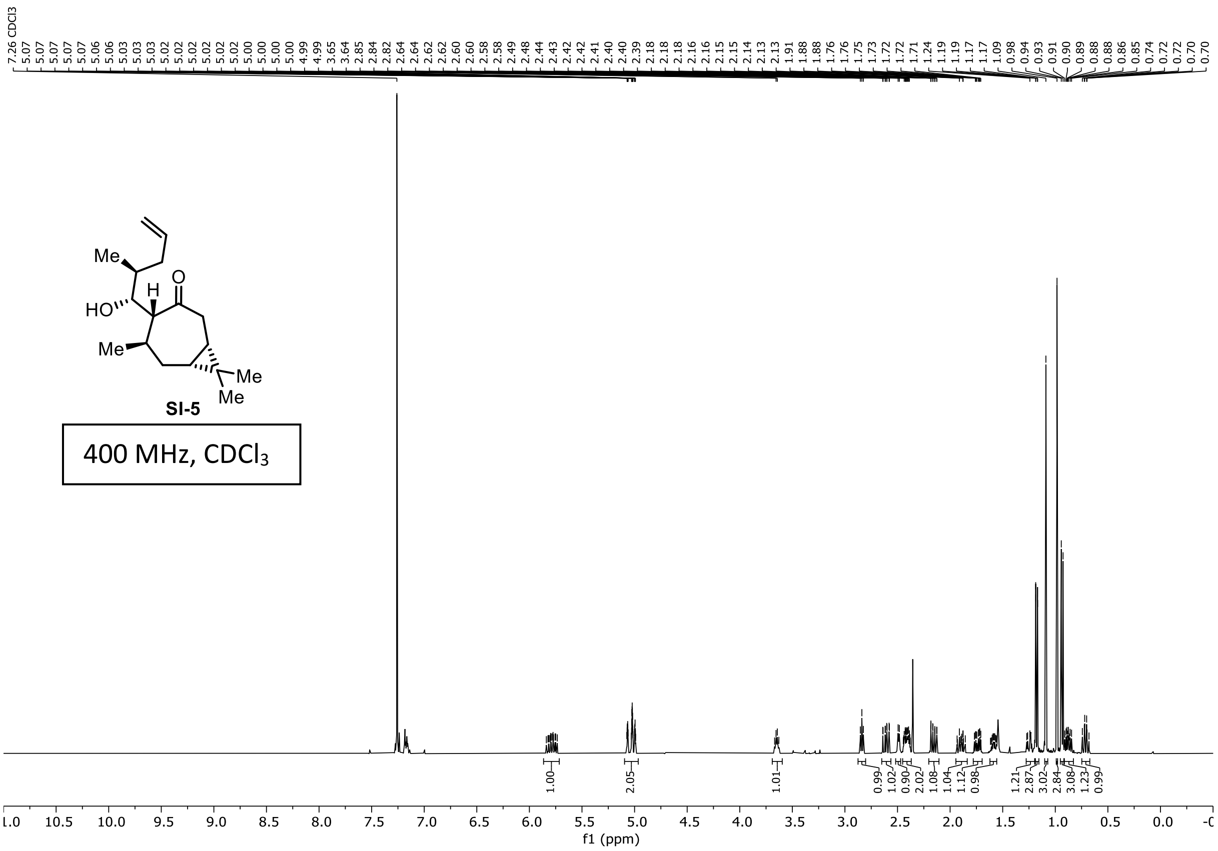
7.26 CDCl3
5.07 5.07 5.07 5.07 5.07 5.06 5.06 5.03 5.03 5.03 5.02 5.02 5.02 5.02 5.02 5.00 5.00 5.00 5.00 4.99 4.99 3.65 3.64 2.85 2.84 2.82 2.64 2.64 2.62 2.62 2.60 2.60 2.58 2.58 2.49 2.48 2.44 2.43 2.42 2.42 2.41 2.40 2.40 2.39 2.18 2.18 2.18 2.16 2.16 2.15 2.15 2.14 2.13 2.13 1.91 1.88 1.88 1.76 1.75 1.73 1.72 1.72 1.72 1.71 1.24 1.19 1.19 1.17 1.17 1.09 0.98 0.94 0.93 0.91 0.90 0.89 0.88 0.88 0.86 0.85 0.74 0.72 0.72 0.70 0.70
Me
HO
H
O
Me
Me
Me
SI-5
400 MHz, CDCl3
1.00 2.05 1.01 0.99 1.02 0.90 2.02 1.08 1.04 1.12 0.98 1.21 2.87 3.02 2.84 3.08 1.23 0.99
f1 (ppm)

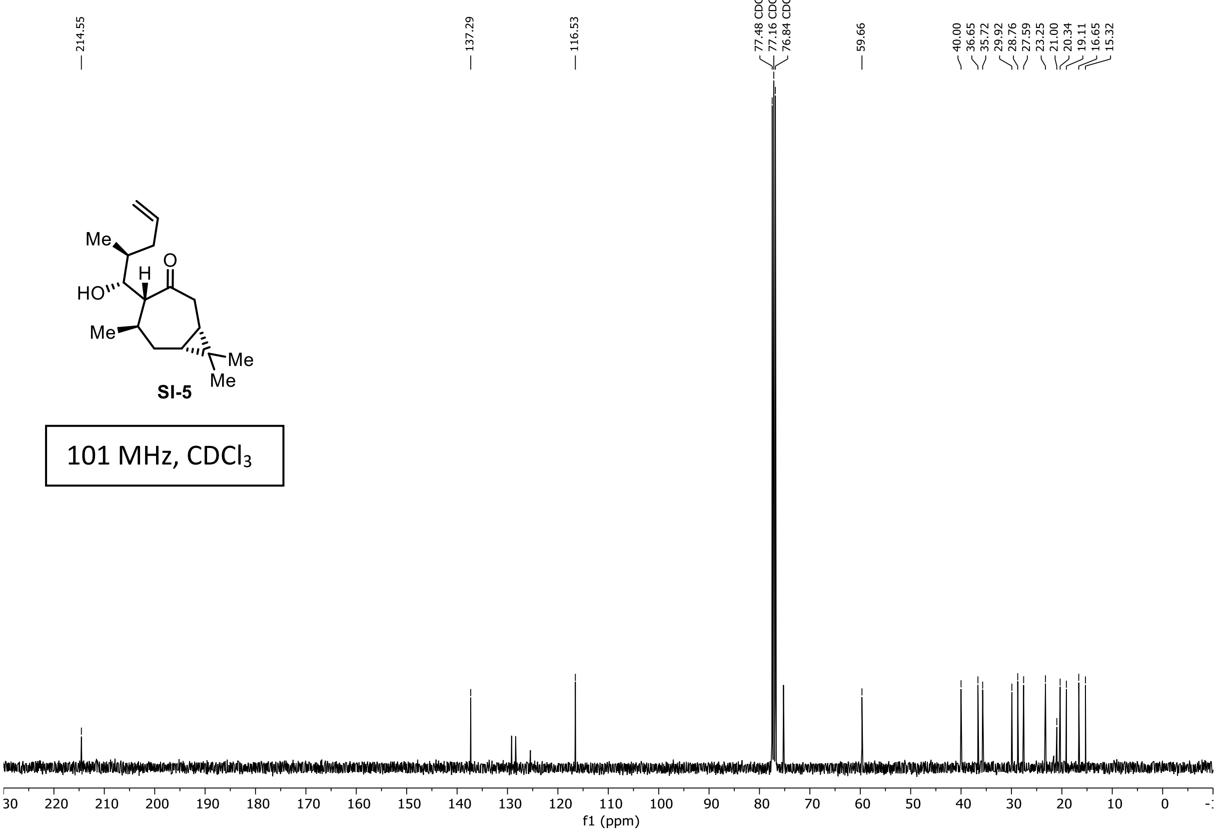
214.55
137.29
116.53
77.48 CDCl3
77.16 CDCl3
76.84 CDCl3
59.66
40.00 36.65 35.72 29.92 28.76 27.59 23.25 21.00 20.34 19.11 16.65 15.32
Me
HO
H
O
Me
Me
Me
SI-5
101 MHz, CDCl3
f1 (ppm)

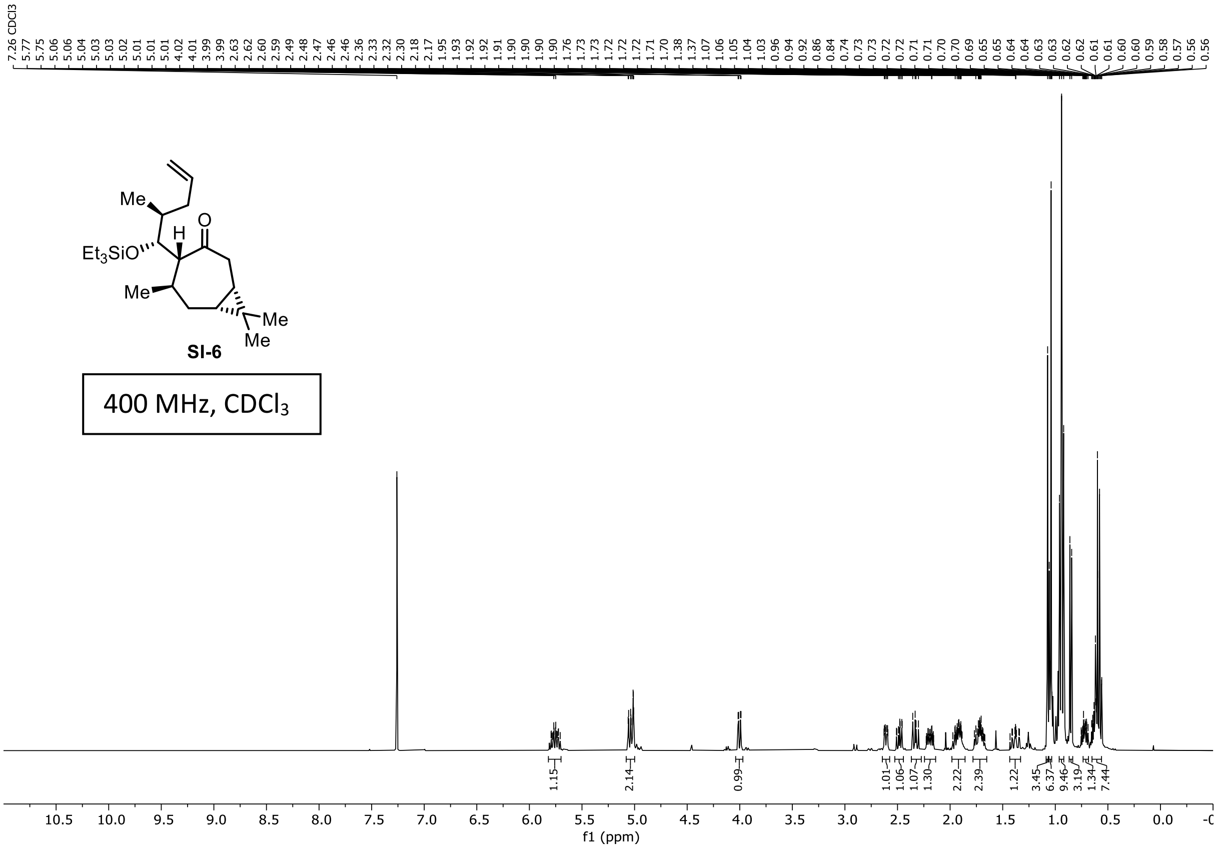
7.26 CDCl3
5.77
5.75
5.06
5.06
5.04
5.03
5.03
5.02
5.01
5.01
5.01
4.02
4.01
3.99
3.99
2.63
2.62
2.60
2.59
2.49
2.48
2.47
2.46
2.46
2.36
2.33
2.32
2.30
2.18
2.17
1.95
1.93
1.92
1.92
1.91
1.91
1.90
1.90
1.90
1.76
1.73
1.73
1.72
1.72
1.72
1.71
1.70
1.38
1.37
1.07
1.06
1.05
1.04
1.03
0.96
0.94
0.92
0.86
0.84
0.74
0.73
0.73
0.72
0.72
0.71
0.71
0.70
0.70
0.69
0.65
0.65
0.64
0.64
0.63
0.63
0.62
0.62
0.61
0.61
0.60
0.60
0.59
0.58
0.57
0.56
0.56
Me
Et3SiO
H
O
Me
Me
Me
SI-6
400 MHz, CDCl3
1.15
2.14
0.99
1.01
1.06
1.07
1.30
2.22
2.39
1.22
3.45
6.37
9.46
3.19
1.34
7.44
f1 (ppm)

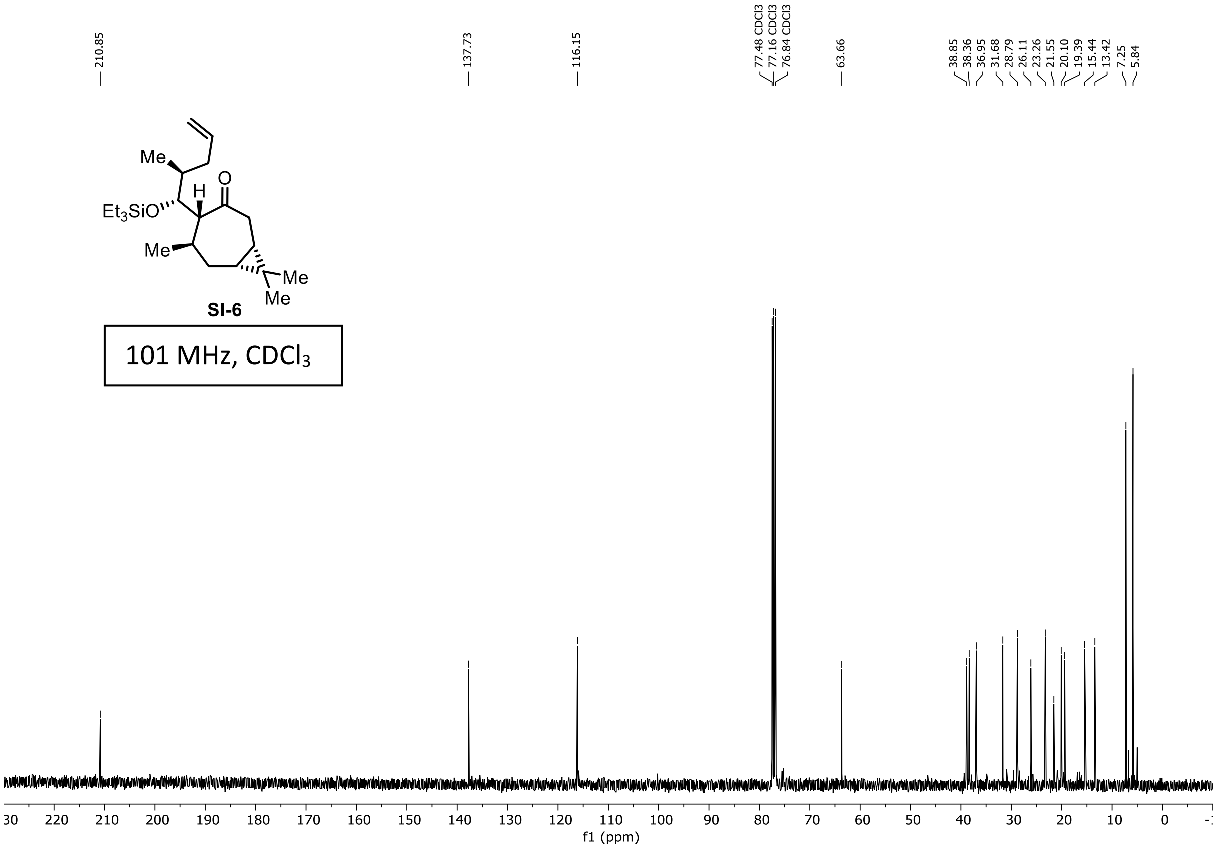
210.85
137.73
116.15
77.48 CDCl3
77.16 CDCl3
76.84 CDCl3
63.66
38.85
38.36
36.95
31.68
28.79
26.11
23.26
21.55
20.10
19.39
15.44
13.42
7.25
5.84
Me
Et3SiO
H
O
Me
Me
Me
SI-6
101 MHz, CDCl3
f1 (ppm)

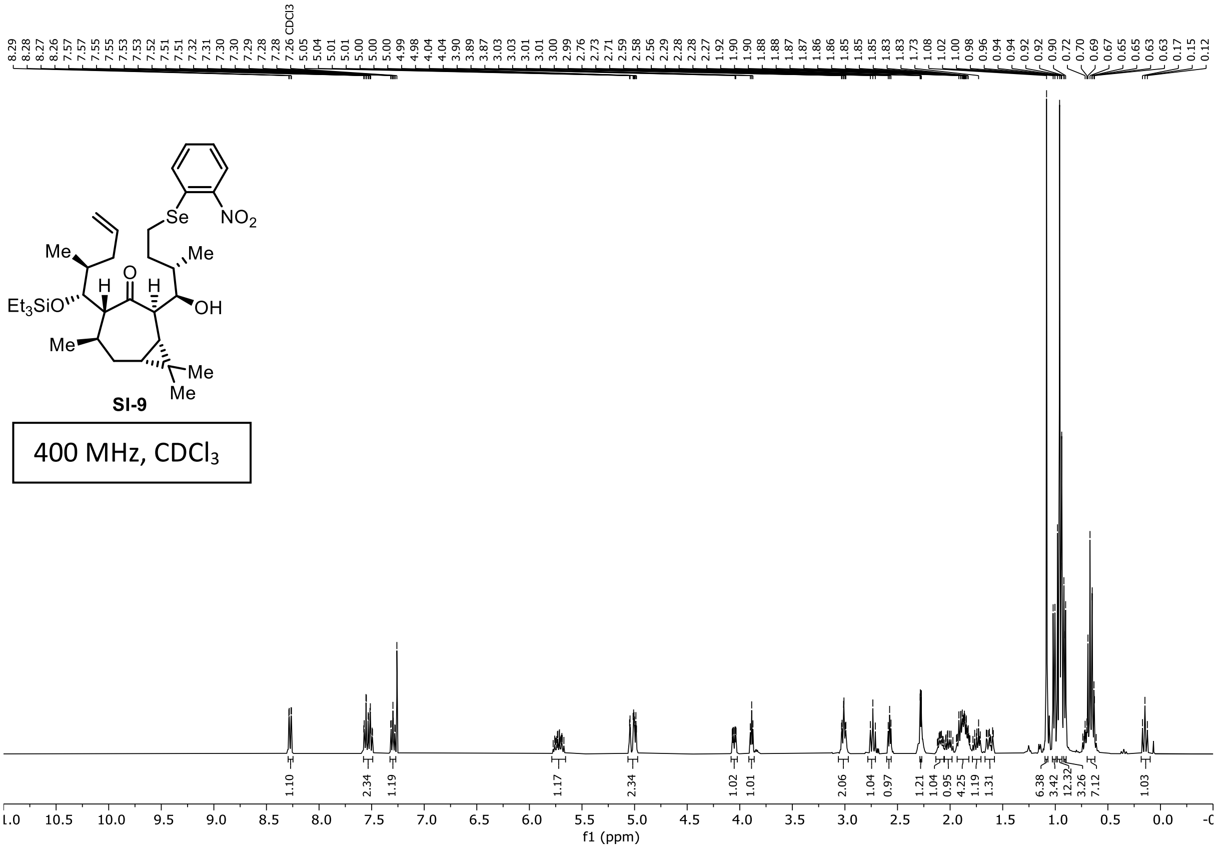
8.29
8.28
8.27
8.26
7.57
7.57
7.55
7.55
7.53
7.53
7.52
7.51
7.51
7.32
7.31
7.30
7.30
7.29
7.28
7.28
7.26 CDCl3
5.05
5.04
5.01
5.01
5.00
5.00
4.99
4.98
4.04
4.04
3.90
3.89
3.87
3.03
3.03
3.01
3.01
3.00
2.99
2.76
2.73
2.71
2.59
2.58
2.56
2.29
2.28
2.28
2.27
1.92
1.90
1.90
1.88
1.88
1.87
1.87
1.86
1.86
1.85
1.85
1.85
1.83
1.83
1.73
1.08
1.02
1.00
0.98
0.96
0.94
0.94
0.92
0.92
0.90
0.72
0.70
0.69
0.67
0.65
0.65
0.63
0.63
0.17
0.15
0.12
Se
NO2
Me
Me
H
O
H
Et3SiO
OH
Me
Me
Me
SI-9
400 MHz, CDCl3
1.10
2.34
1.19
1.17
2.34
1.02
1.01
2.06
1.04
0.97
1.21
1.04
0.95
4.25
1.19
1.31
6.38
3.42
12.32
3.26
7.12
1.03
10.5
10.0
9.5
9.0
8.5
8.0
7.5
7.0
6.5
6.0
5.5
5.0
4.5
4.0
3.5
3.0
2.5
2.0
1.5
1.0
0.5
0.0
f1 (ppm)

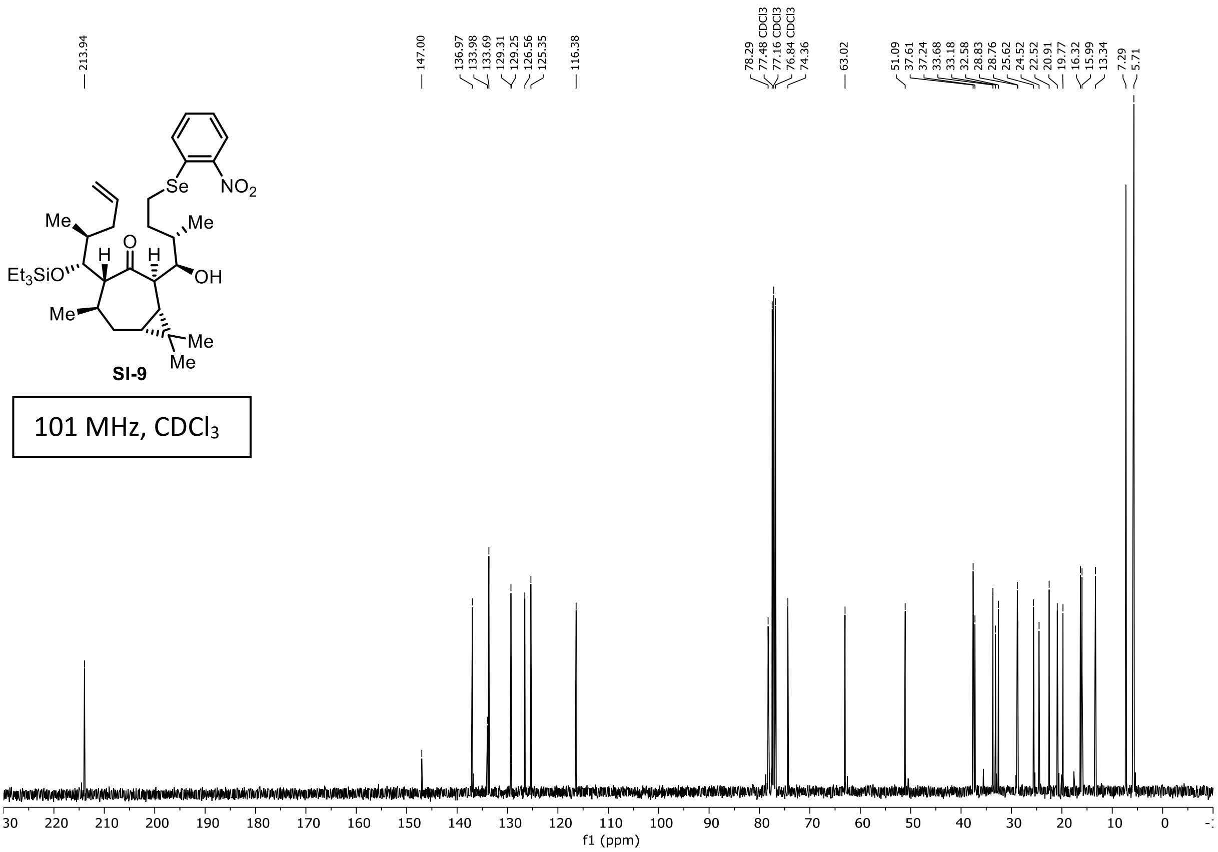
213.94
147.00
136.97
133.98
133.69
129.31
129.25
126.56
125.35
116.38
78.29
77.48 CDCl3
77.16 CDCl3
76.84 CDCl3
74.36
63.02
51.09
37.61
37.24
33.68
33.18
32.58
28.83
28.76
25.62
24.52
22.52
20.91
19.77
16.32
15.99
13.34
7.29
5.71
Se
NO2
Me
Me
H
O
H
Et3SiO
OH
Me
Me
Me
SI-9
101 MHz, CDCl3
220
210
200
190
180
170
160
150
140
130
120
110
100
90
80
70
60
50
40
30
20
10
0
f1 (ppm)

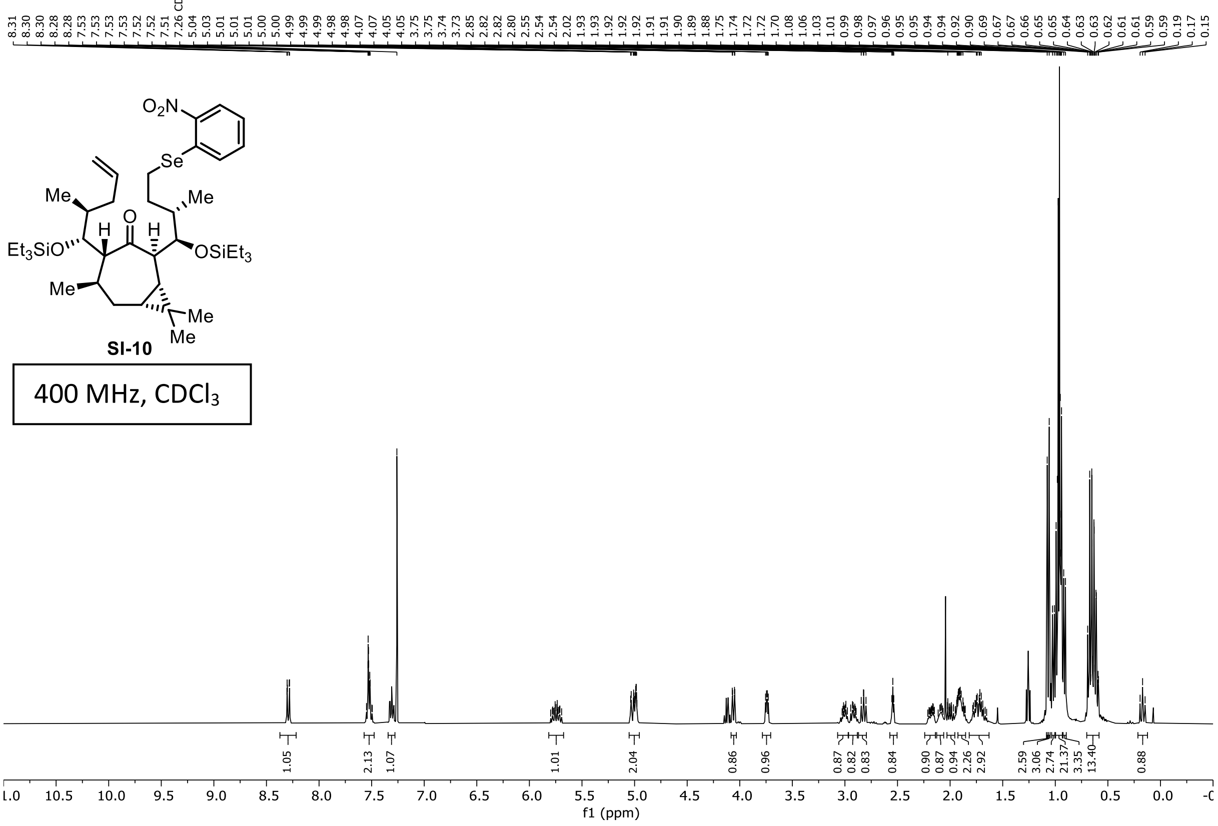
SI-10
400 MHz, CDCl3
f1 (ppm)

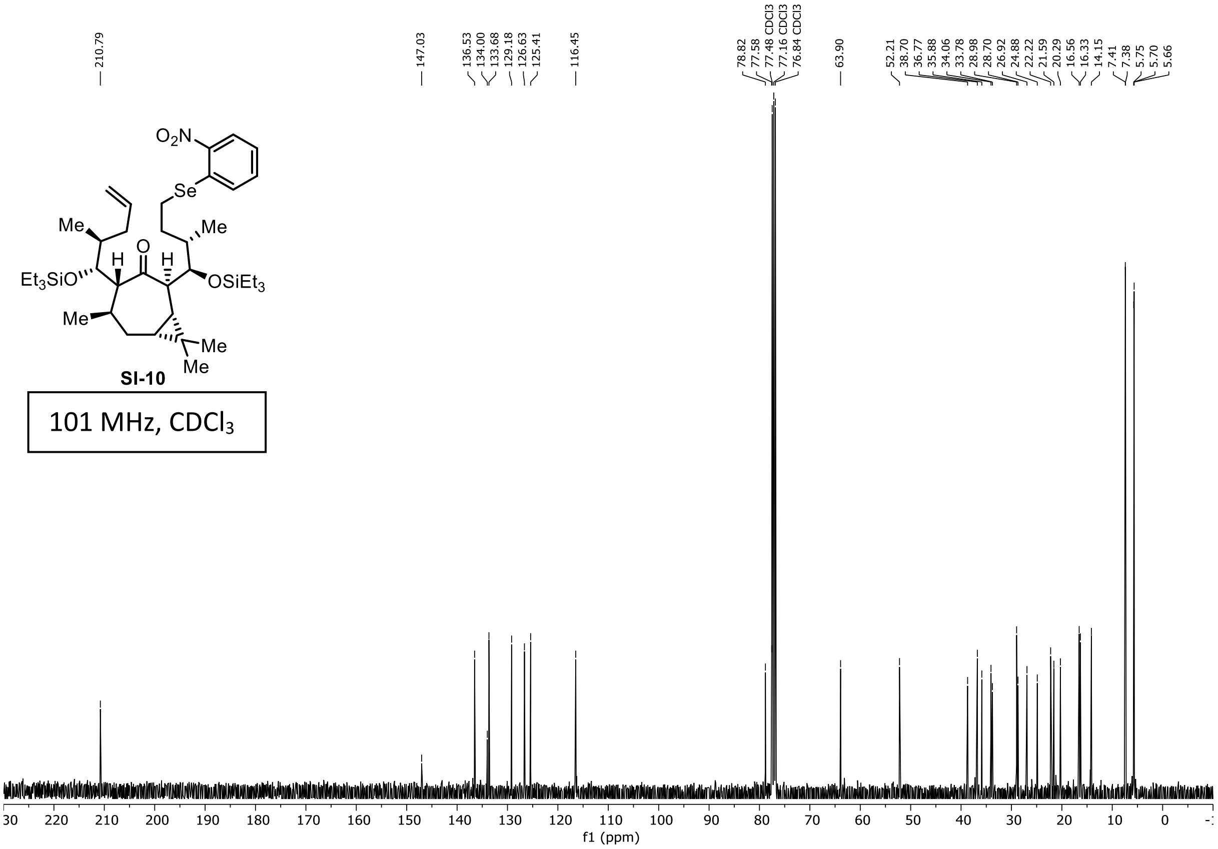
SI-10
101 MHz, CDCl3
f1 (ppm)

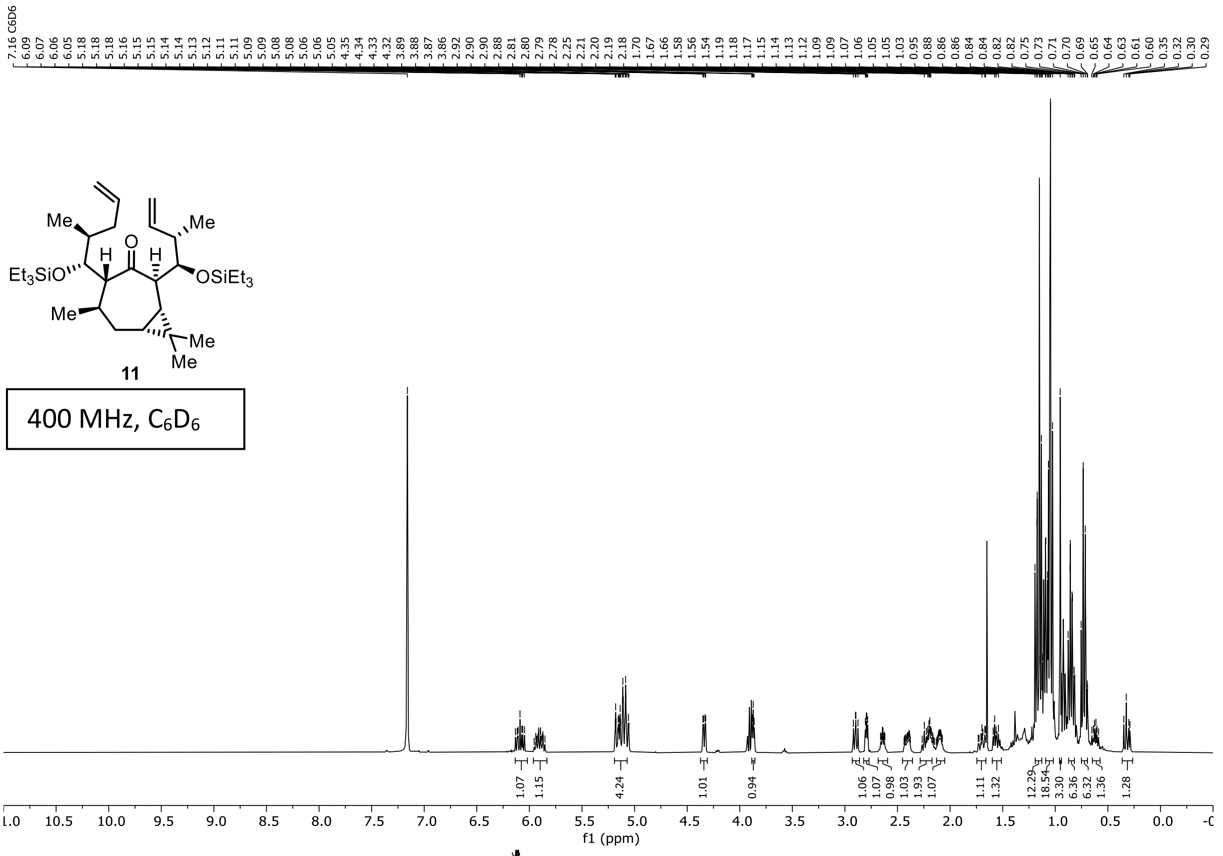
11
400 MHz, C_6D_6
f1 (ppm)

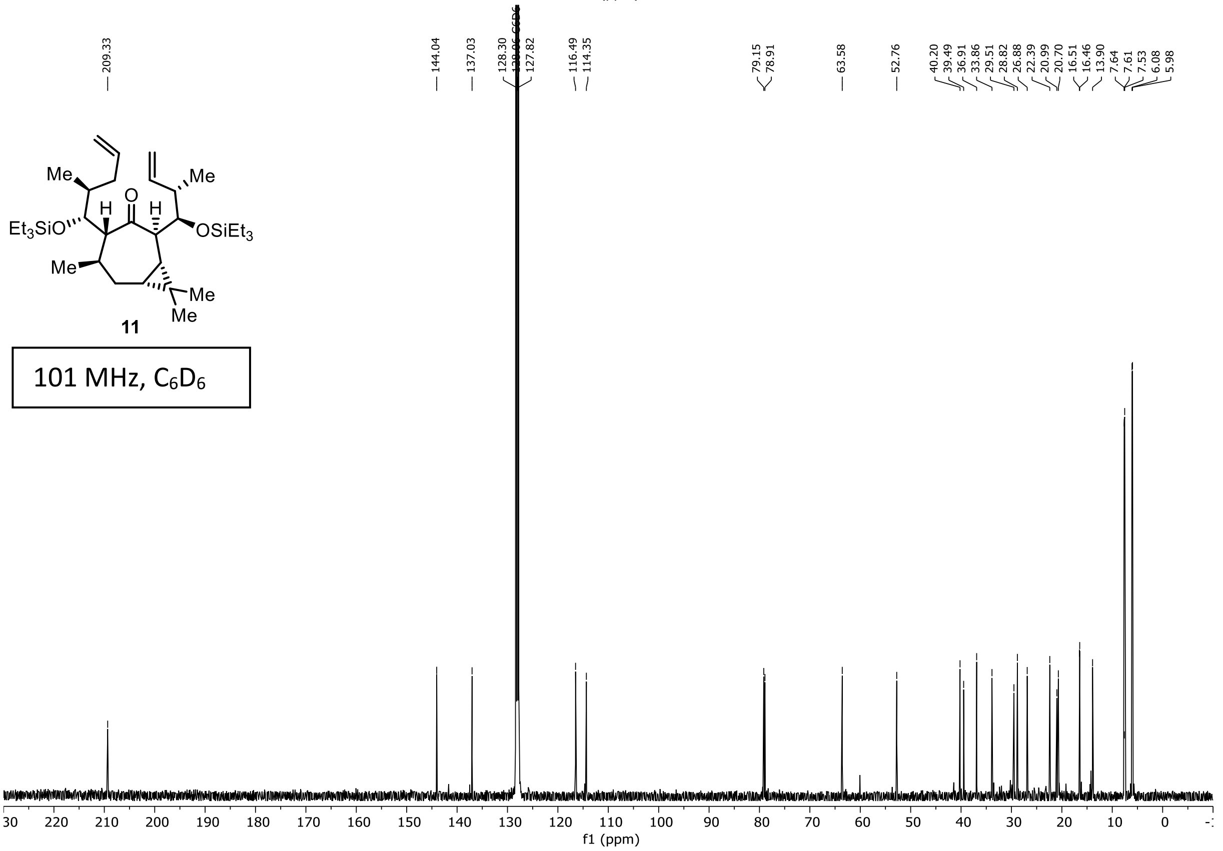
11
101 MHz, C_6D_6
f1 (ppm)

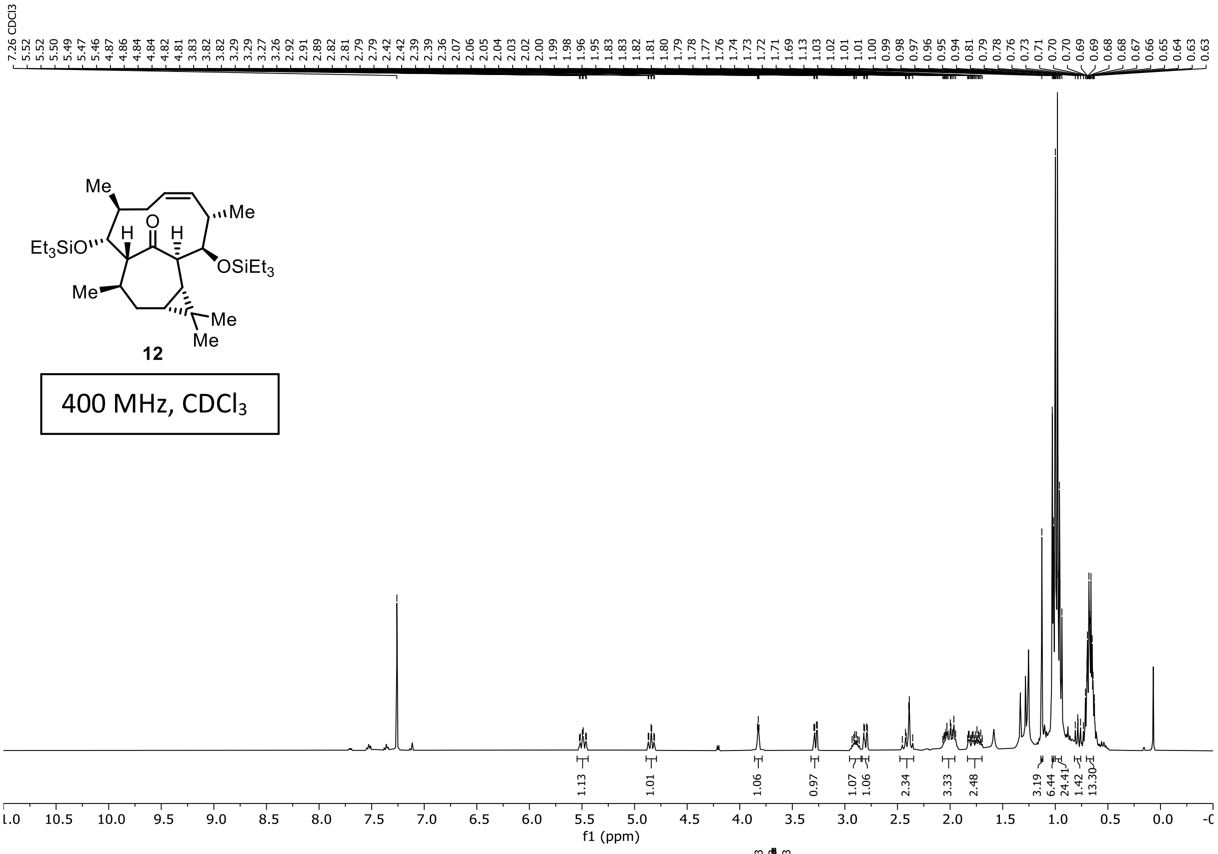
Me
O
H
H
Me
Et3SiO
OSiEt3
Me
Me
Me
12
400 MHz, CDCl3
7.26 CDCl3
1.13
1.01
1.06
0.97
1.07
1.06
2.34
3.33
2.48
3.19
6.44
24.41
1.42
13.30
f1 (ppm)

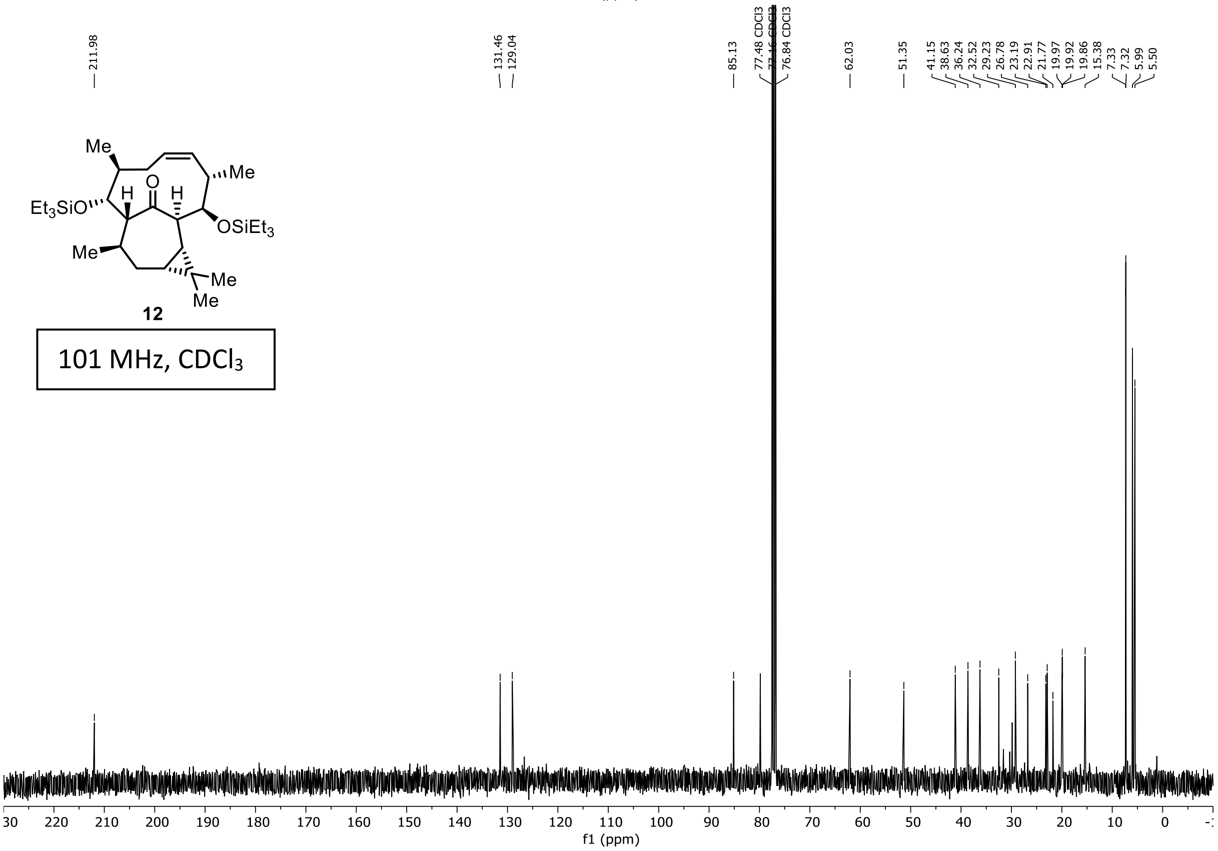
211.98
131.46
129.04
85.13
77.48 CDCl3
76.84 CDCl3
62.03
51.35
41.15
38.63
36.24
32.52
29.23
26.78
23.19
22.91
21.77
19.97
19.92
19.86
15.38
7.33
7.32
5.99
5.50
Me
O
H
H
Me
Et3SiO
OSiEt3
Me
Me
Me
12
101 MHz, CDCl3
f1 (ppm)

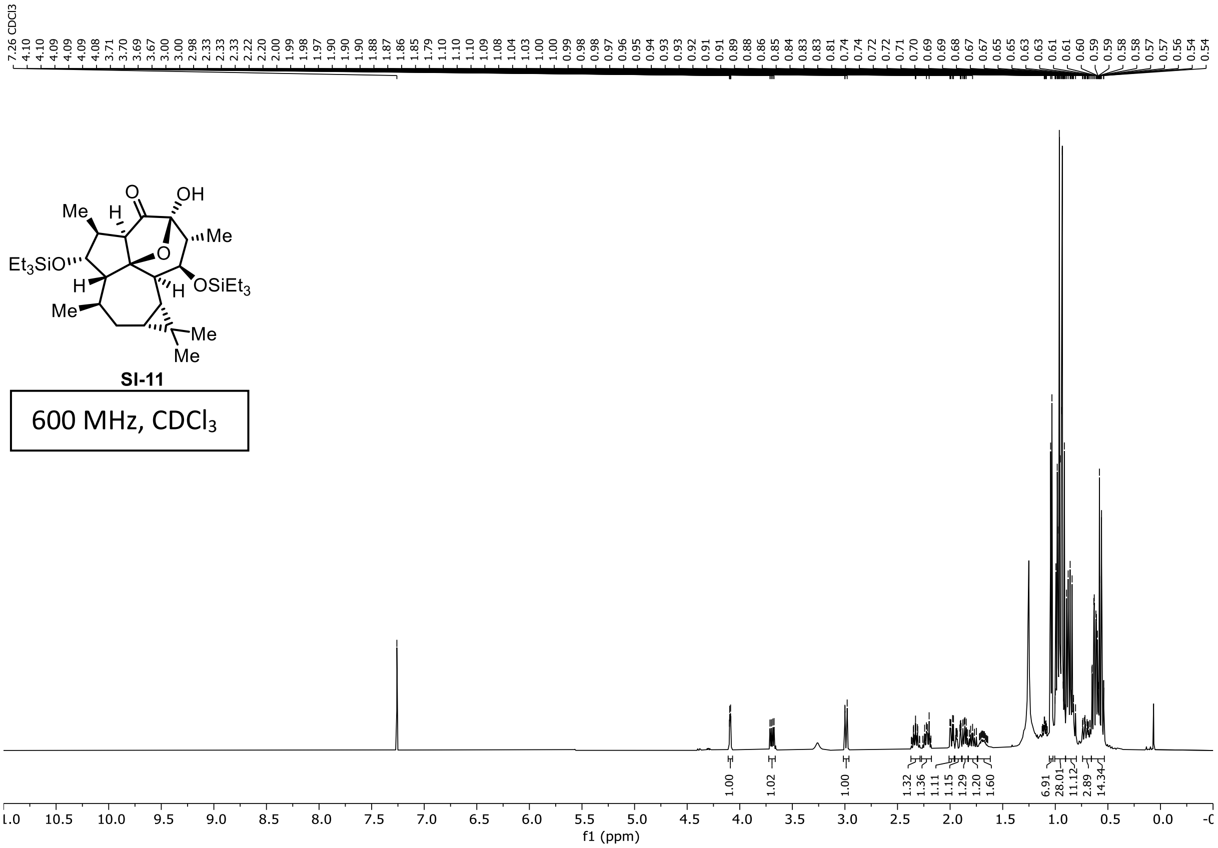

SI-11

600 MHz, $CDCl_3$

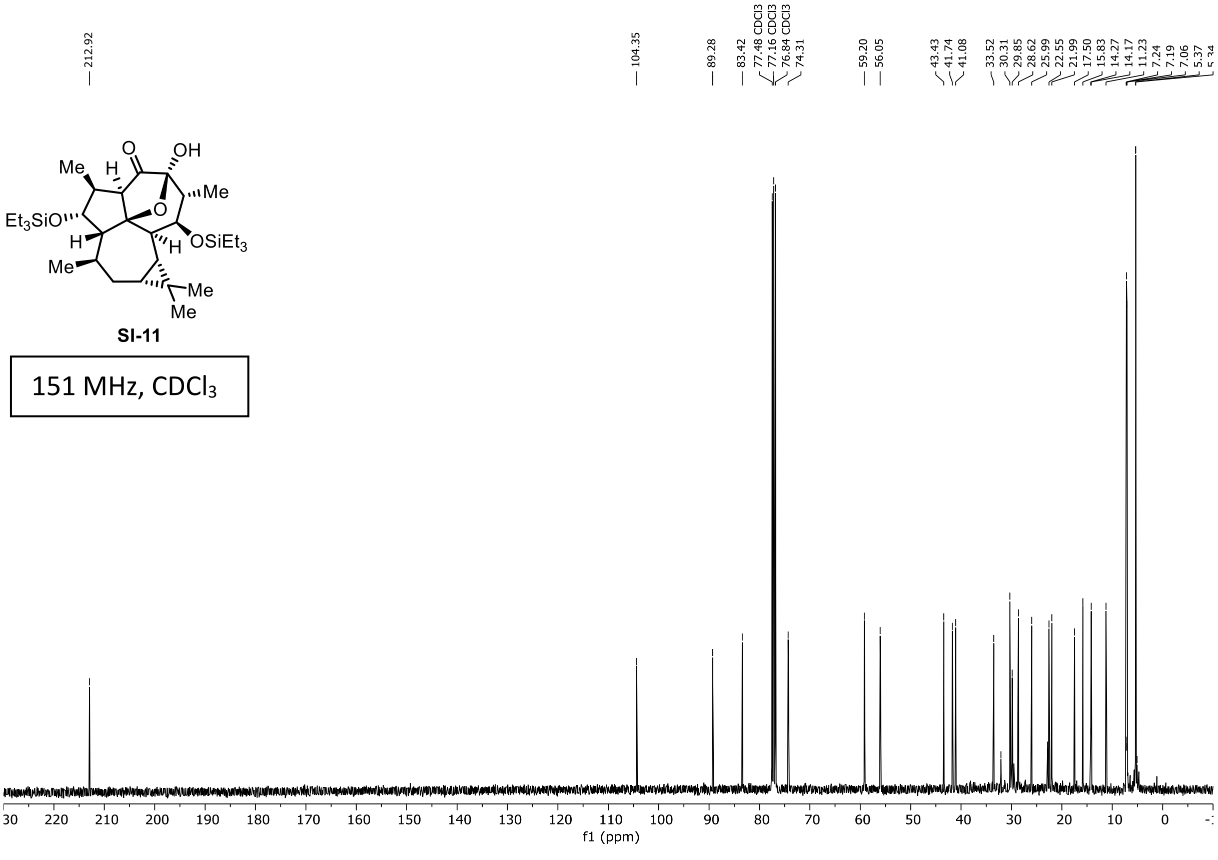

SI-11

151 MHz, $CDCl_3$

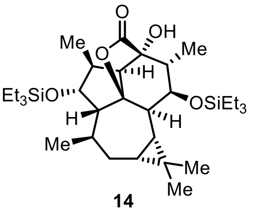

14

600 MHz, $CDCl_3$

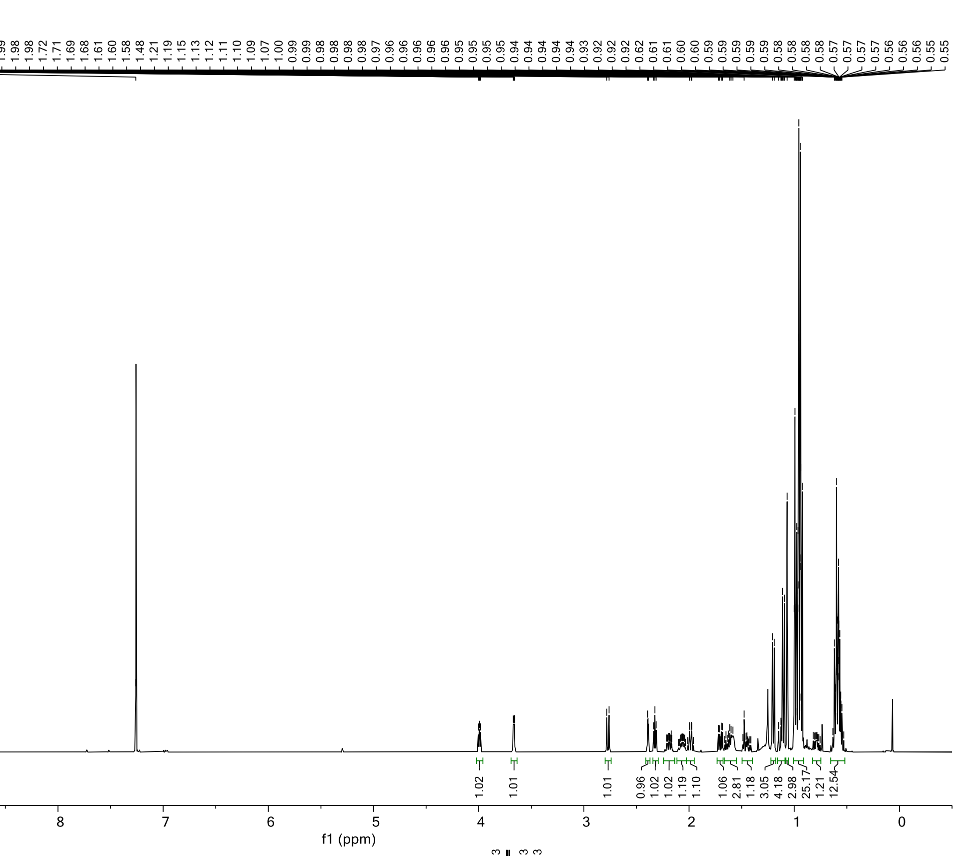

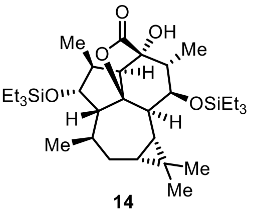

14

151 MHz, $CDCl_3$

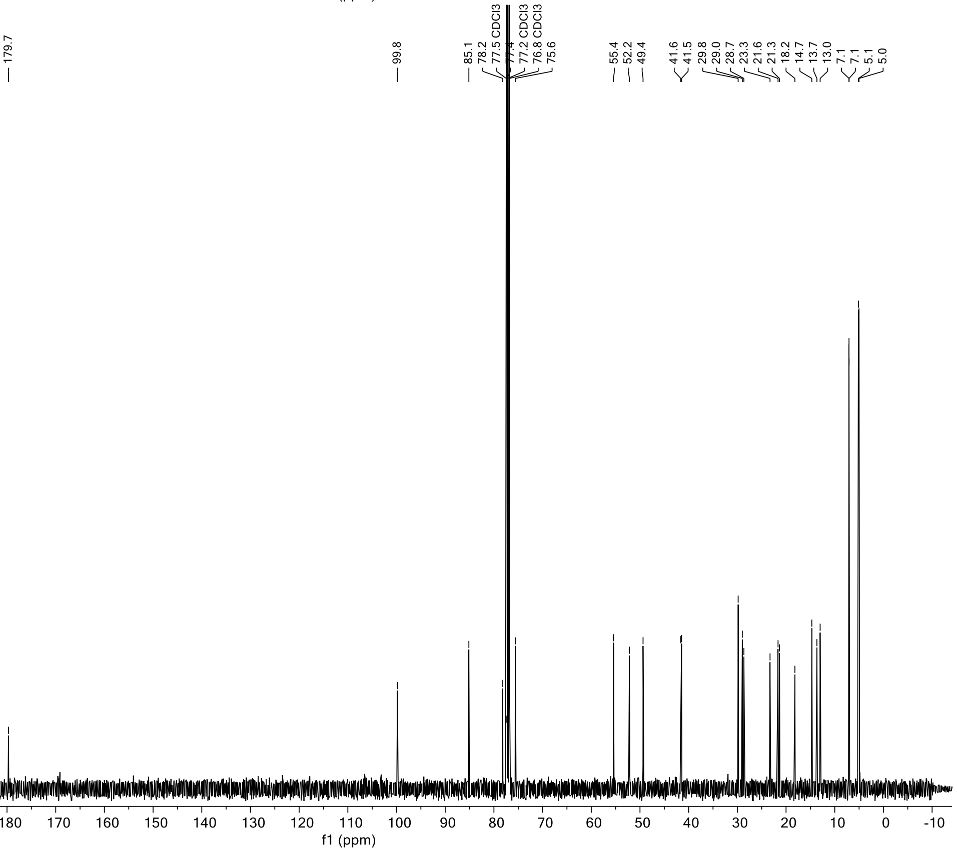

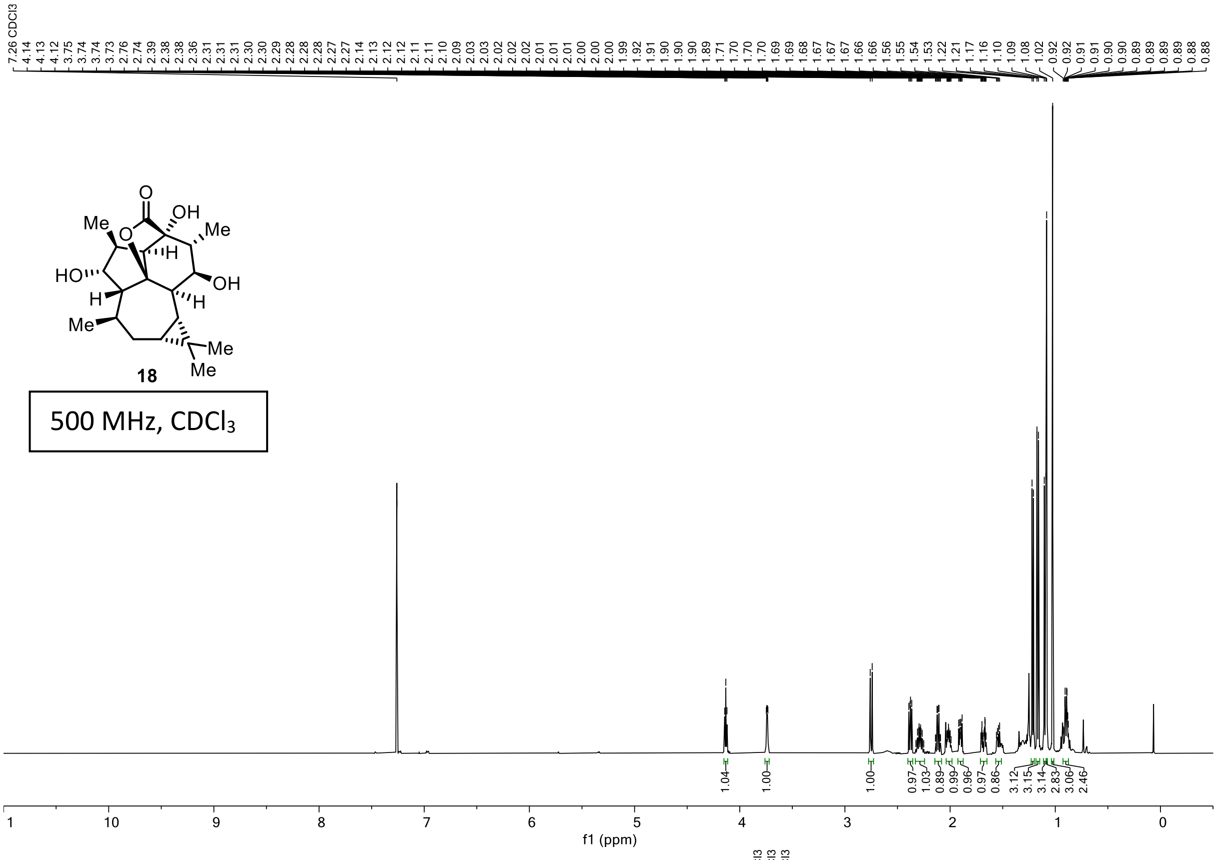

18
500 MHz, $CDCl_3$
f1 (ppm)

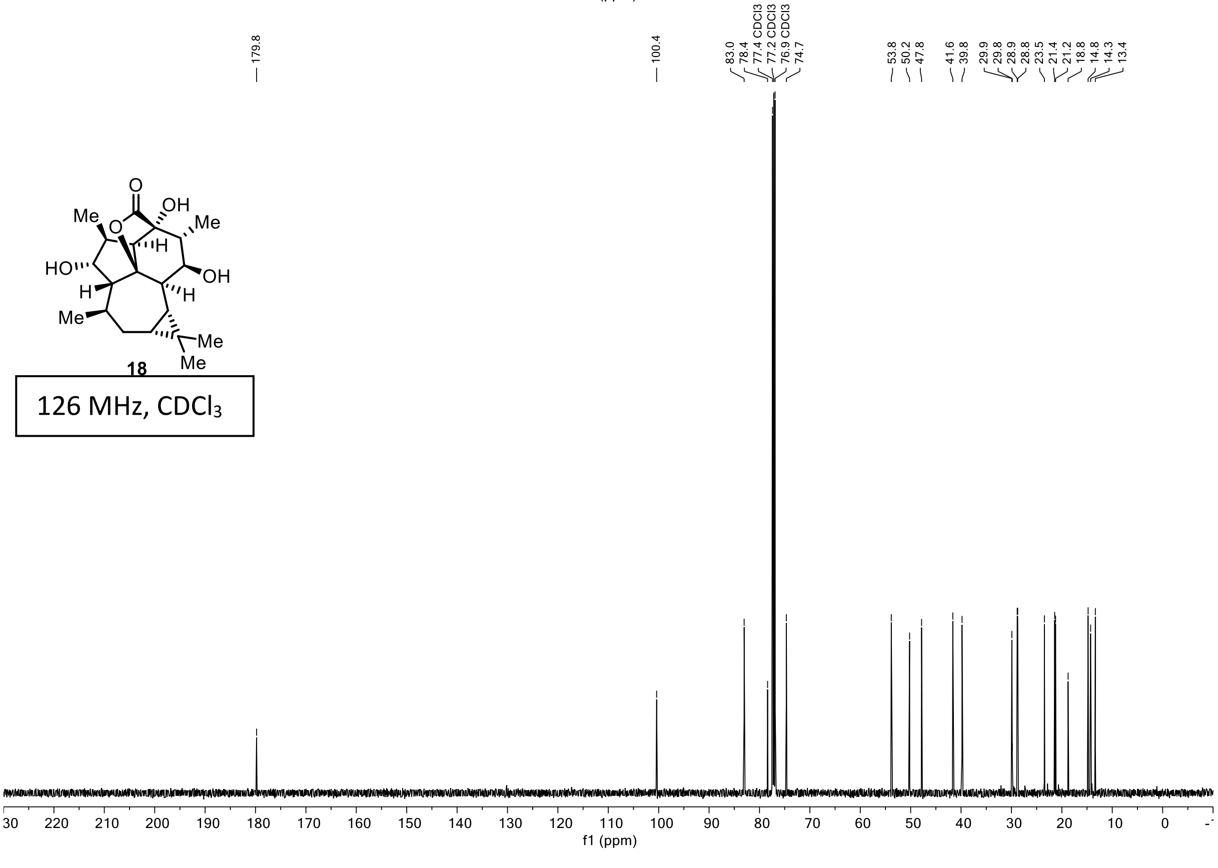

179.8
100.4
83.0
78.4
77.4 CDCl3
77.2 CDCl3
76.9 CDCl3
74.7
53.8
50.2
47.8
41.6
39.8
29.9
29.8
28.9
28.8
23.5
21.4
21.2
18.8
14.8
14.3
13.4
18
126 MHz, $CDCl_3$
f1 (ppm)

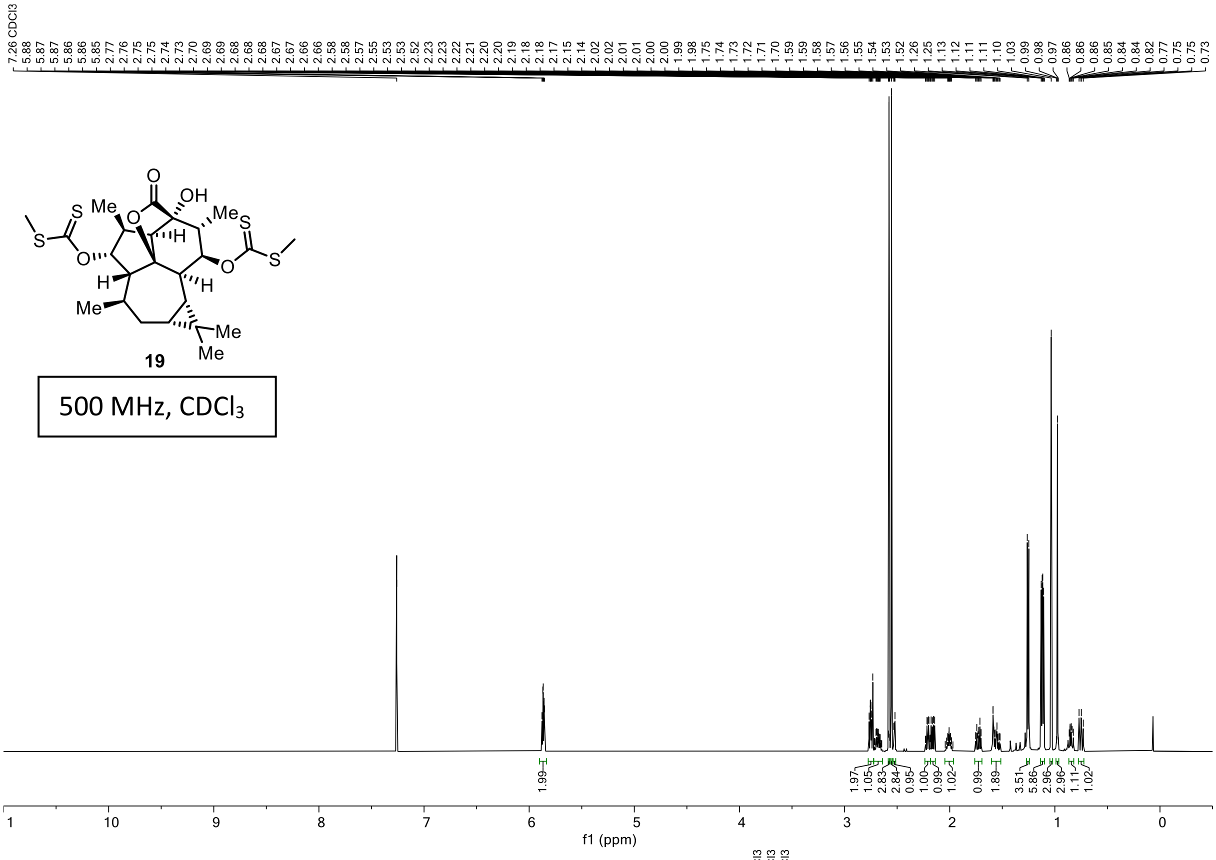
7.26 CDCl3
5.88 5.87 5.87 5.86 5.86 5.85 2.77 2.76 2.75 2.75 2.74 2.73 2.70 2.69 2.69 2.68 2.68 2.68 2.67 2.67 2.66 2.66 2.58 2.58 2.57 2.55 2.53 2.53 2.52 2.23 2.23 2.22 2.21 2.20 2.20 2.19 2.18 2.18 2.17 2.15 2.14 2.02 2.02 2.01 2.01 2.00 2.00 1.99 1.98 1.75 1.74 1.73 1.72 1.71 1.70 1.59 1.59 1.58 1.57 1.56 1.55 1.54 1.53 1.52 1.26 1.25 1.13 1.12 1.11 1.11 1.10 1.03 0.99 0.98 0.97 0.86 0.86 0.86 0.85 0.84 0.84 0.82 0.77 0.75 0.73
Me
O
OH
Me
S
S
O
O
S
S
H
H
H
Me
Me
Me
19
500 MHz, CDCl3
1.99
1.97 1.05 2.83 2.84 0.95 1.00 0.99 1.02 0.99 1.89 3.51 5.86 2.96 2.90 1.11 1.02
f1 (ppm)

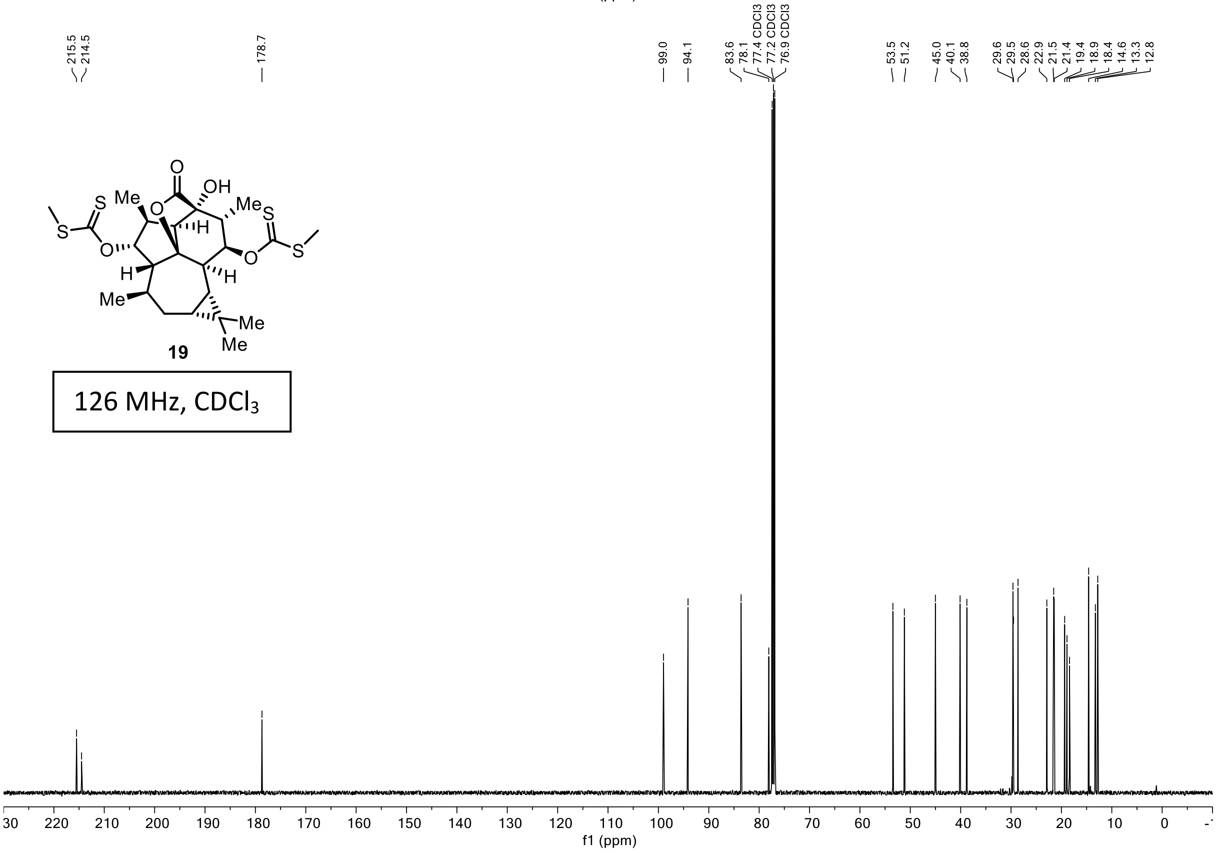
215.5 214.5
178.7
99.0
94.1
83.6 78.1 77.4 CDCl3 77.2 CDCl3 76.9 CDCl3
53.5 51.2
45.0 40.1 38.8
29.6 29.5 28.6 22.9 21.5 21.4 19.4 18.9 18.4 14.6 13.3 12.8
Me
O
OH
Me
S
S
O
O
S
S
H
H
H
Me
Me
Me
19
126 MHz, CDCl3
f1 (ppm)

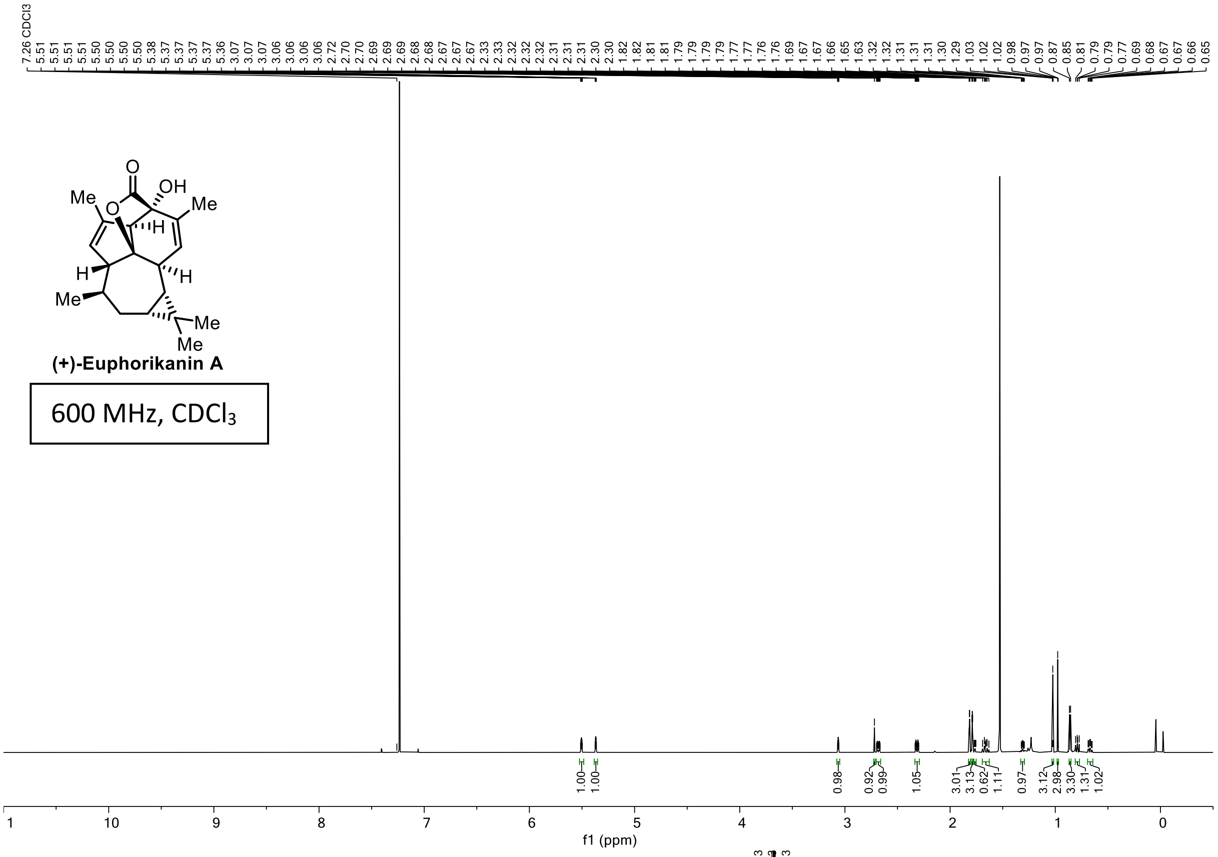

(+)-Euphorikanin A
600 MHz, CDCl3
f1 (ppm)

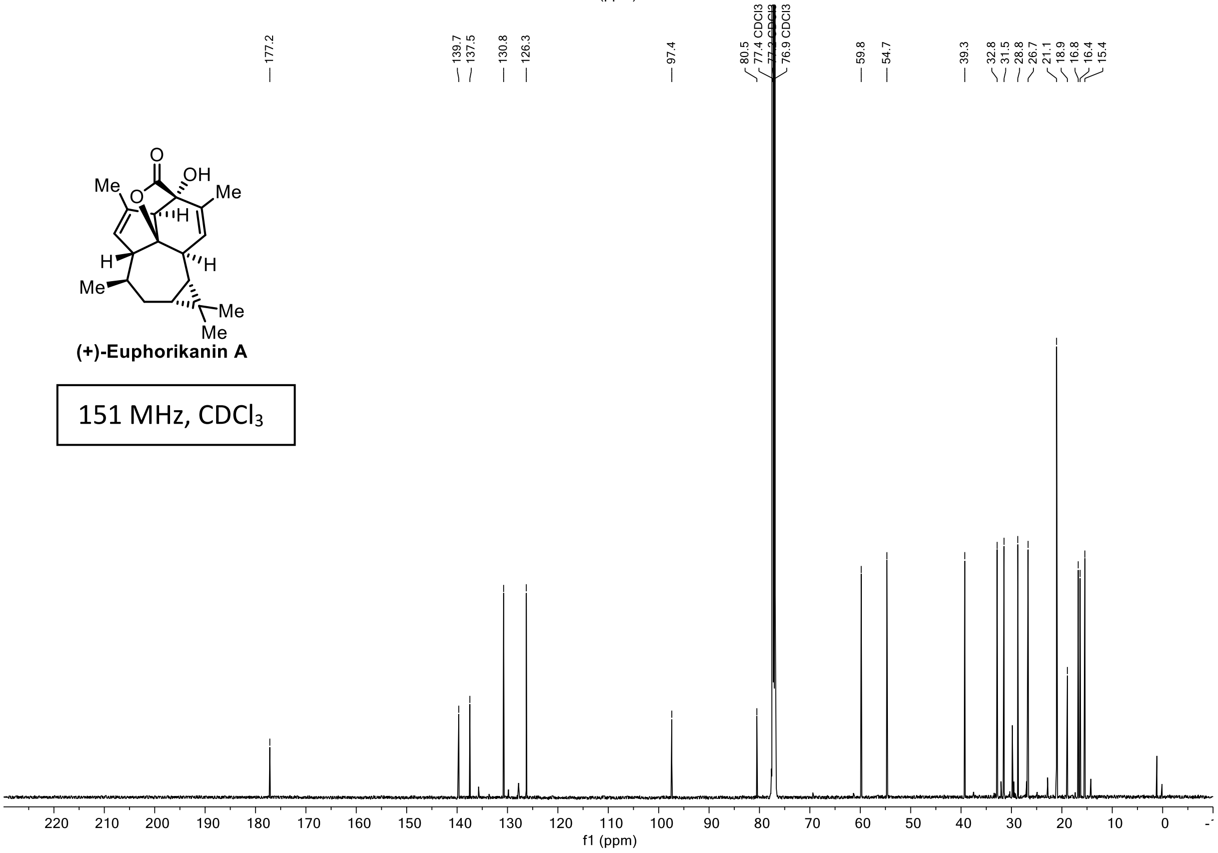

177.2
139.7
137.5
130.8
126.3
97.4
80.5
77.4 CDCl3
76.9 CDCl3
59.8
54.7
39.3
32.8
31.5
28.8
26.7
21.1
18.9
16.8
16.4
15.4
(+)-Euphorikanin A
151 MHz, CDCl3
f1 (ppm)

Comparison of natural and synthetic 1.

H	Natural [ppm][8]	Synthetic [ppm]
**1**	5.53	5.53
**2**	-	-
**3**	3.09	3.09
**4**	-	-
**5**	2.71	2.71
**6**	1.34	1.35 – 1.32
**7**	1.79, 1.68	1.79, 1.69
**8**	0.68	0.69
**9**	-	-
**10**	0.81	0.81
**11**	2.34	2.34
**12**	5.40	5.39
**13**	-	-
**14**	-	-
**15**	-	-
**16**	1.84	1.84
**17**	0.89	0.88
**18**	1.05	1.05
**19**	1.00	1.00
**20**	1.81	1.81

C	Natural [ppm][8]	Synthetic [ppm]
**1**	130.6	130.6
**2**	137.3	137.3
**3**	59.6	59.7
**4**	97.3	97.3
**5**	54.5	54.6
**6**	31.3	31.3
**7**	32.7	32.7
**8**	20.8	20.9
**9**	18.8	18.8
**10**	26.5	26.6
**11**	39.1	39.1
**12**	126.1	126.1
**13**	139.5	139.5
**14**	80.4	80.4
**15**	177.0	177.0
**16**	16.2	16.3
**17**	20.9	20.9
**18**	28.6	28.6
**19**	15.3	15.3
**20**	16.6	16.6

IR spectrum of 1:

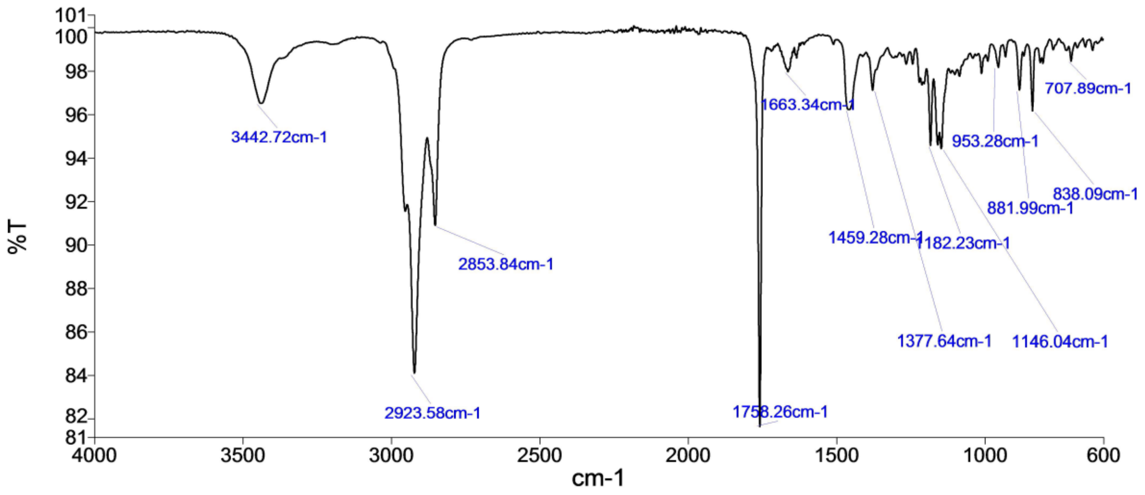

X-Ray Crystallographic Data

Ten membered ring triketone **9**:

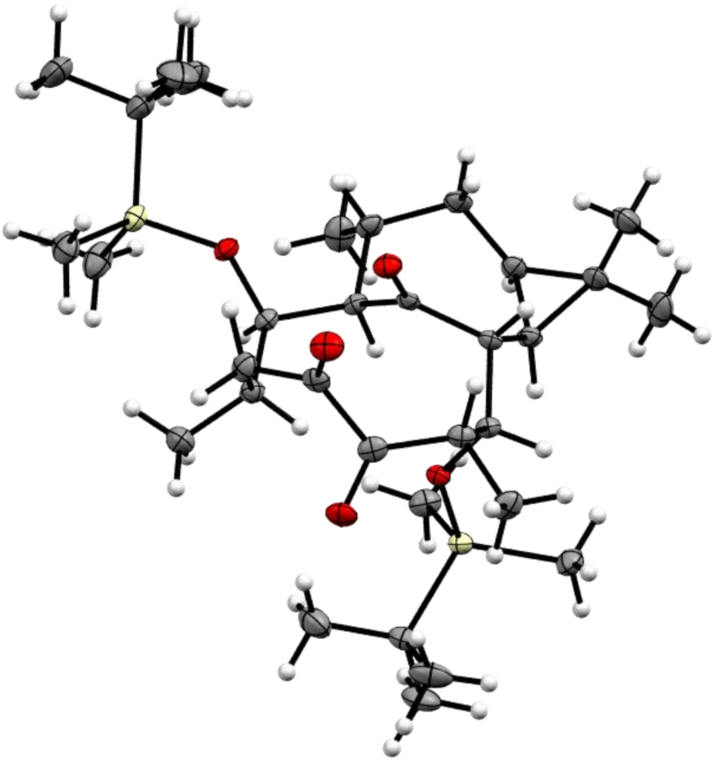

Table 1 Crystal data and structure refinement for ca260221_1_1.

Identification code	ca260221_1_1
Empirical formula	$C_{32}H_{58}O_5Si_2$
Formula weight	578.96
Temperature/K	100.0(1)
Crystal system	monoclinic
Space group	$P2_1$
a/Å	10.102(3)
b/Å	12.087(4)
c/Å	14.846(4)
α/°	90
β/°	102.971(7)
γ/°	90
Volume/Å^3	1766.5(8)
Z	2
ρ_{calc}g/cm^3	1.088
μ/mm^{-1}	0.134
F(000)	636.0
Crystal size/mm^3	0.335 × 0.21 × 0.06
Radiation	MoKα (λ = 0.71073)

2Θ range for data collection/°	4.138 to 54.956
Index ranges	$-13 \le h \le 13$, $-15 \le k \le 8$, $-19 \le l \le 19$
Reflections collected	28705
Independent reflections	6281 [R_{int} = 0.0325, R_{sigma} = 0.0283]
Data/restraints/parameters	6281/1/367
Goodness-of-fit on F^2	1.044
Final R indexes [I>=2σ (I)]	R_1 = 0.0307, wR_2 = 0.0735
Final R indexes [all data]	R_1 = 0.0352, wR_2 = 0.0758
Largest diff. peak/hole / e Å^{-3}	0.30/-0.19
Flack parameter	0.03(4)

Table 2 Fractional Atomic Coordinates (×10^4) and Equivalent Isotropic Displacement Parameters (Å^2×10^3) for ca260221_1_1. U_{eq} is defined as 1/3 of of the trace of the orthogonalised U_{IJ} tensor.

Atom	*x*	*y*	*z*	U(eq)
Si1	4410.9(5)	8221.2(5)	2571.9(4)	15.96(12)
Si2	1286.9(6)	2428.1(5)	1261.2(4)	18.79(13)
O1	4484.1(13)	7013.2(12)	3111.6(9)	14.9(3)
O2	6741.9(14)	5451.8(14)	3171.2(10)	22.8(3)
O3	5866.2(15)	3412.6(14)	4254.5(10)	25.3(4)
O4	1850.2(13)	3368.6(12)	2059.6(9)	18.2(3)
O5	3118.4(14)	4335.6(13)	4020.5(10)	20.3(3)
C1	2761.9(18)	5214.9(18)	3641.1(13)	14.3(4)
C2	1088(2)	7494(2)	4533.1(15)	22.1(5)
C3	4565(2)	9376(2)	3418.5(15)	24.3(5)
C4	3885(2)	4490.3(19)	2009.7(14)	18.0(4)
C5	4166(2)	4394(2)	1044.0(15)	25.9(5)
C6	5816(2)	8296(2)	1920.0(14)	22.6(4)
C7	730(2)	8710(2)	4572.2(18)	32.6(6)
C8	6003.5(19)	5209.8(19)	3684.6(14)	17.9(4)
C9	2740(2)	8259(2)	1713.0(14)	23.2(4)
C10	2336.9(19)	4462.7(18)	1967.1(13)	15.7(4)
C11	-74(2)	1632(2)	1664.4(17)	29.8(5)
C12	5480(2)	4014.3(19)	3597.0(15)	18.9(4)
C13	7006(2)	6645(2)	4861.0(16)	24.4(5)
C14	4516.9(19)	6752.1(18)	4053.8(13)	15.0(4)
C15	-1320(3)	2359(3)	1664(2)	47.9(8)
C16	548(2)	6918.6(19)	3620.3(15)	19.1(4)
C17	106(2)	5724.7(19)	3553.0(15)	20.4(4)
C18	3130.1(19)	6297.1(17)	4167.0(13)	14.5(4)
C19	5675(2)	9386(2)	1376.0(17)	31.5(6)
C20	397(2)	5122(2)	2708.4(15)	19.5(4)

Table 2 Fractional Atomic Coordinates (×10^4) and Equivalent Isotropic Displacement Parameters (Å^2×10^3) for ca260221_1_1. U_{eq} is defined as 1/3 of of the trace of the orthogonalised U_{IJ} tensor.

Atom	*x*	*y*	*z*	U(eq)
C21	5724(3)	7322(2)	1253.0(17)	35.0(6)
C22	5733.7(19)	5961.1(19)	4434.1(14)	18.0(4)
C23	609(3)	3077(2)	111.9(16)	31.6(5)
C24	2056.1(19)	7201.4(18)	3921.7(14)	16.9(4)
C25	1171(2)	6898(2)	5436.8(16)	30.7(6)
C26	4714(2)	3612.3(19)	2663.2(14)	21.5(4)
C27	7219(2)	8270(3)	2589.0(18)	34.5(6)
C28	1909.9(18)	5264.6(18)	2657.0(13)	15.1(4)
C29	-508(3)	638(3)	1030(2)	47.7(8)
C30	-568(2)	5500(2)	1811.9(16)	29.2(5)
C31	492(3)	1200(3)	2638(2)	47.3(8)
C32	2683(2)	1456(2)	1158.8(17)	27.4(5)

Table 3 Anisotropic Displacement Parameters (Å^2×10^3) for ca260221_1_1. The Anisotropic displacement factor exponent takes the form: -2π^2[h^{2}a*^{2}U$_{11}$+2hka*b*U$_{12}$+…].

Atom	U_{11}	U_{22}	U_{33}	U_{23}	U_{13}	U_{12}
Si1	14.7(2)	15.2(3)	18.5(3)	2.0(2)	4.79(19)	-0.4(2)
Si2	21.6(3)	13.7(3)	20.1(3)	-1.5(2)	2.8(2)	-0.8(2)
O1	13.7(6)	16.4(8)	15.7(6)	1.3(6)	5.5(5)	0.2(5)
O2	12.5(7)	28.2(10)	29.0(8)	1.4(7)	7.6(6)	0.4(6)
O3	22.6(7)	22.9(9)	29.5(8)	7.6(7)	4.1(6)	4.6(7)
O4	19.8(7)	12.8(8)	21.8(7)	-1.0(6)	4.3(5)	-2.8(6)
O5	19.5(7)	17.9(8)	23.5(7)	3.0(6)	4.8(6)	0.3(6)
C1	9.7(8)	16.2(10)	18.9(9)	0.3(8)	6.9(7)	-0.6(8)
C2	14.5(9)	26.8(12)	27.3(11)	-7.5(10)	9.4(8)	-2.6(9)
C3	30.2(11)	17.6(12)	25.5(11)	-0.7(9)	6.8(9)	-3.5(10)
C4	16.5(9)	17.4(11)	20.3(10)	-0.8(9)	4.6(8)	-1.0(8)
C5	25.2(11)	27.6(13)	27.7(11)	-3.1(10)	11.7(9)	-2.4(10)
C6	20.5(10)	24.6(12)	24.8(10)	7.5(10)	9.5(8)	1.2(10)
C7	20.9(11)	31.2(15)	47.0(14)	-17.0(12)	10.5(10)	2.0(10)
C8	10.5(8)	19.8(11)	21.0(10)	4.3(9)	-1.5(7)	3.0(8)
C9	20.0(9)	23.9(12)	24.1(10)	4.5(10)	1.6(8)	-0.5(10)
C10	16.3(9)	12.7(10)	17.5(9)	0.1(8)	2.6(7)	-1.0(8)
C11	29.9(12)	22.9(13)	39.0(14)	-8.6(11)	12.6(10)	-10.2(10)
C12	11.6(9)	19.6(11)	27.0(11)	1.1(9)	7.6(8)	4.3(8)
C13	14.6(10)	26.5(13)	29.1(12)	-1.7(10)	-1.3(8)	-0.3(9)
C14	13.5(9)	14.7(10)	16.7(9)	1.7(8)	3.0(7)	-1.5(8)
C15	26.2(12)	39.0(18)	81(2)	-16.8(17)	18.1(13)	-12.1(13)
C16	12.0(9)	20.2(11)	26.2(11)	-2.3(9)	6.6(8)	1.5(8)

Table 3 Anisotropic Displacement Parameters (Å^2×10^3) for ca260221_1_1. The Anisotropic displacement factor exponent takes the form: -2π^2[h^{2}a*^{2}U$_{11}$+2hka*b*U$_{12}$+…].

Atom	U_{11}	U_{22}	U_{33}	U_{23}	U_{13}	U_{12}
C17	11.8(9)	22.9(12)	27.7(11)	-1.3(9)	6.9(8)	-1.9(8)
C18	12.1(9)	16.5(10)	16.0(9)	0.7(8)	5.0(7)	-0.9(8)
C19	29.9(12)	32.9(15)	35.5(13)	14.9(12)	15.3(10)	1.2(11)
C20	11.7(9)	18.9(11)	27.1(11)	-1.8(9)	2.6(8)	-1.8(8)
C21	38.6(13)	36.0(16)	36.9(13)	1.1(12)	22.3(11)	3.8(12)
C22	13.5(9)	20.3(11)	19.3(10)	3.7(9)	2.0(7)	0.7(8)
C23	40.5(13)	24.8(14)	24.8(11)	-0.4(10)	-2.9(10)	-0.2(11)
C24	13.1(9)	16.6(11)	22.6(10)	0.3(8)	7.0(7)	-0.7(8)
C25	24.2(11)	43.8(16)	28.4(12)	-7.7(11)	14.8(9)	-6.1(11)
C26	18.2(10)	17.4(11)	28.5(11)	-3.5(9)	4.5(8)	3.6(8)
C27	17.9(10)	44.6(16)	42.8(13)	15.4(13)	10.5(9)	1.5(11)
C28	12.3(8)	13.3(10)	19.1(9)	1.0(8)	2.3(7)	-1.1(8)
C29	48.4(16)	36.1(18)	64.5(19)	-22.4(15)	24.8(15)	-23.3(14)
C30	18.8(10)	35.1(15)	30.3(12)	-5.4(11)	-1.7(9)	5.6(10)
C31	58.8(19)	45(2)	46.1(17)	9.0(14)	28.8(15)	-9.2(15)
C32	32.2(12)	19.1(12)	31.9(12)	-0.9(10)	9.5(10)	5.0(9)

Table 4 Bond Lengths for ca260221_1_1.

Atom	Atom	Length/Å	Atom	Atom	Length/Å
Si1	O1	1.6592(16)	C4	C26	1.550(3)
Si1	C3	1.862(2)	C6	C19	1.535(3)
Si1	C6	1.892(2)	C6	C21	1.528(4)
Si1	C9	1.874(2)	C6	C27	1.537(3)
Si2	O4	1.6487(15)	C8	C12	1.534(3)
Si2	C11	1.882(2)	C8	C22	1.508(3)
Si2	C23	1.863(2)	C10	C28	1.540(3)
Si2	C32	1.868(2)	C11	C15	1.535(4)
O1	C14	1.427(2)	C11	C29	1.529(4)
O2	C8	1.216(3)	C11	C31	1.522(4)
O3	C12	1.210(3)	C12	C26	1.508(3)
O4	C10	1.428(3)	C13	C22	1.539(3)
O5	C1	1.219(3)	C14	C18	1.549(3)
C1	C18	1.526(3)	C14	C22	1.560(3)
C1	C28	1.521(3)	C16	C17	1.507(3)
C2	C7	1.518(4)	C16	C24	1.527(3)
C2	C16	1.512(3)	C17	C20	1.535(3)
C2	C24	1.518(3)	C18	C24	1.525(3)
C2	C25	1.509(3)	C20	C28	1.557(3)
C4	C5	1.527(3)	C20	C30	1.533(3)
C4	C10	1.551(3)			

Table 5 Bond Angles for ca260221_1_1.

Atom	Atom	Atom	Angle/°	Atom	Atom	Atom	Angle/°
O1	Si1	C3	110.24(9)	O4	C10	C4	112.44(17)
O1	Si1	C6	109.40(9)	O4	C10	C28	111.40(16)
O1	Si1	C9	106.60(9)	C28	C10	C4	112.93(16)
C3	Si1	C6	110.55(11)	C15	C11	Si2	111.42(19)
C3	Si1	C9	111.53(11)	C29	C11	Si2	109.55(17)
C9	Si1	C6	108.41(10)	C29	C11	C15	109.1(2)
O4	Si2	C11	106.41(9)	C31	C11	Si2	109.38(18)
O4	Si2	C23	111.39(10)	C31	C11	C15	109.3(2)
O4	Si2	C32	110.31(10)	C31	C11	C29	108.1(3)
C23	Si2	C11	111.22(12)	O3	C12	C8	117.07(19)
C23	Si2	C32	108.62(11)	O3	C12	C26	123.7(2)
C32	Si2	C11	108.85(12)	C26	C12	C8	118.51(18)
C14	O1	Si1	131.05(13)	O1	C14	C18	111.24(15)
C10	O4	Si2	129.79(12)	O1	C14	C22	109.48(15)
O5	C1	C18	119.88(17)	C18	C14	C22	113.68(17)
O5	C1	C28	121.54(19)	C2	C16	C24	59.91(13)
C28	C1	C18	118.55(17)	C17	C16	C2	122.8(2)
C16	C2	C7	116.0(2)	C17	C16	C24	119.66(18)
C16	C2	C24	60.54(13)	C16	C17	C20	113.61(17)
C24	C2	C7	116.1(2)	C1	C18	C14	111.80(15)
C25	C2	C7	113.3(2)	C24	C18	C1	114.42(16)
C25	C2	C16	120.9(2)	C24	C18	C14	109.39(17)
C25	C2	C24	120.4(2)	C17	C20	C28	110.96(16)
C5	C4	C10	111.01(16)	C30	C20	C17	111.51(18)
C5	C4	C26	110.33(18)	C30	C20	C28	111.36(18)
C26	C4	C10	114.12(17)	C8	C22	C13	110.77(16)
C19	C6	Si1	108.57(15)	C8	C22	C14	111.13(16)
C19	C6	C27	108.7(2)	C13	C22	C14	109.71(18)
C21	C6	Si1	110.53(16)	C2	C24	C16	59.55(13)
C21	C6	C19	109.60(19)	C2	C24	C18	122.91(18)
C21	C6	C27	108.4(2)	C18	C24	C16	121.27(18)
C27	C6	Si1	110.95(15)	C12	C26	C4	116.63(18)
O2	C8	C12	114.95(19)	C1	C28	C10	115.54(16)
O2	C8	C22	123.8(2)	C1	C28	C20	107.37(15)
C22	C8	C12	120.92(18)	C10	C28	C20	112.79(16)

Table 6 Torsion Angles for ca260221_1_1.

A	B	C	D	Angle/°	A	B	C	D	Angle/°
Si1	O1	C14	C18	105.14(18)	C9	Si1	C6	C19	60.50(19)

Table 6 Torsion Angles for ca260221_1_1.

A	B	C	D	Angle/˚	A	B	C	D	Angle/˚
Si1	O1	C14	C22	-128.38(16)	C9	Si1	C6	C21	-59.75(19)
Si2	O4	C10	C4	-88.54(19)	C9	Si1	C6	C27	179.92(19)
Si2	O4	C10	C28	143.54(14)	C10	C4	C26	C12	-101.7(2)
O1	Si1	C6	C19	176.39(15)	C11	Si2	O4	C10	-146.52(17)
O1	Si1	C6	C21	56.13(18)	C12	C8	C22	C13	138.23(19)
O1	Si1	C6	C27	-64.2(2)	C12	C8	C22	C14	-99.5(2)
O1	C14	C18	C1	62.8(2)	C14	C18	C24	C2	-131.19(19)
O1	C14	C18	C24	-65.0(2)	C14	C18	C24	C16	157.06(17)
O1	C14	C22	C8	-32.4(2)	C16	C2	C24	C18	-109.7(2)
O1	C14	C22	C13	90.4(2)	C16	C17	C20	C28	52.1(2)
O2	C8	C12	O3	113.8(2)	C16	C17	C20	C30	-72.7(2)
O2	C8	C12	C26	-56.5(2)	C17	C16	C24	C2	-113.0(2)
O2	C8	C22	C13	-34.5(3)	C17	C16	C24	C18	-0.6(3)
O2	C8	C22	C14	87.8(2)	C17	C20	C28	C1	38.1(2)
O3	C12	C26	C4	160.01(19)	C17	C20	C28	C10	166.49(18)
O4	Si2	C11	C15	67.2(2)	C18	C1	C28	C10	139.31(18)
O4	Si2	C11	C29	-172.03(19)	C18	C1	C28	C20	-93.87(19)
O4	Si2	C11	C31	-53.7(2)	C18	C14	C22	C8	92.6(2)
O4	C10	C28	C1	79.5(2)	C18	C14	C22	C13	-144.53(17)
O4	C10	C28	C20	-44.6(2)	C22	C8	C12	O3	-59.4(2)
O5	C1	C18	C14	89.6(2)	C22	C8	C12	C26	130.20(19)
O5	C1	C18	C24	-145.38(18)	C22	C14	C18	C1	-61.4(2)
O5	C1	C28	C10	-42.8(2)	C22	C14	C18	C24	170.86(17)
O5	C1	C28	C20	84.1(2)	C23	Si2	O4	C10	-25.15(19)
C1	C18	C24	C2	102.5(2)	C23	Si2	C11	C15	-54.3(2)
C1	C18	C24	C16	30.8(3)	C23	Si2	C11	C29	66.5(2)
C2	C16	C17	C20	-145.77(18)	C23	Si2	C11	C31	-175.23(19)
C2	C16	C24	C18	112.4(2)	C24	C2	C16	C17	107.9(2)
C3	Si1	O1	C14	6.34(18)	C24	C16	C17	C20	-74.4(2)
C3	Si1	C6	C19	-62.03(18)	C25	C2	C16	C17	-1.9(3)
C3	Si1	C6	C21	177.72(16)	C25	C2	C16	C24	-109.8(2)
C3	Si1	C6	C27	57.4(2)	C25	C2	C24	C16	110.7(2)
C4	C10	C28	C1	-48.2(2)	C25	C2	C24	C18	1.0(3)
C4	C10	C28	C20	-172.20(17)	C26	C4	C10	O4	-33.8(2)
C5	C4	C10	O4	91.6(2)	C26	C4	C10	C28	93.3(2)
C5	C4	C10	C28	-141.31(19)	C28	C1	C18	C14	-92.45(19)
C5	C4	C26	C12	132.50(19)	C28	C1	C18	C24	32.6(2)

Table 6 Torsion Angles for ca260221_1_1.

A	B	C	D	Angle/°	A	B	C	D	Angle/°
C6	Si1	O1	C14	128.11(16)	C30	C20	C28	C1	162.91(19)
C7	C2	C16	C17	-145.5(2)	C30	C20	C28	C10	-68.7(2)
C7	C2	C16	C24	106.7(2)	C32	Si2	O4	C10	95.56(18)
C7	C2	C24	C16	-106.5(2)	C32	Si2	C11	C15	-173.96(18)
C7	C2	C24	C18	143.8(2)	C32	Si2	C11	C29	-53.2(2)
C8	C12	C26	C4	-30.3(3)	C32	Si2	C11	C31	65.1(2)
C9	Si1	O1	C14	-114.86(16)					

Table 7 Hydrogen Atom Coordinates (Å×10^4) and Isotropic Displacement Parameters (Å^2×10^3) for ca260221_1_1.

Atom	*x*	*y*	*z*	U(eq)
H3A	3739.06	9414.21	3660.07	37
H3B	4684.16	10075.37	3111.97	37
H3C	5351.4	9247.3	3929.21	37
H4	4223.31	5232.52	2256.9	22
H5A	3887.48	3661.11	788.99	39
H5B	5139.67	4496.35	1081.17	39
H5C	3652.79	4964.2	642.28	39
H7A	1399.73	9075.06	5061.8	49
H7B	-175.19	8780.67	4703.02	49
H7C	733.08	9059.08	3976.96	49
H9A	2619.33	7572.8	1352.3	35
H9B	2722.49	8891.32	1297.3	35
H9C	2002.77	8332.94	2040.33	35
H10	1876.03	4723.65	1335.36	19
H13A	7759.01	6144.6	5119.11	37
H13B	6814.12	7116.64	5353.16	37
H13C	7253.28	7107.55	4382.41	37
H14	4690.81	7456.56	4414.18	18
H15A	-1051.38	2989.34	2079.93	72
H15B	-2009.28	1921.91	1875.91	72
H15C	-1695.87	2628.92	1036.41	72
H16	20.51	7406.9	3123.31	23
H17A	-881.74	5690.94	3525.53	24
H17B	577.82	5332.56	4119.84	24
H18	3228.16	6126.78	4837.9	17
H19A	6385.57	9429.28	1023.6	47
H19B	5767.91	10010.72	1806.84	47
H19C	4780.73	9415.09	950.14	47
H20	234.44	4314.67	2786.09	23
H21A	4861.15	7357.35	793.23	52

Table 7 Hydrogen Atom Coordinates (Å×10^4) and Isotropic Displacement Parameters (Å^2×10^3) for ca260221_1_1.

Atom	*x*	*y*	*z*	U(eq)
H21B	5771.69	6626.69	1598.31	52
H21C	6479.71	7357.2	939.74	52
H22	5497.92	5492.37	4930.66	22
H23A	-90.72	3619.93	163.71	47
H23B	212.45	2504.7	-335.15	47
H23C	1348.84	3448.23	-97.33	47
H24	2341.18	7847.23	3589.09	20
H25A	1375.36	6114.51	5363.08	46
H25B	300.67	6963.12	5619.49	46
H25C	1891.63	7227.62	5914.75	46
H26A	4082.78	3021.14	2761.63	26
H26B	5374.71	3273.24	2344.06	26
H27A	7346.31	7556.87	2910.27	52
H27B	7278.04	8868.14	3042.24	52
H27C	7928.1	8369.71	2240.71	52
H28	2004.47	6030.55	2424.7	18
H29A	-899.65	898.6	401.09	72
H29B	-1188.42	206.75	1255.88	72
H29C	284.24	172.67	1025.1	72
H30A	-426.54	6289.31	1714.56	44
H30B	-1509.64	5377.2	1857	44
H30C	-384.22	5075.81	1290.74	44
H31A	1237.91	684.11	2629.89	71
H31B	-227.47	816.81	2860.15	71
H31C	830.77	1821.35	3049.24	71
H32A	3436.54	1874.06	1008.5	41
H32B	2343.36	917.59	667.75	41
H32C	3002.33	1066.34	1746.32	41

Experimental

Single crystals of $C_{32}H_{58}O_5Si_2$ **[ca260221_1_1]** were **[]**. A suitable crystal was selected and **[]** on a **Bruker APEX-II Duo (Mo)** diffractometer. The crystal was kept at 100.0(1) K during data collection. Using Olex2 [1], the structure was solved with the SHELXT [2] structure solution program using Intrinsic Phasing and refined with the SHELXL [3] refinement package using Least Squares minimisation.

1. Dolomanov, O.V., Bourhis, L.J., Gildea, R.J, Howard, J.A.K. & Puschmann, H. (2009), J. Appl. Cryst. 42, 339-341.
2. Sheldrick, G.M. (2015). Acta Cryst. A71, 3-8.
3. Sheldrick, G.M. (2015). Acta Cryst. C71, 3-8.

Crystal structure determination of [ca260221_1_1]

Crystal Data for $C_{32}H_{58}O_5Si_2$ (M =578.96 g/mol): monoclinic, space group $P2_1$ (no. 4), a = 10.102(3) Å, b = 12.087(4) Å, c = 14.846(4) Å, β = 102.971(7)°, V = 1766.5(8) Å^3, Z = 2, T = 100.0(1) K, μ(MoKα) = 0.134 mm^{-1}, $Dcalc$ = 1.088 g/cm^3, 28705 reflections measured (4.138° ≤ 2Θ ≤ 54.956°), 6281 unique (R_{int} = 0.0325, R_{sigma} = 0.0283) which were used in all calculations. The final R_1 was 0.0307 (I > 2σ(I)) and wR_2 was 0.0758 (all data).

Refinement model description

Number of restraints - 1, number of constraints - unknown.

Details:

```
1. Fixed Uiso
 At 1.2 times of:
  All C(H) groups, All C(H,H) groups
 At 1.5 times of:
  All C(H,H,H) groups
2.a Ternary CH refined with riding coordinates:
 C4(H4), C10(H10), C14(H14), C16(H16), C18(H18), C20(H20), C22(H22), C24(H24),
 C28(H28)
2.b Secondary CH2 refined with riding coordinates:
 C17(H17A,H17B), C26(H26A,H26B)
2.c Idealised Me refined as rotating group:
 C3(H3A,H3B,H3C), C5(H5A,H5B,H5C), C7(H7A,H7B,H7C), C9(H9A,H9B,H9C), C13(H13A,
 H13B,H13C), C15(H15A,H15B,H15C), C19(H19A,H19B,H19C), C21(H21A,H21B,H21C),
 C23(H23A,H23B,H23C), C25(H25A,H25B,H25C), C27(H27A,H27B,H27C), C29(H29A,H29B,
 H29C), C30(H30A,H30B,H30C), C31(H31A,H31B,H31C), C32(H32A,H32B,H32C)
```

Rearrangement product γ-lactone **10**:

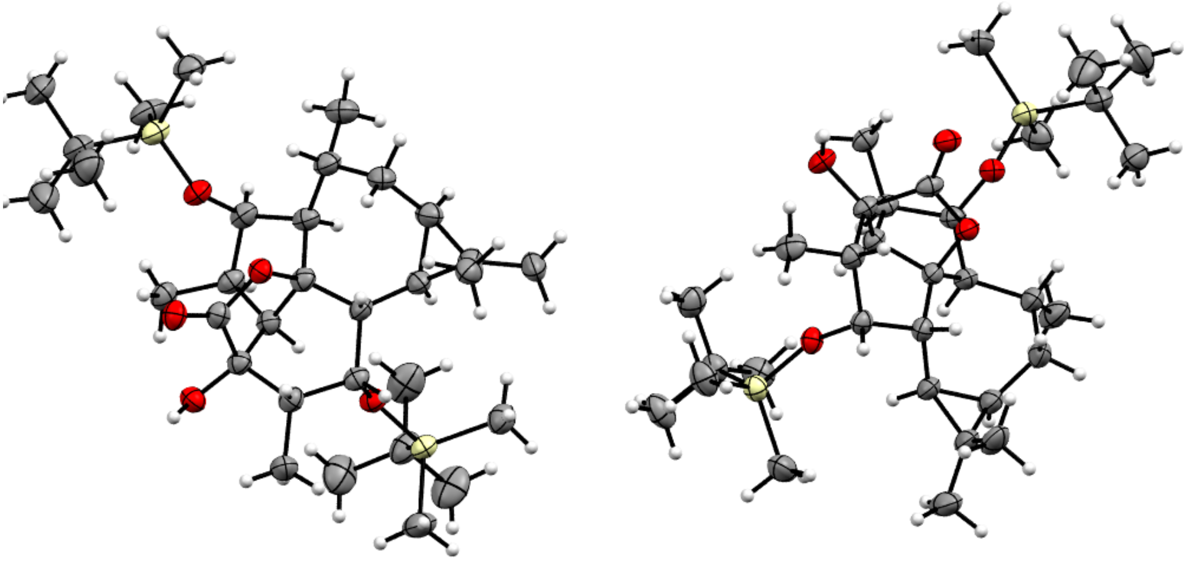

Table 1 Crystal data and structure refinement for ca080923_1_1.

Identification code	ca080923_1_1
Empirical formula	$C_{32}H_{58}O_5Si_2$
Formula weight	578.96
Temperature/K	100.0(1)
Crystal system	monoclinic
Space group	C2
a/Å	32.1203(10)
b/Å	7.2454(2)
c/Å	33.1269(12)
α/°	90
β/°	116.597(4)
γ/°	90
Volume/Å^3	6893.6(4)
Z	8
ρ_{calc}g/cm^3	1.116
μ/mm^{-1}	1.204
F(000)	2544.0
Crystal size/mm^3	0.233 × 0.046 × 0.019
Radiation	Cu Kα (λ = 1.54184)

2Θ range for data collection/°	7.954 to 161.868
Index ranges	-38 ≤ h ≤ 40, -8 ≤ k ≤ 9, -39 ≤ l ≤ 42
Reflections collected	43812
Independent reflections	13239 [R_{int} = 0.0713, R_{sigma} = 0.0671]
Data/restraints/parameters	13239/3/739
Goodness-of-fit on F^2	1.087
Final R indexes [I>=2σ (I)]	R_1 = 0.0530, wR_2 = 0.1276
Final R indexes [all data]	R_1 = 0.0848, wR_2 = 0.1485
Largest diff. peak/hole / e Å^{-3}	0.26/-0.48
Flack parameter	-0.05(2)

Table 2 Fractional Atomic Coordinates (×10^4) and Equivalent Isotropic Displacement Parameters (Å^2×10^3) for ca080923_1_1. U_{eq} is defined as 1/3 of the trace of the orthogonalised U_{IJ} tensor.

Atom	*x*	*y*	*z*	U(eq)
Si1A	4049.7(4)	6076(2)	5685.6(5)	36.9(3)
Si2A	3316.4(4)	9611.2(18)	2820.9(4)	32.6(3)
O1A	3850.3(10)	7193(5)	5199.6(10)	33.1(7)
O2A	3781.6(10)	10404(4)	4731.4(10)	31.1(7)
O3A	4540.0(10)	10957(5)	5143.1(11)	36.7(8)
O4A	3475.3(10)	9768(4)	3360.3(10)	29.8(7)
O5A	4664.9(10)	8847(5)	4450.0(11)	33.5(7)
C1A	4034.4(16)	7777(9)	6109.7(18)	45.7(13)
C2A	4418(2)	9215(11)	6225(3)	76(2)
C3A	4102(3)	6705(12)	6543(2)	71(2)
C4A	3567.0(18)	8775(9)	5938.8(19)	48.8(14)
C5A	3676.7(19)	4021(9)	5627(2)	50.3(14)
C6A	4663.2(19)	5272(10)	5887.7(19)	53.4(16)
C7A	3595.4(15)	6516(7)	4748.6(16)	32.6(11)
C8A	3901.2(14)	6319(7)	4499.8(16)	30.1(10)
C9A	4390.9(15)	5590(7)	4789.5(17)	32.7(11)
C10A	3848.5(14)	8133(6)	4240.7(16)	27.8(10)
C11A	3491.3(14)	9304(6)	4316.2(14)	28.2(10)
C12A	3218.4(14)	7916(7)	4446.8(15)	30.9(10)
C13A	4236.7(14)	9589(6)	4383.7(15)	28.3(9)
C14A	4224.7(14)	10355(6)	4802.1(16)	30.0(10)
C15A	4065.7(15)	11261(7)	4032.6(16)	31.7(10)
C16A	4371.8(16)	11502(8)	3794.1(18)	41.1(12)
C17A	3543.3(14)	11142(7)	3693.7(15)	29.2(10)
C18A	3241.9(14)	10734(7)	3938.5(16)	30.0(10)
C19A	2745.5(15)	10164(7)	3629.5(17)	33.0(11)
C20A	2338.0(16)	11504(8)	3497.4(17)	39.7(12)

Table 2 Fractional Atomic Coordinates (×10⁴) and Equivalent Isotropic Displacement Parameters (Å²×10³) for ca080923_1_1. U_{eq} is defined as 1/3 of the trace of the orthogonalised U_{IJ} tensor.

Atom	*x*	*y*	*z*	U(eq)
C21A	2414.0(16)	10004(8)	3842.8(17)	38.7(12)
C22A	1930.7(17)	11199(10)	3037.6(19)	52.8(15)
C23A	2411.7(19)	13509(8)	3626(2)	49.9(15)
C24A	2605.0(17)	10318(8)	4344.9(17)	40.0(12)
C25A	2893.1(16)	8683(8)	4633.9(17)	37.9(12)
C26A	2577.2(18)	7138(9)	4659(2)	52.4(16)
C27A	3396.7(17)	7079(7)	2757.8(17)	37.1(11)
C28A	3237(2)	6594(9)	2257.8(19)	54.1(15)
C29A	3914.5(18)	6570(8)	3034.2(19)	46.4(13)
C30A	3110.0(19)	5945(8)	2938.1(19)	45.4(13)
C31A	3685(2)	10978(9)	2629(2)	49.3(14)
C32A	2701.7(19)	10345(9)	2480.0(19)	50.7(15)
Si1B	6435.7(4)	-967(2)	1532.1(5)	39.4(3)
Si2B	3489.0(4)	2612.9(19)	956.4(5)	34.2(3)
O1B	5928.2(10)	40(5)	1376.7(12)	37.5(8)
O2B	5409.5(10)	3312(5)	1204.3(11)	31.9(7)
O3B	5429.3(11)	3744(5)	544.3(11)	36.7(8)
O4B	4013.5(10)	2649(5)	986.9(10)	33.0(7)
O5B	4572.1(11)	1592(5)	151.7(11)	35.5(8)
C1B	6631.7(17)	-524(9)	1079.9(19)	50.5(14)
C2B	6315(2)	-1531(14)	637(2)	77(2)
C3B	7129.9(19)	-1277(12)	1240(2)	65.8(19)
C4B	6633(2)	1544(11)	994(3)	72(2)
C5B	6871.0(17)	111(9)	2065(2)	50.5(15)
C6B	6392.1(18)	-3469(8)	1629(2)	52.9(15)
C7B	5550.3(15)	-522(7)	1465.7(17)	36.3(11)
C8B	5096.3(15)	-778(7)	1024.2(16)	34.2(11)
C9B	5168.4(17)	-1513(7)	628.8(17)	37.7(12)
C10B	4814.3(15)	1031(7)	955.4(15)	30.6(10)
C11B	5106.1(14)	2269(7)	1359.3(16)	30.3(10)
C12B	5424.2(15)	964(7)	1728.7(16)	33.0(11)
C13B	4741.2(14)	2394(7)	579.8(16)	30.5(10)
C14B	5227.3(16)	3199(7)	751.4(16)	31.9(10)
C15B	4430.0(15)	4064(7)	602.3(16)	34.1(10)
C16B	3970.9(17)	4235(8)	171.3(17)	41.6(12)
C17B	4346.2(14)	4040(7)	1028.6(15)	30.9(10)
C18B	4811.7(14)	3729(7)	1455.4(15)	31.1(10)
C19B	4763.9(15)	3344(7)	1879.6(16)	33.6(11)
C20B	4855.6(16)	4809(8)	2240.2(17)	39.9(12)
C21B	5211.5(16)	3287(8)	2321.4(17)	38.5(12)
C22B	4576.0(18)	4649(10)	2504.8(18)	49.7(14)
C23B	4955.5(18)	6781(8)	2169.9(19)	43.8(13)

Table 2 Fractional Atomic Coordinates (×10⁴) and Equivalent Isotropic Displacement Parameters (Å²×10³) for ca080923_1_1. U_{eq} is defined as 1/3 of the trace of the orthogonalised U_{IJ} tensor.

Atom	*x*	*y*	*z*	U(eq)
C24B	5670.5(16)	3564(8)	2308.7(17)	38.7(12)
C25B	5824.5(16)	1818(7)	2145.9(17)	37.6(12)
C26B	6030.4(18)	375(9)	2524.5(19)	47.7(14)
C27B	3338.2(18)	96(8)	872(2)	49.2(14)
C28B	2860(2)	-265(10)	861(3)	72(2)
C29B	3325(2)	-510(10)	414(2)	68.5(19)
C30B	3713(2)	-1014(9)	1259(2)	63.7(17)
C31B	3053.1(18)	3976(10)	478(2)	55.9(16)
C32B	3490.9(18)	3520(9)	1479.0(19)	46.0(13)

Table 3 Anisotropic Displacement Parameters (Å²×10³) for ca080923_1_1. The Anisotropic displacement factor exponent takes the form: -$2\pi^2[h^2a^{*2}U_{11}+2hka^*b^*U_{12}+\dots]$.

Atom	U_{11}	U_{22}	U_{33}	U_{23}	U_{13}	U_{12}
Si1A	30.8(6)	44.1(8)	34.5(7)	4.8(6)	13.5(5)	6.5(6)
Si2A	37.0(7)	30.4(7)	32.0(7)	0.1(6)	16.9(5)	0.9(5)
O1A	29.0(15)	38.5(19)	29.8(17)	1.6(14)	11.3(13)	3.0(13)
O2A	26.3(15)	33.8(18)	29.9(17)	-5.6(14)	9.8(13)	2.4(13)
O3A	29.4(16)	35.6(19)	36.5(19)	-3.8(15)	7.1(14)	4.5(14)
O4A	33.2(15)	25.8(16)	34.0(17)	-4.4(14)	18.2(13)	-1.9(13)
O5A	23.7(15)	31.9(18)	44.5(19)	1.5(15)	14.8(14)	3.0(13)
C1A	32(3)	64(4)	37(3)	1(3)	13(2)	5(3)
C2A	49(3)	83(6)	92(5)	-38(5)	27(3)	-15(4)
C3A	86(5)	84(6)	44(4)	5(4)	30(3)	27(4)
C4A	41(3)	54(4)	47(3)	-5(3)	16(2)	7(3)
C5A	52(3)	52(3)	47(3)	5(3)	22(3)	-3(3)
C6A	47(3)	70(4)	38(3)	9(3)	15(2)	21(3)
C7A	26(2)	36(3)	32(3)	1(2)	9.7(19)	0.1(19)
C8A	25(2)	28(2)	35(3)	-3(2)	10.9(19)	-1.4(18)
C9A	29(2)	27(3)	40(3)	2(2)	15(2)	3.1(18)
C10A	25(2)	27(2)	32(2)	-2.7(19)	12.8(19)	-0.7(17)
C11A	24(2)	32(3)	28(2)	-3.1(19)	11.1(18)	1.4(18)
C12A	24(2)	38(3)	30(2)	4(2)	10.7(18)	5.1(19)
C13A	20.0(19)	27(2)	36(2)	-1(2)	10.6(18)	0.1(17)
C14A	25(2)	23(2)	36(3)	0(2)	8(2)	0.8(18)
C15A	32(2)	25(2)	36(3)	-3(2)	15(2)	0.7(19)
C16A	33(2)	41(3)	49(3)	5(2)	18(2)	-7(2)
C17A	31(2)	26(2)	32(2)	-2(2)	14.9(19)	3.0(18)
C18A	29(2)	32(3)	31(2)	-2.3(19)	14.8(19)	3.0(18)
C19A	24(2)	40(3)	35(3)	3(2)	14.0(19)	4.7(19)

Table 3 Anisotropic Displacement Parameters (Å^2×10^3) for ca080923_1_1. The Anisotropic displacement factor exponent takes the form: -2π^2[h^{2}a*^{2}U$_{11}$+2hka*b*U$_{12}$+…].

Atom	U$_{11}$	U$_{22}$	U$_{33}$	U$_{23}$	U$_{13}$	U$_{12}$
C20A	28(2)	54(3)	38(3)	12(3)	17(2)	12(2)
C21A	28(2)	51(3)	39(3)	9(2)	17(2)	6(2)
C22A	30(2)	77(4)	48(3)	17(3)	14(2)	9(3)
C23A	44(3)	52(4)	60(4)	16(3)	29(3)	24(3)
C24A	35(2)	54(3)	39(3)	8(2)	23(2)	11(2)
C25A	29(2)	54(3)	35(3)	6(2)	17(2)	8(2)
C26A	36(3)	68(4)	60(4)	26(3)	28(3)	13(3)
C27A	45(3)	34(3)	35(3)	-1(2)	20(2)	-1(2)
C28A	76(4)	45(4)	41(3)	-11(3)	26(3)	-1(3)
C29A	50(3)	38(3)	54(3)	-2(3)	26(3)	9(2)
C30A	52(3)	33(3)	48(3)	-2(2)	19(3)	-8(2)
C31A	68(4)	42(3)	45(3)	1(3)	32(3)	-7(3)
C32A	54(3)	57(4)	34(3)	3(3)	14(3)	12(3)
Si1B	27.3(6)	40.1(8)	45.8(8)	-1.4(6)	11.9(6)	2.3(6)
Si2B	25.9(6)	37.7(7)	37.9(7)	0.4(6)	13.1(5)	0.7(5)
O1B	28.6(16)	39(2)	45(2)	1.9(16)	17.0(15)	1.4(14)
O2B	30.0(16)	34.3(18)	33.4(18)	0.5(14)	16.0(14)	-3.2(13)
O3B	37.5(17)	36.4(19)	43.8(19)	-1.8(16)	25.0(15)	-4.2(14)
O4B	27.7(15)	35.0(18)	35.7(18)	1.9(15)	13.6(13)	-1.8(14)
O5B	36.3(17)	39(2)	29.8(18)	-1.9(15)	13.2(14)	-2.8(14)
C1B	33(3)	66(4)	50(3)	7(3)	16(2)	11(3)
C2B	47(3)	129(7)	55(4)	-20(4)	23(3)	-1(4)
C3B	38(3)	99(6)	62(4)	-3(4)	24(3)	14(3)
C4B	63(4)	88(6)	81(5)	25(4)	46(4)	12(4)
C5B	31(3)	59(4)	53(3)	-2(3)	11(2)	2(2)
C6B	34(3)	42(3)	70(4)	-2(3)	11(3)	8(2)
C7B	32(2)	35(3)	44(3)	6(2)	18(2)	3(2)
C8B	30(2)	29(3)	43(3)	2(2)	16(2)	0.6(19)
C9B	38(3)	31(3)	45(3)	-4(2)	19(2)	3(2)
C10B	30(2)	31(2)	32(2)	2(2)	14.9(19)	-1.1(19)
C11B	26(2)	33(3)	35(3)	3(2)	15.2(19)	-1.8(18)
C12B	26(2)	34(3)	36(3)	5(2)	11.4(19)	1.0(19)
C13B	27(2)	33(3)	33(3)	1(2)	14.1(19)	-1.4(19)
C14B	34(2)	29(2)	36(3)	-1(2)	18(2)	-0.5(19)
C15B	33(2)	33(3)	37(3)	1(2)	17(2)	0(2)
C16B	37(2)	48(3)	37(3)	5(2)	15(2)	9(2)
C17B	26(2)	29(2)	37(3)	1(2)	13.9(19)	-3.1(18)
C18B	25(2)	36(3)	35(3)	1(2)	14.8(19)	1.7(18)
C19B	27(2)	40(3)	33(3)	0(2)	13(2)	-1.9(19)
C20B	32(2)	51(3)	38(3)	-6(3)	17(2)	-1(2)
C21B	33(2)	46(3)	33(3)	2(2)	11(2)	-1(2)
C22B	42(3)	75(4)	35(3)	-10(3)	20(2)	-4(3)

Table 3 Anisotropic Displacement Parameters (Å^2×10^3) for ca080923_1_1. The Anisotropic displacement factor exponent takes the form: -2π^2[h^{2}a*^{2}U$_{11}$+2hka*b*U$_{12}$+…].

Atom	U_{11}	U_{22}	U_{33}	U_{23}	U_{13}	U_{12}
C23B	38(3)	48(3)	40(3)	-10(3)	13(2)	0(2)
C24B	30(2)	47(3)	34(3)	-2(2)	9(2)	-1(2)
C25B	27(2)	43(3)	39(3)	2(2)	12(2)	2(2)
C26B	38(3)	51(3)	43(3)	7(3)	9(2)	4(2)
C27B	39(3)	46(3)	67(4)	-6(3)	27(3)	-5(2)
C28B	59(4)	60(4)	108(6)	-12(4)	48(4)	-24(3)
C29B	68(4)	60(4)	80(5)	-29(4)	35(4)	-14(3)
C30B	73(4)	37(3)	93(5)	14(3)	48(4)	2(3)
C31B	33(3)	74(4)	54(3)	13(3)	13(2)	11(3)
C32B	39(3)	50(3)	53(3)	-3(3)	24(2)	-1(2)

Table 4 Bond Lengths for ca080923_1_1.

Atom	Atom	Length/Å	Atom	Atom	Length/Å
Si1A	O1A	1.653(3)	Si1B	O1B	1.644(3)
Si1A	C1A	1.886(6)	Si1B	C1B	1.894(6)
Si1A	C5A	1.866(6)	Si1B	C5B	1.864(6)
Si1A	C6A	1.868(5)	Si1B	C6B	1.857(6)
Si2A	O4A	1.628(3)	Si2B	O4B	1.642(3)
Si2A	C27A	1.878(5)	Si2B	C27B	1.875(6)
Si2A	C31A	1.862(6)	Si2B	C31B	1.860(6)
Si2A	C32A	1.860(5)	Si2B	C32B	1.849(6)
O1A	C7A	1.431(6)	O1B	C7B	1.430(6)
O2A	C11A	1.499(5)	O2B	C11B	1.494(5)
O2A	C14A	1.336(5)	O2B	C14B	1.347(6)
O3A	C14A	1.211(5)	O3B	C14B	1.203(6)
O4A	C17A	1.430(6)	O4B	C17B	1.430(5)
O5A	C13A	1.400(5)	O5B	C13B	1.398(6)
C1A	C2A	1.526(9)	C1B	C2B	1.544(9)
C1A	C3A	1.560(9)	C1B	C3B	1.543(7)
C1A	C4A	1.529(7)	C1B	C4B	1.525(10)
C7A	C8A	1.546(7)	C7B	C8B	1.546(7)
C7A	C12A	1.552(6)	C7B	C12B	1.549(7)
C8A	C9A	1.524(6)	C8B	C9B	1.525(7)
C8A	C10A	1.537(7)	C8B	C10B	1.551(7)
C10A	C11A	1.535(6)	C10B	C11B	1.533(7)
C10A	C13A	1.537(6)	C10B	C13B	1.522(7)
C11A	C12A	1.519(6)	C11B	C12B	1.522(6)
C11A	C18A	1.544(6)	C11B	C18B	1.544(6)
C12A	C25A	1.538(6)	C12B	C25B	1.535(7)
C13A	C14A	1.509(7)	C13B	C14B	1.519(6)

Table 4 Bond Lengths for ca080923_1_1.

Atom	Atom	Length/Å	Atom	Atom	Length/Å
C13A	C15A	1.597(7)	C13B	C15B	1.592(7)
C15A	C16A	1.522(7)	C15B	C16B	1.531(6)
C15A	C17A	1.548(6)	C15B	C17B	1.552(7)
C17A	C18A	1.544(6)	C17B	C18B	1.547(6)
C18A	C19A	1.516(6)	C18B	C19B	1.507(7)
C19A	C20A	1.528(7)	C19B	C20B	1.525(7)
C19A	C21A	1.523(7)	C19B	C21B	1.525(6)
C20A	C21A	1.517(7)	C20B	C21B	1.523(7)
C20A	C22A	1.515(7)	C20B	C22B	1.514(7)
C20A	C23A	1.502(9)	C20B	C23B	1.505(8)
C21A	C24A	1.510(7)	C21B	C24B	1.507(7)
C24A	C25A	1.544(7)	C24B	C25B	1.541(7)
C25A	C26A	1.538(8)	C25B	C26B	1.537(7)
C27A	C28A	1.539(8)	C27B	C28B	1.542(8)
C27A	C29A	1.541(7)	C27B	C29B	1.563(9)
C27A	C30A	1.540(7)	C27B	C30B	1.537(9)

Table 5 Bond Angles for ca080923_1_1.

Atom	Atom	Atom	Angle/°	Atom	Atom	Atom	Angle/°
O1A	Si1A	C1A	106.1(2)	O1B	Si1B	C1B	108.1(2)
O1A	Si1A	C5A	109.8(2)	O1B	Si1B	C5B	109.2(2)
O1A	Si1A	C6A	112.8(2)	O1B	Si1B	C6B	110.3(2)
C5A	Si1A	C1A	111.6(3)	C5B	Si1B	C1B	107.7(3)
C5A	Si1A	C6A	108.3(3)	C6B	Si1B	C1B	112.3(3)
C6A	Si1A	C1A	108.2(3)	C6B	Si1B	C5B	109.1(3)
O4A	Si2A	C27A	101.5(2)	O4B	Si2B	C27B	102.4(2)
O4A	Si2A	C31A	113.8(2)	O4B	Si2B	C31B	113.8(2)
O4A	Si2A	C32A	111.9(2)	O4B	Si2B	C32B	111.3(2)
C31A	Si2A	C27A	110.0(3)	C31B	Si2B	C27B	110.1(3)
C32A	Si2A	C27A	111.9(3)	C32B	Si2B	C27B	112.2(3)
C32A	Si2A	C31A	107.8(3)	C32B	Si2B	C31B	107.1(3)
C7A	O1A	Si1A	129.8(3)	C7B	O1B	Si1B	128.5(3)
C14A	O2A	C11A	108.9(3)	C14B	O2B	C11B	109.1(3)
C17A	O4A	Si2A	139.6(3)	C17B	O4B	Si2B	136.0(3)
C2A	C1A	Si1A	110.2(4)	C2B	C1B	Si1B	111.1(4)
C2A	C1A	C3A	110.3(5)	C3B	C1B	Si1B	109.2(4)
C2A	C1A	C4A	108.6(5)	C3B	C1B	C2B	107.5(5)
C3A	C1A	Si1A	108.7(5)	C4B	C1B	Si1B	110.1(5)
C4A	C1A	Si1A	112.5(4)	C4B	C1B	C2B	110.0(6)
C4A	C1A	C3A	106.5(5)	C4B	C1B	C3B	108.8(6)
O1A	C7A	C8A	112.7(3)	O1B	C7B	C8B	111.5(4)
O1A	C7A	C12A	111.4(4)	O1B	C7B	C12B	112.2(4)

Table 5 Bond Angles for ca080923_1_1.

Atom	Atom	Atom	Angle/°	Atom	Atom	Atom	Angle/°
C8A	C7A	C12A	102.5(4)	C8B	C7B	C12B	104.0(4)
C9A	C8A	C7A	114.8(4)	C7B	C8B	C10B	106.2(4)
C9A	C8A	C10A	118.2(4)	C9B	C8B	C7B	114.4(4)
C10A	C8A	C7A	106.4(4)	C9B	C8B	C10B	118.2(4)
C8A	C10A	C13A	122.9(4)	C11B	C10B	C8B	106.1(4)
C11A	C10A	C8A	106.6(4)	C13B	C10B	C8B	122.3(4)
C11A	C10A	C13A	97.6(3)	C13B	C10B	C11B	98.3(4)
O2A	C11A	C10A	103.9(3)	O2B	C11B	C10B	103.5(4)
O2A	C11A	C12A	107.0(4)	O2B	C11B	C12B	106.5(3)
O2A	C11A	C18A	105.7(4)	O2B	C11B	C18B	106.2(4)
C10A	C11A	C18A	113.2(4)	C10B	C11B	C18B	112.9(4)
C12A	C11A	C10A	104.4(4)	C12B	C11B	C10B	105.4(4)
C12A	C11A	C18A	121.3(3)	C12B	C11B	C18B	120.8(4)
C11A	C12A	C7A	103.5(3)	C11B	C12B	C7B	103.3(4)
C11A	C12A	C25A	117.3(4)	C11B	C12B	C25B	117.6(4)
C25A	C12A	C7A	116.8(4)	C25B	C12B	C7B	117.5(4)
O5A	C13A	C10A	112.9(4)	O5B	C13B	C10B	114.0(4)
O5A	C13A	C14A	115.1(3)	O5B	C13B	C14B	114.0(4)
O5A	C13A	C15A	113.5(4)	O5B	C13B	C15B	113.4(4)
C10A	C13A	C15A	109.2(3)	C10B	C13B	C15B	109.0(4)
C14A	C13A	C10A	100.7(4)	C14B	C13B	C10B	100.8(4)
C14A	C13A	C15A	104.4(4)	C14B	C13B	C15B	104.6(4)
O2A	C14A	C13A	108.2(3)	O2B	C14B	C13B	107.5(4)
O3A	C14A	O2A	122.3(4)	O3B	C14B	O2B	122.6(4)
O3A	C14A	C13A	129.4(4)	O3B	C14B	C13B	129.7(4)
C16A	C15A	C13A	111.9(4)	C16B	C15B	C13B	112.4(4)
C16A	C15A	C17A	111.9(4)	C16B	C15B	C17B	111.3(4)
C17A	C15A	C13A	113.2(4)	C17B	C15B	C13B	113.7(4)
O4A	C17A	C15A	109.9(4)	O4B	C17B	C15B	110.3(4)
O4A	C17A	C18A	110.9(4)	O4B	C17B	C18B	111.1(4)
C18A	C17A	C15A	111.1(4)	C18B	C17B	C15B	110.1(4)
C11A	C18A	C17A	109.5(3)	C11B	C18B	C17B	110.0(4)
C19A	C18A	C11A	111.8(4)	C19B	C18B	C11B	113.2(4)
C19A	C18A	C17A	114.5(4)	C19B	C18B	C17B	114.8(4)
C18A	C19A	C20A	121.8(5)	C18B	C19B	C20B	122.5(4)
C18A	C19A	C21A	116.7(4)	C18B	C19B	C21B	117.0(4)
C21A	C19A	C20A	59.6(3)	C20B	C19B	C21B	59.9(3)
C21A	C20A	C19A	60.1(3)	C21B	C20B	C19B	60.1(3)
C22A	C20A	C19A	116.0(5)	C22B	C20B	C19B	116.3(5)
C22A	C20A	C21A	115.4(5)	C22B	C20B	C21B	115.8(5)
C23A	C20A	C19A	121.8(4)	C23B	C20B	C19B	121.3(5)
C23A	C20A	C21A	121.9(5)	C23B	C20B	C21B	121.3(4)
C23A	C20A	C22A	112.4(5)	C23B	C20B	C22B	112.6(5)
C20A	C21A	C19A	60.3(3)	C20B	C21B	C19B	60.0(3)

Table 5 Bond Angles for ca080923_1_1.

Atom	Atom	Atom	Angle/°	Atom	Atom	Atom	Angle/°
C24A	C21A	C19A	118.4(4)	C24B	C21B	C19B	118.9(4)
C24A	C21A	C20A	124.9(5)	C24B	C21B	C20B	124.4(5)
C21A	C24A	C25A	113.7(5)	C21B	C24B	C25B	111.8(4)
C12A	C25A	C24A	110.8(4)	C12B	C25B	C24B	112.0(4)
C26A	C25A	C12A	110.0(5)	C12B	C25B	C26B	110.0(4)
C26A	C25A	C24A	111.4(4)	C26B	C25B	C24B	111.2(4)
C28A	C27A	Si2A	110.3(4)	C28B	C27B	Si2B	110.9(4)
C28A	C27A	C29A	109.6(5)	C28B	C27B	C29B	109.6(5)
C28A	C27A	C30A	109.1(5)	C29B	C27B	Si2B	108.1(5)
C29A	C27A	Si2A	109.4(4)	C30B	C27B	Si2B	109.3(4)
C30A	C27A	Si2A	110.0(4)	C30B	C27B	C28B	109.3(6)
C30A	C27A	C29A	108.4(4)	C30B	C27B	C29B	109.5(5)

Table 6 Torsion Angles for ca080923_1_1.

A	B	C	D	Angle/°	A	B	C	D	Angle/°
Si1A	O1A	C7A	C8A	-103.5(4)	Si1B	O1B	C7B	C8B	-123.1(4)
Si1A	O1A	C7A	C12A	142.0(3)	Si1B	O1B	C7B	C12B	120.7(4)
Si2A	O4A	C17A	C15A	-113.8(4)	Si2B	O4B	C17B	C15B	-116.0(4)
Si2A	O4A	C17A	C18A	122.9(4)	Si2B	O4B	C17B	C18B	121.6(4)
O1A	Si1A	C1A	C2A	-72.6(5)	O1B	Si1B	C1B	C2B	-67.5(5)
O1A	Si1A	C1A	C3A	166.4(4)	O1B	Si1B	C1B	C3B	174.1(5)
O1A	Si1A	C1A	C4A	48.7(5)	O1B	Si1B	C1B	C4B	54.7(5)
O1A	C7A	C8A	C9A	39.4(6)	O1B	C7B	C8B	C9B	34.4(6)
O1A	C7A	C8A	C10A	-93.3(4)	O1B	C7B	C8B	C10B	-97.8(4)
O1A	C7A	C12A	C11A	80.6(4)	O1B	C7B	C12B	C11B	83.4(4)
O1A	C7A	C12A	C25A	-49.9(6)	O1B	C7B	C12B	C25B	-47.9(6)
O2A	C11A	C12A	C7A	-71.4(4)	O2B	C11B	C12B	C7B	-72.4(4)
O2A	C11A	C12A	C25A	58.8(5)	O2B	C11B	C12B	C25B	58.9(5)
O2A	C11A	C18A	C17A	90.7(4)	O2B	C11B	C18B	C17B	91.9(4)
O2A	C11A	C18A	C19A	-141.3(4)	O2B	C11B	C18B	C19B	-138.3(4)
O4A	Si2A	C27A	C28A	176.8(4)	O4B	Si2B	C27B	C28B	176.8(5)
O4A	Si2A	C27A	C29A	-62.5(4)	O4B	Si2B	C27B	C29B	-63.0(4)
O4A	Si2A	C27A	C30A	56.5(4)	O4B	Si2B	C27B	C30B	56.2(5)
O4A	C17A	C18A	C11A	82.4(4)	O4B	C17B	C18B	C11B	81.8(5)
O4A	C17A	C18A	C19A	-44.1(5)	O4B	C17B	C18B	C19B	-47.2(6)
O5A	C13A	C14A	O2A	-158.5(4)	O5B	C13B	C14B	O2B	-158.5(4)
O5A	C13A	C14A	O3A	25.9(7)	O5B	C13B	C14B	O3B	24.6(7)
O5A	C13A	C15A	C16A	8.4(5)	O5B	C13B	C15B	C16B	9.2(6)
O5A	C13A	C15A	C17A	135.9(4)	O5B	C13B	C15B	C17B	136.8(4)
C1A	Si1A	O1A	C7A	-148.7(4)	C1B	Si1B	O1B	C7B	148.0(4)
C5A	Si1A	O1A	C7A	-27.9(4)	C5B	Si1B	O1B	C7B	-95.1(5)
C5A	Si1A	C1A	C2A	167.7(4)	C5B	Si1B	C1B	C2B	174.6(5)

Table 6 Torsion Angles for ca080923_1_1.

A	B	C	D	Angle/°	A	B	C	D	Angle/°
C5A	Si1A	C1A	C3A	46.8(5)	C5B	Si1B	C1B	C3B	56.2(5)
C5A	Si1A	C1A	C4A	-70.9(5)	C5B	Si1B	C1B	C4B	-63.2(5)
C6A	Si1A	O1A	C7A	93.1(4)	C6B	Si1B	O1B	C7B	24.8(5)
C6A	Si1A	C1A	C2A	48.7(5)	C6B	Si1B	C1B	C2B	54.4(5)
C6A	Si1A	C1A	C3A	-72.3(5)	C6B	Si1B	C1B	C3B	-64.0(5)
C6A	Si1A	C1A	C4A	170.0(4)	C6B	Si1B	C1B	C4B	176.6(4)
C7A	C8A	C10A	C11A	-3.6(4)	C7B	C8B	C10B	C11B	-0.8(5)
C7A	C8A	C10A	C13A	107.4(5)	C7B	C8B	C10B	C13B	110.3(5)
C7A	C12A	C25A	C24A	168.9(4)	C7B	C12B	C25B	C24B	166.7(4)
C7A	C12A	C25A	C26A	-67.5(5)	C7B	C12B	C25B	C26B	-69.1(6)
C8A	C7A	C12A	C11A	-40.1(4)	C8B	C7B	C12B	C11B	-37.3(5)
C8A	C7A	C12A	C25A	-170.6(4)	C8B	C7B	C12B	C25B	-168.7(4)
C8A	C10A	C11A	O2A	90.4(4)	C8B	C10B	C11B	O2B	89.1(4)
C8A	C10A	C11A	C12A	-21.6(4)	C8B	C10B	C11B	C12B	-22.6(5)
C8A	C10A	C11A	C18A	-155.5(4)	C8B	C10B	C11B	C18B	-156.6(4)
C8A	C10A	C13A	O5A	51.4(6)	C8B	C10B	C13B	O5B	51.2(5)
C8A	C10A	C13A	C14A	-71.8(5)	C8B	C10B	C13B	C14B	-71.4(5)
C8A	C10A	C13A	C15A	178.7(4)	C8B	C10B	C13B	C15B	178.9(4)
C9A	C8A	C10A	C11A	-134.3(4)	C9B	C8B	C10B	C11B	-130.9(4)
C9A	C8A	C10A	C13A	-23.4(6)	C9B	C8B	C10B	C13B	-19.8(6)
C10A	C11A	C12A	C7A	38.4(4)	C10B	C11B	C12B	C7B	37.2(4)
C10A	C11A	C12A	C25A	168.6(4)	C10B	C11B	C12B	C25B	168.5(4)
C10A	C11A	C18A	C17A	-22.4(5)	C10B	C11B	C18B	C17B	-20.9(5)
C10A	C11A	C18A	C19A	105.6(4)	C10B	C11B	C18B	C19B	108.9(4)
C10A	C13A	C14A	O2A	-36.8(4)	C10B	C13B	C14B	O2B	-35.9(5)
C10A	C13A	C14A	O3A	147.6(5)	C10B	C13B	C14B	O3B	147.2(5)
C10A	C13A	C15A	C16A	-118.6(4)	C10B	C13B	C15B	C16B	-118.9(4)
C10A	C13A	C15A	C17A	8.9(5)	C10B	C13B	C15B	C17B	8.6(5)
C11A	O2A	C14A	O3A	-171.5(4)	C11B	O2B	C14B	O3B	-171.6(4)
C11A	O2A	C14A	C13A	12.6(5)	C11B	O2B	C14B	C13B	11.3(5)
C11A	C10A	C13A	O5A	166.8(4)	C11B	C10B	C13B	O5B	166.3(3)
C11A	C10A	C13A	C14A	43.6(4)	C11B	C10B	C13B	C14B	43.7(4)
C11A	C10A	C13A	C15A	-65.9(4)	C11B	C10B	C13B	C15B	-66.0(4)
C11A	C12A	C25A	C24A	45.2(6)	C11B	C12B	C25B	C24B	42.2(6)
C11A	C12A	C25A	C26A	168.9(4)	C11B	C12B	C25B	C26B	166.4(4)
C11A	C18A	C19A	C20A	132.2(5)	C11B	C18B	C19B	C20B	130.7(5)
C11A	C18A	C19A	C21A	62.9(6)	C11B	C18B	C19B	C21B	60.7(6)
C12A	C7A	C8A	C9A	159.3(4)	C12B	C7B	C8B	C9B	155.6(4)
C12A	C7A	C8A	C10A	26.6(4)	C12B	C7B	C8B	C10B	23.4(5)
C12A	C11A	C18A	C17A	-147.6(4)	C12B	C11B	C18B	C17B	-147.0(4)
C12A	C11A	C18A	C19A	-19.6(6)	C12B	C11B	C18B	C19B	-17.1(6)
C13A	C10A	C11A	O2A	-37.4(4)	C13B	C10B	C11B	O2B	-38.1(4)
C13A	C10A	C11A	C12A	-149.3(3)	C13B	C10B	C11B	C12B	-149.8(4)
C13A	C10A	C11A	C18A	76.8(4)	C13B	C10B	C11B	C18B	76.3(4)

Table 6 Torsion Angles for ca080923_1_1.

A	B	C	D	Angle/°	A	B	C	D	Angle/°
C13A	C15A	C17A	O4A	-75.5(5)	C13B	C15B	C17B	O4B	-75.0(5)
C13A	C15A	C17A	C18A	47.7(5)	C13B	C15B	C17B	C18B	47.9(5)
C14A	O2A	C11A	C10A	16.8(5)	C14B	O2B	C11B	C10B	17.7(5)
C14A	O2A	C11A	C12A	126.9(4)	C14B	O2B	C11B	C12B	128.6(4)
C14A	O2A	C11A	C18A	-102.5(4)	C14B	O2B	C11B	C18B	-101.4(4)
C14A	C13A	C15A	C16A	134.5(4)	C14B	C13B	C15B	C16B	134.0(4)
C14A	C13A	C15A	C17A	-98.0(4)	C14B	C13B	C15B	C17B	-98.4(4)
C15A	C13A	C14A	O2A	76.4(4)	C15B	C13B	C14B	O2B	77.1(4)
C15A	C13A	C14A	O3A	-99.2(5)	C15B	C13B	C14B	O3B	-99.8(6)
C15A	C17A	C18A	C11A	-40.2(5)	C15B	C17B	C18B	C11B	-40.7(5)
C15A	C17A	C18A	C19A	-166.7(4)	C15B	C17B	C18B	C19B	-169.7(4)
C16A	C15A	C17A	O4A	52.0(5)	C16B	C15B	C17B	O4B	53.1(5)
C16A	C15A	C17A	C18A	175.2(4)	C16B	C15B	C17B	C18B	176.1(4)
C17A	C18A	C19A	C20A	-102.5(5)	C17B	C18B	C19B	C20B	-101.9(5)
C17A	C18A	C19A	C21A	-171.8(4)	C17B	C18B	C19B	C21B	-171.9(4)
C18A	C11A	C12A	C7A	167.5(4)	C18B	C11B	C12B	C7B	166.6(4)
C18A	C11A	C12A	C25A	-62.2(6)	C18B	C11B	C12B	C25B	-62.1(6)
C18A	C19A	C20A	C21A	-104.4(5)	C18B	C19B	C20B	C21B	-104.7(5)
C18A	C19A	C20A	C22A	149.9(5)	C18B	C19B	C20B	C22B	149.4(5)
C18A	C19A	C20A	C23A	6.6(8)	C18B	C19B	C20B	C23B	5.9(7)
C18A	C19A	C21A	C20A	112.9(5)	C18B	C19B	C21B	C20B	113.7(5)
C18A	C19A	C21A	C24A	-3.2(7)	C18B	C19B	C21B	C24B	-1.5(7)
C19A	C20A	C21A	C24A	105.7(5)	C19B	C20B	C21B	C24B	106.4(5)
C19A	C21A	C24A	C25A	-76.0(6)	C19B	C21B	C24B	C25B	-77.2(6)
C20A	C19A	C21A	C24A	-116.1(6)	C20B	C19B	C21B	C24B	-115.2(6)
C20A	C21A	C24A	C25A	-148.0(5)	C20B	C21B	C24B	C25B	-148.9(5)
C21A	C19A	C20A	C22A	-105.6(5)	C21B	C19B	C20B	C22B	-106.0(5)
C21A	C19A	C20A	C23A	111.1(6)	C21B	C19B	C20B	C23B	110.6(5)
C21A	C24A	C25A	C12A	43.0(6)	C21B	C24B	C25B	C12B	45.4(6)
C21A	C24A	C25A	C26A	-79.9(6)	C21B	C24B	C25B	C26B	-78.1(5)
C22A	C20A	C21A	C19A	106.7(5)	C22B	C20B	C21B	C19B	106.9(5)
C22A	C20A	C21A	C24A	-147.6(5)	C22B	C20B	C21B	C24B	-146.8(5)
C23A	C20A	C21A	C19A	-111.0(5)	C23B	C20B	C21B	C19B	-110.5(5)
C23A	C20A	C21A	C24A	-5.3(7)	C23B	C20B	C21B	C24B	-4.2(8)
C27A	Si2A	O4A	C17A	176.1(4)	C27B	Si2B	O4B	C17B	176.7(4)
C31A	Si2A	O4A	C17A	58.1(5)	C31B	Si2B	O4B	C17B	57.9(5)
C31A	Si2A	C27A	C28A	-62.3(4)	C31B	Si2B	C27B	C28B	-61.8(5)
C31A	Si2A	C27A	C29A	58.3(4)	C31B	Si2B	C27B	C29B	58.4(5)
C31A	Si2A	C27A	C30A	177.3(4)	C31B	Si2B	C27B	C30B	177.6(4)
C32A	Si2A	O4A	C17A	-64.4(5)	C32B	Si2B	O4B	C17B	-63.3(5)
C32A	Si2A	C27A	C28A	57.4(5)	C32B	Si2B	C27B	C28B	57.4(5)
C32A	Si2A	C27A	C29A	178.0(4)	C32B	Si2B	C27B	C29B	177.6(4)
C32A	Si2A	C27A	C30A	-63.0(4)	C32B	Si2B	C27B	C30B	-63.2(5)

Table 7 Hydrogen Atom Coordinates (Å×10^4) and Isotropic Displacement Parameters (Å^2×10^3) for ca080923_1_1.

Atom	*x*	*y*	*z*	U(eq)
H5A	4911(14)	9660(60)	4599(17)	50
H2AA	4720.96	8595.85	6343.04	114
H2AB	4413.97	10064.06	6453.52	114
H2AC	4365.51	9909.55	5952.41	114
H3AA	3840.02	5855.63	6469.34	107
H3AB	4113.76	7583.58	6773.16	107
H3AC	4392.94	6003.34	6659.13	107
H4AA	3528.4	9600.07	5690	73
H4AB	3558.57	9498.9	6184.97	73
H4AC	3314.24	7866.52	5831.85	73
H5AA	3356.14	4423.57	5538.69	76
H5AB	3795.33	3368.75	5916.85	76
H5AC	3682.67	3192.01	5396.41	76
H6AA	4670.48	4226.94	5703.22	80
H6AB	4787.98	4885.65	6203.94	80
H6AC	4852.89	6281.97	5862.06	80
H7A	3447.55	5305.31	4751.64	39
H8A	3744.83	5357.85	4263.08	36
H9AA	4566.88	6496.6	5023.88	49
H9AB	4548.22	5379.16	4599.38	49
H9AC	4372.49	4426.93	4931.5	49
H10A	3728.31	7863.06	3911.46	33
H12A	3014.47	7237.34	4163.75	37
H15A	4103.75	12407.94	4213.9	38
H16A	4691.13	11782.13	4017.17	62
H16B	4251.65	12518.86	3576.93	62
H16C	4369.84	10360.57	3634.56	62
H17A	3445.32	12361.47	3538.12	35
H18A	3223.9	11906.24	4089.38	36
H19A	2709.39	9201.4	3399.78	40
H21A	2190.07	8950.83	3725.77	46
H22A	1906.08	9882.87	2961.97	79
H22B	1979.69	11904.49	2810.07	79
H22C	1642.91	11612.02	3043.38	79
H23A	2126.16	14022.64	3618.24	75
H23B	2489.57	14178.61	3411.38	75
H23C	2667.29	13632.74	3930.55	75
H24A	2342.27	10555.82	4417.21	48
H24B	2803.56	11435.25	4427.89	48
H25A	3089.47	9143.12	4947.15	45
H26A	2353.78	6777.09	4352.62	79

Table 7 Hydrogen Atom Coordinates (Å×10^4) and Isotropic Displacement Parameters (Å^2×10^3) for ca080923_1_1.

Atom	*x*	*y*	*z*	U(eq)
H26B	2408.62	7581.9	4823.79	79
H26C	2767.93	6068.82	4816.04	79
H28A	2909.7	6937.64	2081.84	81
H28B	3273.18	5264.32	2228.64	81
H28C	3427.85	7270.78	2144.76	81
H29A	4104.22	7340.05	2937.32	70
H29B	3960.08	5267.21	2985.3	70
H29C	4008.31	6780.77	3355.58	70
H30A	3230.44	6154.46	3263.32	68
H30B	3134.17	4630.02	2882.06	68
H30C	2783.12	6327.46	2783.59	68
H31A	3654.03	12294.43	2677.33	74
H31B	3584.55	10752.72	2306.51	74
H31C	4011.51	10606.45	2800.6	74
H32A	2494.71	9513.64	2539.19	76
H32B	2624.61	10300.81	2158.49	76
H32C	2661.83	11608.69	2562.6	76
H5B	4587(19)	2480(70)	-45(16)	53
H2BA	6302.9	-2848.39	698.38	116
H2BB	6440.07	-1374.66	418.31	116
H2BC	6000.21	-1010.45	513.01	116
H3BA	7131.65	-2610.81	1290.5	99
H3BB	7339.93	-667.01	1522.43	99
H3BC	7234.69	-1033.15	1009.15	99
H4BA	6316.37	2031	885.13	108
H4BB	6743.18	1759.56	765.57	108
H4BC	6839.98	2168.55	1274.86	108
H5BA	6878.18	1447.28	2022.36	76
H5BB	7179.56	-410.12	2145.12	76
H5BC	6784.05	-135.72	2308.37	76
H6BA	6315.46	-3632.6	1882.08	79
H6BB	6690.34	-4069.37	1700.42	79
H6BC	6147.39	-4025.55	1356.62	79
H7B	5631.78	-1702.83	1640.35	44
H8B	4915.6	-1752.64	1091.14	41
H9BA	5327.76	-578.5	534.83	57
H9BB	4865.59	-1797.38	375.99	57
H9BC	5358.13	-2636.4	721.5	57
H10B	4508.96	751.37	954.56	37
H12B	5220.72	329.97	1842.24	40
H15B	4609.86	5214.81	621.97	41
H16D	4036	4528.44	-83.66	62
H16E	3782.2	5220.67	208.7	62

Table 7 Hydrogen Atom Coordinates (Å×10^4) and Isotropic Displacement Parameters (Å^2×10^3) for ca080923_1_1.

Atom	*x*	*y*	*z*	U(eq)
H16F	3800.69	3064.71	112.96	62
H17B	4218.25	5266.89	1055.27	37
H18B	4987.79	4915.37	1509.68	37
H19B	4525.58	2395.6	1849.54	40
H21B	5216.48	2308.12	2536.39	46
H22D	4523.93	3343.28	2544.77	75
H22E	4275.46	5269.74	2339.76	75
H22F	4747.63	5230.7	2801.09	75
H23D	5103.94	7411.98	2462.08	66
H23E	4663.37	7406.25	1975.07	66
H23F	5164.39	6807.28	2026.65	66
H24C	5911.39	3893.75	2614.58	46
H24D	5642.25	4603.49	2103.96	46
H25B	6074.33	2184.63	2059.94	45
H26D	5807.81	123.98	2645.23	72
H26E	6320.97	847.93	2766.21	72
H26F	6093.79	-767.45	2403.48	72
H28D	2876.64	18.78	1156.74	107
H28E	2775.23	-1564.99	787.66	107
H28F	2624.8	520.01	630.66	107
H29D	3088.91	210.85	167.19	103
H29E	3247.61	-1825.23	363.09	103
H29F	3630.68	-296.58	423.14	103
H30D	4013.63	-857.19	1253.48	96
H30E	3628.22	-2323.4	1223.58	96
H30F	3737.57	-567.9	1548.48	96
H31D	3159.57	5256.15	501.02	84
H31E	2753.43	3944.85	489.42	84
H31F	3016.61	3443.8	192.21	84
H32D	3700.29	2773.68	1736.9	69
H32E	3174.95	3463.98	1452.89	69
H32F	3598.45	4803.83	1524.1	69

Experimental

Single crystals of $C_{32}H_{58}O_5Si_2$ **[ca080923_1_1]** were **[]**. A suitable crystal was selected and **[]** on a **XtaLAB Synergy, Dualflex, Pilatus 300K** diffractometer. The crystal was kept at 100.0(1) K during data collection. Using Olex2 [1], the structure was solved with the SHELXT [2] structure solution program using Intrinsic Phasing and refined with the SHELXL [3] refinement package using Least Squares minimisation.

1. Dolomanov, O.V., Bourhis, L.J., Gildea, R.J, Howard, J.A.K. & Puschmann, H. (2009), J. Appl. Cryst. 42, 339-341.
2. Sheldrick, G.M. (2015). Acta Cryst. A71, 3-8.
3. Sheldrick, G.M. (2015). Acta Cryst. C71, 3-8.

Crystal structure determination of [ca080923_1_1]

Crystal Data for $C_{32}H_{58}O_5Si_2$ (M =578.96 g/mol): monoclinic, space group C2 (no. 5), a = 32.1203(10) Å, b = 7.2454(2) Å, c = 33.1269(12) Å, β = 116.597(4)°, V = 6893.6(4) Å^3, Z = 8, T = 100.0(1) K, μ(Cu Kα) = 1.204 mm^{-1}, $Dcalc$ = 1.116 g/cm^3, 43812 reflections measured (7.954° ≤ 2Θ ≤ 161.868°), 13239 unique (R_{int} = 0.0713, R_{sigma} = 0.0671) which were used in all calculations. The final R_1 was 0.0530 (I > 2σ(I)) and wR_2 was 0.1485 (all data).

Refinement model description

Number of restraints - 3, number of constraints - unknown.

Details:

```
1. Fixed Uiso
 At 1.2 times of:
  All C(H) groups, All C(H,H) groups
 At 1.5 times of:
  All C(H,H,H) groups, All O(H) groups
2. Restrained distances
 O5A-H5A = O5B-H5B
 0.93 with sigma of 0.01
3.a Ternary CH refined with riding coordinates:
 C7A(H7A), C8A(H8A), C10A(H10A), C12A(H12A), C15A(H15A), C17A(H17A),
 C18A(H18A), C19A(H19A), C21A(H21A), C25A(H25A), C7B(H7B), C8B(H8B), C10B(H10B),
  C12B(H12B), C15B(H15B), C17B(H17B), C18B(H18B), C19B(H19B), C21B(H21B),
 C25B(H25B)
3.b Secondary CH2 refined with riding coordinates:
 C24A(H24A,H24B), C24B(H24C,H24D)
3.c Idealised Me refined as rotating group:
 C2A(H2AA,H2AB,H2AC), C3A(H3AA,H3AB,H3AC), C4A(H4AA,H4AB,H4AC), C5A(H5AA,H5AB,
 H5AC), C6A(H6AA,H6AB,H6AC), C9A(H9AA,H9AB,H9AC), C16A(H16A,H16B,H16C),
 C22A(H22A,H22B,H22C), C23A(H23A,H23B,H23C), C26A(H26A,H26B,H26C), C28A(H28A,
 H28B,H28C), C29A(H29A,H29B,H29C), C30A(H30A,H30B,H30C), C31A(H31A,H31B,H31C),
 C32A(H32A,H32B,H32C), C2B(H2BA,H2BB,H2BC), C3B(H3BA,H3BB,H3BC), C4B(H4BA,H4BB,
 H4BC), C5B(H5BA,H5BB,H5BC), C6B(H6BA,H6BB,H6BC), C9B(H9BA,H9BB,H9BC),
 C16B(H16D,H16E,H16F), C22B(H22D,H22E,H22F), C23B(H23D,H23E,H23F), C26B(H26D,
 H26E,H26F), C28B(H28D,H28E,H28F), C29B(H29D,H29E,H29F), C30B(H30D,H30E,H30F),
 C31B(H31D,H31E,H31F), C32B(H32D,H32E,H32F)
```

ten membered ring olefine **12**:

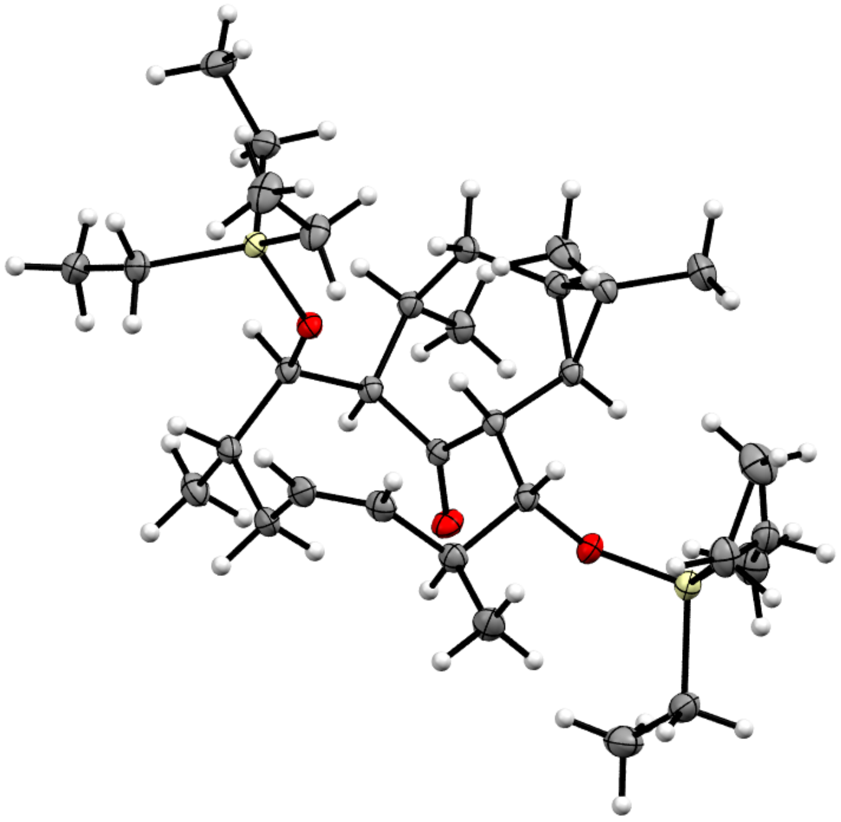

Table 1 Crystal data and structure refinement for ca080223_1_1.

Identification code	ca080223_1_1
Empirical formula	$C_{32}H_{60}O_3Si_2$
Formula weight	548.98
Temperature/K	100.0(1)
Crystal system	monoclinic
Space group	$P2_1$
a/Å	10.37740(10)
b/Å	10.82020(10)
c/Å	15.52420(10)
α/°	90
β/°	107.8970(10)
γ/°	90
Volume/Å^3	1658.79(3)
Z	2
ρ_{calc}g/cm^3	1.099
μ/mm^{-1}	1.178
F(000)	608.0
Crystal size/mm^3	0.286 × 0.253 × 0.148
Radiation	Cu Kα (λ = 1.54184)

2Θ range for data collection/°	8.954 to 159.674
Index ranges	$-13 \le h \le 13$, $-13 \le k \le 12$, $-19 \le l \le 19$
Reflections collected	51224
Independent reflections	7033 [R_{int} = 0.0399, R_{sigma} = 0.0211]
Data/restraints/parameters	7033/1/345
Goodness-of-fit on F^2	1.084
Final R indexes [I>=2σ (I)]	R_1 = 0.0286, wR_2 = 0.0760
Final R indexes [all data]	R_1 = 0.0291, wR_2 = 0.0764
Largest diff. peak/hole / e $Å^{-3}$	0.23/-0.20
Flack parameter	-0.012(10)

Table 2 Fractional Atomic Coordinates (×10^4) and Equivalent Isotropic Displacement Parameters (Å^2×10^3) for ca080223_1_1. U_{eq} is defined as 1/3 of the trace of the orthogonalised U_{IJ} tensor.

Atom	*x*	*y*	*z*	U(eq)
Si1	8932.9(5)	7880.0(4)	6547.4(3)	16.64(11)
Si2	5322.8(5)	2515.8(5)	9025.7(3)	20.17(12)
O1	5672.2(14)	3422.5(14)	6319.4(9)	22.6(3)
O2	7978.2(13)	6637.8(12)	6492.4(9)	17.3(3)
O3	5577.6(13)	3307.3(13)	8190.4(9)	20.4(3)
C1	9566(2)	3892.5(18)	8811.5(13)	20.9(4)
C2	10147(2)	2997(2)	9582.0(14)	27.7(4)
C3	9825(2)	5228(2)	9090.5(15)	27.7(4)
C4	9590.8(18)	3458.2(18)	7890.0(12)	18.4(4)
C5	8254.5(18)	3512.5(18)	8095.7(12)	17.1(4)
C6	7168.6(18)	4442.5(17)	7617.8(12)	16.0(3)
C7	6659.9(18)	4082.7(17)	6618.3(12)	15.6(3)
C8	7439.5(18)	4498.6(17)	5971.3(12)	16.1(3)
C9	8911.4(19)	3998.3(17)	6215.5(13)	18.1(4)
C10	8887(2)	2608.6(18)	6022.5(13)	22.2(4)
C11	9839.9(19)	4293.2(18)	7177.4(13)	19.8(4)
C12	6059.3(19)	4513.1(17)	8093.2(13)	17.4(4)
C13	4847.6(19)	5341.5(19)	7598.9(13)	21.4(4)
C14	3862(2)	5473(2)	8156.3(16)	28.5(4)
C15	5308(2)	6605.9(19)	7413.4(14)	22.5(4)
C16	5313(2)	7053.3(19)	6616.6(14)	23.2(4)
C17	4878.1(19)	6400(2)	5723.4(13)	22.8(4)
C18	5933.3(18)	6383.3(18)	5204.7(12)	19.5(4)
C19	5353(2)	5702(2)	4306.1(14)	25.9(4)
C20	7357.4(18)	5888.7(17)	5721.5(12)	17.0(4)
C21	9148(2)	8447(2)	7724.5(14)	26.2(4)
C22	9447(3)	9821(2)	7914.3(16)	32.8(5)

Table 2 Fractional Atomic Coordinates (×10^4) and Equivalent Isotropic Displacement Parameters (Å^2×10^3) for ca080223_1_1. U_{eq} is defined as 1/3 of the trace of the orthogonalised U_{IJ} tensor.

Atom	*x*	*y*	*z*	U(eq)
C23	10585.5(19)	7406(2)	6390.8(15)	25.7(4)
C24	11589(2)	8464(2)	6442.7(16)	29.6(5)
C25	8160(2)	9117.4(19)	5699.9(14)	23.2(4)
C26	8061(2)	8874(2)	4706.8(14)	28.2(5)
C27	3486(2)	2054(2)	8659.8(15)	27.4(4)
C28	2879(2)	1777(2)	7649.9(16)	34.5(5)
C29	6446(2)	1122(2)	9221.2(14)	24.4(4)
C30	6149(3)	192(2)	8439.0(17)	34.4(5)
C31	5689(2)	3420(2)	10105.6(14)	29.0(4)
C32	7178(3)	3552(3)	10664.1(16)	38.0(5)

Table 3 Anisotropic Displacement Parameters (Å^2×10^3) for ca080223_1_1. The Anisotropic displacement factor exponent takes the form: -2π^2[h^{2}a*^{2}U_{11}+2hka*b*U_{12}+…].

Atom	U_{11}	U_{22}	U_{33}	U_{23}	U_{13}	U_{12}
Si1	17.5(2)	12.4(2)	18.9(2)	0.56(18)	3.91(17)	-1.28(18)
Si2	22.6(2)	18.8(3)	20.5(2)	2.81(19)	8.80(19)	1.36(19)
O1	24.3(7)	20.4(6)	22.6(6)	-3.6(6)	6.6(5)	-7.3(6)
O2	18.8(6)	13.2(6)	18.4(6)	-0.5(5)	3.4(5)	-1.6(5)
O3	24.1(6)	17.3(7)	20.7(6)	1.0(5)	8.5(5)	-2.9(5)
C1	22.1(9)	19.0(10)	18.0(9)	1.1(7)	0.8(7)	1.6(7)
C2	30.3(10)	27.4(11)	21.4(9)	3.8(8)	2.1(8)	5.1(9)
C3	29.1(10)	21.3(10)	25.6(10)	-4.5(8)	-1.7(8)	-2.5(8)
C4	18.8(8)	15.2(9)	19.8(8)	0.6(7)	3.8(7)	1.7(7)
C5	19.6(8)	13.0(8)	18.0(8)	1.0(7)	4.7(7)	0.5(7)
C6	17.4(8)	12.8(8)	16.5(8)	0.4(7)	3.3(6)	-0.8(7)
C7	17.0(8)	10.5(8)	18.2(8)	0.2(7)	3.6(7)	1.2(6)
C8	18.4(8)	13.2(8)	15.8(8)	-0.6(7)	3.9(7)	-0.7(7)
C9	19.1(9)	15.8(9)	19.7(9)	2.1(7)	6.7(7)	0.0(7)
C10	25.9(9)	18.3(10)	22.7(9)	-0.4(7)	7.8(7)	2.9(7)
C11	17.6(8)	16.7(9)	23.3(9)	2.3(7)	4.0(7)	-0.2(7)
C12	20.5(9)	13.8(8)	17.5(8)	0.6(7)	5.0(7)	-0.8(7)
C13	21.4(9)	20.9(9)	22.4(9)	1.9(8)	7.5(7)	2.9(7)
C14	26.6(10)	28.1(11)	34.1(11)	5.6(9)	14.1(9)	7.5(8)
C15	21.6(9)	17.6(9)	27.5(10)	-0.2(8)	6.4(8)	3.8(7)
C16	21.7(9)	17.4(9)	30.5(10)	3.6(8)	8.2(8)	4.4(7)
C17	18.6(9)	21.5(10)	25.5(9)	7.1(8)	2.6(7)	2.5(7)
C18	19.4(9)	16.4(9)	20.0(9)	3.9(7)	2.3(7)	-0.6(7)
C19	27.1(10)	24.2(10)	20.8(9)	1.2(8)	-0.9(8)	0.1(8)
C20	19.3(9)	13.9(8)	16.9(8)	0.5(7)	4.4(7)	-2.0(7)

Table 3 Anisotropic Displacement Parameters (Å^2×10^3) for ca080223_1_1. The Anisotropic displacement factor exponent takes the form: -2π^2[h^{2}a*^{2}U$_{11}$+2hka*b*U$_{12}$+…].

Atom	U_{11}	U_{22}	U_{33}	U_{23}	U_{13}	U_{12}
C21	36.2(11)	17.9(9)	23.0(10)	-2.7(8)	6.7(8)	-6.5(9)
C22	46.6(13)	20.0(11)	31.6(11)	-5.6(9)	11.8(10)	-7.1(10)
C23	19.9(9)	22.6(10)	33.5(10)	3.4(9)	6.7(8)	0.3(8)
C24	22.0(9)	34.4(12)	32.7(11)	-0.7(10)	8.6(8)	-8.8(9)
C25	25.5(10)	16.7(9)	26.9(10)	4.8(8)	7.3(8)	2.0(8)
C26	24.4(10)	33.5(12)	26.4(10)	9.6(9)	7.4(8)	-2.1(9)
C27	24.7(9)	25.7(10)	34.8(11)	4.6(9)	13.3(8)	-0.5(8)
C28	24.1(10)	36.6(13)	40.1(13)	-0.6(10)	5.9(9)	-3.3(9)
C29	25.5(10)	22.3(10)	26.6(10)	6.5(8)	9.7(8)	2.4(8)
C30	40.7(13)	27.5(12)	37.9(13)	1.4(10)	16.1(10)	9.7(10)
C31	36.9(11)	29.0(11)	22.5(10)	0.3(9)	11.2(8)	5.2(10)
C32	42.3(13)	40.1(14)	25.9(11)	-6.5(10)	2.3(10)	0.4(11)

Table 4 Bond Lengths for ca080223_1_1.

Atom	Atom	Length/Å	Atom	Atom	Length/Å
Si1	O2	1.6563(14)	C7	C8	1.538(2)
Si1	C21	1.875(2)	C8	C9	1.554(3)
Si1	C23	1.876(2)	C8	C20	1.549(3)
Si1	C25	1.876(2)	C9	C10	1.532(3)
Si2	O3	1.6418(14)	C9	C11	1.541(3)
Si2	C27	1.881(2)	C12	C13	1.543(3)
Si2	C29	1.873(2)	C13	C14	1.536(3)
Si2	C31	1.876(2)	C13	C15	1.506(3)
O1	C7	1.218(2)	C15	C16	1.330(3)
O2	C20	1.425(2)	C16	C17	1.497(3)
O3	C12	1.422(2)	C17	C18	1.546(3)
C1	C2	1.513(3)	C18	C19	1.528(3)
C1	C3	1.509(3)	C18	C20	1.544(2)
C1	C4	1.513(3)	C21	C22	1.528(3)
C1	C5	1.526(2)	C23	C24	1.533(3)
C4	C5	1.517(2)	C25	C26	1.536(3)
C4	C11	1.511(3)	C27	C28	1.529(3)
C5	C6	1.523(2)	C29	C30	1.533(3)
C6	C7	1.528(2)	C31	C32	1.529(3)
C6	C12	1.548(2)			

Table 5 Bond Angles for ca080223_1_1.

Atom	Atom	Atom	Angle/°	Atom	Atom	Atom	Angle/°
O2	Si1	C21	101.89(8)	O1	C7	C8	118.22(16)
O2	Si1	C23	109.12(9)	C6	C7	C8	120.12(15)
O2	Si1	C25	115.04(8)	C7	C8	C9	114.44(15)
C21	Si1	C23	112.09(10)	C7	C8	C20	116.62(15)
C21	Si1	C25	110.31(10)	C20	C8	C9	111.95(15)
C25	Si1	C23	108.36(9)	C10	C9	C8	109.79(15)
O3	Si2	C27	107.10(9)	C10	C9	C11	111.13(16)
O3	Si2	C29	107.75(8)	C11	C9	C8	116.06(15)
O3	Si2	C31	113.35(10)	C4	C11	C9	112.88(16)
C29	Si2	C27	110.95(10)	O3	C12	C6	110.02(15)
C29	Si2	C31	109.26(10)	O3	C12	C13	109.05(15)
C31	Si2	C27	108.44(10)	C13	C12	C6	113.78(15)
C20	O2	Si1	127.86(12)	C14	C13	C12	110.83(16)
C12	O3	Si2	135.21(12)	C15	C13	C12	111.41(16)
C2	C1	C4	115.48(17)	C15	C13	C14	109.21(17)
C2	C1	C5	116.91(17)	C16	C15	C13	126.93(19)
C3	C1	C2	113.20(17)	C15	C16	C17	127.1(2)
C3	C1	C4	121.04(18)	C16	C17	C18	115.31(16)
C3	C1	C5	120.48(17)	C19	C18	C17	109.74(16)
C4	C1	C5	59.88(12)	C19	C18	C20	110.66(16)
C1	C4	C5	60.46(12)	C20	C18	C17	116.83(15)
C11	C4	C1	124.25(17)	O2	C20	C8	111.48(14)
C11	C4	C5	120.28(16)	O2	C20	C18	110.65(15)
C4	C5	C1	59.66(12)	C18	C20	C8	115.90(15)
C4	C5	C6	120.70(15)	C22	C21	Si1	117.55(16)
C6	C5	C1	122.54(16)	C24	C23	Si1	114.96(16)
C5	C6	C7	107.42(15)	C26	C25	Si1	117.63(15)
C5	C6	C12	110.34(15)	C28	C27	Si2	114.94(15)
C7	C6	C12	114.91(15)	C30	C29	Si2	115.87(15)
O1	C7	C6	121.59(16)	C32	C31	Si2	116.59(16)

Table 6 Torsion Angles for ca080223_1_1.

A	B	C	D	Angle/°	A	B	C	D	Angle/°
Si1	O2	C20	C8	140.18(13)	C7	C8	C9	C11	-56.1(2)
Si1	O2	C20	C18	-89.29(17)	C7	C8	C20	O2	64.9(2)
Si2	O3	C12	C6	130.67(15)	C7	C8	C20	C18	-62.9(2)
Si2	O3	C12	C13	-103.88(18)	C8	C9	C11	C4	78.8(2)
O1	C7	C8	C9	-115.24(19)	C9	C8	C20	O2	-69.63(19)
O1	C7	C8	C20	111.37(19)	C9	C8	C20	C18	162.61(15)
O2	Si1	C21	C22	-155.07(17)	C10	C9	C11	C4	-47.5(2)

Table 6 Torsion Angles for ca080223_1_1.

A	B	C	D	Angle/˚	A	B	C	D	Angle/˚
O2	Si1	C23	C24	-178.96(14)	C11	C4	C5	C1	-114.7(2)
O2	Si1	C25	C26	-72.46(17)	C11	C4	C5	C6	-2.6(3)
O3	Si2	C27	C28	34.3(2)	C12	C6	C7	O1	-29.6(2)
O3	Si2	C29	C30	-64.07(18)	C12	C6	C7	C8	153.61(16)
O3	Si2	C31	C32	-78.9(2)	C12	C13	C15	C16	107.1(2)
O3	C12	C13	C14	62.5(2)	C13	C15	C16	C17	-0.7(3)
O3	C12	C13	C15	-175.67(15)	C14	C13	C15	C16	-130.1(2)
C1	C4	C5	C6	112.17(19)	C15	C16	C17	C18	-126.5(2)
C1	C4	C11	C9	-138.93(18)	C16	C17	C18	C19	-179.40(16)
C1	C5	C6	C7	138.22(17)	C16	C17	C18	C20	53.6(2)
C1	C5	C6	C12	-95.8(2)	C17	C18	C20	O2	-61.9(2)
C2	C1	C4	C5	107.64(19)	C17	C18	C20	C8	66.3(2)
C2	C1	C4	C11	-143.98(19)	C19	C18	C20	O2	171.58(15)
C2	C1	C5	C4	-105.3(2)	C19	C18	C20	C8	-60.2(2)
C2	C1	C5	C6	145.57(18)	C20	C8	C9	C10	-153.53(15)
C3	C1	C4	C5	-109.6(2)	C20	C8	C9	C11	79.44(19)
C3	C1	C4	C11	-1.2(3)	C21	Si1	O2	C20	172.55(15)
C3	C1	C5	C4	110.5(2)	C21	Si1	C23	C24	-66.88(18)
C3	C1	C5	C6	1.3(3)	C21	Si1	C25	C26	173.00(15)
C4	C1	C5	C6	-109.16(19)	C23	Si1	O2	C20	-68.78(16)
C4	C5	C6	C7	66.8(2)	C23	Si1	C21	C22	88.40(19)
C4	C5	C6	C12	-167.29(16)	C23	Si1	C25	C26	49.95(18)
C5	C1	C4	C11	108.38(19)	C25	Si1	O2	C20	53.22(17)
C5	C4	C11	C9	-66.0(2)	C25	Si1	C21	C22	-32.4(2)
C5	C6	C7	O1	93.6(2)	C25	Si1	C23	C24	55.09(18)
C5	C6	C7	C8	-83.20(19)	C27	Si2	O3	C12	122.26(18)
C5	C6	C12	O3	-51.75(19)	C27	Si2	C29	C30	52.87(19)
C5	C6	C12	C13	-174.45(15)	C27	Si2	C31	C32	162.35(18)
C6	C7	C8	C9	61.7(2)	C29	Si2	O3	C12	-118.32(18)
C6	C7	C8	C20	-71.7(2)	C29	Si2	C27	C28	-83.02(19)
C6	C12	C13	C14	-174.26(17)	C29	Si2	C31	C32	41.3(2)
C6	C12	C13	C15	-52.4(2)	C31	Si2	O3	C12	2.7(2)
C7	C6	C12	O3	69.9(2)	C31	Si2	C27	C28	156.99(17)
C7	C6	C12	C13	-52.8(2)	C31	Si2	C29	C30	172.38(17)
C7	C8	C9	C10	70.93(19)					

Table 7 Hydrogen Atom Coordinates (Å×10[4]) and Isotropic Displacement Parameters (Å[2]×10[3]) for ca080223_1_1.

Atom	*x*	*y*	*z*	U(eq)
H2A	11128.62	3115.18	9823.41	42
H2B	9953.15	2148.15	9359.93	42
H2C	9734.18	3148.02	10061.69	42
H3A	9433.12	5409.59	9576.19	41
H3B	9405.98	5762.4	8569.65	41
H3C	10802.59	5380.76	9306.71	41
H4	9952.88	2601.89	7889.19	22
H5	7895.55	2688.74	8204.14	21
H6	7605.14	5273.99	7663.42	19
H8	6958.37	4077.37	5387.62	19
H9	9331.72	4401.6	5787.6	22
H10A	8391.67	2180.55	6379.07	33
H10B	9817.25	2294.67	6188.18	33
H10C	8437.4	2461.63	5376.81	33
H11A	10795.98	4209.75	7190.95	24
H11B	9693.61	5161.89	7324.69	24
H12	6480.49	4861.57	8712.79	21
H13	4355.18	4945.51	7007.16	26
H14A	3514.95	4655.81	8245.5	43
H14B	3105.1	6006.94	7832.88	43
H14C	4335.91	5838.8	8745.98	43
H15	5627.9	7141.17	7919.45	27
H16	5627.82	7877.43	6613.88	28
H17A	4042.2	6798.18	5335.31	27
H17B	4648.89	5535.25	5825.89	27
H18	6058.24	7262.43	5048.31	23
H19A	5136.99	4849.38	4423.03	39
H19B	6022.3	5700.21	3977.3	39
H19C	4528.43	6121.22	3941.62	39
H20	7915.9	5995.41	5304.11	20
H21A	8310.29	8248.1	7874.3	31
H21B	9893.05	7971.05	8145.36	31
H22A	8688.47	10311.51	7537.94	49
H22B	10275.12	10041.04	7771.89	49
H22C	9570.13	9990.78	8554.61	49
H23A	11020.44	6787.35	6859.95	31
H23B	10397.3	6997.97	5794.05	31
H24A	11190.76	9062.36	5959.98	44
H24B	12427.36	8132.75	6369.22	44
H24C	11788.95	8874.79	7031.97	44
H25A	7235.05	9279.6	5727.57	28
H25B	8691.62	9883.88	5893.11	28
H26A	8924.64	8543.62	4674.65	42

Table 7 Hydrogen Atom Coordinates (Å×10⁴) and Isotropic Displacement Parameters (Å²×10³) for ca080223_1_1.

Atom	*x*	*y*	*z*	U(eq)
H26B	7856.66	9648.43	4364.84	42
H26C	7338.9	8273.34	4447.25	42
H27A	3385.18	1311.67	9005.81	33
H27B	2956.61	2728.09	8820.89	33
H28A	3016.15	2488.43	7297.42	52
H28B	1907.13	1615	7510.49	52
H28C	3324.41	1049.15	7494.5	52
H29A	7394.67	1404.32	9355.74	29
H29B	6372.61	686.35	9765.17	29
H30A	5243.69	-158.53	8335.25	52
H30B	6824.05	-471.35	8592.67	52
H30C	6190.65	613.34	7889.16	52
H31A	5304.65	4258.43	9955.66	35
H31B	5204.18	3021.66	10490.33	35
H32A	7581.12	2730.45	10816.04	57
H32B	7245.5	4006.75	11222.25	57
H32C	7663.18	4004.58	10312.02	57

Experimental

Single crystals of $C_{32}H_{60}O_3Si_2$ **[ca080223_1_1]** were **[]**. A suitable crystal was selected and **[]** on a **XtaLAB Synergy, Dualflex, Pilatus 200K** diffractometer. The crystal was kept at 100.0(1) K during data collection. Using Olex2 [1], the structure was solved with the SHELXT [2] structure solution program using Intrinsic Phasing and refined with the SHELXL [3] refinement package using Least Squares minimisation.

1. Dolomanov, O.V., Bourhis, L.J., Gildea, R.J, Howard, J.A.K. & Puschmann, H. (2009), J. Appl. Cryst. 42, 339-341.
2. Sheldrick, G.M. (2015). Acta Cryst. A71, 3-8.
3. Sheldrick, G.M. (2015). Acta Cryst. C71, 3-8.

Crystal structure determination of [ca080223_1_1]

Crystal Data for $C_{32}H_{60}O_3Si_2$ (M =548.98 g/mol): monoclinic, space group P2$_1$ (no. 4), a = 10.37740(10) Å, b = 10.82020(10) Å, c = 15.52420(10) Å, β = 107.8970(10)°, V = 1658.79(3) Å^3, Z = 2, T = 100.0(1) K, μ(Cu Kα) = 1.178 mm^{-1}, $Dcalc$ = 1.099 g/cm^3, 51224 reflections measured (8.954° ≤ 2Θ ≤ 159.674°), 7033 unique (R_{int} = 0.0399, R_{sigma} = 0.0211) which were used in all calculations. The final R_1 was 0.0286 (I > 2σ(I)) and wR_2 was 0.0764 (all data).

Refinement model description

Number of restraints - 1, number of constraints - unknown.

Details:

```
1. Fixed Uiso
 At 1.2 times of:
  All C(H) groups, All C(H,H) groups
 At 1.5 times of:
  All C(H,H,H) groups
2.a Ternary CH refined with riding coordinates:
 C4(H4), C5(H5), C6(H6), C8(H8), C9(H9), C12(H12), C13(H13), C18(H18), C20(H20)
2.b Secondary CH2 refined with riding coordinates:
 C11(H11A,H11B), C17(H17A,H17B), C21(H21A,H21B), C23(H23A,H23B), C25(H25A,
 H25B), C27(H27A,H27B), C29(H29A,H29B), C31(H31A,H31B)
2.c Aromatic/amide H refined with riding coordinates:
 C15(H15), C16(H16)
2.d Idealised Me refined as rotating group:
 C2(H2A,H2B,H2C), C3(H3A,H3B,H3C), C10(H10A,H10B,H10C), C14(H14A,H14B,H14C),
```

C19(H19A,H19B,H19C), C22(H22A,H22B,H22C), C24(H24A,H24B,H24C), C26(H26A,H26B, H26C), C28(H28A,H28B,H28C), C30(H30A,H30B,H30C), C32(H32A,H32B,H32C)

Rearrangement product γ-lactone **14**:

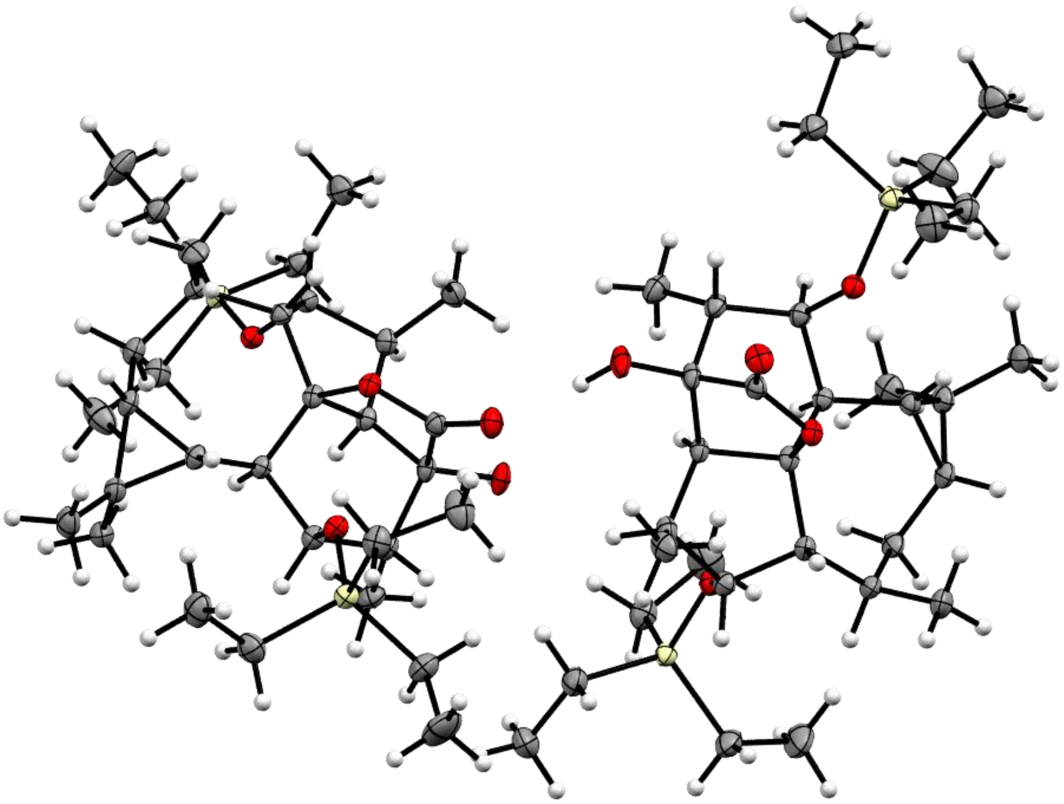

Table 1 Crystal data and structure refinement for ca290323_2_1.

Identification code	ca290323_2_1
Empirical formula	$C_{32}H_{58}O_5Si_2$
Formula weight	578.96
Temperature/K	100.0(1)
Crystal system	orthorhombic
Space group	$P2_12_12_1$
a/Å	9.63390(10)
b/Å	22.8529(2)
c/Å	30.0711(2)
α/°	90
β/°	90
γ/°	90
Volume/Å^3	6620.53(10)
Z	8
ρ_{calc}g/cm^3	1.162
μ/mm^{-1}	1.254
F(000)	2544.0
Crystal size/mm^3	0.268 × 0.083 × 0.026
Radiation	Cu Kα (λ = 1.54184)

2Θ range for data collection/°	7.038 to 140.15
Index ranges	$-11 \le h \le 10$, $-27 \le k \le 27$, $-36 \le l \le 36$
Reflections collected	125567
Independent reflections	12505 [R_{int} = 0.0646, R_{sigma} = 0.0271]
Data/restraints/parameters	12505/2/731
Goodness-of-fit on F^2	1.084
Final R indexes [I>=2σ (I)]	R_1 = 0.0290, wR_2 = 0.0745
Final R indexes [all data]	R_1 = 0.0325, wR_2 = 0.0778
Largest diff. peak/hole / e $Å^{-3}$	0.26/-0.23
Flack parameter	-0.006(7)

Table 2 Fractional Atomic Coordinates (×10^4) and Equivalent Isotropic Displacement Parameters ($Å^2$×10^3) for ca290323_2_1. U_{eq} is defined as 1/3 of the trace of the orthogonalised U_{IJ} tensor.

Atom	*x*	*y*	*z*	U(eq)
Si1A	5422.4(6)	2866.2(3)	7415.6(2)	17.78(13)
Si2A	-898.1(6)	5886.5(2)	7533.6(2)	16.02(12)
O1A	4602.4(15)	3498.7(6)	7423.9(5)	17.3(3)
O2A	5923.1(16)	4501.6(7)	6773.1(5)	22.7(3)
O3A	3505.7(17)	4516.4(7)	6262.2(5)	21.7(3)
O4A	4592.1(15)	4683.6(6)	7366.9(4)	16.4(3)
O5A	443.7(15)	5440.1(6)	7488.6(5)	17.2(3)
C1A	4617(3)	2349.0(11)	7826.6(8)	26.6(5)
C2A	5204(3)	2394.6(12)	8301.6(8)	32.4(6)
C3A	7235(2)	3051.2(11)	7578.6(8)	24.9(5)
C4A	8049(3)	3381.9(13)	7223.4(9)	35.7(6)
C5A	5391(3)	2541.0(10)	6844.2(8)	25.3(5)
C6A	6256(4)	1976.4(13)	6804.5(10)	45.8(8)
C7A	3175(2)	3610.7(9)	7323.4(6)	15.5(4)
C8A	3032(2)	3759.5(9)	6821.7(7)	16.5(4)
C9A	1604(2)	3591.6(10)	6639.9(7)	21.4(5)
C10A	3387(2)	4409.6(10)	6722.8(7)	16.5(4)
C11A	2419(2)	4791.7(9)	6999.1(6)	14.6(4)
C12A	3080(2)	4703.1(9)	7464.5(6)	14.5(4)
C13A	2627(2)	4099.0(9)	7640.1(6)	14.5(4)
C14A	4786(2)	4532.0(9)	6939.1(7)	17.3(4)
C15A	2833(2)	5287.1(9)	7716.3(7)	16.0(4)
C16A	2460(2)	5316.7(9)	8215.7(7)	18.7(4)
C17A	3795(3)	5354.0(11)	8493.4(7)	25.7(5)
C18A	1493(2)	4830.7(10)	8386.9(7)	19.1(4)
C19A	2293(2)	4281.2(10)	8494.0(7)	18.9(4)
C20A	1827(2)	3660.2(10)	8406.3(7)	19.2(4)

Table 2 Fractional Atomic Coordinates (×10^4) and Equivalent Isotropic Displacement Parameters (Å^2×10^3) for ca290323_2_1. U_{eq} is defined as 1/3 of the trace of the orthogonalised U_{IJ} tensor.

Atom	*x*	*y*	*z*	U(eq)
C21A	416(2)	3528.5(10)	8213.1(7)	21.5(5)
C22A	2293(3)	3206.2(11)	8742.2(7)	26.4(5)
C23A	2959(2)	3938.7(9)	8117.8(7)	17.0(4)
C24A	2280(2)	5457.5(9)	6934.8(7)	17.9(4)
C25A	3575(2)	5787.3(10)	6777.6(8)	23.9(5)
C26A	1839(2)	5627.9(9)	7411.2(7)	17.4(4)
C27A	-1067(2)	6380.5(10)	7040.4(7)	21.5(5)
C28A	-42(3)	6891.1(11)	7011.5(8)	29.6(6)
C29A	-675(3)	6338.8(10)	8048.8(7)	23.2(5)
C30A	-1858(3)	6769.8(12)	8133.2(9)	36.3(6)
C31A	-2470(2)	5412.9(10)	7572.4(7)	22.7(5)
C32A	-2376(3)	4908.3(13)	7902.5(9)	36.3(6)
Si1B	10597.5(6)	6504.4(3)	5261.1(2)	19.32(13)
Si2B	4133.9(6)	3525.4(3)	5128.7(2)	17.53(13)
O1B	9798.3(15)	5865.9(7)	5274.2(5)	18.8(3)
O2B	10925.6(17)	4860.5(7)	5970.5(5)	23.3(3)
O3B	8410.4(18)	4892.0(8)	6432.1(5)	24.7(4)
O4B	9702.7(15)	4655.3(7)	5358.0(4)	17.0(3)
O5B	5521.2(16)	3952.5(6)	5154.5(5)	17.4(3)
C1B	9703(3)	7000.8(12)	4854.8(10)	36.2(6)
C2B	10494(3)	7540.6(11)	4704.1(8)	31.2(6)
C3B	12386(2)	6329.2(11)	5069.4(7)	24.4(5)
C4B	13237(3)	5970.6(13)	5403.6(9)	33.8(6)
C5B	10630(3)	6827.2(11)	5834.5(8)	28.3(5)
C6B	11519(3)	7378.8(12)	5889.1(8)	33.1(6)
C7B	8358(2)	5752.1(9)	5345.6(7)	17.2(4)
C8B	8121(2)	5629.1(10)	5849.6(7)	18.6(4)
C9B	6700(2)	5837.0(11)	6010.0(8)	25.4(5)
C10B	8396(2)	4979.0(10)	5968.1(7)	18.5(4)
C11B	7443(2)	4600.7(9)	5680.0(6)	15.7(4)
C12B	8207(2)	4651.8(9)	5230.5(6)	15.0(4)
C13B	7876(2)	5254.4(9)	5027.9(6)	15.6(4)
C14B	9822(2)	4828.8(9)	5783.1(7)	17.8(4)
C15B	7965(2)	4053.7(10)	4991.8(6)	16.2(4)
C16B	7662(2)	3988.8(10)	4486.4(7)	19.1(4)
C17B	9000(3)	3828.2(11)	4239.0(8)	26.4(5)
C18B	6903(2)	4505.1(10)	4268.8(7)	20.7(5)
C19B	7874(2)	5010.0(10)	4177.5(7)	20.2(5)
C20B	7485(2)	5648.4(10)	4210.5(7)	20.4(5)
C21B	6018(2)	5834.8(11)	4320.4(8)	25.4(5)
C22B	8192(3)	6053.2(10)	3880.6(7)	25.8(5)
C23B	8419(2)	5369.0(9)	4563.3(6)	17.0(4)

Table 2 Fractional Atomic Coordinates (×10^4) and Equivalent Isotropic Displacement Parameters (Å^2×10^3) for ca290323_2_1. U_{eq} is defined as 1/3 of the trace of the orthogonalised U_{IJ} tensor.

Atom	*x*	*y*	*z*	U(eq)
C24B	7213(2)	3944.9(10)	5760.7(7)	18.2(4)
C25B	8430(3)	3597.7(11)	5955.6(7)	24.5(5)
C26B	6867(2)	3749.4(9)	5282.1(7)	16.7(4)
C27B	4005(3)	3087.8(11)	5653.8(7)	25.2(5)
C28B	2686(3)	2712.7(11)	5681.6(8)	31.7(6)
C29B	4246(3)	2998.1(11)	4652.2(7)	28.8(5)
C30B	4071(4)	3241.2(14)	4185.2(8)	41.8(7)
C31B	2626(2)	4030.2(11)	5071.7(8)	25.5(5)
C32B	2844(3)	4561.3(13)	4769.1(10)	38.6(6)

Table 3 Anisotropic Displacement Parameters (Å^2×10^3) for ca290323_2_1. The Anisotropic displacement factor exponent takes the form: -2π^2[h^{2}a*^{2}U$_{11}$+2hka*b*U$_{12}$+…].

Atom	U_{11}	U_{22}	U_{33}	U_{23}	U_{13}	U_{12}
Si1A	18.5(3)	18.1(3)	16.7(3)	0.1(2)	-0.8(2)	2.9(2)
Si2A	17.6(3)	17.7(3)	12.8(3)	-1.4(2)	-0.2(2)	1.2(2)
O1A	16.0(7)	19.7(7)	16.4(7)	-1.2(6)	-1.3(5)	2.7(6)
O2A	16.8(8)	29.3(9)	21.9(8)	0.7(6)	4.5(6)	0.6(7)
O3A	23.1(9)	31.3(9)	10.6(7)	3.3(6)	1.8(6)	5.5(7)
O4A	13.8(7)	20.8(8)	14.7(7)	-1.5(6)	-0.6(5)	-0.1(6)
O5A	17.7(8)	17.9(7)	16.0(7)	-1.0(6)	0.7(6)	-0.8(6)
C1A	27.1(13)	24.8(12)	28.0(12)	5.5(9)	0.0(10)	0.9(10)
C2A	41.2(16)	33.4(14)	22.5(12)	6.8(10)	1.4(10)	5.7(11)
C3A	21.4(12)	31.2(13)	22.1(11)	2.1(9)	-2.6(9)	4.5(9)
C4A	23.4(14)	52.3(17)	31.4(14)	8.4(12)	-2.2(10)	-5.0(12)
C5A	27.1(13)	25.7(12)	23.0(11)	-5.2(9)	-0.4(9)	3.6(10)
C6A	62(2)	35.1(16)	40.8(16)	-13.3(13)	2.1(14)	18.9(14)
C7A	14.1(10)	17.2(11)	15.2(10)	-0.2(8)	-1.2(7)	2.1(8)
C8A	17.3(11)	20.0(11)	12.2(10)	-2.6(8)	-0.5(8)	2.0(8)
C9A	23.3(12)	21.8(12)	18.9(11)	-3.6(9)	-3.9(8)	-0.3(9)
C10A	16.2(11)	23.0(11)	10.4(10)	1.0(8)	0.0(7)	1.3(8)
C11A	14.1(10)	17.9(10)	11.7(9)	0.2(8)	-1.8(7)	0.7(8)
C12A	12.2(10)	18.9(11)	12.5(9)	-0.3(8)	-1.4(7)	0.1(8)
C13A	14.5(10)	16.2(10)	12.8(10)	-0.3(8)	-0.2(7)	0.6(8)
C14A	19.4(12)	17.1(11)	15.3(10)	2.0(8)	0.8(8)	0.5(8)
C15A	17.0(11)	16.0(11)	15.0(10)	-1.0(8)	-1.4(8)	-1.3(8)
C16A	22.3(12)	18.7(11)	15.1(10)	-4.1(8)	-2.3(8)	1.4(9)
C17A	29.0(14)	28.8(13)	19.2(11)	-1.7(9)	-9.5(9)	-7.6(10)
C18A	20.5(11)	23.2(12)	13.7(10)	-1.5(8)	1.8(8)	1.9(9)
C19A	22.1(12)	23.0(12)	11.5(10)	-1.2(8)	0.5(8)	0.6(9)

Table 3 Anisotropic Displacement Parameters (Å^2×10^3) for ca290323_2_1. The Anisotropic displacement factor exponent takes the form: -2π^2[h^{2}a*^{2}U$_{11}$+2hka*b*U$_{12}$+…].

Atom	U_{11}	U_{22}	U_{33}	U_{23}	U_{13}	U_{12}
C20A	21.9(12)	20.9(11)	14.9(10)	0.8(8)	2.7(8)	0.1(9)
C21A	24.1(12)	21.3(11)	19.0(10)	-0.4(9)	3.6(8)	-3.3(9)
C22A	34.1(14)	26.8(13)	18.1(11)	5.3(9)	3.7(9)	2.8(10)
C23A	18.0(11)	19.3(11)	13.6(10)	0.3(8)	-1.3(8)	1.1(8)
C24A	17.1(11)	21.2(11)	15.2(10)	1.3(8)	0.4(8)	0.9(8)
C25A	25.4(13)	22.4(12)	23.9(12)	4.3(9)	4.3(9)	-1.4(9)
C26A	17.8(11)	16.7(10)	17.6(10)	-1.2(8)	1.1(8)	-0.6(8)
C27A	26.3(12)	21.7(12)	16.4(10)	-1.6(8)	-2.3(8)	4.3(9)
C28A	40.8(16)	25.0(13)	23.0(12)	5.2(9)	-1.5(10)	-0.7(11)
C29A	28.7(13)	25.1(12)	15.8(10)	-2.2(8)	0.5(8)	1.9(10)
C30A	42.9(17)	39.9(16)	26.2(13)	-10.9(11)	3.5(11)	14.0(13)
C31A	20.6(12)	26.3(12)	21.3(11)	0.3(9)	-0.2(8)	0.1(9)
C32A	31.6(15)	41.9(16)	35.4(14)	14.2(12)	-9.7(11)	-13.1(12)
Si1B	19.1(3)	20.4(3)	18.5(3)	0.4(2)	-1.4(2)	-2.8(2)
Si2B	16.5(3)	21.7(3)	14.4(3)	-1.9(2)	0.5(2)	-2.0(2)
O1B	17.0(8)	21.6(8)	17.8(7)	-1.4(6)	0.3(6)	-2.2(6)
O2B	17.8(8)	30.3(9)	21.8(8)	2.9(6)	-5.3(6)	-2.2(7)
O3B	24.4(9)	39.7(10)	10.0(7)	1.3(6)	-0.6(6)	-9.5(7)
O4B	13.1(7)	23.2(8)	14.6(7)	0.3(6)	-0.3(5)	-0.2(6)
O5B	17.0(8)	20.8(8)	14.3(7)	1.1(6)	-0.2(5)	-0.6(6)
C1B	31.5(15)	29.6(14)	47.5(16)	12.5(12)	-15.0(12)	-4.0(11)
C2B	37.3(15)	26.9(13)	29.3(13)	6.8(10)	-2.6(11)	1.5(11)
C3B	23.1(12)	30.7(13)	19.4(11)	0.8(9)	-0.1(9)	-4.3(10)
C4B	25.4(14)	40.7(15)	35.4(14)	4.6(12)	-2.3(10)	4.7(11)
C5B	29.0(14)	28.8(13)	27.1(12)	-7.3(10)	4.5(10)	-6.3(10)
C6B	39.6(16)	31.7(14)	28.0(13)	-9.2(11)	1.6(11)	-11.9(11)
C7B	15.9(11)	19.4(11)	16.2(11)	-0.2(8)	0.2(8)	0.4(8)
C8B	17.6(11)	23.4(11)	14.6(10)	-4.3(8)	0.2(8)	-2.8(9)
C9B	22.5(12)	30.5(13)	23.1(12)	-6.5(10)	3.6(9)	1.7(10)
C10B	17.8(11)	27.7(12)	9.9(10)	0.6(8)	0.3(8)	-4.1(9)
C11B	14.3(11)	22.0(11)	10.9(9)	-0.2(8)	0.5(7)	-1.7(8)
C12B	10.2(10)	21.7(11)	13.0(9)	0.1(8)	-0.3(7)	-0.6(8)
C13B	15.0(11)	18.0(11)	13.7(10)	-0.3(8)	-0.9(7)	0.1(8)
C14B	20.8(12)	18.7(11)	13.9(10)	3.2(8)	-1.0(8)	-1.4(8)
C15B	16.1(11)	18.4(11)	14.0(10)	0.2(8)	1.4(8)	0.5(8)
C16B	24.4(12)	19.4(11)	13.4(10)	-2.2(8)	2.4(8)	-4.3(9)
C17B	30.9(14)	28.2(13)	20.2(11)	-2.1(9)	9.7(10)	-1.4(10)
C18B	24.7(12)	25.2(12)	12.2(10)	-0.5(8)	-1.9(8)	-5.7(9)
C19B	26.2(12)	23.4(12)	10.9(10)	-0.1(8)	0.6(8)	-2.9(9)
C20B	25.6(12)	22.7(12)	12.8(10)	2.8(8)	-2.2(8)	-1.7(9)
C21B	26.5(13)	26.8(13)	22.9(11)	4.8(9)	-6.0(9)	0.8(10)
C22B	36.7(14)	24.2(12)	16.6(11)	3.9(9)	-0.3(9)	-3.7(10)

Table 3 Anisotropic Displacement Parameters (Å^2×10^3) for ca290323_2_1. The Anisotropic displacement factor exponent takes the form: -2π^2[h^{2}a*^{2}U$_{11}$+2hka*b*U$_{12}$+…].

Atom	U11	U22	U33	U23	U13	U12
C23B	18.8(11)	19.3(11)	12.9(10)	0.8(8)	0.2(8)	-3.5(9)
C24B	17.3(11)	24.2(12)	13.1(10)	2.2(8)	-0.1(8)	-3.6(8)
C25B	28.6(13)	26.5(13)	18.6(11)	6.5(9)	-5.5(9)	-1.4(10)
C26B	17.7(11)	18.2(11)	14.3(10)	0.6(8)	-1.4(8)	0.5(8)
C27B	27.3(13)	28.7(13)	19.5(11)	-0.7(9)	1.9(9)	-1.8(10)
C28B	35.7(15)	30.1(14)	29.2(13)	0.3(10)	8.9(10)	-8.6(11)
C29B	29.8(14)	33.7(14)	22.9(12)	-9.2(10)	3.2(10)	-6.4(10)
C30B	52.6(19)	52.0(18)	20.9(13)	-8.3(11)	3.4(12)	-15.2(14)
C31B	20.6(12)	32.1(14)	23.7(12)	-1.4(10)	-0.4(9)	2.2(10)
C32B	31.7(16)	38.1(16)	46.2(16)	8.8(13)	-0.2(12)	9.2(12)

Table 4 Bond Lengths for ca290323_2_1.

Atom	Atom	Length/Å	Atom	Atom	Length/Å
Si1A	O1A	1.6476(15)	Si1B	O1B	1.6504(16)
Si1A	C1A	1.878(2)	Si1B	C1B	1.877(3)
Si1A	C3A	1.862(2)	Si1B	C3B	1.861(2)
Si1A	C5A	1.872(2)	Si1B	C5B	1.875(2)
Si2A	O5A	1.6524(16)	Si2B	O5B	1.6569(16)
Si2A	C27A	1.871(2)	Si2B	C27B	1.873(2)
Si2A	C29A	1.875(2)	Si2B	C29B	1.875(2)
Si2A	C31A	1.865(2)	Si2B	C31B	1.863(2)
O1A	C7A	1.431(3)	O1B	C7B	1.428(3)
O2A	C14A	1.206(3)	O2B	C14B	1.205(3)
O3A	C10A	1.411(2)	O3B	C10B	1.409(3)
O4A	C12A	1.486(3)	O4B	C12B	1.491(2)
O4A	C14A	1.345(2)	O4B	C14B	1.343(3)
O5A	C26A	1.430(3)	O5B	C26B	1.429(3)
C1A	C2A	1.540(3)	C1B	C2B	1.519(4)
C3A	C4A	1.525(3)	C3B	C4B	1.534(3)
C5A	C6A	1.541(3)	C5B	C6B	1.533(3)
C7A	C8A	1.553(3)	C7B	C8B	1.558(3)
C7A	C13A	1.559(3)	C7B	C13B	1.556(3)
C8A	C9A	1.529(3)	C8B	C9B	1.528(3)
C8A	C10A	1.553(3)	C8B	C10B	1.550(3)
C10A	C11A	1.524(3)	C10B	C11B	1.530(3)
C10A	C14A	1.522(3)	C10B	C14B	1.522(3)
C11A	C12A	1.551(3)	C11B	C12B	1.544(3)
C11A	C24A	1.540(3)	C11B	C24B	1.534(3)
C12A	C13A	1.541(3)	C12B	C13B	1.539(3)
C12A	C15A	1.553(3)	C12B	C15B	1.561(3)

Table 4 Bond Lengths for ca290323_2_1.

Atom	Atom	Length/Å	Atom	Atom	Length/Å
C13A	C23A	1.517(3)	C13B	C23B	1.515(3)
C15A	C16A	1.546(3)	C15B	C16B	1.555(3)
C15A	C26A	1.538(3)	C15B	C26B	1.538(3)
C16A	C17A	1.536(3)	C16B	C17B	1.533(3)
C16A	C18A	1.538(3)	C16B	C18B	1.535(3)
C18A	C19A	1.508(3)	C18B	C19B	1.510(3)
C19A	C20A	1.512(3)	C19B	C20B	1.510(3)
C19A	C23A	1.518(3)	C19B	C23B	1.515(3)
C20A	C21A	1.509(3)	C20B	C21B	1.512(3)
C20A	C22A	1.516(3)	C20B	C22B	1.518(3)
C20A	C23A	1.532(3)	C20B	C23B	1.531(3)
C24A	C25A	1.532(3)	C24B	C25B	1.532(3)
C24A	C26A	1.544(3)	C24B	C26B	1.543(3)
C27A	C28A	1.531(3)	C27B	C28B	1.535(3)
C29A	C30A	1.528(3)	C29B	C30B	1.520(4)
C31A	C32A	1.524(3)	C31B	C32B	1.531(4)

Table 5 Bond Angles for ca290323_2_1.

Atom	Atom	Atom	Angle/°	Atom	Atom	Atom	Angle/°
O1A	Si1A	C1A	110.13(10)	O1B	Si1B	C1B	109.63(10)
O1A	Si1A	C3A	104.26(10)	O1B	Si1B	C3B	104.43(10)
O1A	Si1A	C5A	110.75(9)	O1B	Si1B	C5B	109.49(10)
C3A	Si1A	C1A	110.92(11)	C3B	Si1B	C1B	110.70(12)
C3A	Si1A	C5A	110.31(11)	C3B	Si1B	C5B	110.73(11)
C5A	Si1A	C1A	110.33(11)	C5B	Si1B	C1B	111.62(13)
O5A	Si2A	C27A	112.06(9)	O5B	Si2B	C27B	109.19(9)
O5A	Si2A	C29A	108.57(9)	O5B	Si2B	C29B	111.59(10)
O5A	Si2A	C31A	106.37(9)	O5B	Si2B	C31B	105.56(10)
C27A	Si2A	C29A	109.41(10)	C27B	Si2B	C29B	107.75(11)
C31A	Si2A	C27A	109.22(10)	C31B	Si2B	C27B	110.88(11)
C31A	Si2A	C29A	111.18(11)	C31B	Si2B	C29B	111.87(12)
C7A	O1A	Si1A	127.91(13)	C7B	O1B	Si1B	128.16(14)
C14A	O4A	C12A	109.41(15)	C14B	O4B	C12B	109.22(15)
C26A	O5A	Si2A	124.29(13)	C26B	O5B	Si2B	123.56(13)
C2A	C1A	Si1A	114.57(18)	C2B	C1B	Si1B	117.06(19)
C4A	C3A	Si1A	114.21(17)	C4B	C3B	Si1B	114.00(16)
C6A	C5A	Si1A	113.25(18)	C6B	C5B	Si1B	115.55(17)
O1A	C7A	C8A	109.25(16)	O1B	C7B	C8B	108.72(17)
O1A	C7A	C13A	108.93(16)	O1B	C7B	C13B	109.30(16)
C8A	C7A	C13A	113.99(16)	C13B	C7B	C8B	114.95(17)
C7A	C8A	C10A	112.09(17)	C9B	C8B	C7B	112.45(18)
C9A	C8A	C7A	111.86(18)	C9B	C8B	C10B	112.23(19)

Table 5 Bond Angles for ca290323_2_1.

Atom	Atom	Atom	Angle/°	Atom	Atom	Atom	Angle/°
C9A	C8A	C10A	111.68(18)	C10B	C8B	C7B	111.81(17)
O3A	C10A	C8A	111.79(17)	O3B	C10B	C8B	111.37(18)
O3A	C10A	C11A	119.07(17)	O3B	C10B	C11B	119.12(18)
O3A	C10A	C14A	108.42(17)	O3B	C10B	C14B	108.72(17)
C11A	C10A	C8A	108.00(17)	C11B	C10B	C8B	108.01(17)
C14A	C10A	C8A	106.79(17)	C14B	C10B	C8B	106.63(18)
C14A	C10A	C11A	101.72(16)	C14B	C10B	C11B	101.97(17)
C10A	C11A	C12A	99.56(16)	C10B	C11B	C12B	99.61(16)
C10A	C11A	C24A	123.45(18)	C10B	C11B	C24B	123.32(18)
C24A	C11A	C12A	106.15(16)	C24B	C11B	C12B	106.33(16)
O4A	C12A	C11A	103.19(15)	O4B	C12B	C11B	103.65(15)
O4A	C12A	C13A	108.56(16)	O4B	C12B	C13B	107.30(16)
O4A	C12A	C15A	105.81(16)	O4B	C12B	C15B	105.51(16)
C11A	C12A	C15A	105.35(16)	C11B	C12B	C15B	105.37(16)
C13A	C12A	C11A	108.05(16)	C13B	C12B	C11B	108.38(17)
C13A	C12A	C15A	124.00(16)	C13B	C12B	C15B	124.77(17)
C12A	C13A	C7A	109.63(16)	C12B	C13B	C7B	110.44(16)
C23A	C13A	C7A	109.53(16)	C23B	C13B	C7B	109.69(17)
C23A	C13A	C12A	118.77(17)	C23B	C13B	C12B	116.61(17)
O2A	C14A	O4A	122.46(19)	O2B	C14B	O4B	122.6(2)
O2A	C14A	C10A	128.07(18)	O2B	C14B	C10B	127.73(19)
O4A	C14A	C10A	109.47(17)	O4B	C14B	C10B	109.69(17)
C16A	C15A	C12A	123.16(17)	C16B	C15B	C12B	124.12(18)
C26A	C15A	C12A	103.88(16)	C26B	C15B	C12B	103.75(16)
C26A	C15A	C16A	114.38(17)	C26B	C15B	C16B	112.51(17)
C17A	C16A	C15A	109.64(19)	C17B	C16B	C15B	109.84(19)
C17A	C16A	C18A	111.43(18)	C17B	C16B	C18B	112.20(18)
C18A	C16A	C15A	115.75(17)	C18B	C16B	C15B	115.65(18)
C19A	C18A	C16A	111.30(19)	C19B	C18B	C16B	111.70(19)
C18A	C19A	C20A	126.4(2)	C18B	C19B	C23B	119.29(17)
C18A	C19A	C23A	119.09(18)	C20B	C19B	C18B	124.9(2)
C20A	C19A	C23A	60.76(14)	C20B	C19B	C23B	60.82(14)
C19A	C20A	C22A	116.00(19)	C19B	C20B	C21B	121.2(2)
C19A	C20A	C23A	59.82(14)	C19B	C20B	C22B	115.75(19)
C21A	C20A	C19A	121.46(19)	C19B	C20B	C23B	59.76(14)
C21A	C20A	C22A	112.8(2)	C21B	C20B	C22B	112.98(19)
C21A	C20A	C23A	120.42(17)	C21B	C20B	C23B	121.04(18)
C22A	C20A	C23A	116.79(19)	C22B	C20B	C23B	116.31(19)
C13A	C23A	C19A	119.48(18)	C13B	C23B	C19B	119.53(18)
C13A	C23A	C20A	119.11(18)	C13B	C23B	C20B	120.52(19)
C19A	C23A	C20A	59.42(14)	C19B	C23B	C20B	59.42(14)
C11A	C24A	C26A	99.01(16)	C11B	C24B	C26B	99.59(16)
C25A	C24A	C11A	116.99(19)	C25B	C24B	C11B	117.11(18)
C25A	C24A	C26A	112.73(18)	C25B	C24B	C26B	111.85(18)

Table 5 Bond Angles for ca290323_2_1.

Atom	Atom	Atom	Angle/°	Atom	Atom	Atom	Angle/°
O5A	C26A	C15A	109.65(17)	O5B	C26B	C15B	108.94(16)
O5A	C26A	C24A	109.50(17)	O5B	C26B	C24B	110.63(17)
C15A	C26A	C24A	104.74(17)	C15B	C26B	C24B	104.47(16)
C28A	C27A	Si2A	116.67(16)	C28B	C27B	Si2B	113.52(17)
C30A	C29A	Si2A	114.03(17)	C30B	C29B	Si2B	117.71(19)
C32A	C31A	Si2A	115.58(17)	C32B	C31B	Si2B	116.05(18)

Table 6 Torsion Angles for ca290323_2_1.

A	B	C	D	Angle/°	A	B	C	D	Angle/°
Si1A	O1A	C7A	C8A	-91.35(19)	Si1B	O1B	C7B	C8B	-94.05(19)
Si1A	O1A	C7A	C13A	143.55(14)	Si1B	O1B	C7B	C13B	139.76(15)
Si2A	O5A	C26A	C15A	136.18(14)	Si2B	O5B	C26B	C15B	141.31(14)
Si2A	O5A	C26A	C24A	-109.42(17)	Si2B	O5B	C26B	C24B	-104.39(17)
O1A	Si1A	C1A	C2A	-87.3(2)	O1B	Si1B	C1B	C2B	-164.0(2)
O1A	Si1A	C3A	C4A	-71.6(2)	O1B	Si1B	C3B	C4B	-67.4(2)
O1A	Si1A	C5A	C6A	174.69(19)	O1B	Si1B	C5B	C6B	171.58(18)
O1A	C7A	C8A	C9A	151.82(17)	O1B	C7B	C8B	C9B	147.64(18)
O1A	C7A	C8A	C10A	-81.8(2)	O1B	C7B	C8B	C10B	-85.0(2)
O1A	C7A	C13A	C12A	80.40(19)	O1B	C7B	C13B	C12B	83.9(2)
O1A	C7A	C13A	C23A	-51.6(2)	O1B	C7B	C13B	C23B	-46.0(2)
O3A	C10A	C11A	C12A	-157.16(18)	O3B	C10B	C11B	C12B	-156.78(19)
O3A	C10A	C11A	C24A	-40.5(3)	O3B	C10B	C11B	C24B	-39.9(3)
O3A	C10A	C14A	O2A	-27.1(3)	O3B	C10B	C14B	O2B	-28.3(3)
O3A	C10A	C14A	O4A	153.43(17)	O3B	C10B	C14B	O4B	152.79(17)
O4A	C12A	C13A	C7A	-49.4(2)	O4B	C12B	C13B	C7B	-51.8(2)
O4A	C12A	C13A	C23A	77.5(2)	O4B	C12B	C13B	C23B	74.3(2)
O4A	C12A	C15A	C16A	-109.7(2)	O4B	C12B	C15B	C16B	-110.8(2)
O4A	C12A	C15A	C26A	118.25(16)	O4B	C12B	C15B	C26B	119.26(16)
O5A	Si2A	C27A	C28A	-75.52(19)	O5B	Si2B	C27B	C28B	175.25(16)
O5A	Si2A	C29A	C30A	-179.50(18)	O5B	Si2B	C29B	C30B	-72.1(2)
O5A	Si2A	C31A	C32A	47.7(2)	O5B	Si2B	C31B	C32B	39.6(2)
C1A	Si1A	O1A	C7A	-61.18(18)	C1B	Si1B	O1B	C7B	-53.0(2)
C1A	Si1A	C3A	C4A	169.87(19)	C1B	Si1B	C3B	C4B	174.70(19)
C1A	Si1A	C5A	C6A	-63.1(2)	C1B	Si1B	C5B	C6B	-66.9(2)
C3A	Si1A	O1A	C7A	179.76(16)	C3B	Si1B	O1B	C7B	-171.64(16)
C3A	Si1A	C1A	C2A	27.6(2)	C3B	Si1B	C1B	C2B	-49.3(3)
C3A	Si1A	C5A	C6A	59.8(2)	C3B	Si1B	C5B	C6B	57.0(2)
C5A	Si1A	O1A	C7A	61.13(18)	C5B	Si1B	O1B	C7B	69.76(19)
C5A	Si1A	C1A	C2A	150.14(18)	C5B	Si1B	C1B	C2B	74.5(2)

Table 6 Torsion Angles for ca290323_2_1.

A	B	C	D	Angle/°	A	B	C	D	Angle/°
C5A	Si1A	C3A	C4A	47.3(2)	C5B	Si1B	C3B	C4B	50.4(2)
C7A	C8A	C10A	O3A	169.09(17)	C7B	C8B	C10B	O3B	170.03(17)
C7A	C8A	C10A	C11A	-58.1(2)	C7B	C8B	C10B	C11B	-57.4(2)
C7A	C8A	C10A	C14A	50.7(2)	C7B	C8B	C10B	C14B	51.6(2)
C7A	C13A	C23A	C19A	-168.12(18)	C7B	C13B	C23B	C19B	-168.67(19)
C7A	C13A	C23A	C20A	-98.9(2)	C7B	C13B	C23B	C20B	-98.8(2)
C8A	C7A	C13A	C12A	-41.9(2)	C8B	C7B	C13B	C12B	-38.7(2)
C8A	C7A	C13A	C23A	-173.84(17)	C8B	C7B	C13B	C23B	-168.55(18)
C8A	C10A	C11A	C12A	73.99(19)	C8B	C10B	C11B	C12B	74.9(2)
C8A	C10A	C11A	C24A	-169.31(18)	C8B	C10B	C11B	C24B	-168.17(19)
C8A	C10A	C14A	O2A	93.5(3)	C8B	C10B	C14B	O2B	91.9(3)
C8A	C10A	C14A	O4A	-86.0(2)	C8B	C10B	C14B	O4B	-87.0(2)
C9A	C8A	C10A	O3A	-64.5(2)	C9B	C8B	C10B	O3B	-62.5(2)
C9A	C8A	C10A	C11A	68.4(2)	C9B	C8B	C10B	C11B	70.1(2)
C9A	C8A	C10A	C14A	177.10(17)	C9B	C8B	C10B	C14B	179.00(17)
C10A	C11A	C12A	O4A	37.45(19)	C10B	C11B	C12B	O4B	36.73(19)
C10A	C11A	C12A	C13A	-77.40(19)	C10B	C11B	C12B	C13B	-77.04(19)
C10A	C11A	C12A	C15A	148.22(17)	C10B	C11B	C12B	C15B	147.36(17)
C10A	C11A	C24A	C25A	-31.4(3)	C10B	C11B	C24B	C25B	-31.6(3)
C10A	C11A	C24A	C26A	-152.71(19)	C10B	C11B	C24B	C26B	-152.33(19)
C11A	C10A	C14A	O2A	-153.4(2)	C11B	C10B	C14B	O2B	-154.9(2)
C11A	C10A	C14A	O4A	27.1(2)	C11B	C10B	C14B	O4B	26.1(2)
C11A	C12A	C13A	C7A	61.8(2)	C11B	C12B	C13B	C7B	59.6(2)
C11A	C12A	C13A	C23A	-171.26(18)	C11B	C12B	C13B	C23B	-174.34(17)
C11A	C12A	C15A	C16A	141.4(2)	C11B	C12B	C15B	C16B	139.9(2)
C11A	C12A	C15A	C26A	9.4(2)	C11B	C12B	C15B	C26B	10.0(2)
C11A	C24A	C26A	O5A	-72.0(2)	C11B	C24B	C26B	O5B	-71.9(2)
C11A	C24A	C26A	C15A	45.5(2)	C11B	C24B	C26B	C15B	45.2(2)
C12A	O4A	C14A	O2A	177.82(19)	C12B	O4B	C14B	O2B	178.8(2)
C12A	O4A	C14A	C10A	-2.7(2)	C12B	O4B	C14B	C10B	-2.2(2)
C12A	C11A	C24A	C25A	82.1(2)	C12B	C11B	C24B	C25B	82.0(2)
C12A	C11A	C24A	C26A	-39.2(2)	C12B	C11B	C24B	C26B	-38.7(2)
C12A	C13A	C23A	C19A	64.9(3)	C12B	C13B	C23B	C19B	64.9(3)
C12A	C13A	C23A	C20A	134.2(2)	C12B	C13B	C23B	C20B	134.7(2)
C12A	C15A	C16A	C17A	90.0(2)	C12B	C15B	C16B	C17B	98.6(2)
C12A	C15A	C16A	C18A	-37.0(3)	C12B	C15B	C16B	C18B	-29.6(3)
C12A	C15A	C26A	O5A	82.77(19)	C12B	C15B	C26B	O5B	83.60(19)
C12A	C15A	C26A	C24A	-34.6(2)	C12B	C15B	C26B	C24B	-34.6(2)
C13A	C7A	C8A	C9A	-86.1(2)	C13B	C7B	C8B	C9B	-89.5(2)
C13A	C7A	C8A	C10A	40.3(2)	C13B	C7B	C8B	C10B	37.8(2)

Table 6 Torsion Angles for ca290323_2_1.

A	B	C	D	Angle/˚	A	B	C	D	Angle/˚
C13A	C12A	C15A	C16A	16.4(3)	C13B	C12B	C15B	C16B	13.8(3)
C13A	C12A	C15A	C26A	-115.6(2)	C13B	C12B	C15B	C26B	-116.1(2)
C14A	O4A	C12A	C11A	-22.5(2)	C14B	O4B	C12B	C11B	-22.5(2)
C14A	O4A	C12A	C13A	91.96(18)	C14B	O4B	C12B	C13B	92.06(18)
C14A	O4A	C12A	C15A	-132.95(16)	C14B	O4B	C12B	C15B	-133.01(16)
C14A	C10A	C11A	C12A	-38.19(19)	C14B	C10B	C11B	C12B	-37.2(2)
C14A	C10A	C11A	C24A	78.5(2)	C14B	C10B	C11B	C24B	79.7(2)
C15A	C12A	C13A	C7A	-174.41(18)	C15B	C12B	C13B	C7B	-175.63(18)
C15A	C12A	C13A	C23A	-47.5(3)	C15B	C12B	C13B	C23B	-49.5(3)
C15A	C16A	C18A	C19A	82.8(2)	C15B	C16B	C18B	C19B	78.2(2)
C16A	C15A	C26A	O5A	-54.2(2)	C16B	C15B	C26B	O5B	-53.0(2)
C16A	C15A	C26A	C24A	-171.58(17)	C16B	C15B	C26B	C24B	-171.24(17)
C16A	C18A	C19A	C20A	-141.1(2)	C16B	C18B	C19B	C20B	-144.7(2)
C16A	C18A	C19A	C23A	-67.6(3)	C16B	C18B	C19B	C23B	-71.6(3)
C17A	C16A	C18A	C19A	-43.4(2)	C17B	C16B	C18B	C19B	-48.9(2)
C18A	C19A	C20A	C21A	-3.1(3)	C18B	C19B	C20B	C21B	-3.1(3)
C18A	C19A	C20A	C22A	-146.5(2)	C18B	C19B	C20B	C22B	-146.2(2)
C18A	C19A	C20A	C23A	106.3(2)	C18B	C19B	C20B	C23B	107.1(2)
C18A	C19A	C23A	C13A	-9.4(3)	C18B	C19B	C23B	C13B	-5.9(3)
C18A	C19A	C23A	C20A	-117.8(2)	C18B	C19B	C23B	C20B	-116.0(2)
C19A	C20A	C23A	C13A	-109.0(2)	C19B	C20B	C23B	C13B	-108.4(2)
C20A	C19A	C23A	C13A	108.4(2)	C20B	C19B	C23B	C13B	110.1(2)
C21A	C20A	C23A	C13A	2.0(3)	C21B	C20B	C23B	C13B	2.0(3)
C21A	C20A	C23A	C19A	111.0(2)	C21B	C20B	C23B	C19B	110.5(2)
C22A	C20A	C23A	C13A	145.1(2)	C22B	C20B	C23B	C13B	145.7(2)
C22A	C20A	C23A	C19A	-105.9(2)	C22B	C20B	C23B	C19B	-105.8(2)
C23A	C19A	C20A	C21A	-109.3(2)	C23B	C19B	C20B	C21B	-110.2(2)
C23A	C19A	C20A	C22A	107.2(2)	C23B	C19B	C20B	C22B	106.8(2)
C24A	C11A	C12A	O4A	-91.64(18)	C24B	C11B	C12B	O4B	-92.33(18)
C24A	C11A	C12A	C13A	153.51(17)	C24B	C11B	C12B	C13B	153.90(17)
C24A	C11A	C12A	C15A	19.1(2)	C24B	C11B	C12B	C15B	18.3(2)
C25A	C24A	C26A	O5A	163.60(17)	C25B	C24B	C26B	O5B	163.68(17)
C25A	C24A	C26A	C15A	-78.9(2)	C25B	C24B	C26B	C15B	-79.2(2)
C26A	C15A	C16A	C17A	-142.31(19)	C26B	C15B	C16B	C17B	-135.13(19)
C26A	C15A	C16A	C18A	90.6(2)	C26B	C15B	C16B	C18B	96.6(2)
C27A	Si2A	O5A	C26A	54.94(17)	C27B	Si2B	O5B	C26B	48.54(17)
C27A	Si2A	C29A	C30A	57.9(2)	C27B	Si2B	C29B	C30B	168.1(2)
C27A	Si2A	C31A	C32A	168.87(19)	C27B	Si2B	C31B	C32B	157.70(19)
C29A	Si2A	O5A	C26A	-66.02(17)	C29B	Si2B	O5B	C26B	-70.47(17)
C29A	Si2A	C27A	C28A	45.0(2)	C29B	Si2B	C27B	C28B	-63.4(2)
C29A	Si2A	C31A	C32A	-70.3(2)	C29B	Si2B	C31B	C32B	-82.0(2)

Table 6 Torsion Angles for ca290323_2_1.

A	B	C	D	Angle/°	A	B	C	D	Angle/°
C31A	Si2A	O5A	C26A	174.24(15)	C31B	Si2B	O5B	C26B	167.80(15)
C31A	Si2A	C27A	C28A	166.87(17)	C31B	Si2B	C27B	C28B	59.3(2)
C31A	Si2A	C29A	C30A	-62.8(2)	C31B	Si2B	C29B	C30B	46.0(2)

Table 7 Hydrogen Atom Coordinates (Å×10^4) and Isotropic Displacement Parameters (Å^2×10^3) for ca290323_2_1.

Atom	*x*	*y*	*z*	U(eq)
H3A	2690(20)	4646(12)	6167(9)	33
H1AA	3605.41	2424.17	7837.55	32
H1AB	4751.12	1943.59	7718.75	32
H2AA	6187.65	2284.63	8299.95	49
H2AB	4690.55	2130.56	8498.62	49
H2AC	5109.14	2797.76	8408.41	49
H3AA	7208.37	3291.55	7852.39	30
H3AB	7734.74	2684.43	7649.71	30
H4AA	8142.88	3135.93	6958.15	54
H4AB	8972.06	3479.89	7338.05	54
H4AC	7553.84	3742.39	7145.74	54
H5AA	4417.3	2453.7	6762.13	30
H5AB	5750.18	2832.82	6629.92	30
H6AA	6212.42	1831.19	6498.2	69
H6AB	5882.56	1679.38	7006.86	69
H6AC	7223.14	2059.56	6883.23	69
H7A	2635.99	3245.18	7383.37	19
H8A	3730.42	3515.8	6659.79	20
H9AA	881.56	3789.59	6813.05	32
H9AB	1480.07	3167.07	6662.58	32
H9AC	1536.03	3710.7	6327.6	32
H11A	1471.81	4613.1	6998.48	18
H13A	1592.54	4089.52	7615.18	17
H15A	3735.73	5500.86	7692.24	19
H16A	1956.36	5694.53	8261.33	22
H17A	4371.79	5009.24	8435.27	39
H17B	3555.85	5368.41	8809.94	39
H17C	4308.65	5708.34	8412.06	39
H18A	1006.71	4968.68	8657.21	23
H18B	784.69	4742.75	8158	23
H19A	2901.82	4320.03	8761.72	23
H21A	-206.81	3395.23	8450.13	32
H21B	501.69	3221.56	7987.23	32
H21C	35.31	3883.33	8076.49	32
H22A	3225.16	3304.76	8849.46	40

Table 7 Hydrogen Atom Coordinates (Å×10^4) and Isotropic Displacement Parameters (Å^2×10^3) for ca290323_2_1.

Atom	*x*	*y*	*z*	U(eq)
H22B	2311.61	2819.78	8601.02	40
H22C	1645.01	3200.59	8993.27	40
H23A	3926.46	3798	8173.96	20
H24A	1496.68	5539.06	6725.69	21
H25A	3361.21	6204.99	6750.67	36
H25B	3867.15	5634.74	6487.73	36
H25C	4325.24	5732.91	6993.81	36
H26A	1932.85	6059.12	7459.07	21
H27A	-963.41	6140.15	6768.54	26
H27B	-2019.24	6543.22	7038.77	26
H28A	-289.73	7142.22	6759.56	44
H28B	899.45	6738.62	6969.31	44
H28C	-80.24	7119.21	7287.2	44
H29A	-596.21	6073.54	8307.85	28
H29B	205.06	6560.28	8025.45	28
H30A	-1920.04	7045.78	7884.56	55
H30B	-1679.7	6985.19	8409.12	55
H30C	-2734.68	6554.85	8160.07	55
H31A	-3270.12	5661.44	7655.79	27
H31B	-2662.88	5249.2	7273.99	27
H32A	-1620.73	4645.05	7814.79	54
H32B	-3254.44	4692.26	7904.19	54
H32C	-2191.58	5063.05	8200.61	54
H3B	7580(30)	4758(13)	6523(10)	37
H1BA	9466.52	6768.21	4587.64	43
H1BB	8819.08	7131.29	4990.06	43
H2BA	9948.43	7750.28	4479.97	47
H2BB	11385.53	7422.28	4575.44	47
H2BC	10658.43	7797.35	4959.76	47
H3BA	12326.48	6108.43	4786.8	29
H3BB	12883.08	6699.64	5007.77	29
H4BA	14138.95	5872.15	5272.88	51
H4BB	12736.24	5609.89	5476.71	51
H4BC	13378.93	6200.62	5674.9	51
H5BA	9665.45	6922.29	5922.12	34
H5BB	10976.24	6525.95	6043.4	34
H6BA	11117.29	7697.86	5713.53	50
H6BB	12464.88	7299.46	5785.34	50
H6BC	11543.68	7491.64	6203.42	50
H7B	7830.38	6114.31	5266.3	21
H8B	8828.11	5864.86	6014.63	22
H9BA	6625.91	6261.35	5969.16	38
H9BB	6590.26	5741.77	6325.78	38

Table 7 Hydrogen Atom Coordinates (Å×10^4) and Isotropic Displacement Parameters (Å^2×10^3) for ca290323_2_1.

Atom	*x*	*y*	*z*	U(eq)
H9BC	5971.35	5641.36	5837.84	38
H11B	6519.65	4797.63	5654.49	19
H13B	6841.64	5281.95	5010.11	19
H15B	8838.24	3826.85	5042.54	19
H16B	7031.14	3643.59	4456.17	23
H17D	9700.29	4132.19	4289.79	40
H17E	8808.16	3797.59	3919.83	40
H17F	9348.4	3452.29	4349.25	40
H18C	6479.25	4374.06	3985.88	25
H18D	6146.71	4638.51	4467.36	25
H19B	8585.74	4925.05	3944.28	24
H21D	5482.39	5874.09	4044.99	38
H21E	6038.26	6211.47	4476	38
H21F	5583.63	5539.54	4511.39	38
H22D	9160.94	5933.22	3842.64	39
H22E	8158.59	6455.72	3992.38	39
H22F	7711.42	6031.83	3593.74	39
H23B	9423.48	5477.28	4545.04	20
H24B	6376.93	3890.26	5953.34	22
H25D	8157.98	3187.19	5991.6	37
H25E	8683.02	3761.25	6245.77	37
H25F	9227.44	3622.51	5754.16	37
H26B	6932.15	3314.42	5252.85	20
H27C	4825.12	2828.64	5676.09	30
H27D	4025.81	3358.32	5910.84	30
H28D	2663.81	2438.2	5431.36	48
H28E	1867	2966.75	5669.49	48
H28F	2684.06	2493.71	5961.83	48
H29C	5160.76	2801.89	4667.41	35
H29D	3529.58	2693.28	4697.47	35
H30D	4210.55	2927.06	3967.87	63
H30E	4755.79	3551.15	4134.48	63
H30F	3133.67	3402.02	4152.02	63
H31C	1828.06	3803.36	4956.66	31
H31D	2372.08	4173.19	5371.61	31
H32D	3005.98	4428.52	4463.65	58
H32E	3648.47	4785.36	4872.68	58
H32F	2015.23	4810	4777.37	58

Experimental

Single crystals of $C_{32}H_{58}O_5Si_2$ **[ca290323_2_1]** were **[]**. A suitable crystal was selected and **[]** on a **XtaLAB Synergy, Dualflex, Pilatus 300K** diffractometer. The crystal was kept at 100.0(1) K during data collection. Using Olex2 [1], the structure was solved with the SHELXT [2] structure solution program using Intrinsic Phasing and refined with the SHELXL [3] refinement package using Least Squares minimisation.

1. Dolomanov, O.V., Bourhis, L.J., Gildea, R.J, Howard, J.A.K. & Puschmann, H. (2009), J. Appl. Cryst. 42, 339-341.
2. Sheldrick, G.M. (2015). Acta Cryst. A71, 3-8.
3. Sheldrick, G.M. (2015). Acta Cryst. C71, 3-8.

Crystal structure determination of [ca290323_2_1]

Crystal Data for $C_{32}H_{58}O_5Si_2$ (M =578.96 g/mol): orthorhombic, space group $P2_12_12_1$ (no. 19), a = 9.63390(10) Å, b = 22.8529(2) Å, c = 30.0711(2) Å, V = 6620.53(10) Å^3, Z = 8, T = 100.0(1) K, μ(Cu Kα) = 1.254 mm^{-1}, $Dcalc$ = 1.162 g/cm^3, 125567 reflections measured (7.038° ≤ 2Θ ≤ 140.15°), 12505 unique (R_{int} = 0.0646, R_{sigma} = 0.0271) which were used in all calculations. The final R_1 was 0.0290 (I > 2σ(I)) and wR_2 was 0.0778 (all data).

Refinement model description

Number of restraints - 2, number of constraints - unknown.

Details:

```
1. Fixed Uiso
 At 1.2 times of:
  All C(H) groups, All C(H,H) groups
 At 1.5 times of:
  All C(H,H,H) groups, All O(H) groups
2. Restrained distances
 O3B-H3B = O3A-H3A
 0.93 with sigma of 0.02
3.a Ternary CH refined with riding coordinates:
 C7A(H7A), C8A(H8A), C11A(H11A), C13A(H13A), C15A(H15A), C16A(H16A),
 C19A(H19A), C23A(H23A), C24A(H24A), C26A(H26A), C7B(H7B), C8B(H8B), C11B(H11B),
  C13B(H13B), C15B(H15B), C16B(H16B), C19B(H19B), C23B(H23B), C24B(H24B),
 C26B(H26B)
3.b Secondary CH2 refined with riding coordinates:
 C1A(H1AA,H1AB), C3A(H3AA,H3AB), C5A(H5AA,H5AB), C18A(H18A,H18B), C27A(H27A,
 H27B), C29A(H29A,H29B), C31A(H31A,H31B), C1B(H1BA,H1BB), C3B(H3BA,H3BB),
 C5B(H5BA,H5BB), C18B(H18C,H18D), C27B(H27C,H27D), C29B(H29C,H29D), C31B(H31C,
 H31D)
3.c Idealised Me refined as rotating group:
 C2A(H2AA,H2AB,H2AC), C4A(H4AA,H4AB,H4AC), C6A(H6AA,H6AB,H6AC), C9A(H9AA,H9AB,
 H9AC), C17A(H17A,H17B,H17C), C21A(H21A,H21B,H21C), C22A(H22A,H22B,H22C),
 C25A(H25A,H25B,H25C), C28A(H28A,H28B,H28C), C30A(H30A,H30B,H30C), C32A(H32A,
 H32B,H32C), C2B(H2BA,H2BB,H2BC), C4B(H4BA,H4BB,H4BC), C6B(H6BA,H6BB,H6BC),
 C9B(H9BA,H9BB,H9BC), C17B(H17D,H17E,H17F), C21B(H21D,H21E,H21F), C22B(H22D,
 H22E,H22F), C25B(H25D,H25E,H25F), C28B(H28D,H28E,H28F), C30B(H30D,H30E,H30F),
 C32B(H32D,H32E,H32F)
```

Sources

(1) Classen, M. J.; Böcker, M. N. A.; Roth, R.; Amberg, W. M.; Carreira, E. M. Enantioselective Total Synthesis of (+)-Euphorikanin A. *J. Am. Chem. Soc.* **2021**, *143*, 8261–8265.
(2) Satoh, T.; Kaneko, Y.; Okuda, T.; Uwaya, S.; Yamakawa, K. Studies on the Terpenoids and Related Alicyclic Compounds. XXXV. Studies Directed toward a Total Synthesis of Ingenol Esters : Synthesis of the C/D-Ring Moiety of Ingenol Esters from (+)-3-Carene via Tin (IV) Chloride-Promoted Intramolecular Directed Aldol Reaction. *Chem. Pharm. BULL.* **1984**, *32*, 3452–3460.
(3) Chakraborty, T. K.; Suresh, V. R. Synthetic studies toward potent cytotoxic agents amphidinolides G and H: Synthesis of the entire C_{15} C_{26} moiety of the top half. *Tetrahedron Lett.* **1998**, *39*, 7775–7778.
(4) Nicolaou, K. C.; Schlawe, D.; Kim, D. W.; Longbottom, D. A.; de Noronha, R. G.; Lizos, D. E.; Manam, R. R.; Faulkner, D. J. Total Synthesis of Halipeptins: Isolation of Halipeptin D and Synthesis of Oxazoline Halipeptin Analogues. *Chem. Eur. J.* **2005**, *11*, 6197–6211.
(5) Konno, K.; Fujishima, T.; Maki, S.; Liu, Z.; Miura, D.; Chokki, M.; Ishizuka, S.; Yamaguchi, K.; Kan, Y.; Kurihara, M.; et al. Synthesis, Biological Evaluation, and Conformational Analysis of A-Ring Diastereomers of 2-Methyl-1,25-dihydroxyvitamin D3 and Their 20-Epimers: Unique Activity Profiles Depending on the Stereochemistry of the A-Ring and at C-20. *J. Med. Chem.* **2000**, *43*, 4247–4265.
(6) Li, N.-S.; Scharf, L.; Adams, E. J.; Piccirilli, J. A. Highly Stereocontrolled Total Synthesis of β-d-Mannosyl Phosphomycoketide: A Natural Product from Mycobacterium tuberculosis. *J. Org. Chem.* **2013**, *78*, 5970–5986.
(7) Kawamura, S.; Chu, H.; Felding, J.; Baran, P. S. Nineteen-step total synthesis of (+)-phorbol. *Nature* **2016**, *532*, 90–93.
(8) Fei, D.-Q.; Dong, L.-L.; Qi, F.-M.; Fan, G.-X.; Li, H.-H.; Li, Z.-Y.; Zhang, Z.-X. Euphorikanin A, a Diterpenoid Lactone with a Fused 5/6/7/3 Ring System from Euphorbia kansui. *Org. Lett.* **2016**, *18*, 2844–2847.
